# An Active Site Tyr Residue Guides the Regioselectivity of Lysine Hydroxylation by Nonheme Iron Lysine-4-hydroxylase Enzymes through Proton-Coupled Electron Transfer

**DOI:** 10.1021/jacs.3c14574

**Published:** 2024-04-18

**Authors:** Yuanxin Cao, Sam Hay, Sam P. de Visser

**Affiliations:** § Manchester Institute of Biotechnology, The University of Manchester, 131 Princess Street, Manchester M1 7DN, United Kingdom; & Department of Chemistry, The University of Manchester, Oxford Road, Manchester M13 9PL, United Kingdom; ⊥ Department of Chemical Engineering, The University of Manchester, Oxford Road, Manchester M13 9PL, United Kingdom

## Abstract

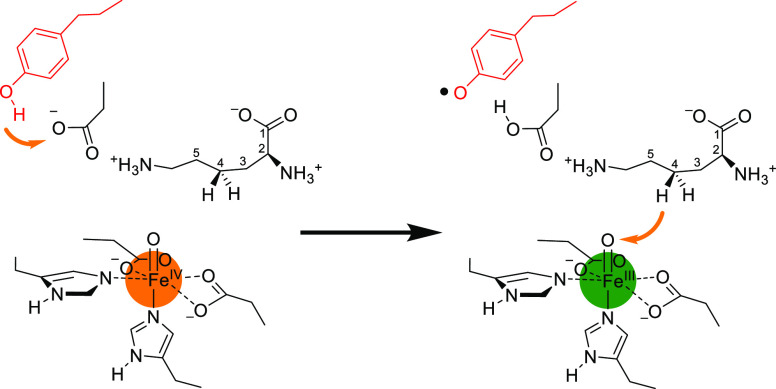

Lysine dioxygenase (KDO) is an important enzyme in human physiology involved in bioprocesses that trigger collagen cross-linking and blood pressure control. There are several KDOs in nature; however, little is known about the factors that govern the regio- and stereoselectivity of these enzymes. To understand how KDOs can selectively hydroxylate their substrate, we did a comprehensive computational study into the mechanisms and features of 4-lysine dioxygenase. In particular, we selected a snapshot from the MD simulation on KDO5 and created large QM cluster models (**A**, **B**, and **C**) containing 297, 312, and 407 atoms, respectively. The largest model predicts regioselectivity that matches experimental observation with rate-determining hydrogen atom abstraction from the C_4_–H position, followed by fast OH rebound to form 4-hydroxylysine products. The calculations show that in model **C**, the dipole moment is positioned along the C_4_–H bond of the substrate and, therefore, the electrostatic and electric field perturbations of the protein assist the enzyme in creating C_4_–H hydroxylation selectivity. Furthermore, an active site Tyr_233_ residue is identified that reacts through proton-coupled electron transfer akin to the axial Trp residue in cytochrome *c* peroxidase. Thus, upon formation of the iron(IV)-oxo species in the catalytic cycle, the Tyr_233_ phenol loses a proton to the nearby Asp_179_ residue, while at the same time, an electron is transferred to the iron to create an iron(III)-oxo active species. This charged tyrosyl residue directs the dipole moment along the C_4_–H bond of the substrate and guides the selectivity to the C_4_-hydroxylation of the substrate.

## Introduction

Lysine hydroxylases are vital enzymes for human health that trigger collagen cross-linking processes of protein chains but also have been linked to blood pressure control and cardiac development in embryos.^1−3^ However, high expression of lysine hydroxylases may lead to cancer development.^4,5^ Several lysine hydroxylases have been characterized in various species, and generally, they are highly stereo- and regioselective and typically hydroxylate a lysine residue at the C_3_-, C_4_-, or C_5_-position.^6−9^ Thus, some lysine hydroxylases bind a collagen or protein chain on the interface of an enzymatic dimer and hydroxylate individual lysine residues of the chain, while others operate on isolated lysine amino acids.^10^ As a consequence, these lysine hydroxylases show major differences in substrate activation and product distributions.

**Figure 1:**
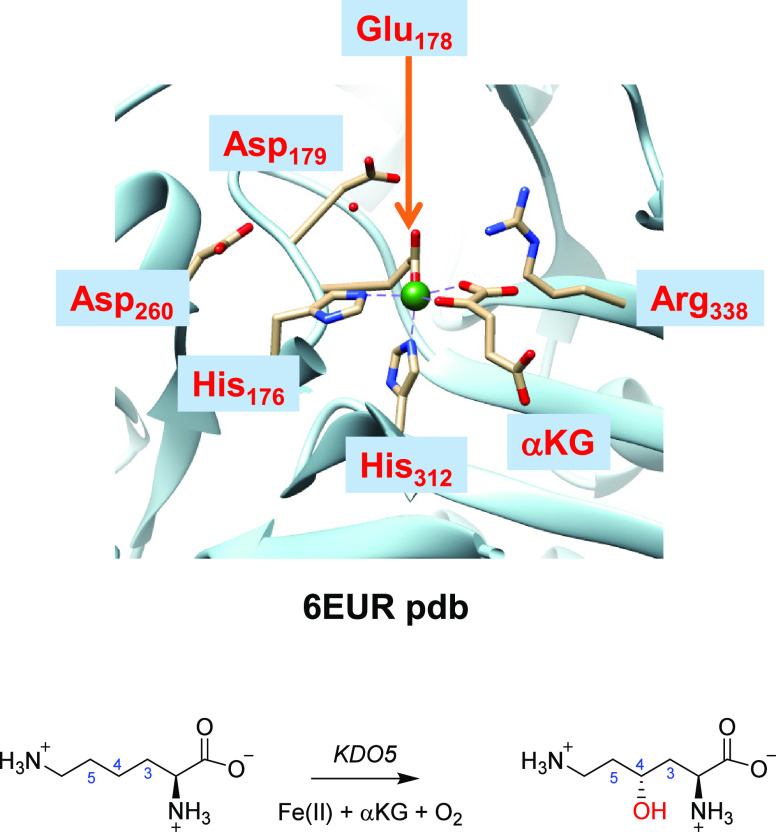
Active site of KDO5 (PDB ID: 6EUR) and the general reaction catalyzed by the enzyme.

One class of lysine hydroxylases is the nonheme iron/α-ketoglutarate (αKG, also called 2-oxoglutarate)-dependent lysine dioxygenases (KDOs). Nonheme iron and αKG-dependent dioxygenases are common enzymes in many biosystems involved in biosynthesis reactions of natural products, including hormones and antibiotics.^11−21^ They all have a central iron(II) atom that is bound to the protein through interactions with the side chains of two histidine and one carboxylate-based, e.g., Asp or Glu, residue. Indeed, the structure of KDO5 displayed in Figure 1 shows the facial 2-His/1-Glu binding orientation of iron(II), where the metal is covalently bound to His_176_, Glu_178_, and His_312_.^22,23^ The cosubstrate αKG binds as a bidentate ligand trans to the His_176_ and Glu_178_ groups. Substrate Lys is missing from the X-ray crystal structure, but the active site has a number of polar residues that are likely involved in substrate binding and positioning, namely, Asp_179_, Asp_260_, and Arg_338_.

There are various KDO isozymes labeled KDO1–KDO6 that all react with a free lysine substrate at a nonheme iron center. However, KDO1 gives *S*-3-hydroxylysine as a product, whereas the other isozymes react with a free lysine amino acid on the iron(II) center with αKG and dioxygen to form *R*-4-hydroxylysine regio- and stereoselectively. To understand this high regio- and stereoselectivity, we performed a detailed computational study of KDO5 enzymes. Similar to other nonheme iron and αKG-dependent dioxygenases, the reaction of dioxygen with αKG on a nonheme iron(II) center initially forms an iron(IV)-oxo species, succinate, and CO_2_.^12−21,24−26^ For various nonheme iron dioxygenases, the iron(IV)-oxo species has been experimentally trapped and characterized with spectroscopic methods. In all cases, the iron was found to be in a quintet spin ground state and reacts through hydrogen atom abstraction from the substrate.^27−30^ Despite the fact that the iron(IV)-oxo species of KDO5 has never been trapped and characterized, biochemical studies have been reported on its activity, substrate scope, and biochemistry.^31,32^ Moreover, engineered KDOs gave insight into substrate binding and orientation.^33^

To gain insight into the high selectivity of KDO5 enzymes, we decided to perform a computational study into lysine activation by KDO5 enzymes using the PDB 6EUR X-ray crystal structure as a starting point.^22,23^ The work shows that key hydrogen bonding and polar residues in the substrate binding pocket direct the local electric field and dipole moment and trigger a proton-coupled electron transfer from an active site Tyr residue to a neighboring Asp residue that guides the reaction to C_4_-hydroxylation even though it is not a thermodynamically favored pathway.

## Methods

### Model Setup

The model setup follows previous quantum cluster model calculations from our group and will be briefly summarized here.^34,35^ We started from the 6EUR PDB file,^22,23^ which is a substrate-devoid but iron(II)- and α-ketoglutarate (αKG)-bound enzymatic tetramer of KDO. We selected chain A of the protein for our work and removed glycerol molecules from the structure. The missing loop in the protein from residues 230–240 was inserted using a homology match found with the UniProt software package for patching missing loops from the AlphaFold model AF-J3BZS6-F1.^36^ Next, substrate lysine was docked into the substrate binding pocket using the Autodock Vina software package,^37^ and the most stable conformation was selected. Thereafter, hydrogen atoms were added in Chimera under pH 7 conditions,^38^ whereby all Lys and Arg residues of the protein chain were in their protonated states, while all Asp and Glu residues were in their deprotonated state. Histidine residues were manually inspected, and all internal histidine residues were taken in their singly protonated states. The structure was solvated with TIP3P-defined water molecules in a cubic box with a 10 Å radius from the outside of the protein.

### Molecular Dynamics Simulations

Parameters of the metal with its first-coordination sphere were obtained from the MCPB program, while the Amber force field was used on protein atoms.^39,40^ The enzyme structure was equilibrated for 1 ns and heated to 298 K over a period of 1 ns using the ff14SB force field.^41^ Thereafter, molecular dynamics simulation was performed for 50 ns in Amber without constraints under NVT conditions; see the Supporting Information for details.^40^ The MD simulation converged the protein to a stable structure, and an analysis of geometries during the MD simulation shows that the system is highly rigid and, particularly, the substrate is bound tightly within the substrate-binding pocket. Geometrically, the last point of the MD simulation shows only minor differences with respect to the original crystal structure coordinates as shown from the overlay of the two structures in Figure S4, Supporting Information.

### Cluster Model Setup

**Figure 2:**
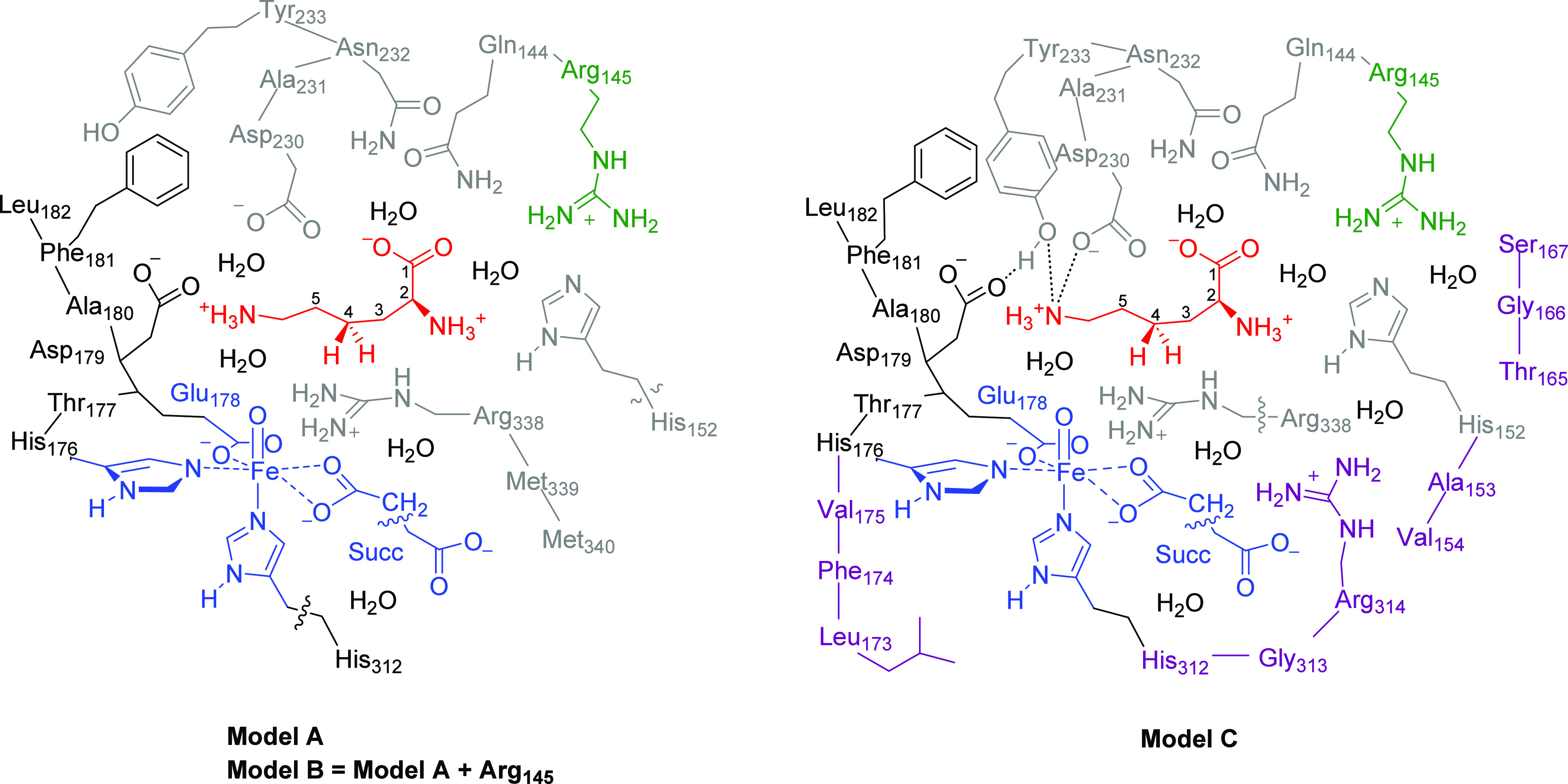
Cluster models **A**, **B**, and **C** of KDO5 as studied in this work. Model **C** is based on model **B** with the second-coordination sphere expanded, as highlighted in purple. Wiggly lines identify where bonds were cut and replaced by C–H.

After the MD simulation, the last step of the equilibrated structure was used to create several cluster models. These cluster models capture all interactions of the substrate and oxidant in the protein and have been shown to accurately reproduce the experimental structure, spectroscopic parameters, and kinetics as well as product distributions.^42−47^ The cluster models were created based on the first- and second-coordination spheres of the iron and substrate, and they are shown in Figure 2. We initially did exploratory calculations using cluster model **A** of 297 atoms and an overall charge of −1. Model **A** included the iron(IV)-oxo(succinate) group, where we truncated succinate to acetate. The axial histidine residue (His_312_) was shortened to methylimidazole, while the other protein ligands of the metal were part of the peptide chain His_176_-Thr_177_-Glu_178_-Asp_179_-Ala_180_-Phe_181_-Leu_182_ with Thr_177_ truncated to Gly. In addition, part of the substrate binding pocket was included in the model, namely, the chains Asp_230_-Ala_231_-Asn_232_-Tyr_233_ and Arg_338_-Met_339_-Met_340_ with the residues Ala_231_ and Met_339_ truncated to a glycine residue. The Gln_144_, His_152_, and Asp_260_ side chains were also included in the model, as well as four water molecules. We further expanded the model to 312 atoms with the amino acid residue Arg_145_ as it has been implicated in substrate recognition and may be involved in the assistance of pulling the substrate into the protein structure.^22^ Model **B** therefore has an overall neutral charge. Models **A** and **B** were calculated in the lowest energy singlet, triplet, quintet, and septet spin states. None of the models use constraints during the geometry optimizations. A comparison of the optimized geometries with crystal structure coordinates showed minor deviations with respect to the starting structures.

Finally, a larger model was created, namely, model **C** with 407 atoms, which included the iron(IV)-oxo(succinate) active site with succinate truncated to acetate. The environment was incorporated through the peptide chain from Leu_173_ to Leu_182_ and the protein chains Gln_144_-Arg_145_, His_152_-Ala_153_-Val_154_, Thr_165_-Gly_166_-Ser_167_, Asp_230_-Ala_231_-Asn_232_-Tyr_233_, and His_312_-Gly_313_-Arg_314_. The residues Phe_174_, Val_175_, Thr_177_, and Ala_231_ were truncated to Gly residues. The model also included the side chain of Arg_338_ and seven water molecules. Model **C** had an overall charge of +2 and was calculated in the quintet spin state only (see below). A final model **C2** was explored, which includes model **C** expanded with the full succinate group and the guanidinium group of Arg_334_.

### Computational Methods

The Gaussian-09 software package was used for all quantum chemical calculations discussed here.^48^ Following previous experience with cluster models of nonheme iron dioxygenases,^34,49^ we utilized the unrestricted B3LYP density functional method in combination with LANL2DZ (with electron core potential) on iron and 6-31G on the rest of the atoms, denoted basis set BS1.^50−54^ Dispersion corrections were included with the GD3 approach and Becke–Johnson damping factor.^55^ To correct the energetics, single point calculations with LACV3P+ (with electron core potential) on iron and 6-311+G* on the rest of the atoms (BS2) were performed. Frequency calculations were performed on all local minima and transition states, and it was confirmed that local minima had real frequencies only, while the transition states had a single imaginary mode for the expected vibration along the reaction coordinate. To test the effect of the basis set, we also did geometry optimizations with def2-SVP on all atoms followed by single point calculations with def2-TZVP. These calculations obtained results similar to those with basis sets BS1 and BS2. Test calculations using an implicit solvent model with a dielectric constant mimicking chlorobenzene were performed on key transition state structures for model **C**, which did not change the ordering of the transition states and predicted the same selectivity; see the Supporting Information.

## Results

### System Setup and MD Simulations

**Figure 3:**
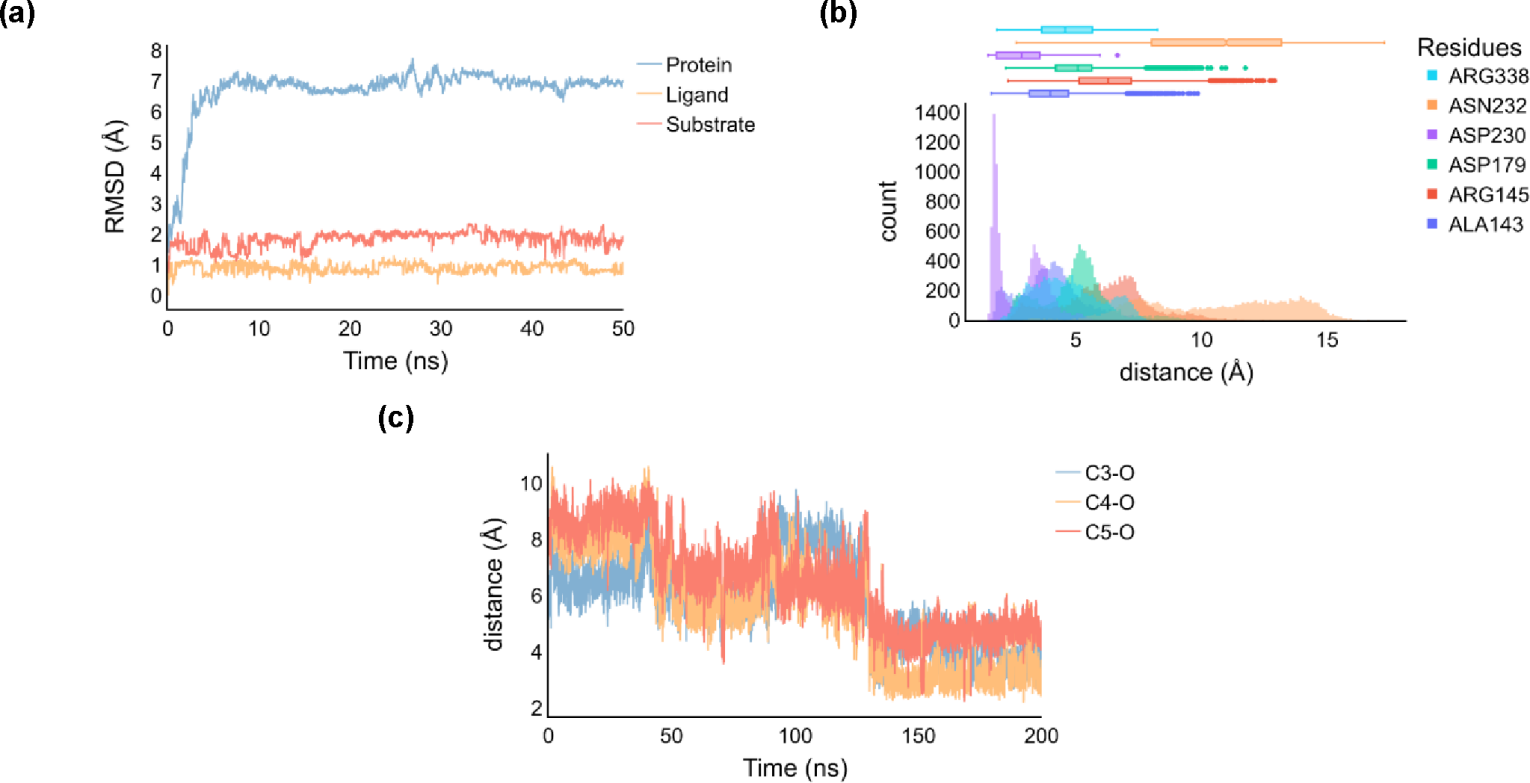
(a) RMSD plot of the MD simulation on a substrate-bound KDO5 structure. (b) Distance plots of key active site amino acid residues with respect to the substrate. (c) Substrate–oxidant C_3_–O, C_4_–O, and C_5_–O distances during the MD simulation.

We started the work with a set of molecular mechanics and molecular dynamics (MD) simulations on the protein structure based on chain A of the X-ray crystal structure of KDO5 solved with bound αKG (PDB: 6EUR). Initially, we docked substrate lysine into the active site; see the Supporting Information for details. The docking pose with the highest score was then selected for further setup and MD simulations. The root-mean-square deviation (RMSD) of the structure converges within a few nanoseconds of MD simulation as shown in Figure 3 and reaches a stable plateau, which applies to the RMSD of the protein, substrate, and ligand components. In general, the structure is highly rigid and little deviation between the crystal structure and the final structure of the MD simulation is seen. The only motion seen during the MD simulation relates to the position of the termini of the protein chain; however, these ends are far from the active site. We then analyzed the substrate positions from each individual snapshot during the MD simulations and measured distances between the substrate and active site amino acids, as defined in Tables S2 and S3 (Supporting Information). Thus, during the full MD simulation, the carboxylic acid group of Asp_179_ is within 2 Å of the substrate ammonium terminus that holds the substrate in a tight orientation and position (Figure 3b). This is common in amino acid hydroxylases and seen before in, e.g., viomycin biosynthesis enzyme VioC.^22,23,56^ Another close amino acid residue from the substrate is the side chain of Arg_338_ that is held within 2–3 Å of the substrate and forms a strong salt bridge interaction with its carboxylate group. Although the Asn_232_ side chain points into the active site, its distance to the substrate is further than 3 Å, and therefore, it may not be an important residue for substrate positioning and catalysis.

As MD trajectories sometimes see major changes in the position of the substrate,^47,57−60^ we analyzed the substrate position with respect to the iron(IV)-oxo species during the MD simulation, and we display the C_3_–O, C_4_–O, and C_5_–O interactions in Figure 3c. After about 40 ns, the substrate–oxidant interaction stabilizes with the C_4_ atom closest to the iron(IV)-oxo species. In the MD run between 100 and 130 ns, however, the C_4_ and C_5_ atoms of the substrate are at similar distances. In the final stages of the MD simulation, i.e., after 130 ns, the substrate falls into a position that is closest to the iron(IV)-oxo species with the C_4_ atom of the substrate nearest to the oxidant. In those structures, the system is clearly set up for C_4_–H activation as the C_3_ and C_5_ atoms are much further away.

### DFT Cluster Calculations on the Reactants

**Figure 4:**
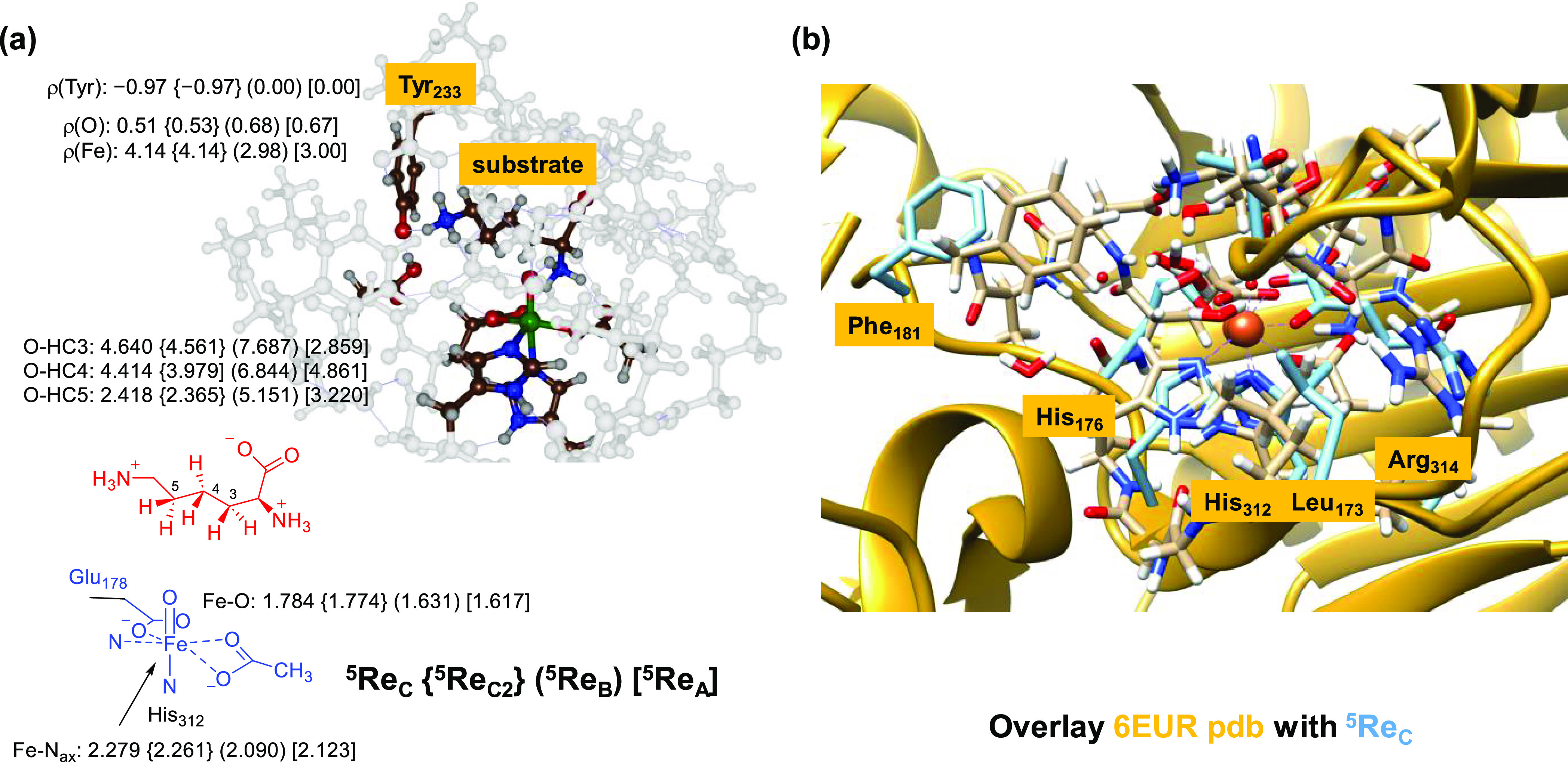
(a) UB3LYP-D3/BS1-optimized geometries of the iron(IV)-oxo species of KDO5 for models **A**, **B**, and **C** with bond lengths in Å. (b) Overlay of the ^5^**Re**_C_ structure (blue) with the crystal structure coordinates of the 6EUR PDB (gold).

Based on the last snapshot of the MD simulation, we created three QM cluster models that describe the metal and substrate and their first- and second-coordination spheres, namely, model **A** (297 atoms, total charge of −1), model **B** (312 atoms, total charge of 0), and model **C** (407 atoms, total charge of +2). DFT-optimized geometries of the iron(IV)-oxo species of KDO for models **A**, **B**, and **C** are shown in Figure 4. The quintet spin state is the ground state for all models, whereby for models **A** and **B**, the system has four unpaired electrons in metal-based molecular orbitals and configuration π*_xy_^1^ π*_xz_^1^ π*_yz_^1^ σ*_x2–*y*2_^1^. These orbitals represent interactions of the atomic 3d orbitals on the iron with first-coordination sphere atoms. In particular, the antibonding interactions of the atomic 3d_*xy*_, 3d_*xz*_, and 3d_*yz*_ orbitals with 2p orbitals on the oxo group yield the molecular π*_xy_, π*_xz_, and π*_yz_ orbitals. In addition, there is a singly occupied orbital (σ*_x2–y2_) for the σ-type antibonding interaction in the equatorial plane with the side chains of His_176_, Glu_178_, and succinate. The σ*_z2_ orbital is virtual and located along the N_ax_–Fe–O axis with N_ax_ being the axial nitrogen atom from His_312_. The orbital occupation and spin ground state observed for KDO5 models **A** and **B** match experimental work on analogous nonheme iron dioxygenases^27−30^ but also reproduce previous computational studies on other nonheme iron systems that gave an iron(IV)-oxo species with the quintet spin ground state.^61−83^ The alternative singlet and triplet spin states were also calculated but found to be higher in energy for model **C** by 22.3 and 5.3 kcal mol^–1^, respectively. These large spin state energy gaps imply that KDO will react on a dominant quintet spin state surface through single-state reactivity.

Interestingly, in model **C**, electron transfer has taken place from an active site Tyr residue (Tyr_233_) to the iron to create an iron(III)-oxo species and tyrosyl radical. Thus, Tyr_233_ has donated a proton to the Asp_179_ carboxylic acid group, and at the same time, an electron is transferred from the Tyr side chain to iron. As a consequence, the metal has five unpaired electrons, and there is considerable spin density found on Tyr_233_, where a down-spin electron is located with ρ = −0.97, while the Tyr residue shows no unpaired spin density in the iron(IV)-oxo complexes in models **A** and **B**. As such, in model **C**, the metal is in the oxidation state iron(III) with orbital occupation π*_xy_^1^ π*_xz_^1^ π*_yz_^1^ σ*_x2–y2_^1^ σ*_z2_^1^. The reactant complex ^5^**Re**_C_ therefore can be described as [Fe^III^(O)---Tyr^•^]. We tried to swap molecular orbitals for ^5^**Re**_C_ and create an iron(IV)-oxo with a neutral Tyr residue; however, during geometry optimization, the proton moved back to the carboxylate of Asp_179_ and the electron transferred to iron. The electronic configuration was also obtained with alternative computational approaches and protocols. Furthermore, expanding model **C** with the complete succinate and the Arg_334_ side chain (model **C2**) did not change the structure dramatically and resulted in a similar electronic configuration with a radical on Tyr_233_, a proton on Asp_179_, and an iron(III)-oxo group; see Figure 4. Therefore, in our large KDO5 model, the iron(IV)-oxo species is a fleeting intermediate that rapidly converged to an iron(III)-oxo species due to deprotonation of Tyr_233_ by the Asp_179_ residue and electron transfer. Interestingly, the Tyr-Asp couple does not seem conserved within the class of lysine dioxygenases;^84^ hence, these enzymes may operate through a different mechanism. Unfortunately, there are no reports of the iron-oxo species in KDO5 being trapped and characterized by electron paramagnetic resonance (EPR) and/or Mössbauer spectroscopy. However, a ground state for [Fe^III^(O)---Tyr^•^] should give it a distinct EPR and Mössbauer spectrum that is different from an [Fe^IV^(O)---Tyr] configuration.

In the heme enzyme cytochrome *c* peroxidase (C*c*P), similar valence tautomerism occurs and the active species is an iron(IV)-oxo(heme) with a nearby Trp radical, i.e., [Fe^IV^(O)(heme)---Trp^•+^].^85^ DFT calculations showed this state to be close in energy to an electronic state with an iron(IV)-oxo heme cation radical and closed-shell Trp group, i.e., [Fe^IV^(O)(heme^•+^)---Trp], whereby external perturbations, e.g., a cation binding site, near the heme determined its ground state.^86,87^ In addition, work on biomimetic model complexes has shown that the second-coordination sphere can change the electronic configuration of a metal complex due to long-range electrostatic interactions through valence tautomerism.^88−93^ As such, valence tautomerism should also be possible in enzymatic systems despite the fact that there is no experimental evidence in the literature of iron(III)-oxo species. Several biomimetic iron(III)-oxo complexes have been characterized in recent years and show differences in reactivity to iron(IV)-oxo complexes.^94−98^

The four optimized iron(IV)-oxo species have a very similar first-coordination sphere of the metal with its direct ligands particularly in the equatorial plane. The axial nitrogen atom of His_312_ (N_ax_) is at a distance of 2.279 Å from the iron atom in model **C**, while it is at 2.090 and 2.123 Å for models **B** and **A**, respectively. This is not surprising as ^5^**Re**_C_ has the σ*_z2_ orbital occupied with one electron that causes antibonding interactions along the O–Fe–N_ax_ axis. As a result, also the Fe–O interaction is elongated to 1.784 Å for model **C**, while it is 1.631 Å for model **B** and 1.617 Å for model **A**. Therefore, the different models give a first-coordination sphere geometry of the active site with bond lengths that match the orbital occupation and configuration.

The lysine substrate is bound in the substrate binding pocket and held by a salt bridge of its carboxylate group with the side chain of Arg_145_, and it forms hydrogen bonding interactions with the hydroxyl groups of Thr_165_ and Ser_167_. The terminal ammonium group of the substrate forms hydrogen bonding interactions with the carboxylic acid group of Asp_230_ and the phenol group of Tyr_233_ and two water molecules. A further hydrogen bonding network in the substrate and oxidant bonding pocket includes the side chains of Asp_179_, Asn_232_, and Arg_338_. As such, the substrate is tightly bound in the substrate binding pocket, which creates a highly rigid structure. These DFT calculations therefore support the results from the MD simulations that the substrate is tightly bound and highly rigid within the substrate binding pocket.

An overlay of the ^5^**Re**_C_-optimized geometry with the X-ray crystal structure coordinates of the 6EUR PDB file is shown on the right-hand side of Figure 4, while the overlay with the last snapshot from the MD simulation is shown in Figure S4 (Supporting Information). The match between the two structures is very good, and most protein backbone groups are in the same position in both systems. Several residues on the edge of the cluster model are highlighted in Figure 4 and are close to their starting position obtained from the crystal structure coordinates. The overlay of the two structures therefore confirms that the protein structure is tight and rigid and shows little flexibility as a function of time. Moreover, our models reflect the enzyme structure well and are expected to give an accurate reflection of the real system.

### Hydrogen Atom Abstraction from Lys

Next, we calculated the hydrogen atom abstraction pathways of substrate Lys on the C_3_–H, C_4_–H, and C_5_–H positions as described in Scheme S1 (Supporting Information). In previous work, we explored *pro*-*R* and *pro*-*S* hydrogen atom abstraction of taurine in taurine/αKG dioxygenase.^45^ As those calculations gave close energy barriers for *pro*-*R* and *pro*-*S* pathways, we considered one stereoisomer only here. In particular, we investigated hydrogen atom abstraction via transition state **TS1** to form radical intermediate **IM1**. Thereafter, an OH rebound barrier via the transition state **TS2** leads to alcohol products (**Pr**). The labeling system gives the spin multiplicity in superscript before the label and the position of the hydrogen on the substrate and the cluster model in subscript after the label. In all cases, hydrogen atom abstraction is rate-determining and the OH rebound has a much lower barrier. Table 1 summarizes the calculated barrier heights for hydrogen atom abstraction from the C_3_–H, C_4_–H, and C_5_–H positions of the Lys substrate by the iron-oxo species of KDO for models **A**, **B**, and **C**.

**Table 1:** Hydrogen Atom Abstraction Transition States Calculated for C_3_–H, C_4_–H, and C_5_–H Abstraction from Lysine by KDO5 Iron(IV)-oxo Species^a^

	model **A**		model **B**		model **C**	
structure	Δ*E* + ZPE	Δ*G*	Δ*E* + ZPE	Δ*G*	Δ*E* + ZPE	Δ*G*
^5^**TS1**_HA,C3_	17.3	14.6	24.6	27.8	26.7	28.6
^5^**TS1**_HA,C4_	17.0	18.5	24.2	28.2	24.7	24.8
^5^**TS1**_HA,C5_	14.4	13.1	13.5	18.6	26.7	27.0

a Energies are calculated at the BS2 level of theory, while ZPE, thermal, and entropic corrections are obtained at the BS1 level of theory.

In models **A** and **B**, the lowest free energy of activation for the hydrogen atom abstraction barrier is via ^5^**TS1**_HA,C5_ with magnitudes of Δ*G*^‡^ = 13.1 kcal mol^–1^ (model **A**) and Δ*G*^‡^ = 18.6 kcal mol^–1^ (model **B**). In model **B**, the other hydrogen atom abstraction barriers have a free energy that is >9 kcal mol^–1^ higher. Consequently, model **B** predicts selective hydrogen atom abstraction from the C_5_–H position, and little or no alternative products are predicted for this model. This is in contrast to experimental data on this KDO structure that measured dominant C_4_–H hydroxylation products.^6,32^

In model **A**, the margins between the hydrogen atom abstraction barriers are much smaller and the C_3_–H hydrogen atom abstraction is only Δ*G* = 1.5 kcal mol^–1^ above the one for C_5_–H, and therefore, some C_3_–H may be expected, although the C_4_–H barrier is still Δ*G* = 5.4 kcal mol^–1^ higher in free energy than the C_5_–H hydrogen atom abstraction barrier. Nevertheless, both models **A** and **B** predict little or no C_4_-hydroxylation products, which disagrees with experiment for this particular isozyme.^6−9^ These models give the dominant C_5_-hydroxylation of Lys rather than the expected C_4_-hydroxylation. Models **A** and **B** differ from each other through the additional Arg residue in model **B**. The large rise in barrier heights in model **B** therefore is the result of the inclusion of this residue, which fixes the substrate in a tighter orientation that makes hydrogen atom abstraction from some positions more difficult. In particular, Arg_145_ strongly destabilizes the pathway for C_3_–H hydrogen atom abstraction. As both models **A** and **B** predict the wrong products, these models do not accurately mimic KDO activity. Therefore, we explored further expanded model **C**, which is an even larger cluster model with an additional Arg residue in the second coordination sphere and several chains surrounding the substrate binding pocket.

In model **C**, we find the lowest energy transition state via ^5^**TS1**_HA,C4_ with a free energy of activation of Δ*G*^‡^ = 24.8 kcal mol^–1^, while the barriers for C_3_–H and C_5_–H activation are 3.8 and 2.2 kcal mol^–1^ higher lying, respectively. Consequently, model **C** predicts dominant C_4_–H hydrogen atom abstraction, in agreement with experimental product distributions. Therefore, we will focus the rest of the paper on the model **C** results. Model **C** includes one additional Arg residue, namely, Arg_314_, and its electrostatic perturbations appear to destabilize the C_5_–H hydrogen atom abstraction pathway. As such, the two Arg residues (Arg_145_ and Arg_314_) are positioned so that they disfavor the hydrogen atom abstraction pathways from the C_3_–H and C_5_–H positions and enable efficient hydrogen atom abstraction from the C_4_–H position. These perturbations, however, do raise the barrier heights for all processes significantly but will succeed in a regioselective reaction pathway.

**Figure 5:**
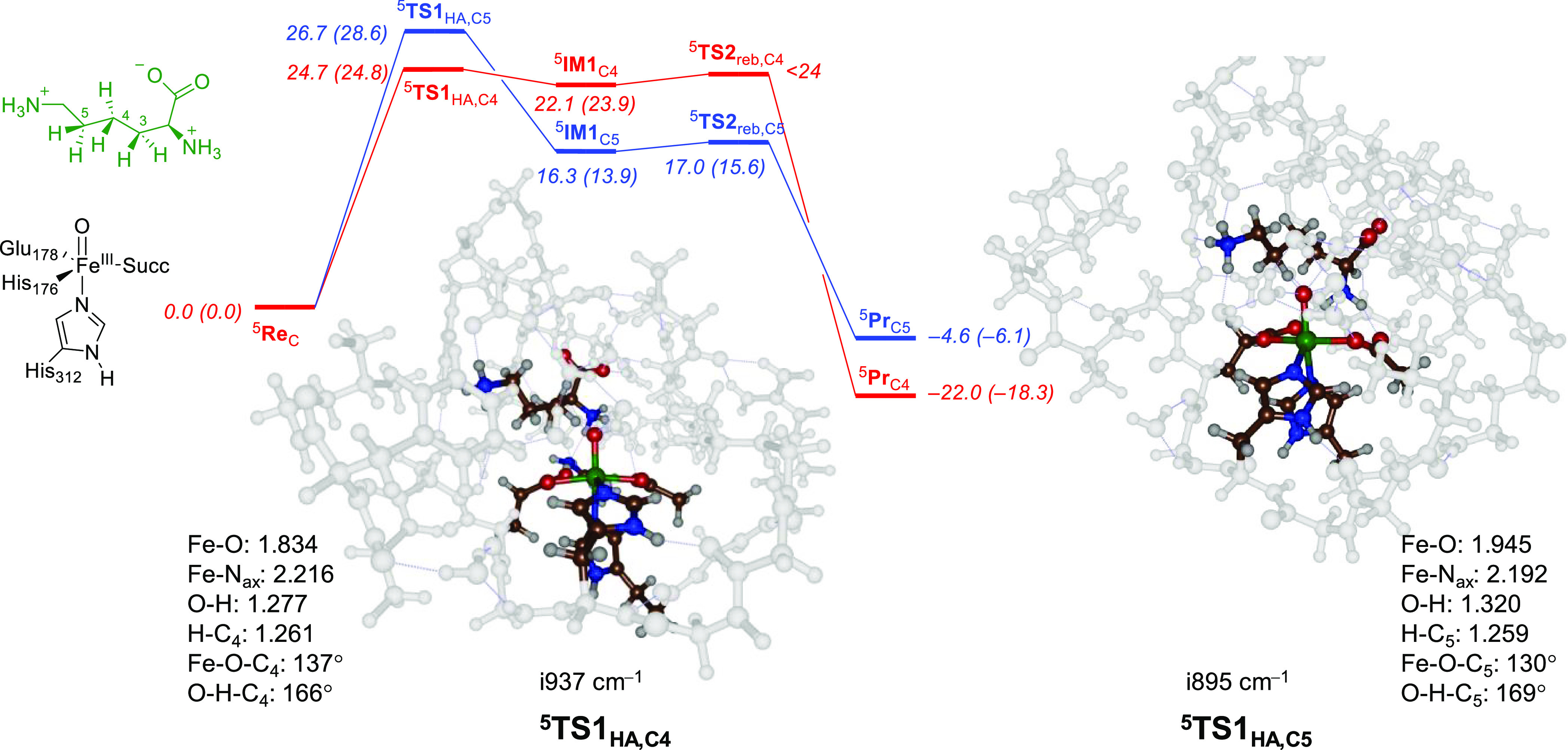
UB3LYP/BS1//UB3LYP/BS2-calculated free energy profile for Lys hydroxylation at the C_4_–H (red) and C_5_–H (blue) positions in KDO5. Energies are in kcal mol^–1^ with Δ*E* + ZPE (outside parentheses) and Δ*G* (in parentheses). Optimized transition state geometries give bond lengths in Å and angles in degrees.

The full energy landscape for Lys hydroxylation at the C_4_–H and C_5_–H positions as calculated for model **C** is given in Figure 5. After the hydrogen atom abstraction, the structures relax to a radical intermediate; however, there is a small barrier for OH rebound, leading to alcohol products with large exothermicity. Consequently, we expect the radical intermediate to have a very short lifetime. The reactant of model **C** has an electronic configuration of π*_xy_^1^ π*_xz_^1^ π*_yz_^1^ σ*_x__2__–y__2_^1^ σ*_z__2_^1^, i.e., [Fe^III^(O)---Tyr^•^]. During the hydrogen atom abstraction step of the Lys hydroxylation mechanism, the Tyr group is reprotonated again and accepts an electron from substrate Lys so that we form an iron(III)-hydroxo group with a Lys radical. In all radical intermediates (^5^**IM1**_C3_, ^5^**IM1**_C4_, and ^5^**IM1**_C5_) for model **C**, there is a down-spin electron on the substrate and five up-spin unpaired electrons in metal-type orbitals. After the radical intermediates, facile OH rebound (with a negligible barrier) leads to alcohol product complexes with large exergonicity. Therefore, the radical intermediates will be short-lived and quickly convert into alcohol product complexes in an irreversible step.

Optimized geometries of ^5^**TS1**_HA,C4_ and ^5^**TS1**_HA,C5_ for model **C** are shown in Figure 5. In both structures, the Fe–O bond is long, probably due to occupation of the σ*_z2_ orbital with one electron. The ^5^**TS1**_HA,C4_ structure has the transferring hydrogen atom almost midway between the donor and the acceptor atom, i.e., the O–H distance is 1.277 Å and the C_4_–H distance is 1.261 Å. This is similar in ^5^**TS1**_HA,C5_, where the transferring hydrogen atom is at an O–H distance of 1.320 Å, while the C_5_–H distance is at 1.259 Å. Therefore, the C_5_–H hydrogen atom abstraction structure is reactant-like, whereas the C_4_–H structure is neither reactant- nor product-like but intermediate. Overall, the structural differences between the two transition states are small and similar angles and first-coordination sphere metal–ligand interactions are found. Moreover, optimized hydrogen atom abstraction transition state structures match previous calculations on nonheme iron enzymes and biomimetic models well.^58−83,99−110^ In both transition state structures, the angle between the substrate and the oxidant is similar. Thus, the Fe–O–C angle of the carbon atom that loses a hydrogen atom is 137° for ^5^**TS1**_HA,C4_, while it is 130° for ^5^**TS1**_HA,C5_. Also, the O–H–C angles are similar at 166° and 169°, respectively. Therefore, the mechanistic and energetic differences observed here are not the result of structural differences in the transition states.

## Discussion

In this work, the regio- and chemoselective hydroxylation of a free Lys residue by KDO enzymes is explored. Three models were investigated, but only the largest model (model **C**) shows a preference for C_4_-hydroxylation products, while models **A** and **B** predict C_5_-hydroxylation. To understand the differences between the cluster models and, particularly, the hydrogen atom abstraction energetics, we analyzed the structure and electronic configuration of all species in detail.

**Figure 6:**
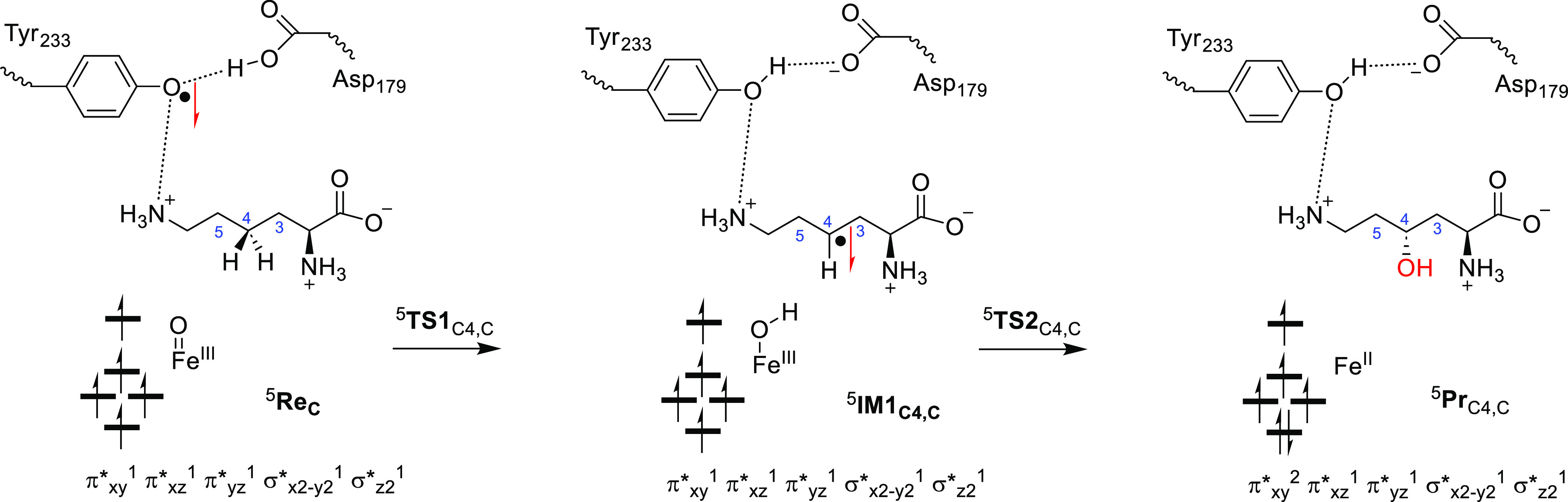
Electronic configuration of the intermediates along the hydroxylation mechanism for model **C**.

Figure 6 shows the electron transfer pathways calculated for the hydrogen atom abstraction mechanisms for model **C** of KDO. As reported above, instead of a reactant that is iron(IV)-oxo with a quintet spin ground state as is commonly observed for αKG-dependent nonheme iron dioxygenases,^27,45,61−77,111,112^ we find an iron(III)-oxo reactant species. This is the result of proton-coupled electron transfer from Tyr_233_ that has released a proton to the carboxylate group of Asp_179_ and an electron donated into the iron set of orbitals. Thus, the carboxylate group of Asp_179_ is within the hydrogen bonding distance (1.596 Å in ^5^**Re**_C_) from the phenol group of Tyr_233_, while on the other side of the Tyr residue, the terminal ammonia group of Lys is at a short distance in the other direction (1.95 Å). The terminal ammonia group of substrate Lys via a bridging water molecule connects to the oxo group of the iron(III)-oxo species through a hydrogen bonding network. We therefore anticipate that the mutation of Asp_179_ by, e.g., Asn_179_ will prevent the proton-coupled electron transfer from happening and presumably affects product distributions dramatically.

The reaction mechanism then continues with another proton-coupled electron transfer, whereby the iron(III)-oxo species abstracts the proton from the C_4_-position. At the same time, an electron is transferred from substrate Lys to the tyrosyl radical of Tyr_233_, which results in proton transfer from Asp_179_ to Tyr_233_. In the radical intermediate, therefore, the metal stays in the iron(III) oxidation state, but the radical disappears from the Tyr_233_ residue and accumulates on the substrate. Indeed, the group spin densities on iron are 4.1 in ^5^**Re**_C_ and 4.2 in ^5^**IM1**_C_. On the other hand, a spin of −0.97 is found on the protein (mostly on Tyr_233_) in ^5^**Re**_C_, while no spin on the substrate is observed. In the radical intermediate, the spin on the substrate is −0.95, while the spin on Tyr has disappeared. Recent combined experimental and computational studies on a biomimetic iron(III)-oxo species, as compared to its iron(IV)-oxo analog, showed that both oxidation states react with toluene with similar hydrogen atom abstraction barriers.^75^ However, iron(III)-oxo by itself can only react as a one-electron oxidant and is not able to perform OH rebound reactions. In the KDO system discussed here, iron(III)-oxo has a nearby protein radical (on Tyr_233_) that can be used as the second oxidation equivalent during the reaction mechanism. Indeed, the OH rebound step transfers the final electron to the metal to form the iron(II)-alcohol complex to complete the catalytic cycle. It would be interesting to investigate what effect site-specific mutations of the Asp_179_ and Tyr_233_ groups will have on the general catalysis by KDO5 enzymes. We predict that mutation of the residues Asp_179_ and Phe_233_ will significantly affect the product distributions and regioselectivity of lysine dioxygenase as it will influence the dipole moment and local electric field effect inside the active site.

**Figure 7:**
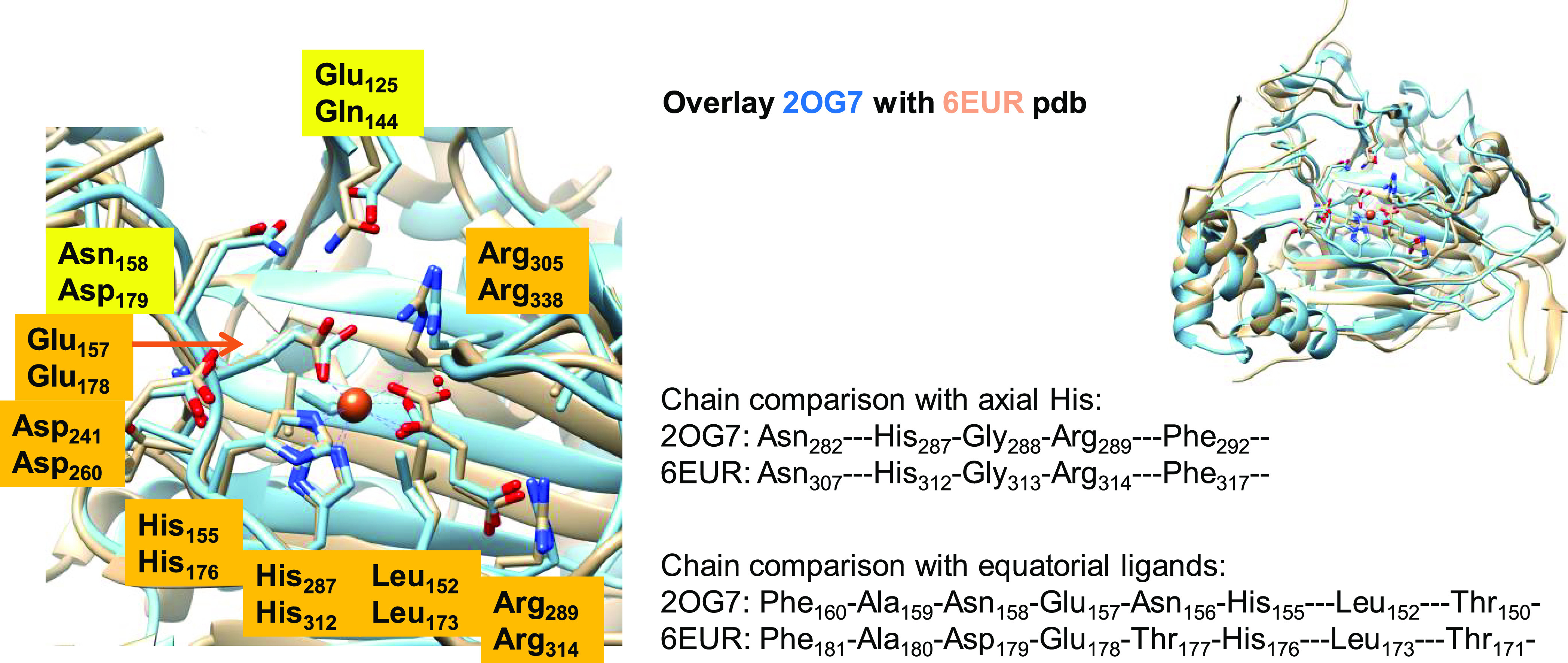
Structure comparison of KDO5 (6EUR PDB file) with asparagine hydroxylase (2OG7 PDB).

The active site structure and overall protein fold of KDO5 are not dramatically different from other nonheme iron dioxygenases and hence do not explain the PCET observation. In particular, a search for analogous enzyme structures shows that the closest enzyme structures of KDO5 are the structures of asparagine hydroxylase (AH, 25.9% homology) and taurine/α-ketoglutarate dioxygenase (TauD, 87.1% homology). Both AH and TauD are α-ketoglutarate-dependent nonheme iron dioxygenases that bind an iron(II) to a 2-His-1-carboxylate orientation of the protein. As such, all three enzymes utilize molecular oxygen and convert α-ketoglutarate to succinate concomitant to the C–H hydroxylation of a substrate. Figure 7 shows the comparison of the structures of KDO5 and asparagine dioxygenase as taken from the 6EUR and 2OG7 PDB files.^22,23,113^ The overlay of the two PDB files is shown in the top right corner of Figure 7 and shows the same structural features and protein fold for the two enzymes with loops, helices, and chains in similar position and orientation. The only difference appears to be an additional loop on the edge of the protein. When we zoom in into the active site region, the strong similarities between the two proteins become even more apparent (left-hand side of Figure 7). Two active site chains are highlighted in Figure 7, namely, part of the chain that includes the equatorial ligands and the part surrounding the axial ligand of the iron(II) ion. Thus, both proteins have the same 2-His/1-Glu coordination environment that holds the metal in position. They also both bind αKG in roughly the same position with the α-keto and carboxylate groups in the same plane as the equatorial ligands. The two protein structures have a protein loop surrounding the αKG cosubstrate that position it in the active site through interactions with the side chains of Leu and Arg residues: Leu_152_ and Arg_289_ in asparagine hydroxylase and Leu_173_ and Arg_314_ in KDO5. Therefore, the αKG binding loop is conserved among αKG-dependent nonheme iron dioxygenases as reported before.^114^ The only major difference between the two protein structures is the position of a carboxylate-containing residue (Asp and Glu) in the substrate binding pocket. Thus, in the position of Glu_125_ in the substrate binding pocket of asparagine dioxygenase, there is a Gln_144_ residue in KDO5, while in KDO5, there is a carboxylate in position 158 (Asp_158_), whereas asparagine dioxygenase has an Asn amino acid in that position. The difference in the position of an anionic amino acid in the substrate binding pocket will lead to differences in the charge and local electric field orientation in the protein. Previously, a similar difference between the VioC and NapI enzymes was identified as a cause to the dipole moment change and to explain the differences in reactivity of the two enzymes, whereby VioC acts as a C_3_-hydroxylase of free Arg substrate, while NapI reacts with l-Arg through desaturation of the C_4_–C_5_ bond.^115^

**Figure 8:**
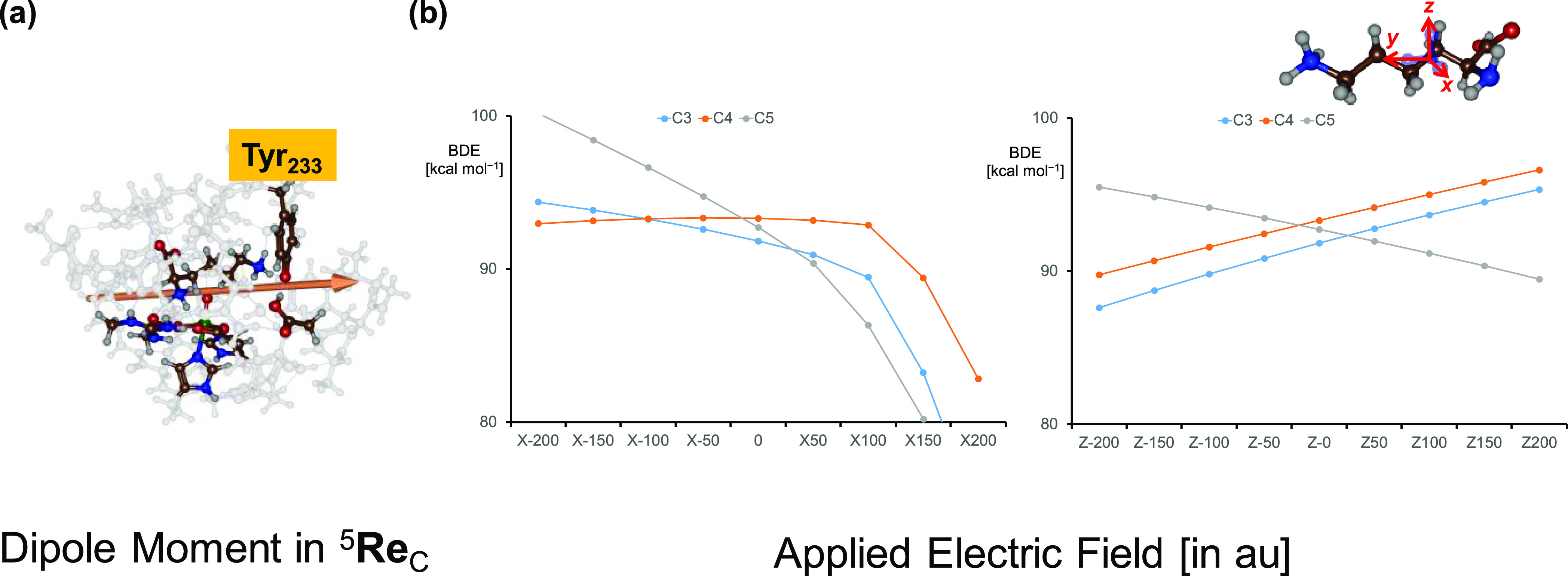
(a) Electric dipole moment in ^5^**Re**_C_. (b) Electric field effects along the *x*- or *z*-direction on the C–H BDEs of an isolated lysine substrate molecule with Δ*G* values (in kcal mol^–1^) as a function of the applied electric field.

Next, we explored the electrostatics of the second coordination sphere of the model and the influence of the electric field effects. The overall dipole moment of structure ^5^**Re**_C_ is shown in Figure 8, while those in models **A** and **B** are shown in Figure S8 (Supporting Information). The dipole moment of model **C** in the ^5^**Re**_C_ state points perpendicular to the chain of atoms of the substrate along the C_4_–H bond. In recent work on the viomycin biosynthesis enzyme VioC, we showed that substrate arginine has its C_3_–H bond aligned with the dipole moment in the active site.^116^ This results in the weakening of the C_3_–H bond and strengthening of the C_4_–H bond of the substrate. In VioC, the dipole moment therefore guides the reaction selectivity and triggers a lesser thermodynamic favorable pathway. By contrast, in the naphthyridinomycin biosynthesis enzyme NapI, the dipole is along the substrate backbone and appear not to influence the C–H bond strengths in the substrate.^117^ As a consequence, in NapI, the weakest C–H bond in the gas phase is activated by the protein, whereas in VioC, one of the stronger bonds is activated instead. The dipole moment in model **C** is also perpendicular to the aromatic ring of Tyr_233_ and hence is likely to stabilize a radical center on this group and trigger an electron transfer from Tyr_233_ to iron(IV)-oxo and a simultaneous proton transfer from Tyr_233_ to Asp_179_. In model **A**, on the other hand, the dipole vector is perpendicular to the one in ^5^**Re**_C_ and therefore keeps the nonheme center in an iron(IV)-oxo state that reacts differently with the substrate. In particular, in ^5^**Re**_A_, the C_5_–H bond is weakened, and abstraction of this hydrogen atom is preferential.

To understand how the substrate C–H bond strengths are influenced by the environment and external perturbations, we calculated the C–H bond strengths of an isolated lysine molecule versus those of a hydrogen atom and lysine with one hydrogen atom removed. In the gas phase, the C_3_–H, C_4_–H, and C_5_–H bond dissociation free energies (BDEs) are Δ*G* = 91.8, 93.3, and 92.7 kcal mol^–1^, respectively. As such, in the gas-phase for an isolated Lys amino acid, the weakest C–H bond is the C_3_–H bond, and hence, hydrogen atom abstraction from the C_3_–H position should give the most exergonic reaction step. Based on the Bell–Evans–Polanyi principle, the most abundant product should therefore originate from C_3_–H hydrogen atom abstraction. Often, enzymes overcome the Bell–Evans–Polanyi rules by substrate and oxidant positioning and external perturbations in the substrate binding pocket and lead to products that are not expected from the thermochemistry of the reactions. This is sometimes termed negative catalysis.^118^ To overcome the large exergonicity of hydrogen atom abstraction from the C_3_–H position, external perturbations and substrate binding orientations may block this reaction channel.

To investigate the effect of external perturbations on lysine C–H bond strengths, we recalculated the C_3_–H, C_4_–H, and C_5_–H BDEs in the presence of an applied electric field along either the molecular *x*-, *y*-, or *z*-axis of the substrate. Previous calculations with electric field effects applied showed these perturbations to affect electronic properties and charge distributions and even lead to selectivity changes.^88,119−127^ The definitions of the *x*-, *y*-, and *z*-axes in the substrate are shown in Figure 8 with the *y*-axis along the carbon chain and the *x*- and *z*-axes along the C–H bonds. Figure 8b shows the BDE patterns as a function of the applied electric field along the *x*- and *z*-axes, which are both perpendicular to the carbon chain of the substrate. In the gas phase, i.e., with zero electric field, the C_5_–H position has the weakest bond followed by the C_3_–H and C_4_–H positions. A field along the positive *x*- or *z*-direction keeps these orderings, although the energy separations change. With a negative direction of the electric field (based on the definition in Gaussian) along the *x*-axis, however, C_4_–H becomes the weakest bond and C_5_–H the strongest bond. Under a local electric field effect in the protein, therefore, C_4_–H hydrogen atom abstraction can be favored over the C_3_–H and C_5_–H channels. On the other hand, a field along the *z*-axis in the negative electric field direction makes the C_3_–H bond of the substrate the weakest and the C_5_–H bond the strongest. These electric field calculations show that the charge distributions in the substrate binding pocket can be crucial in determining the weakest C–H bond of the substrate within the substrate binding pocket. The charge distributions ultimately determine the regio- and stereoselectivity of the reaction process.

To find out whether the BDEs of the substrate C–H bonds are indeed affected by the cluster models, we took the ^5^**Re**_A_ (model **A**), ^5^**Re**_B_ (model **B**), and ^5^**Re**_C_ (model **C**) structures and ran a single point energy calculation in the sextet spin state with one hydrogen atom removed from the substrate from position C_3_–H, C_4_–H, or C_5_–H. Using ^5^**Re**_A_ as a starting point, all substrate BDE values are calculated to be within 0.3 kcal mol^–1^ of each other. By contrast, for ^5^**Re**_C_, the weakest substrate bond is the C_4_–H interaction with a BDE of Δ*E* = 109.3 kcal mol^–1^. On the other hand, the C_3_–H and C_5_–H BDEs are Δ*E* = 112.1 and 112.9 kcal mol^–1^. Finally, for model **B**, the weakest C–H bond is the C_3_–H bond at 109.2 kcal mol^–1^, while the C_4_–H bond has a BDE of 110.1 kcal mol^–1^. Therefore, the dipole moment in model **C** and the local electric field strengths polarize the substrate and lead to preferential C_4_–H hydroxylation of substrate lysine, while that is not the case with the other cluster models.

We further applied electric field calculations on a minimal cluster model of iron(IV)-oxo with its first coordination sphere only. These calculations give a strong field effect on the reduction potential of the iron(IV)-oxo species with a field located along the molecular *z*-axis. This is not surprising as iron(IV)-oxo reduction leads to filling of the σ*_z2_ orbital with one electron, which is an orbital along the molecular *z*-axis. In addition, electric field effect calculations were done on a minimal model containing *p*-cresol and acetate. These calculations show that proton transfer can be triggered by an electric field effect parallel to the hydrogen bond axis. Our calculations therefore indicate that subtle perturbations from protein residues can trigger a proton transfer, electron transfer, or a combination of the two to obtain valence tautomerism in the active oxidant.

## Conclusions

In this work, a computational study is presented on a 4-lysine dioxygenase. We set up three cluster models based on an MD simulation of the substrate-bound iron(IV)-oxo species. The MD shows tight substrate binding with strong interactions between the substrate and polar active site residues. For all three clusters, a full reaction mechanism for the hydroxylation of l-Lys on the C_3_-, C_4_-, and C_5_-positions was calculated. In general, the hydrogen atom abstraction transition state is rate-determining and the OH rebound step is much lower in energy. Only the largest cluster model predicts C_4_-hydroxylation, while the other two models predict C_5_-hydroxylation. As such, the polarity of the models and the second-coordination sphere of the substrate and oxidant influences reactivities and kinetics of the substrate activation processes. We then analyzed the structures and found that the dipole moment is different in the three cluster models and particularly points along the C_4_–H bond in model **C**, while it does not point along the C_4_–H bond in the other models. Further analysis of the structures shows that KDO reacts through negative catalysis where the electric dipole moment and charge distributions in the substrate binding pocket affect the various substrate C–H bond strengths and trigger the C_4_-hydroxylation mechanism. Our work shows that KDOs can be selectively engineered to give the preferred reaction pathways with substrate lysine or alternative substrates. Finally, in the largest cluster model, we observe unusual proton-coupled electron transfer where an active site Tyr residue is involved in the catalysis and reduces the iron from iron(IV) to iron(III) while donating its proton to a nearby Asp residue. This implies that in KDO5, substrate activation is performed by an iron(III)-oxo species rather than an iron(IV)-oxo species. Despite the lower valency of the metal center, the reaction still proceeds through substrate hydroxylation, which further shows that iron(III)-oxo species are capable of substrate oxidation events. This unusual proton-coupled electron transfer with assistance of an active site Tyr residue may be common in highly selective and energy-demanding (negative catalysis) reaction processes in nature, and we are searching for further applications of this interesting phenomenon.

## Supporting Information Available

Tables with absolute and relative energies, group spin densities and charges, and Cartesian coordinates of optimized geometries discussed in this work (PDF)

## Abbreviations

αKG: α-ketoglutarate
BDE: bond dissociation energy
KDO: lysine dioxygenase
PCET: proton-coupled electron transfer
PDB: Protein Data Bank
ZPE: zero-point energy
