# Supplementary material for: An Active Site Tyr Residue Guides the Regioselectivity of Lysine Hydroxylation by Nonheme Iron Lysine-4-hydroxylase Enzymes through Proton-Coupled Electron Transfer
Source: J Am Chem Soc. 2024 Apr 18;146(17):11726–39. doi: 10.1021/jacs.3c14574 (PMC11066847; doi:10.1021/jacs.3c14574)
Supplement: Supplementary file 1 — ja3c14574_si_001.pdf [file ja3c14574_si_001.pdf]

Supporting Information

An Active Site Tyr Residue Guides the Regioselectivity of Lysine Hydroxylation by the Nonheme Iron Lysine-4-Hydroxylase enzymes Through Proton-Coupled-Electron-Transfer.

Yuanxin Cao,[§,&] Sam Hay,[§,&] and Sam P. de Visser*[§,⊥]

[§] Manchester Institute of Biotechnology, The University of Manchester, 131 Princess Street, Manchester M1 7DN, United Kingdom

[&] Department of Chemistry, The University of Manchester, Oxford Road, Manchester M13 9PL, United Kingdom

[⊥] Department of Chemical Engineering, The University of Manchester, Oxford Road, Manchester M13 9PL, United Kingdom

Email: sam.devisser@manchester.ac.uk

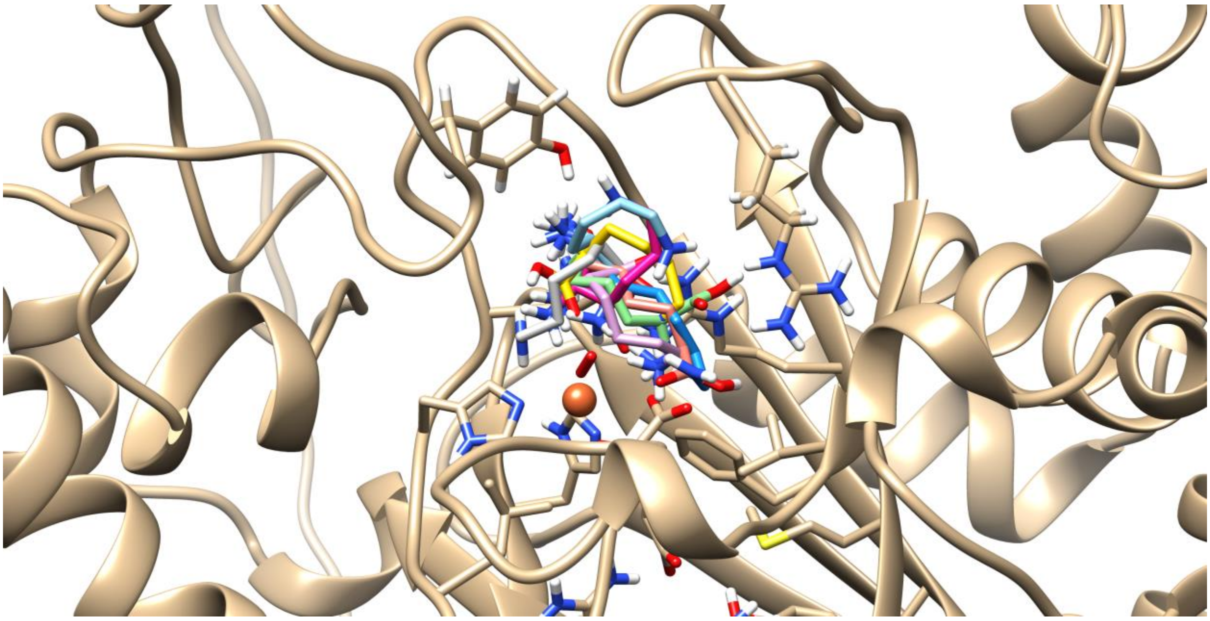

Figure S1: Overlay of the docked substrate positions in the active site of KDO5. The light-blue structure/position was selected as starting point for the MD simulation.

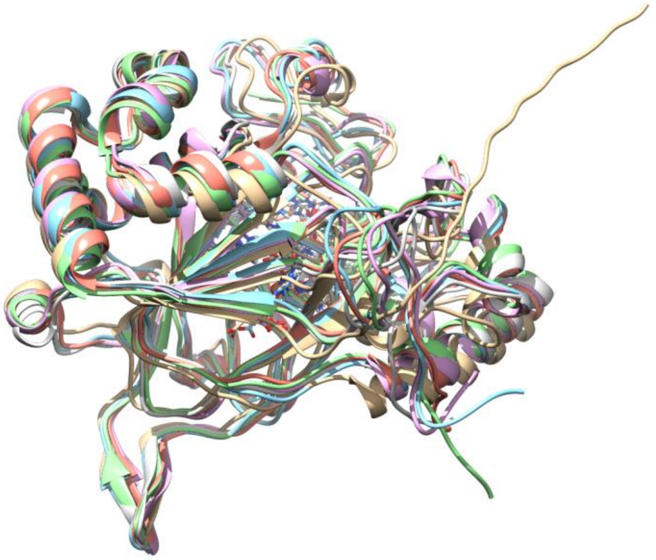

Figure S2: Overlay of the MD frames after 0 ns (starting point, yellow), 10 ns, 20 ns, 30 ns, 40 ns and 50 ns (other colors).

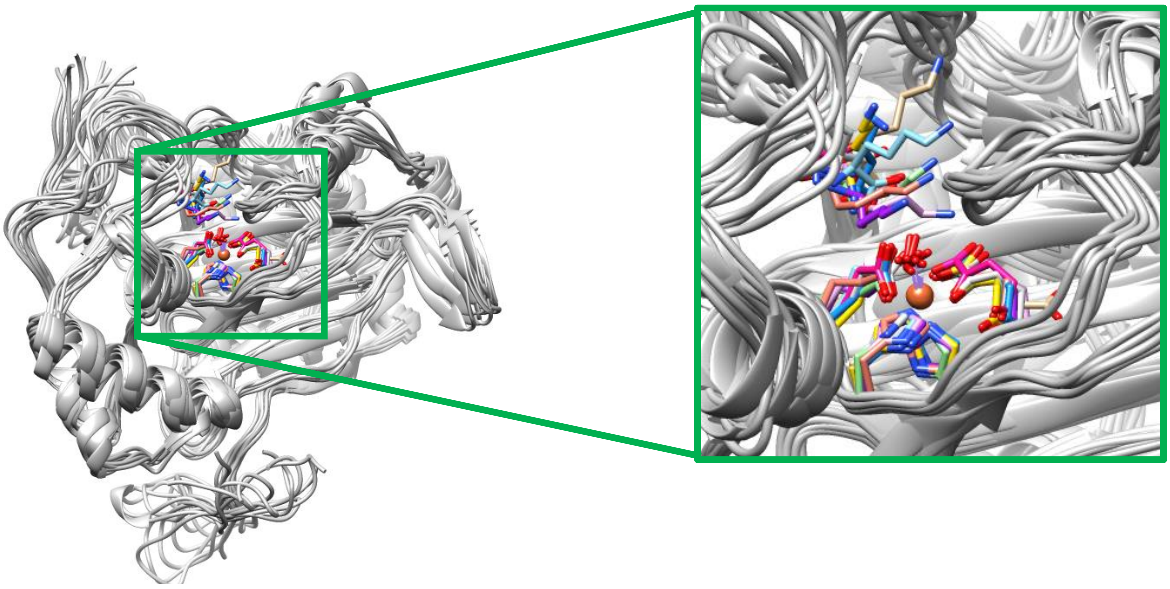

Figure S3: Overlay of the MD frames after 20, 40, 60, 80, 100, 120, 140, 160, 180, and 200 ns. As can be seen the protein chains are highly similar throughout the MD simulation and also the metal with its first coordination appears rigid during the full MD simulation. Even the αKG is in similar positions throughout the MD simulation.

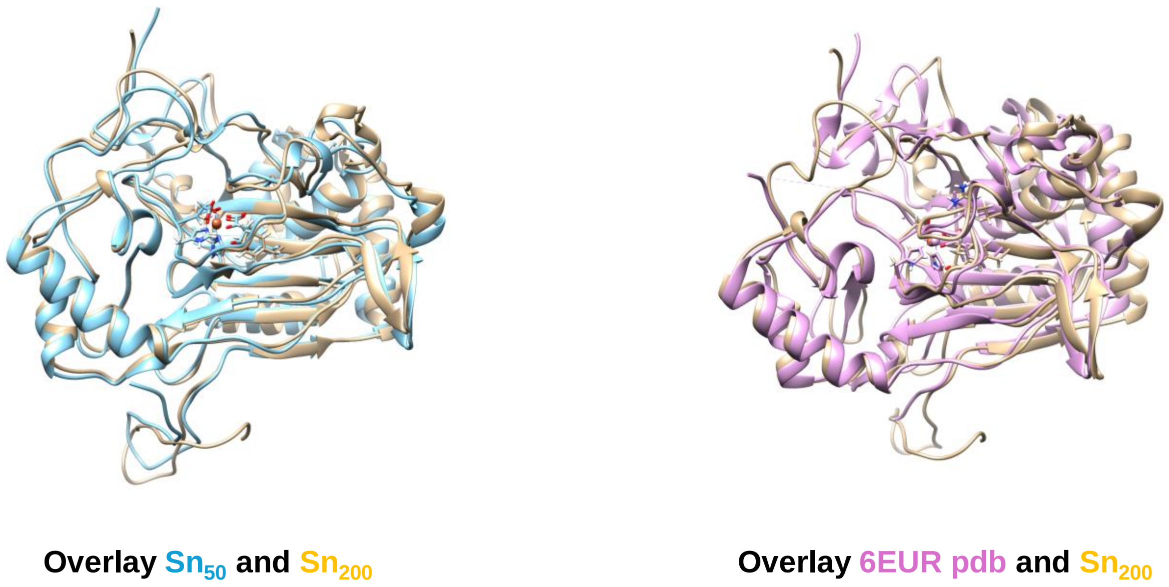

Figure S4: Overlay of the MD frames after 50 ns and 200 ns (left-hand-side) and an overlay of the MD frame after 200 ns with the original 6EUR pdb file. As can be seen, both MD frames have almost identical fold and metal first-coordination sphere. The overlay of the last frame from the MD with the crystal structure coordinates also shows similar fold.

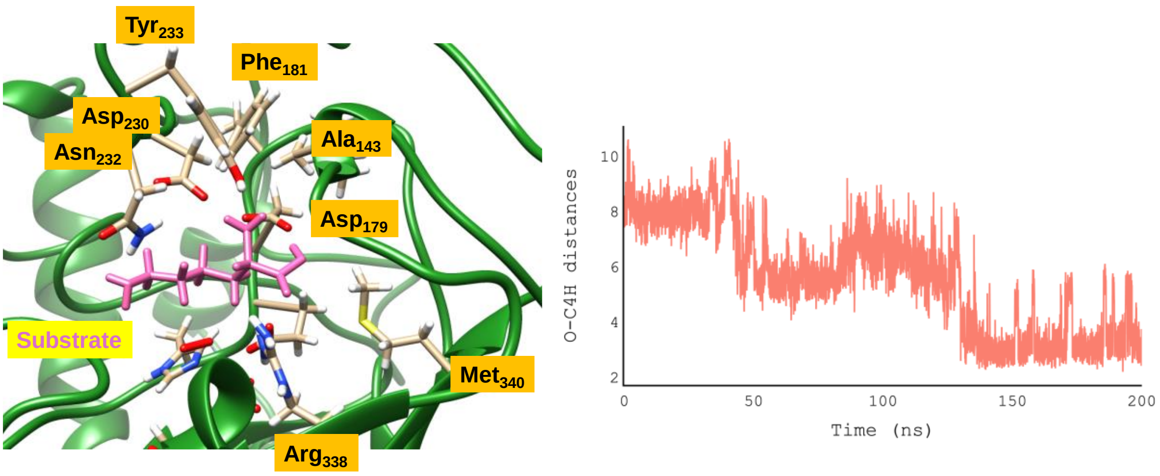

Figure S5: Left: Substrate positioning in the active site in the snapshot after 200 ns from the MD simulation. Right O−HC_4 distance measured along the 200 ns MD simulation.

Table S1: Docking results given by Autodock vina on L-Lys docking into the 6EUR pdb file (chain A).

Position	Score	RMSD Å	BFE kcal/mol
1	-4.816	0.000	-6.878
2	-4.798	3.191	-6.854
3	-4.751	3.565	-6.788
4	-4.746	1.881	-6.782
5	-4.715	2.599	-6.738
6	-4.712	2.694	-6.735
7	-4.578	2.469	-6.550
8	-4.556	3.407	-6.519
9	-4.527	4.241	-6.479

Table S2: Atoms selected in the measurement of residue-substrate distances in the MD structural analysis.

atom1		atom2	
ALA143	HB2	L-LYS	HC
ARG145	2HH1	L-LYS	H22
ASP179	OD2	L-LYS	H15
ASP230	OD1	L-LYS	HC4
ASN232	OD1	L-LYS	HC7
ARG338	2HH2	L-LYS	O1

Table S3: Atomic names and coordinates of L-Lysine used as a substrate in MD simulations.

C3	28.39	34.07	59.40
HC2	28.22	34.59	60.35
HC3	28.95	33.16	59.63
C1	29.13	34.93	58.34
HC	28.45	35.64	57.88
C2	30.15	35.94	59.04
O1	29.83	36.53	60.02
O2	31.30	36.28	58.42
H15	31.27	35.93	57.52
N1	29.75	34.08	57.22
2HN1	30.15	33.28	57.73
3HN1	30.42	34.53	56.58
1HN1	28.96	33.73	56.66
C4	26.95	33.62	58.89
HC4	26.45	32.97	59.62
HC5	27.05	33.10	57.93
C5	25.88	34.74	58.62
HC6	24.96	34.19	58.38
HC7	26.06	35.37	57.74
C6	25.73	35.68	59.84
HC8	24.83	36.28	59.65
HC9	25.59	35.22	60.82
N2	26.76	36.71	59.86
H21	26.44	37.39	60.55
H22	27.57	36.27	60.30

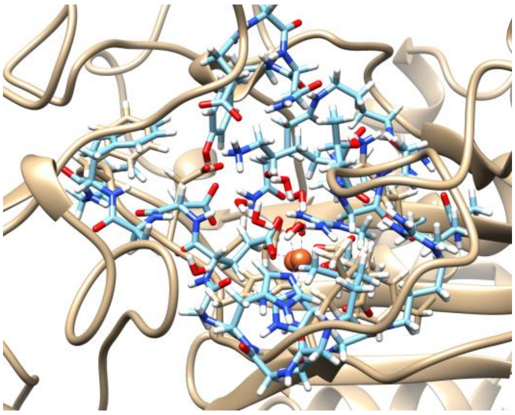

Overlay $^{5}Re_{C}$ and Sn_{200}

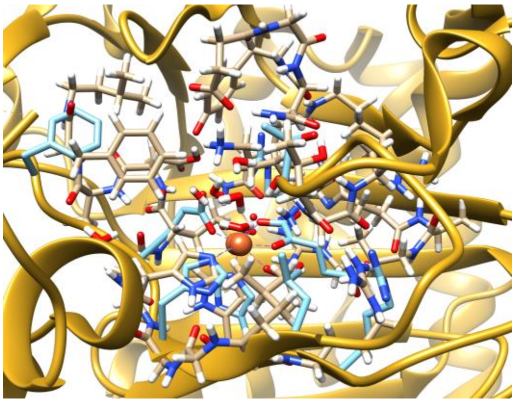

Overlay $^{5}Re_{C}$ and 6EUR pdb

Figure S6: Overlay of the optimized reactant spin structure ($^{5}Re_{C}$) with the last snapshot of the MD simulation (left-hand-side) and with the crystal structure coordinates of the 6EUR pdb (right-hand-side).

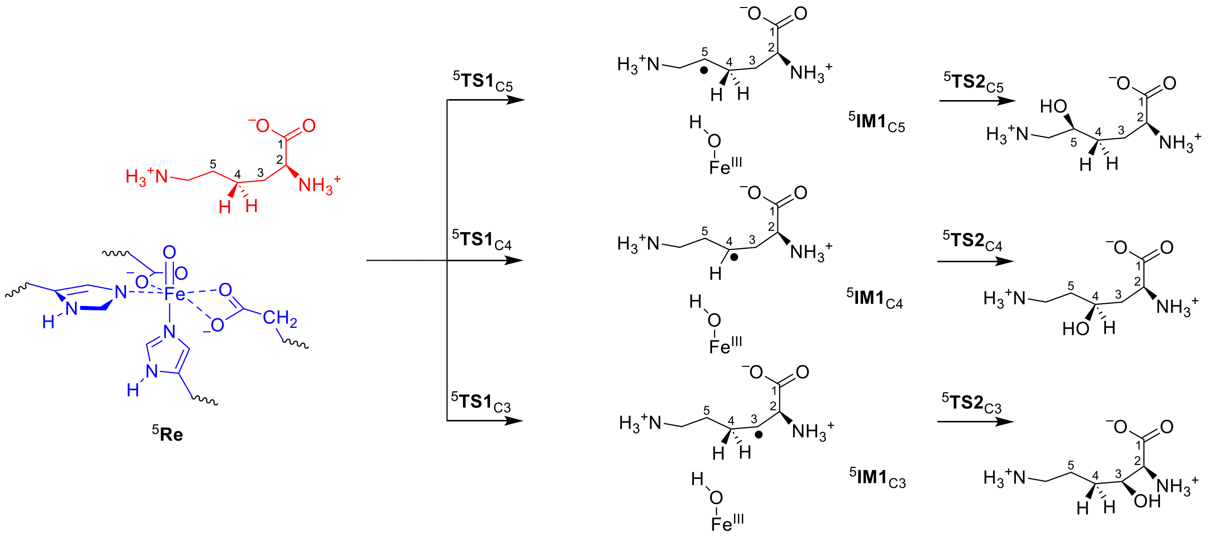

Scheme S1: Reaction scheme calculated with DFT methods in this work with definition of the nomenclature of the structures of local minima and transition states.

Table S4: Absolute (in au) energies and free energies of UB3LYP/BS1 optimized geometries along the reaction mechanisms calculated for KDO5 in Gaussian-09. Gc is the free energy correction at 298 K.

Model 1	E [BS1, au]	ZPE [au]	G [au]	E [BS2, au]	Gc [au]
^{5}**Re**$_{A}$	-7650.646028	2.507816	-7648.378123	-7652.729653	2.267905
^{5}**TS1**$_{A,C5}$	-7650.610004	2.498905	-7648.353059	-7652.697867	2.256945
^{5}**IM1**$_{A,C5}$	-7650.641737	2.504120	-7648.375615	-7652.728843	2.266114
^{5}**TS2**$_{A,C5}$	-7650.621548	2.503945	-7648.356197	-7652.707399	2.265456
^{5}**Pr**$_{A,C5}$	-7650.729836	2.509469	-7648.456352	-7652.813251	2.273280
^{5}**TS1**$_{A,C4}$	-7650.605494	2.499527	-7648.343429	-7652.694329	2.262057
^{5}**IM1**$_{A,C4}$	-7650.642883	2.503369	-7648.377702	-7652.730585	2.265126
^{5}**TS2**$_{A,C4}$	-7650.625957	2.501926	-7648.362692	-7652.715353	2.262899
^{5}**Pr**$_{A,C4}$	-7650.735192	2.508804	-7648.464784	-7652.822205	2.270259
^{5}**TS1**$_{A,C3}$	-7650.607613	2.498632	-7648.353178	-7652.692831	2.254407
^{5}**IM1**$_{A,C3}$	-7650.641982	2.503959	-7648.376087	-7652.730180	2.265909
^{5}**TS2**$_{A,C3}$	-7650.637967	2.502395	-7648.374000	-7652.722477	2.263792
^{5}**Pr**$_{A,C3}$	-7650.719555	2.508531	-7648.447306	-7652.802216	2.272007
Model 2					
^{5}**Re**$_{B}$	-8063.345994	2.635052	-8060.956658	-8065.676410	2.389239
^{5}**TS1**$_{B,C5}$	-8063.314679	2.628298	-8060.923727	-8065.648193	2.390679
^{5}**IM1**$_{B,C5}$	-8063.350480	2.631786	-8060.965946	-8065.687672	2.384390
^{5}**TS2**$_{B,C5}$	-8063.337647	2.631481	-8060.946380	-8065.667092	2.391106
^{5}**Pr**$_{B,C5}$	-8063.430289	2.636468	-8061.039137	-8065.754705	2.391144
^{5}**TS1**$_{B,C4}$	-8063.310985	2.630503	-8060.920033	-8065.633251	2.390936
^{5}**IM1**$_{B,C4}$	-8063.354446	2.633997	-8060.964838	-8065.683408	2.389364
^{5}**TS2**$_{B,C4}$	-8063.334099	2.633260	-8060.943995	-8065.662965	2.389808
^{5}**Pr**$_{B,C4}$	-8063.442634	2.640735	-8061.039116	-8065.748459	2.403373
^{5}**TS1**$_{B,C3}$	-8063.307444	2.628488	-8060.919484	-8065.630661	2.387755
^{5}**IM1**$_{B,C3}$	-8063.364949	2.631786	-8060.975699	-8065.688797	2.389035
^{5}**TS2**$_{B,C3}$	-8063.344781	2.633608	-8060.943012	-8065.652929	2.401419
^{5}**Pr**$_{B,C3}$	-8063.430289	2.637738	-8061.031498	-8065.732990	2.399506
Model 3					
^{1}**Re**$_{C}$	-11045.049473	3.468631	-11041.848519	-11056.082383	3.201001
^{3}**Re**$_{C}$	-11045.076807	3.469389	-11041.876789	-11056.110260	3.199625
^{5}**Re**$_{C}$	-11045.085570	3.468591	-11041.888015	-11056.117861	3.197507
^{5}**TS1**$_{C,C5}$	-11045.040713	3.461841	-11041.848896	-11056.068558	3.191272
^{5}**IM1**$_{C,C5}$	-11045.046489	3.463608	-11041.846505	-11056.078657	3.199610
^{5}**Pr**$_{C,C5}$	-11045.134049	3.469995	-11041.930091	-11056.156385	3.203388
^{5}**TS1**$_{C,C4}$	-11045.043038	3.462502	-11041.851395	-11056.072437	3.191642
^{5}**IM1**$_{C,C4}$	-11045.047015	3.464278	-11041.849144	-11056.078397	3.197707
^{5}**Pr**$_{C,C4}$	-11045.132566	3.469799	-11041.927643	-11056.154066	3.204616
^{5}**TS1**$_{C,C3}$	-11045.041669	3.466475	-11041.842899	-11056.073214	3.198467
^{5}**IM1**$_{C,C3}$	-11045.046998	3.462083	-11041.859441	-11056.085439	3.187202
^{5}**TS2**$_{C,C3}$	-11045.043761	3.463368	-11041.853183	-11056.085486	3.190121

^{5}**Pr**$_{C,C3}$	-11045.085273	3.467007	-11041.891361	-11056.123567	3.193559

Table S5: Relative (in kcal mol^{-1}) energies and free energies of UB3LYP/BS1 optimized geometries along the reaction mechanisms calculated for KDO5 in Gaussian-09.

Model 1	ΔE [BS1]	ΔE+ZPE	ΔG [BS1]	ΔE [BS2]	ΔE+ZPE	ΔG [BS2]
^{5}**Re**$_A$	0.00	0.00	0.00	0.00	0.00	0.00
^{5}**TS1**$_{A,C5}$	22.61	17.01	15.73	19.95	14.35	13.07
^{5}**IM1**$_{A,C5}$	2.69	0.37	1.57	0.51	-1.81	-0.62
^{5}**TS2**$_{A,C5}$	15.36	12.93	13.76	13.96	11.54	12.43
^{5}**Pr**$_{A,C5}$	-52.59	-51.55	-49.09	-52.46	-51.42	-49.09
^{5}**TS1**$_{A,C4}$	25.44	20.23	21.77	22.17	16.96	18.50
^{5}**IM1**$_{A,C4}$	1.97	-0.82	0.26	-0.58	-3.38	-2.33
^{5}**TS2**$_{A,C4}$	12.59	8.90	9.68	8.97	5.28	5.83
^{5}**Pr**$_{A,C4}$	-55.95	-55.33	-54.38	-58.08	-57.46	-56.60
^{5}**TS1**$_{A,C3}$	24.11	18.34	15.65	23.11	17.34	14.64
^{5}**IM1**$_{A,C3}$	2.54	0.12	1.28	-0.33	-2.75	-1.58
^{5}**TS2**$_{A,C3}$	5.06	1.66	2.59	4.50	1.10	1.92
^{5}**Pr**$_{A,C3}$	-46.14	-45.69	-43.41	-45.53	-45.09	-42.96
Model 2						
^{5}**Re**$_B$	0.00	0.00	0.00	0.00	0.00	0.00
^{5}**TS1**$_{B,C5}$	19.65	15.41	20.66	17.71	13.47	18.61
^{5}**IM1**$_{B,C5}$	-2.82	-4.86	-5.83	-7.07	-9.12	-10.11
^{5}**TS2**$_{B,C5}$	5.24	3.00	6.45	5.85	3.61	7.02
^{5}**Pr**$_{B,C5}$	-52.90	-52.01	-51.76	-49.13	-48.24	-47.94
^{5}**TS1**$_{B,C4}$	21.97	19.11	22.98	27.08	24.23	28.15
^{5}**IM1**$_{B,C4}$	-5.30	-5.97	-5.13	-4.39	-5.05	-4.31
^{5}**TS2**$_{B,C4}$	7.46	6.34	7.95	8.44	7.31	8.79
^{5}**Pr**$_{B,C4}$	-60.64	-57.08	-51.74	-45.21	-41.65	-36.34
^{5}**TS1**$_{B,C3}$	24.19	20.07	23.33	28.71	24.59	27.78
^{5}**IM1**$_{B,C3}$	-11.89	-13.94	-11.95	-7.77	-9.82	-7.90
^{5}**TS2**$_{B,C3}$	0.76	-0.14	8.56	14.73	13.83	22.38
^{5}**Pr**$_{B,C3}$	-52.90	-51.21	-46.96	-35.50	-33.82	-29.06
Model 3						
^{1}**Re**$_C$	22.65	22.68	24.78	22.26	22.29	24.46
^{3}**Re**$_C$	5.50	6.00	7.04	4.77	5.27	6.10
^{5}**Re**$_C$	0.00	0.00	0.00	0.00	0.00	0.00
^{5}**TS1**$_{C,C5}$	28.15	23.91	24.55	30.94	26.70	27.03
^{5}**IM1**$_{C,C5}$	24.52	21.40	26.05	24.60	21.47	25.92
^{5}**Pr**$_{C,C5}$	-30.42	-29.54	-26.40	-24.17	-23.29	-20.48
^{5}**TS1**$_{C,C4}$	26.69	22.87	22.98	28.50	24.68	24.82
^{5}**IM1**$_{C,C4}$	24.19	21.49	24.39	24.76	22.06	24.89
^{5}**Pr**$_{C,C4}$	-29.49	-28.73	-24.87	-22.72	-21.96	-18.26
^{5}**TS1**$_{C,C3}$	27.55	26.22	28.31	28.02	26.69	28.62
^{5}**IM1**$_{C,C3}$	24.20	20.12	17.93	20.35	16.26	13.88

^{5}**TS2**$_{C,C3}$	26.23	22.96	21.86	20.32	17.04	15.68
^{5}**Pr**$_{C,C3}$	0.19	-0.81	-2.10	-3.58	-4.58	-6.06

Table S6: Group charges and group spin densities of UB3LYP/BS1 optimized geometries along the reaction mechanisms calculated for KDO5 in Gaussian-09.

Charge	Fe	O	Succ	SubH	Prot	Wat	Total
Model 1							
^{5}**Re**$_{A}$	0.71	-0.40	-0.34	0.71	-1.68	0.00	-1.00
^{5}**TS1**$_{A,C5}$	0.80	-0.61	-0.38	0.95	-1.77	0.00	-1.00
^{5}**IM1**$_{A,C5}$	0.81	-0.81	-0.41	1.20	-1.80	0.00	-1.00
^{5}**TS2**$_{A,C5}$	0.61	-0.77	-0.48	1.49	-1.85	0.00	-1.00
^{5}**Pr**$_{A,C5}$	0.58	-0.69	-0.49	1.50	-1.90	0.00	-1.00
^{5}**TS1**$_{A,C4}$	0.80	-0.60	-0.36	0.91	-1.75	0.00	-1.00
^{5}**IM1**$_{A,C4}$	0.81	-0.81	-0.40	1.19	-1.80	0.00	-1.00
^{5}**TS2**$_{A,C4}$	0.81	-0.78	-0.36	1.17	-1.83	0.00	-1.00
^{5}**Pr**$_{A,C4}$	0.56	-0.67	-0.55	1.47	-1.81	0.00	-1.00
^{5}**TS1**$_{A,C3}$	0.80	-0.63	-0.36	0.97	-1.79	0.00	-1.00
^{5}**IM1**$_{A,C3}$	0.81	-0.81	-0.40	1.18	-1.79	0.00	-1.00
^{5}**TS2**$_{A,C3}$	0.63	-0.74	-0.47	1.51	-1.94	0.00	-1.00
^{5}**Pr**$_{A,C3}$	0.62	-0.68	-0.47	1.49	-1.96	0.00	-1.00
Model 2							
^{5}**Re**$_{B}$	0.83	-0.50	-0.42	0.72	-0.64	0.00	0.00
^{5}**TS1**$_{B,C5}$	0.84	-0.62	-0.35	0.91	-0.78	0.00	0.00
^{5}**IM1**$_{B,C5}$	0.87	-0.81	-0.36	1.12	-0.82	0.00	0.00
^{5}**TS2**$_{B,C5}$	0.61	-0.77	-0.49	1.62	-0.97	0.00	0.00
^{5}**Pr**$_{B,C5}$	0.80	-0.72	-0.54	1.39	-0.94	0.00	0.00
^{5}**TS1**$_{B,C4}$	0.70	-0.70	-0.36	1.09	-0.73	0.00	0.00
^{5}**IM1**$_{B,C4}$	0.79	-0.80	-0.47	1.13	-0.66	0.00	0.00
^{5}**TS2**$_{B,C4}$	0.83	-0.78	-0.51	1.32	-0.86	0.00	0.00
^{5}**Pr**$_{B,C4}$	0.72	-0.69	-0.52	1.40	-0.91	0.00	0.00
^{5}**TS1**$_{B,C3}$	0.80	-0.65	-0.37	1.03	-0.81	0.00	0.00
^{5}**IM1**$_{B,C3}$	0.85	-0.81	-0.37	1.12	-0.79	0.00	0.00
^{5}**TS2**$_{B,C3}$	0.74	-0.73	-0.42	1.30	-0.89	0.00	0.00
^{5}**Pr**$_{B,C3}$	0.65	-0.66	-0.46	1.44	-0.98	0.00	0.00
Model 3							
^{5}**Re**$_{C}$	0.89	-0.72	-0.48	0.06	1.94	0.30	2.00
^{1}**Re**$_{C}$	0.76	-1.06	-0.55	0.12	2.76	-0.02	2.00
^{3}**Re**$_{C}$	0.96	-1.05	-0.54	0.11	2.51	0.01	2.00
^{7}**Re**$_{C}$	1.08	-1.06	-0.59	0.10	2.47	0.00	2.00
^{5}**TS1**$_{C,C5}$	0.75	-0.62	-0.52	0.20	1.84	0.35	2.00
^{5}**IM1**$_{C,C5}$	0.89	-0.53	-0.44	0.27	1.21	0.59	2.00
^{5}**Pr**$_{C,C5}$	0.81	-0.43	-0.48	0.56	1.22	0.32	2.00
^{5}**TS1**$_{C,C4}$	0.75	-0.65	-0.49	0.01	2.00	0.38	2.00
^{5}**IM1**$_{C,C4}$	0.89	-0.54	-0.44	0.19	1.57	0.34	2.00

^{5}**Pr**$_{C,C4}$	0.85	-0.44	-0.52	0.48	1.27	0.36	2.00
^{5}**TS1**$_{C,C3}$	0.74	-0.63	-0.50	-0.03	2.06	0.36	2.00
^{5}**IM1**$_{C,C3}$	0.81	-0.58	-0.53	0.13	1.96	0.21	2.00
^{5}**Pr**$_{C,C3}$	0.75	-0.49	-0.54	0.59	1.37	0.33	2.00
^{5}**Re**$_{C,PBE0}$	0.99	-1.05	-0.57	0.04	2.58	0.01	2.00
^{3}**Re**$_{C,PBE0}$	0.82	-1.02	-0.50	0.05	2.63	0.02	2.00
^{5}**TS1**$_{C,C3,PBE0}$	0.73	-0.93	-0.56	0.05	2.69	0.03	2.00
^{5}**TS1**$_{C,C4,PBE0}$	0.93	-1.04	-0.63	-0.05	2.72	0.07	2.00
^{5}**TS1**$_{C,C5,PBE0}$	0.88	-0.98	-0.65	0.25	2.43	0.07	2.00
^{5}**Re**$_{C,Solv}$	1.13	-1.07	-0.59	0.11	2.43	0.00	2.00
^{5}**TS1**$_{C,C3, Solv}$	0.87	-0.97	-0.58	0.12	2.55	0.01	2.00
^{5}**TS1**$_{C,C4, Solv}$	1.10	-1.07	-0.65	0.02	2.56	0.05	2.00
^{5}**TS1**$_{C,C5, Solv}$	1.05	-1.02	-0.67	0.31	2.28	0.05	2.00
Spin density	Fe	O	Succ	SubH	Prot	Wat	Total
Model 1							
^{5}**Re**$_{A}$	3.00	0.67	0.14	0.00	0.19	0.00	4.00
^{5}**TS1**$_{A,C5}$	3.89	0.01	0.20	-0.36	0.26	0.00	4.00
^{5}**IM1**$_{A,C5}$	4.05	0.44	0.19	-0.99	0.31	0.00	4.00
^{5}**TS2**$_{A,C5}$	3.73	-0.06	0.08	0.14	0.12	0.00	4.00
^{5}**Pr**$_{A,C5}$	3.75	0.02	0.08	0.00	0.14	0.00	4.00
^{5}**TS1**$_{A,C4}$	3.81	0.06	0.19	-0.31	0.25	0.00	4.00
^{5}**IM1**$_{A,C4}$	4.05	0.44	0.19	-0.99	0.31	0.00	4.00
^{5}**TS2**$_{A,C4}$	4.06	0.42	0.21	-0.99	0.31	0.00	4.00
^{5}**Pr**$_{A,C4}$	3.76	0.02	0.07	0.00	0.15	0.00	4.00
^{5}**TS1**$_{A,C3}$	3.82	0.13	0.17	-0.39	0.26	0.00	4.00
^{5}**IM1**$_{A,C3}$	4.05	0.43	0.19	-0.99	0.31	0.00	4.00
^{5}**TS2**$_{A,C3}$	3.71	-0.15	0.07	0.26	0.12	0.00	4.00
^{5}**Pr**$_{A,C3}$	3.77	0.01	0.08	0.01	0.14	0.00	4.00
Model 2							
^{5}**Re**$_{B}$	2.98	0.68	0.14	0.00	0.19	0.00	4.00
^{5}**TS1**$_{B,C5}$	3.77	0.02	0.18	-0.25	0.28	0.00	4.00
^{5}**IM1**$_{B,C5}$	4.07	0.31	0.23	-0.99	0.38	0.00	4.00
^{5}**TS2**$_{B,C5}$	3.74	0.00	0.07	0.04	0.15	0.00	4.00
^{5}**Pr**$_{B,C5}$	3.69	0.00	0.11	0.00	0.20	0.00	4.00
^{5}**TS1**$_{B,C4}$	2.83	0.37	0.07	0.64	0.10	0.00	4.00
^{5}**IM1**$_{B,C4}$	2.75	0.07	0.02	0.99	0.16	0.00	4.00
^{5}**TS2**$_{B,C4}$	3.94	0.38	0.14	-0.72	0.25	0.00	4.00
^{5}**Pr**$_{B,C4}$	3.74	0.00	0.09	0.00	0.16	0.00	4.00
^{5}**TS1**$_{B,C3}$	3.95	0.09	0.16	-0.49	0.29	0.00	4.00
^{5}**IM1**$_{B,C3}$	4.08	0.35	0.18	-0.99	0.39	0.00	4.00
^{5}**TS2**$_{B,C3}$	3.93	0.36	0.11	-0.63	0.22	0.00	4.00
^{5}**Pr**$_{B,C3}$	3.76	0.01	0.07	0.00	0.15	0.00	4.00
Model 3							

^{5}**Re**$_{C}$	4.14	0.51	0.11	0.00	-0.78	0.01	4.00
^{1}**Re**$_{C}$	0.93	0.11	-0.01	0.00	-1.03	0.00	0.00
^{3}**Re**$_{C}$	2.75	0.23	-0.03	0.00	-0.96	0.01	2.00
^{7}**Re**$_{C}$	4.14	0.51	0.11	0.00	1.22	0.02	6.00
^{5}**TS1**$_{C,C5}$	3.61	-0.40	0.00	-0.32	1.10	0.01	4.00
^{5}**IM1**$_{C,C5}$	4.25	0.21	0.15	-0.95	0.36	-0.03	4.00
^{5}**Pr**$_{C,C5}$	3.78	0.00	0.06	0.00	0.16	0.00	4.00
^{5}**TS1**$_{C,C4}$	4.11	0.44	0.09	0.14	-0.79	0.01	4.00
^{5}**IM1**$_{C,C4}$	4.26	0.19	0.14	-0.96	0.38	0.00	4.00
^{5}**Pr**$_{C,C4}$	3.80	0.00	0.05	0.00	0.12	0.02	4.00
^{5}**TS1**$_{C,C3}$	2.87	0.13	-0.03	-0.02	1.04	0.01	4.00
^{5}**IM1**$_{C,C3}$	3.81	0.03	0.05	-1.04	1.15	0.00	4.00
^{5}**Pr**$_{C,C3}$	3.80	0.02	0.05	0.01	0.12	0.00	4.00
^{5}**Re**$_{C,PBE0}$	4.23	0.47	0.09	0.00	-0.82	0.02	4.00
^{3}**Re**$_{C,PBE0}$	2.85	0.17	-0.04	0.00	-0.98	0.01	2.00
^{5}**TS1**$_{C,C3,PBE0}$	2.99	0.04	-0.03	-0.04	1.01	0.02	4.00
^{5}**TS1**$_{C,C4,PBE0}$	4.20	0.41	0.07	0.13	-0.83	0.01	4.00
^{5}**TS1**$_{C,C5,PBE0}$	3.71	-0.45	0.00	-0.35	1.08	0.01	4.00
^{5}**Re**$_{C,Solv}$	4.15	0.50	0.11	0.00	-0.78	0.02	4.00
^{5}**TS1**$_{C,C3, Solv}$	2.85	0.14	-0.02	-0.01	1.04	0.02	4.00
^{5}**TS1**$_{C,C4, Solv}$	4.12	0.43	0.09	0.13	-0.79	0.01	4.00
^{5}**TS1**$_{C,C5, Solv}$	3.58	-0.38	0.00	-0.32	1.10	0.01	4.00

Table S7: UPBE0/BS2 single-point calculations on the optimized UB3LYP/BS1 geometries for model C. ZPE, thermal and entropic corrections taken from the UB3LYP/BS1 frequency calculations.

	E [BS2, au]	ΔE+ZPE [kcal mol^{-1}]	ΔG [kcal mol^{-1}]
^{3}**Re**$_{C,PBE0}$	-11044.291712	8.93	9.76
^{5}**Re**$_{C,PBE0}$	-11044.305142	0.00	0.00
^{5}**TS1**$_{C,C3,PBE0}$	-11044.256874	28.96	30.89
^{5}**TS1**$_{C,C4,PBE0}$	-11044.262420	22.99	23.13
^{5}**TS1**$_{C,C5,PBE0}$	-11044.255177	27.12	27.44

Table S8: UB3LYP/BS2(solvent) single-point calculations on the optimized UB3LYP/BS1 geometries for model C. Self-consistent reaction field model with dielectric constant for chlorobenzene used. ZPE, thermal and entropic corrections taken from the UB3LYP/BS1 frequency calculations.

	E [BS2, au]	ΔE+ZPE [kcal mol^{-1}]	ΔG [kcal mol^{-1}]
^{5}**Re**$_{C,Solv}$	-11056.298612	0.00	0.00
^{5}**TS1**$_{C,C3, Solv}$	-11056.251448	28.27	30.20
^{5}**TS1**$_{C,C4, Solv}$	-11056.251355	25.83	25.97
^{5}**TS1**$_{C,C5, Solv}$	-11056.248900	26.96	27.28

Table S9: UB3LYP/BS2 calculated triplet-quintet energies for model A and B. Triplet and quintet spin geometry optimizations done.

	E [BS2, au]	ΔE [kcal mol^{-1}]
^{5}**Re**$_{A}$	-7650.646028	0.00
^{3}**Re**$_{A}$	-7650.639200	4.28
^{5}**Re**$_{B}$	-9198.999783	0.00
^{3}**Re**$_{B}$	-9198.984363	9.68

Table S10: Absolute (in au) energies of UB3LYP/6-311+G* calculated diabatic BDE values for an isolated lysine molecule for the C-H bond strength for the C_3, C_4 and C_5 position under an applied electric field ranging from -200 au to 200 au along the X, Y or Z axis.

Field direction and strength	E [6-311+G*, au]			
	Reactant	C_3-radical	C_4-radical	C_5-radical
X-200	-497.687	-497.037	-497.040	-497.030
X-150	-497.636	-496.987	-496.989	-496.982
X-100	-497.588	-496.940	-496.941	-496.937
X-50	-497.542	-496.896	-496.895	-496.895
X-0	-497.500	-496.854	-496.853	-496.855
X+50	-497.460	-496.816	-496.813	-496.819
X+100	-497.423	-496.781	-496.777	-496.789
X+150	-497.395	-496.764	-496.754	-496.771
X+200	-497.388	-496.772	-496.758	-496.773
Y-200	-497.492	-496.857	-496.852	-496.850
Y-150	-497.490	-496.852	-496.849	-496.848
Y-100	-497.491	-496.851	-496.848	-496.848
Y-50	-497.494	-496.852	-496.849	-496.851
Y-0	-497.500	-496.854	-496.853	-496.855
Y+50	-497.507	-496.860	-496.859	-496.862
Y+100	-497.516	-496.867	-496.866	-496.871
Y+150	-497.528	-496.877	-496.876	-496.883
Y+200	-497.542	-496.889	-496.888	-496.896
Z-200	-497.510	-496.872	-496.869	-496.862
Z-150	-497.505	-496.865	-496.862	-496.857
Z-100	-497.501	-496.859	-496.857	-496.854
Z-50	-497.499	-496.856	-496.854	-496.854
Z-0	-497.500	-496.854	-496.853	-496.855
Z+50	-497.502	-496.855	-496.854	-496.859
Z+100	-497.506	-496.858	-496.856	-496.864
Z+150	-497.512	-496.862	-496.861	-496.871
Z+200	-497.520	-496.869	-496.868	-496.881

Table S11: Relative (in kcal/mol) energies of UB3LYP/6-311+G* calculated diabatic BDE values for an isolated lysine molecule for the C-H bond strength for the C_3, C_4 and C_5 position under an applied electric field ranging from -200 au to 200 au along the X, Y or Z axis.

Electric Field Strength	BDE [6-311+G*, kcal/mol]		
[in au]	C_3	C_4	C_5
X-200	93.15	91.25	97.56
X-150	92.63	91.44	95.81
X-100	92.04	91.57	94.01
X-50	91.37	91.62	92.12
X-0	90.60	91.60	90.10
X+50	89.71	91.47	87.76
X+100	88.24	91.17	83.72
X+150	81.99	87.71	77.56
X+200	72.49	81.10	71.62
Y-200	84.34	87.06	88.41
Y-150	86.10	88.24	89.04
Y-100	87.70	89.39	89.48
Y-50	89.19	90.51	89.82
Y-0	90.60	91.60	90.10
Y+50	91.93	92.67	90.35
Y+100	93.20	93.71	90.56
Y+150	94.40	94.74	90.76
Y+200	95.54	95.75	90.95
Z-200	86.40	88.04	92.87
Z-150	87.51	88.96	92.22
Z-100	88.58	89.86	91.55
Z-50	89.61	90.74	90.84
Z-0	90.60	91.60	90.10
Z+50	91.55	92.45	89.34
Z+100	92.45	93.28	88.55
Z+150	93.30	94.10	87.72
Z+200	94.11	94.90	86.86

Table S12: Absolute (in au) energies and unpaired spin contribution on Tyr_{233} of UB3LYP/BS1 geometries for the reactant cluster model C and model C2.

	E [BS1, au]	spin on Tyr
^{5}**Re**$_{C}$	-11045.086	-0.97
^{5}**Re**$_{C2}$	-11517.444	-0.97

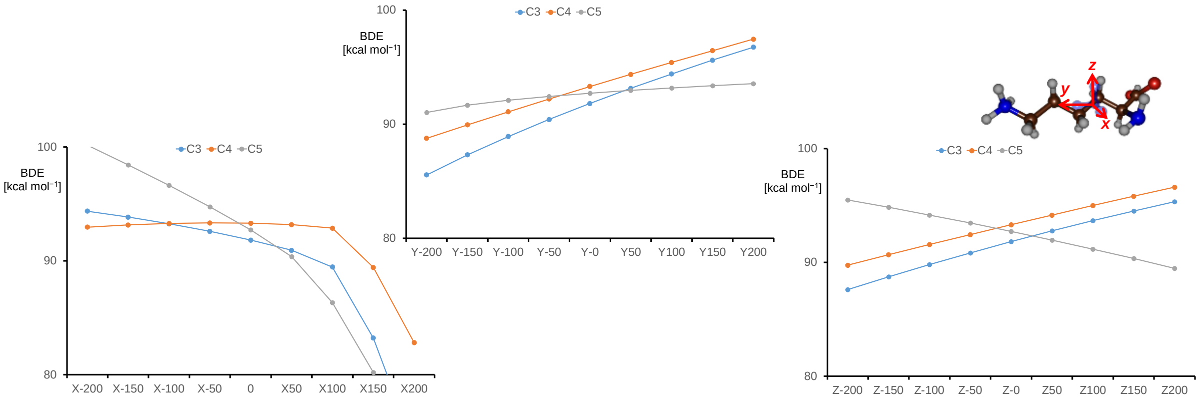

Applied Electric Field [in au]

Figure S7: Electric field effects on the BDE values for the C_3−H, C_4−H and C_5−H bonds of an isolated substrate molecule with an electric field applied along the x-, y- or z-direction (directions defined as in Gaussian).

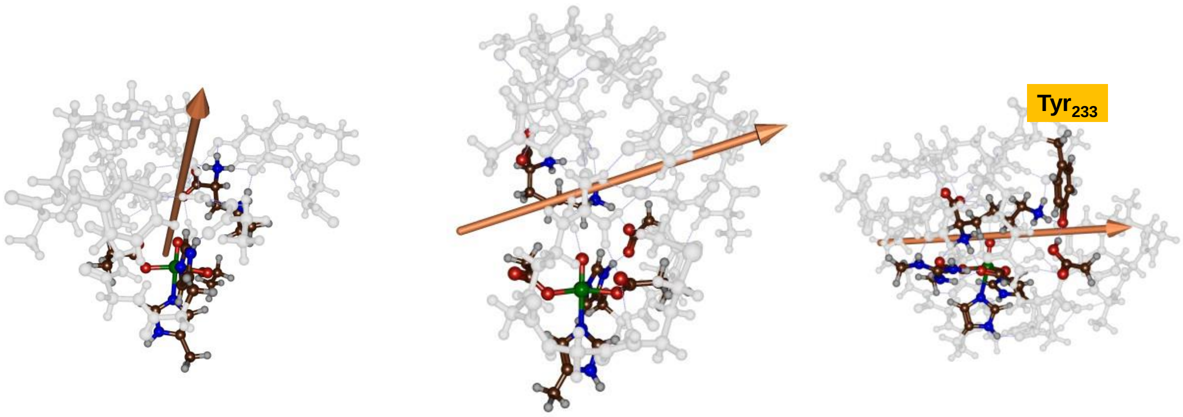

Dipole Moment in ^{5}**Re$_{A}$** Dipole Moment in ^{5}**Re$_{B}$** Dipole Moment in ^{5}**Re**$_{C}$

Figure S8: Electric dipole moment vectors in ^{5}**Re**$_{A}$, ^{5}**Re**$_{B}$ and ^{5}**Re**$_{C}$.

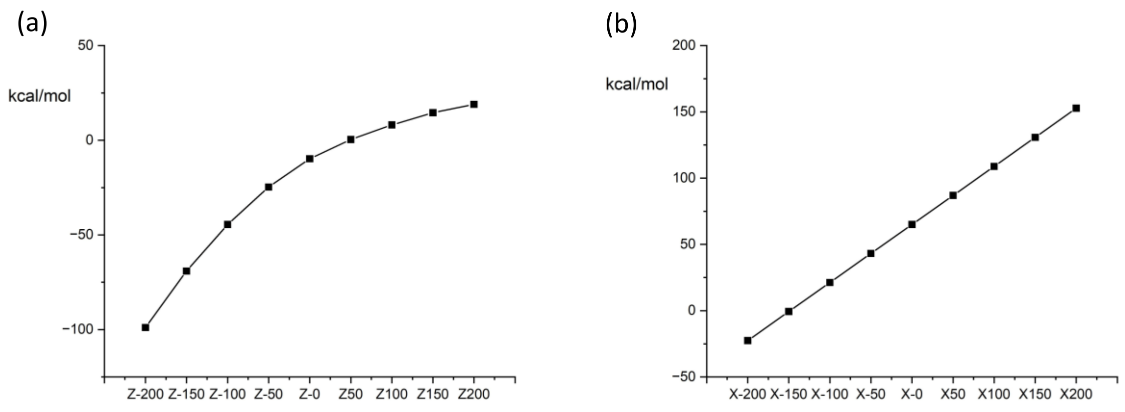

Figure S9: (a) Electric field effects on the iron(IV)-oxo reduction potential along the z-coordinate as calculated from the quintet/sextet energy gap. **(b)** Ionization potential of the p-cresol-acetate couple and its ability to perform PCET as calculated from the singlet/doublet energy gap.

Cartesian coordinates of optimized geometries:

Model 1 structures:

^{5}**Re**$_{A}$

C	-0.29965	2.945358	-4.97702
H	-1.31963	2.565371	-5.12216
C	-0.30546	4.267329	-4.20365
H	0.712471	4.663969	-4.12657
H	-0.88654	5.021322	-4.75204
C	-0.87812	4.112208	-2.7889
H	-1.89053	3.690537	-2.82541
H	-0.26057	3.400856	-2.22159
C	-0.90951	5.422891	-2.0145
O	-0.07107	6.317318	-2.20906
N	-1.9027	5.560338	-1.10777
H	-2.54684	4.796861	-0.91057
H	-1.91493	6.396984	-0.5306
C	-5.4134	8.980307	-0.31534
H	-6.30099	8.866833	-0.94957
C	-5.20849	7.767871	0.597373
H	-5.07594	6.866644	-0.01716
H	-4.26448	7.891564	1.140952
C	-6.33297	7.529943	1.564422
N	-6.31975	6.458347	2.45006
C	-7.44764	6.5237	3.131367
H	-7.7646	5.833412	3.901334
N	-8.19541	7.587566	2.740632
H	-9.09994	7.84855	3.104808
C	-7.49706	8.238785	1.738838
H	-7.88702	9.118777	1.252231
C	3.629824	-7.30228	2.476803
C	4.66022	-6.19797	2.72417
C	2.454265	-7.00273	1.524424
O	5.231311	-6.12014	3.809324
C	1.674473	-5.76858	1.87772
C	0.87233	-5.42938	2.943849
N	1.693771	-4.65101	1.064841
C	0.92804	-3.70304	1.637685
N	0.411322	-4.13887	2.785167
H	4.176381	-8.17893	2.100515
H	2.814148	-6.89666	0.496316
H	1.790758	-7.87685	1.530653
H	0.598488	-6.01748	3.807873
H	0.750809	-2.73165	1.202335
H	2.284057	-4.58459	0.188534
N	4.941173	-5.3449	1.689602

H	4.494878	-5.43272	0.775401
C	6.007842	-4.36962	1.822577
H	6.71119	-4.72445	2.579938
C	5.616934	-2.93563	2.214957
O	6.498968	-2.07809	2.296655
N	4.29947	-2.69926	2.413999
C	3.711188	-1.3788	2.669376
C	4.401164	-0.29711	1.806851
C	3.670983	-0.99436	4.157785
O	4.80185	0.762305	2.2736
C	2.682465	0.141033	4.472788
C	1.19158	-0.16326	4.270769
O	0.39644	0.805097	4.200515
O	0.850011	-1.39718	4.210476
H	3.671631	-3.48603	2.310466
H	2.678265	-1.45406	2.309344
H	3.404457	-1.89381	4.721668
H	4.676179	-0.68897	4.46443
H	2.936217	1.031766	3.892166
H	2.79268	0.432254	5.527782
N	4.510973	-0.64211	0.489604
H	3.820181	-1.30195	0.142778
C	4.820035	0.35367	-0.53117
H	4.954196	-0.2247	-1.45595
C	3.671728	1.340623	-0.76119
H	3.477954	1.887086	0.169228
H	3.96515	2.083909	-1.5055
C	2.370547	0.657515	-1.22395
O	1.533693	1.39632	-1.81498
O	2.226946	-0.57817	-0.96685
C	6.180079	1.025866	-0.30832
O	6.446673	2.119232	-0.82022
N	7.123526	0.270089	0.319537
H	6.822805	-0.53387	0.868683
C	8.468811	0.789938	0.543149
H	8.390768	1.848845	0.815526
C	9.162789	0.02255	1.672083
H	9.203097	-1.05037	1.453667
H	8.617349	0.151904	2.611337
H	10.18245	0.398932	1.78553
C	9.341811	0.790019	-0.73398
O	10.44651	1.323216	-0.73792
N	8.783718	0.179021	-1.81614
H	7.877163	-0.24989	-1.68442
C	9.330242	0.219167	-3.15622
H	10.40286	0.415444	-3.05411
C	9.13669	-1.14291	-3.87325

H	9.771324	-1.88158	-3.37035
H	9.504466	-1.01568	-4.895
C	7.696525	-1.61806	-3.87591
C	7.208947	-2.43993	-2.8472
H	7.894644	-2.79268	-2.079
C	5.860482	-2.80541	-2.7906
H	5.493747	-3.43081	-1.98054
C	4.974032	-2.36366	-3.77691
H	3.924493	-2.64056	-3.72482
C	5.453195	-1.56699	-4.82097
H	4.773411	-1.22482	-5.59709
C	6.798637	-1.19562	-4.86899
H	7.161486	-0.56066	-5.67276
C	8.786553	1.378004	-4.03547
O	9.120965	1.434985	-5.22245
N	7.994898	2.284182	-3.41935
H	7.721222	2.144022	-2.44974
C	7.504044	3.489491	-4.07597
H	7.976585	3.527144	-5.05952
C	5.971429	3.555221	-4.17813
H	5.556881	3.492797	-3.16309
H	5.70873	4.55273	-4.56438
C	5.303363	2.48331	-5.06027
H	5.562112	1.500479	-4.6432
C	3.773809	2.626466	-5.00621
H	3.285302	1.860567	-5.62093
H	3.383852	2.523403	-3.98673
H	3.45761	3.606801	-5.38967
C	5.806901	2.523253	-6.51207
H	6.877464	2.30262	-6.57706
H	5.276583	1.783906	-7.12579
H	5.629692	3.510068	-6.96318
C	-4.66337	-2.78538	-6.06582
H	-3.86083	-3.45713	-6.38526
C	-4.22927	-2.00069	-4.81953
H	-3.36961	-1.36409	-5.06601
H	-5.03401	-1.3288	-4.50247
C	-3.83889	-2.86979	-3.61836
O	-3.89976	-4.12364	-3.74049
O	-3.48938	-2.25312	-2.5605
C	-5.96352	-3.55135	-5.84511
O	-7.0373	-2.98331	-5.63709
N	-5.86388	-4.91441	-5.91067
H	-4.93426	-5.29801	-5.80282
C	-6.99865	-5.76593	-5.60556
H	-7.8773	-5.37353	-6.12299
C	-7.40203	-5.90366	-4.12506

O	-8.41143	-6.53313	-3.8311
N	-6.5958	-5.28279	-3.22074
H	-5.7415	-4.82281	-3.53227
C	-6.90208	-5.28775	-1.79843
H	-7.381	-6.24127	-1.55732
C	-5.6201	-5.14156	-0.9566
H	-5.94066	-5.07355	0.086815
H	-5.11135	-4.20988	-1.2281
C	-4.68666	-6.34717	-1.08074
O	-4.96968	-7.43701	-0.60819
N	-3.48581	-6.09308	-1.72332
H	-3.50611	-5.37341	-2.46095
H	-2.976	-6.9401	-1.95501
C	-7.90508	-4.19612	-1.35301
O	-8.30751	-4.18639	-0.19127
N	-8.27901	-3.27262	-2.2748
H	-7.96333	-3.36312	-3.23371
C	-9.31785	-2.30193	-1.95202
H	-9.11977	-1.92778	-0.94277
C	-9.40041	-1.14992	-2.96354
H	-9.52855	-1.56463	-3.9716
H	-10.33	-0.60607	-2.74123
C	-8.23356	-0.17969	-2.96617
C	-7.4948	0.049154	-4.13491
H	-7.72737	-0.5178	-5.03192
C	-6.4332	0.952774	-4.15835
H	-5.86272	1.12262	-5.06722
C	-6.06993	1.642921	-2.99629
O	-4.97922	2.463939	-3.05573
H	-4.70473	2.769517	-2.14316
C	-6.81087	1.443892	-1.82201
H	-6.54075	1.987902	-0.9206
C	-7.87888	0.545187	-1.81984
H	-8.43942	0.40392	-0.89781
C	4.577361	-7.04887	-2.45185
H	5.320923	-6.6368	-1.76324
C	3.735053	-5.93544	-3.07622
H	4.379006	-5.27447	-3.67235
H	2.993799	-6.34007	-3.7742
C	2.983007	-5.03908	-2.07871
O	1.976652	-4.43022	-2.50791
O	3.447052	-4.95998	-0.88449
C	0.646894	-6.54132	8.441942
C	0.656163	-5.37636	7.507636
C	-0.227	-4.95769	6.543699
N	1.677987	-4.4395	7.474046
C	1.385635	-3.52131	6.514789

N	0.23784	-3.80817	5.935188
H	0.64765	-6.22674	9.493857
H	-0.25308	-7.13986	8.276125
H	-1.16615	-5.39764	6.241266
H	2.015991	-2.68172	6.266735
H	2.517528	-4.45477	8.035355
C	-1.10978	9.402791	2.800423
H	-0.31373	10.08645	3.118116
C	-1.17532	8.199712	3.770863
H	-0.2373	7.631064	3.714717
H	-1.23751	8.594566	4.794436
C	-2.36108	7.275335	3.48256
H	-3.28952	7.865176	3.48675
H	-2.25524	6.888321	2.466265
C	-2.53607	6.084107	4.434802
H	-1.61052	5.518456	4.568105
H	-2.85105	6.406089	5.433676
N	-3.58038	5.178249	3.918806
H	-4.49242	5.62462	3.858718
C	-3.32635	4.420612	2.806316
N	-2.13501	3.886697	2.600921
H	-1.40817	3.819006	3.335142
H	-1.9977	3.323146	1.749224
N	-4.33813	4.226326	1.942093
H	-5.12197	4.878912	1.995449
H	-4.1665	3.761036	1.046306
C	-0.9155	9.00085	1.336492
O	-1.7844	8.378216	0.710932
N	0.258168	9.39437	0.786116
H	1.003569	9.735529	1.384152
C	0.726125	8.95517	-0.51909
H	0.025232	8.235534	-0.94367
C	2.128089	8.358905	-0.32726
O	2.889327	8.849657	0.512834
N	2.417571	7.30653	-1.12161
H	1.650626	6.897076	-1.65855
C	3.634327	6.517516	-0.94989
H	4.345702	7.143012	-0.40487
C	3.379132	5.19461	-0.20289
H	2.687118	4.581114	-0.79302
H	4.319993	4.634043	-0.14385
C	2.811014	5.413631	1.203082
H	3.531034	5.94972	1.830612
H	1.900916	6.02057	1.162007
S	2.424562	3.868733	2.115577
C	1.005805	3.233477	1.14773
H	0.586088	2.402509	1.719394

H	0.239801	4.004771	1.031939
H	1.302215	2.860608	0.16562
O	-2.3548	-1.86599	5.418105
C	-2.95361	-2.95849	5.20663
O	-2.42048	-3.86959	4.484832
C	-4.32012	-3.23436	5.819415
C	-4.95159	-2.03733	6.529592
H	-4.97002	-3.61436	5.021184
H	-4.19204	-4.07639	6.513867
H	-5.11659	-1.20796	5.833933
H	-5.91861	-2.31298	6.965832
O	-1.57747	-1.91047	2.214974
O	-2.1946	0.232737	3.598531
Fe	-0.6101	-2.80094	4.129663
C	-2.50142	-0.38945	-0.00734
H	-1.82747	0.135473	0.66938
H	-1.86005	-0.97397	-0.66966
C	-3.25321	0.666482	-0.83136
H	-4.33592	0.53581	-0.73828
C	-2.94184	2.127051	-0.3903
O	-3.79275	3.026893	-0.73777
O	-1.93312	2.327811	0.304203
H	-0.9825	-3.09623	-0.68137
N	-2.9529	0.468634	-2.29011
H	-1.89119	0.419581	-2.49714
H	-3.43214	1.179606	-2.86121
H	-3.2956	-0.48696	-2.55185
C	-3.46615	-1.2791	0.789483
H	-4.01373	-0.64044	1.493352
C	-2.77588	-2.39265	1.59674
C	-2.48884	-3.67785	0.814625
H	-1.79468	-4.29634	1.384684
H	-3.42544	-4.22812	0.704443
N	-1.93904	-3.54208	-0.58011
H	-1.88795	-4.49047	-0.97442
H	-2.58159	-3.03056	-1.23909
H	1.51442	-7.19664	8.290137
H	3.257819	-7.57301	3.469302
H	5.105102	-7.62112	-3.2245
H	3.954497	-7.75123	-1.88419
H	6.544845	-4.28606	0.872161
H	7.856886	4.359652	-3.50565
H	-4.30159	-1.66861	7.328614
H	-4.85084	-2.07689	-6.88127
H	-6.80749	-6.77028	-5.99543
H	-10.2931	-2.80854	-1.90738
H	-4.54315	9.106184	-0.9656

H	-5.52776	9.902581	0.267605
H	-2.05445	9.958559	2.85611
H	0.798325	9.812033	-1.20362
H	4.058264	6.301046	-1.93746
H	0.148442	3.074479	-5.96952
H	0.266194	2.17266	-4.44635
H	-2.4446	-0.31105	4.375255
H	-1.27213	0.525672	3.823915
O	-0.42669	-0.02108	-2.9026
H	-0.22062	-0.94435	-2.64455
H	0.303049	0.525646	-2.46889
O	0.026671	3.607848	4.401375
H	0.203813	2.64166	4.464477
H	0.799222	3.945774	3.903025
O	0.393854	-2.43704	-1.46374
H	0.986895	-3.14264	-1.84464
H	1.017141	-1.69313	-1.21191
H	-1.80182	-1.01655	2.635769
H	-4.21664	-1.7307	0.125984
H	-3.46555	-2.72786	2.383323

^{5}**TS1**$_{A,C3}$

C	-2.05171	1.662626	-5.34794
H	-3.08995	1.306121	-5.3033
C	-1.93689	3.085955	-4.79104
H	-0.91064	3.45201	-4.90833
H	-2.57053	3.773115	-5.36534
C	-2.32651	3.160006	-3.31119
H	-3.32892	2.739004	-3.15707
H	-1.639	2.544213	-2.71533
C	-2.30608	4.577241	-2.74933
O	-1.84712	5.531451	-3.39589
N	-2.82718	4.734203	-1.51013
H	-3.14405	3.938744	-0.96078
H	-2.86021	5.671137	-1.11604
C	-6.83277	7.10689	-1.22541
H	-7.65867	6.524374	-1.65107
C	-6.26063	6.443592	0.030629
H	-5.87411	5.448231	-0.22772
H	-5.38719	7.015068	0.366497
C	-7.24653	6.302875	1.155234
N	-6.90642	5.682566	2.352396
C	-7.98962	5.706704	3.105413
H	-8.07729	5.297745	4.102798
N	-9.01886	6.317277	2.464261
H	-9.9528	6.449417	2.823695
C	-8.55991	6.701322	1.216263
H	-9.19653	7.20258	0.504469
C	4.815234	-5.59394	3.286836
C	5.599028	-4.40021	3.837171
C	3.971804	-5.34414	2.023416
O	5.733449	-4.22033	5.043658
C	3.035967	-4.17938	2.165186
C	2.47105	-3.58035	3.266502
N	2.575229	-3.46674	1.076048
C	1.762836	-2.49114	1.528448
N	1.672738	-2.52744	2.858131
H	5.529819	-6.40391	3.087603
H	4.599298	-5.19595	1.136339
H	3.407888	-6.26052	1.802269
H	2.575917	-3.83546	4.308765
H	1.27577	-1.77477	0.887266
H	2.77007	-3.75142	0.081525
N	6.161747	-3.56834	2.904116
H	5.982858	-3.73614	1.923642
C	6.963251	-2.41289	3.25891
H	7.149612	-2.48008	4.3353

C	6.337668	-1.04252	2.94995
O	7.069551	-0.08821	2.682292
N	4.987435	-0.9706	3.012619
C	4.215355	0.264174	2.816305
C	4.724472	1.048652	1.585815
C	4.152575	1.152347	4.071259
O	4.986991	2.243632	1.631894
C	2.953356	2.111657	4.074779
C	1.553491	1.480007	4.15966
O	0.549626	2.195928	4.101121
O	1.534604	0.187701	4.324594
H	4.470492	-1.80602	3.260664
H	3.201265	-0.07502	2.58627
H	4.108481	0.494192	4.945342
H	5.077444	1.733322	4.134416
H	2.98345	2.744857	3.181404
H	3.035104	2.802671	4.92446
N	4.853042	0.286389	0.457172
H	4.280254	-0.54919	0.406387
C	5.034945	0.892661	-0.86091
H	5.137863	0.041781	-1.54666
C	3.811074	1.69787	-1.31263
H	3.599676	2.491232	-0.58703
H	4.025861	2.195626	-2.26235
C	2.551859	0.826311	-1.48565
O	1.551877	1.400784	-1.99401
O	2.621334	-0.3861	-1.10267
C	6.357923	1.657668	-0.99616
O	6.546655	2.424667	-1.94666
N	7.352728	1.336706	-0.12249
H	7.119185	0.823506	0.724158
C	8.622656	2.058705	-0.14901
H	8.414503	3.113795	-0.36019
C	9.338318	1.941363	1.200123
H	9.50876	0.892839	1.467393
H	8.734044	2.394192	1.991475
H	10.30186	2.453089	1.137627
C	9.556744	1.622331	-1.29858
O	10.58114	2.25206	-1.54166
N	9.15123	0.526425	-1.99948
H	8.306143	0.064381	-1.68997
C	9.784203	0.065618	-3.21783
H	10.76657	0.549563	-3.25079
C	9.988487	-1.47037	-3.20877
H	10.77023	-1.70284	-2.47705
H	10.35831	-1.74326	-4.20121
C	8.730525	-2.24646	-2.87151

C	8.525453	-2.74866	-1.57814
H	9.303252	-2.61786	-0.82834
C	7.341109	-3.41083	-1.24398
H	7.214158	-3.80333	-0.23614
C	6.329333	-3.5784	-2.19151
H	5.395456	-4.06799	-1.92467
C	6.532008	-3.09254	-3.48751
H	5.757211	-3.22194	-4.23885
C	7.718807	-2.44022	-3.82663
H	7.869219	-2.0686	-4.83633
C	9.061838	0.518973	-4.51536
O	9.397575	0.02408	-5.59546
N	8.124015	1.483525	-4.37844
H	7.864716	1.814619	-3.45218
C	7.455097	2.103242	-5.51672
H	7.90243	1.6727	-6.41461
C	5.928297	1.931372	-5.5017
H	5.53992	2.381987	-4.57909
H	5.522286	2.524942	-6.33555
C	5.403421	0.486851	-5.6123
H	5.791067	-0.07582	-4.75082
C	3.868687	0.474825	-5.5316
H	3.479993	-0.55047	-5.54722
H	3.500243	0.954046	-4.61708
H	3.428134	1.008141	-6.38522
C	5.886744	-0.21894	-6.88957
H	6.976743	-0.32102	-6.9139
H	5.454992	-1.22516	-6.96282
H	5.575386	0.336684	-7.78556
C	-5.50073	-3.28919	-5.28815
H	-4.76073	-3.88894	-5.82801
C	-4.84431	-2.61466	-4.07279
H	-4.12812	-1.85777	-4.41395
H	-5.60468	-2.0767	-3.49419
C	-4.10706	-3.56367	-3.12149
O	-4.28003	-4.80459	-3.2448
O	-3.36232	-3.01661	-2.24205
C	-6.72292	-4.12793	-4.92862
O	-7.78548	-3.62288	-4.56363
N	-6.56911	-5.48079	-5.06905
H	-5.61425	-5.81632	-5.06963
C	-7.62009	-6.39664	-4.6681
H	-8.5725	-6.01817	-5.04647
C	-7.82041	-6.62859	-3.15933
O	-8.75994	-7.31463	-2.7739
N	-6.92307	-6.02469	-2.33216
H	-6.12448	-5.53097	-2.72734

C	-7.00972	-6.16341	-0.88531
H	-7.44253	-7.14486	-0.66844
C	-5.61383	-6.08515	-0.24204
H	-5.76105	-6.09907	0.840745
H	-5.12909	-5.14045	-0.51536
C	-4.7478	-7.28829	-0.61691
O	-4.96809	-8.40732	-0.17182
N	-3.69956	-7.00781	-1.45685
H	-3.7457	-6.18273	-2.06436
H	-3.21942	-7.82216	-1.82138
C	-7.9454	-5.14336	-0.19736
O	-8.14435	-5.22437	1.012947
N	-8.50544	-4.18286	-0.9767
H	-8.35017	-4.19665	-1.9778
C	-9.49592	-3.27515	-0.41456
H	-9.14019	-2.97034	0.574509
C	-9.76121	-2.05599	-1.30788
H	-10.1029	-2.39822	-2.29326
H	-10.6116	-1.51883	-0.86321
C	-8.59014	-1.10888	-1.48864
C	-8.11883	-0.7937	-2.77005
H	-8.56508	-1.27162	-3.63712
C	-7.05074	0.082307	-2.95583
H	-6.6856	0.318799	-3.95103
C	-6.41169	0.6526	-1.85076
O	-5.32696	1.453231	-2.08498
H	-4.89458	1.740749	-1.23371
C	-6.87834	0.364512	-0.56059
H	-6.39456	0.814942	0.30255
C	-7.95858	-0.50308	-0.39365
H	-8.30287	-0.71752	0.615815
C	3.988535	-7.36511	-2.02253
H	4.889026	-6.74758	-2.08945
C	2.779407	-6.63676	-2.60823
H	2.939868	-6.42512	-3.67477
H	1.880724	-7.26521	-2.57093
C	2.431297	-5.2975	-1.94026
O	1.315211	-4.78601	-2.25586
O	3.276393	-4.78234	-1.15158
C	2.291805	-4.66633	8.439018
C	2.028057	-3.54282	7.491642
C	1.150071	-3.40265	6.446798
N	2.722521	-2.34272	7.526348
C	2.260349	-1.55015	6.525365
N	1.307528	-2.16413	5.851668
H	2.127317	-4.37216	9.483862
H	1.618276	-5.49928	8.21948

H	0.41528	-4.10022	6.074783
H	2.622083	-0.55715	6.313424
H	3.476755	-2.1119	8.157072
C	-2.39526	9.524374	0.993997
H	-1.7015	10.37318	0.979704
C	-2.10786	8.641528	2.22981
H	-1.09545	8.222233	2.156917
H	-2.1094	9.293895	3.1141
C	-3.12586	7.510171	2.401825
H	-4.14208	7.931087	2.383672
H	-3.05933	6.851467	1.533596
C	-2.97539	6.653292	3.666018
H	-1.94892	6.307353	3.811993
H	-3.24723	7.213834	4.567805
N	-3.87205	5.488002	3.585538
H	-4.85428	5.741657	3.495115
C	-3.5594	4.418119	2.796487
N	-2.30794	4.050952	2.575284
H	-1.4999	4.443311	3.089746
H	-2.15096	3.158182	2.082871
N	-4.57758	3.720892	2.259286
H	-5.48725	4.178781	2.23531
H	-4.36066	3.037249	1.531622
C	-2.34427	8.772853	-0.33732
O	-3.09627	7.819081	-0.57681
N	-1.45381	9.256955	-1.23952
H	-0.71855	9.877733	-0.91926
C	-1.13466	8.572917	-2.48401
H	-1.78723	7.707372	-2.60279
C	0.350174	8.185944	-2.43979
O	1.164689	8.956141	-1.92184
N	0.649578	6.987705	-2.98682
H	-0.12923	6.386735	-3.26216
C	1.986378	6.409096	-2.88616
H	2.669794	7.238601	-2.68838
C	2.106433	5.338009	-1.78341
H	1.522013	4.451027	-2.05692
H	3.152858	5.012806	-1.73726
C	1.669075	5.859799	-0.41067
H	2.266491	6.731795	-0.12357
H	0.621952	6.178736	-0.43579
S	1.862597	4.66206	0.964944
C	0.57289	3.429284	0.547548
H	0.462402	2.776529	1.41688
H	-0.38323	3.928258	0.362453
H	0.858392	2.828323	-0.31901
O	-1.37117	-1.01415	5.305629

C	-1.76603	-2.2222	5.153572
O	-1.07593	-3.01876	4.454497
C	-3.0764	-2.66278	5.774028
C	-3.30552	-2.10805	7.184688
H	-3.86521	-2.29893	5.100778
H	-3.11513	-3.75647	5.756436
H	-3.26412	-1.01554	7.180432
H	-4.28514	-2.41851	7.564274
O	-0.52946	-0.79411	2.556559
O	-3.342	0.127592	3.53781
Fe	0.290886	-1.28458	3.994546
C	-2.00937	-1.26685	0.60814
H	-1.37586	-0.84895	1.625742
H	-1.16648	-1.38122	-0.08029
C	-3.02227	-0.26363	0.055216
H	-4.02913	-0.56001	0.371707
C	-2.89215	1.229272	0.476683
O	-3.73159	2.039733	-0.05535
O	-2.05323	1.528537	1.342186
H	-0.46617	-3.24915	-0.97247
N	-3.00922	-0.34996	-1.45164
H	-2.03492	-0.12417	-1.85331
H	-3.70249	0.295724	-1.85843
H	-3.23776	-1.32177	-1.77913
C	-2.65626	-2.57505	1.071011
H	-3.38901	-2.89965	0.315608
H	-3.2332	-2.33325	1.972154
C	-1.67095	-3.72126	1.381002
H	-2.04318	-4.30131	2.231371
H	-0.72524	-3.29736	1.723704
C	-1.41078	-4.72613	0.254333
H	-0.56024	-5.3623	0.516004
H	-2.27052	-5.37618	0.072504
N	-1.08547	-4.08412	-1.06184
H	-0.50809	-4.70266	-1.6585
H	-1.95957	-3.7874	-1.5652
H	3.320902	-5.03806	8.353024
H	4.182944	-5.93498	4.111487
H	4.175367	-8.30569	-2.55514
H	3.832335	-7.60178	-0.96378
H	7.927804	-2.43282	2.742916
H	7.688827	3.176822	-5.51332
H	-2.54116	-2.47057	7.882013
H	-5.85689	-2.50803	-5.96938
H	-7.44345	-7.37102	-5.13401
H	-10.4422	-3.81142	-0.24996
H	-6.05153	7.196137	-1.98548

H	-7.20406	8.116353	-1.00926
H	-3.40776	9.94037	1.082018
H	-1.29626	9.251246	-3.33267
H	2.255602	5.969376	-3.85362
H	-1.73498	1.625422	-6.39723
H	-1.43207	0.963689	-4.77501
H	-2.74394	-0.089	4.275552
H	-2.74158	0.595325	2.930319
O	-0.61584	-0.05263	-2.60314
H	-0.20019	-0.94297	-2.52358
H	0.141842	0.55308	-2.34049
O	0.061037	4.908767	3.804947
H	0.324533	3.984421	4.035347
H	0.627736	5.12087	3.035016
O	0.782268	-2.18824	-1.64541
H	1.216364	-3.00571	-2.00079
H	1.495054	-1.50017	-1.44553

[5]**IM1**$_{A,C3}$

C	-1.88626	1.964752	-4.88818
H	-2.87493	1.780951	-4.44873
C	-1.42104	3.394017	-4.59379
H	-0.49392	3.618879	-5.13208
H	-2.17187	4.108186	-4.95987
C	-1.17381	3.644354	-3.0938
H	-2.04767	3.343306	-2.50216
H	-0.32931	3.027276	-2.75898
C	-0.82286	5.102221	-2.83785
O	0.070658	5.673699	-3.47864
N	-1.55751	5.754386	-1.90498
H	-2.25125	5.27235	-1.35129
H	-1.38485	6.743473	-1.7236
C	-5.11889	8.734729	-2.17486
H	-6.02062	8.339123	-2.65731
C	-4.72937	7.908633	-0.94545
H	-4.51616	6.875435	-1.25152
H	-3.78466	8.293529	-0.54077
C	-5.77753	7.882739	0.130958
N	-5.62017	7.127663	1.287272
C	-6.71157	7.314024	2.005501
H	-6.92014	6.866357	2.967681
N	-7.57275	8.155153	1.378108
H	-8.47827	8.445147	1.717229
C	-6.99079	8.525522	0.178294
H	-7.48422	9.190239	-0.51306
C	2.680773	-6.09146	4.20553
C	4.017018	-5.34962	4.138263
C	1.591203	-5.70655	3.18368
O	4.681883	-5.17791	5.156107
C	1.296988	-4.23358	3.120265
C	0.842089	-3.33298	4.053821
N	1.497604	-3.49357	1.967532
C	1.167566	-2.21044	2.221719
N	0.768365	-2.07866	3.48638
H	2.892901	-7.16577	4.111657
H	1.874983	-6.04281	2.182655
H	0.680188	-6.25918	3.44862
H	0.558615	-3.50013	5.080891
H	1.234295	-1.4101	1.497992
H	1.907044	-3.90596	1.0973
N	4.435247	-4.90679	2.910242
H	3.90782	-5.11396	2.063956
C	5.726195	-4.2568	2.766014
H	6.366454	-4.58101	3.590971

C	5.742048	-2.71742	2.758103
O	6.783982	-2.13371	2.453242
N	4.580245	-2.09907	3.073199
C	4.354423	-0.64793	3.032669
C	5.028051	-0.02962	1.78784
C	4.72336	0.07848	4.33633
O	5.692868	0.998459	1.832109
C	4.085233	1.471927	4.464041
C	2.555678	1.55211	4.598332
O	2.013624	2.657584	4.587757
O	1.933619	0.414199	4.763556
H	3.800582	-2.68802	3.333932
H	3.275224	-0.53509	2.888001
H	4.403117	-0.55672	5.169664
H	5.812606	0.176418	4.388553
H	4.370985	2.095444	3.611654
H	4.494815	1.978588	5.348667
N	4.799276	-0.73691	0.641082
H	3.978461	-1.33238	0.626419
C	5.06244	-0.1774	-0.67903
H	4.922086	-1.01875	-1.37197
C	4.05411	0.908581	-1.06649
H	4.052012	1.694763	-0.30165
H	4.351136	1.381093	-2.0053
C	2.628701	0.34616	-1.21715
O	1.815082	1.027902	-1.8953
O	2.380807	-0.76331	-0.63792
C	6.531125	0.217511	-0.8708
O	6.869241	1.042549	-1.72557
N	7.438349	-0.53229	-0.18381
H	7.118344	-1.07182	0.618966
C	8.868095	-0.26495	-0.30017
H	9.017267	0.82049	-0.33578
C	9.621586	-0.84343	0.901304
H	9.447332	-1.92124	0.994317
H	9.283242	-0.36827	1.826698
H	10.69222	-0.66859	0.770219
C	9.481478	-0.77587	-1.62317
O	10.64031	-0.50339	-1.91925
N	8.649093	-1.51662	-2.40883
H	7.720229	-1.70372	-2.05508
C	8.971631	-1.9369	-3.75667
H	10.06208	-1.88226	-3.84277
C	8.528211	-3.4	-4.01275
H	9.117035	-4.04786	-3.35317
H	8.795552	-3.63366	-5.04675
C	7.04774	-3.6383	-3.78599

C	6.559795	-4.01849	-2.52549
H	7.263146	-4.19414	-1.71349
C	5.189018	-4.17721	-2.29826
H	4.821707	-4.46026	-1.31523
C	4.280902	-3.97154	-3.34007
H	3.215332	-4.09245	-3.16365
C	4.755019	-3.61081	-4.60428
H	4.056081	-3.45274	-5.42177
C	6.123228	-3.44162	-4.82423
H	6.486879	-3.14126	-5.80291
C	8.423857	-1.0008	-4.86651
O	8.551862	-1.33499	-6.04802
N	7.860962	0.158006	-4.45615
H	7.763812	0.351708	-3.46222
C	7.402505	1.190605	-5.37774
H	7.69047	0.868479	-6.3803
C	5.894964	1.477812	-5.28312
H	5.667609	1.778099	-4.25159
H	5.686693	2.354214	-5.91681
C	4.953848	0.328693	-5.69269
H	5.159063	-0.52434	-5.03141
C	3.487611	0.742174	-5.48589
H	2.807834	-0.07491	-5.7571
H	3.272296	1.01313	-4.44561
H	3.232015	1.605226	-6.11651
C	5.191676	-0.13379	-7.13947
H	6.197359	-0.54491	-7.27581
H	4.471716	-0.91345	-7.41912
H	5.061812	0.699693	-7.84457
C	-5.39774	-3.63787	-5.26381
H	-4.67826	-4.38374	-5.61776
C	-4.79186	-2.82922	-4.10571
H	-3.96512	-2.21463	-4.48057
H	-5.54021	-2.12931	-3.71491
C	-4.26476	-3.6625	-2.9317
O	-4.59034	-4.8773	-2.85319
O	-3.53187	-3.04763	-2.0901
C	-6.73025	-4.29218	-4.91521
O	-7.76057	-3.63881	-4.7408
N	-6.71156	-5.65832	-4.84042
H	-5.80303	-6.07655	-4.68667
C	-7.88781	-6.40028	-4.427
H	-8.74973	-6.02959	-4.98691
C	-8.28351	-6.32764	-2.94099
O	-9.33637	-6.8349	-2.5719
N	-7.42014	-5.67057	-2.11902
H	-6.53169	-5.32907	-2.48308

C	-7.70761	-5.48915	-0.70414
H	-8.27996	-6.35822	-0.36513
C	-6.40704	-5.40428	0.114527
H	-6.69318	-5.18265	1.145505
H	-5.78275	-4.58401	-0.25944
C	-5.64857	-6.73159	0.106465
O	-6.04193	-7.70569	0.734838
N	-4.491	-6.73362	-0.63179
H	-4.37152	-6.04953	-1.3873
H	-4.07923	-7.64921	-0.76899
C	-8.59826	-4.26592	-0.38339
O	-8.94977	-4.06258	0.777087
N	-8.95128	-3.46345	-1.42022
H	-8.66768	-3.70796	-2.3624
C	-9.91722	-2.3917	-1.22469
H	-9.72757	-1.95966	-0.23796
C	-9.85381	-1.32344	-2.32458
H	-10.0307	-1.79745	-3.29839
H	-10.7058	-0.64768	-2.15844
C	-8.5665	-0.52365	-2.3888
C	-7.84814	-0.42391	-3.58803
H	-8.19513	-0.96688	-4.46248
C	-6.66942	0.315797	-3.67102
H	-6.11398	0.384443	-4.60191
C	-6.1687	0.962923	-2.5373
O	-4.97658	1.626495	-2.65138
H	-4.66125	1.934871	-1.75912
C	-6.88048	0.890832	-1.33172
H	-6.50036	1.403488	-0.45183
C	-8.0665	0.158127	-1.27113
H	-8.602	0.111533	-0.32539
C	3.401165	-7.82741	-0.31177
H	4.280929	-7.27404	0.028772
C	2.571425	-6.98702	-1.2831
H	3.163431	-6.76654	-2.18282
H	1.688403	-7.53332	-1.63314
C	2.097043	-5.63143	-0.74147
O	1.097523	-5.10761	-1.31114
O	2.753915	-5.10144	0.212448
C	-0.37345	-3.32498	9.426318
C	0.086343	-2.43637	8.317672
C	-0.47997	-2.08816	7.117228
N	1.29433	-1.75456	8.341591
C	1.409122	-1.04477	7.188085
N	0.349373	-1.22477	6.425132
H	-0.48637	-2.77885	10.37207
H	-1.3468	-3.7551	9.174848

H	-1.42413	-2.40018	6.696937
H	2.255064	-0.42785	6.930005
H	1.98291	-1.78973	9.079583
C	-0.18844	9.91362	0.597548
H	0.676941	10.56945	0.750218
C	-0.27734	8.893714	1.75957
H	0.651549	8.310953	1.813438
H	-0.33968	9.462943	2.697294
C	-1.47795	7.951009	1.632339
H	-2.37814	8.543833	1.417801
H	-1.33316	7.301673	0.764834
C	-1.7656	7.061549	2.849223
H	-0.90018	6.465966	3.149419
H	-2.04875	7.663531	3.720766
N	-2.89542	6.172494	2.545866
H	-3.76113	6.659952	2.313852
C	-2.76837	5.007575	1.865092
N	-1.64522	4.291358	1.874266
H	-0.88703	4.467574	2.558705
H	-1.73251	3.324898	1.554762
N	-3.81091	4.605307	1.119235
H	-4.63937	5.1911	1.103989
H	-3.82004	3.681298	0.669531
C	-0.13136	9.252997	-0.77831
O	-1.12301	8.687221	-1.26203
N	1.059494	9.32882	-1.41517
H	1.878397	9.668366	-0.92143
C	1.382021	8.581934	-2.62239
H	0.564606	7.904155	-2.86946
C	2.699939	7.842078	-2.35649
O	3.589247	8.405663	-1.71087
N	2.775971	6.593554	-2.86411
H	1.918219	6.177903	-3.23004
C	3.906392	5.71476	-2.57425
H	4.733747	6.36027	-2.26975
C	3.601598	4.672681	-1.48087
H	2.849118	3.96366	-1.84732
H	4.514096	4.091158	-1.30337
C	3.131999	5.31118	-0.16929
H	3.891964	5.999838	0.21551
H	2.21975	5.89551	-0.33149
S	2.818731	4.126063	1.198036
C	1.33615	3.251577	0.573828
H	1.020989	2.563246	1.364327
H	0.529875	3.964298	0.373691
H	1.555858	2.672167	-0.32459
O	-1.43781	0.898296	5.312549

C	-2.26862	-0.0709	5.203892
O	-1.91478	-1.11927	4.587137
C	-3.65138	0.052717	5.809303
C	-3.62259	0.560278	7.257908
H	-4.19238	0.773316	5.183273
H	-4.14995	-0.91856	5.731733
H	-3.13495	1.537604	7.310485
H	-4.64115	0.6593	7.649368
O	-0.08632	0.565323	2.680717
O	-3.38812	2.626105	3.903241
Fe	0.12112	-0.19571	4.321196
C	-2.53565	-1.25131	0.633848
H	-1.49029	-1.03612	0.8359
C	-3.30882	-0.24702	-0.15095
H	-4.38189	-0.46538	-0.08737
C	-3.14319	1.231466	0.330398
O	-3.76614	2.118335	-0.33024
O	-2.46208	1.454056	1.360333
H	-0.84483	-3.26697	-0.44526
N	-2.9743	-0.34464	-1.62522
H	-1.93031	-0.19605	-1.85123
H	-3.53108	0.330967	-2.17087
H	-3.21381	-1.31456	-1.9471
C	-3.22125	-2.3368	1.403897
H	-3.65083	-1.89554	2.32098
C	-2.2897	-3.47727	1.859154
C	-1.98651	-4.57983	0.839991
H	-1.1985	-5.22837	1.233035
H	-2.86506	-5.19972	0.639535
N	-1.51076	-4.07838	-0.49092
H	-0.93998	-4.79178	-0.96933
H	-2.31401	-3.78849	-1.10655
H	0.321634	-4.15613	9.603253
H	2.321448	-5.93825	5.227451
H	3.74097	-8.75441	-0.78847
H	2.819277	-8.10417	0.576084
H	6.19416	-4.56999	1.82821
H	7.95155	2.116248	-5.15738
H	-3.0717	-0.1262	7.911825
H	-5.60196	-2.95407	-6.09567
H	-7.75397	-7.45444	-4.68823
H	-10.9355	-2.80734	-1.19057
H	-4.30717	8.72091	-2.90782
H	-5.30732	9.781613	-1.90747
H	-1.08516	10.54485	0.607902
H	1.529793	9.270068	-3.46622
H	4.197951	5.199847	-3.49696

H	-1.96895	1.800066	-5.96959
H	-1.19294	1.22069	-4.48014
H	-2.60913	2.386014	4.43795
H	-3.19938	2.199927	3.047524
O	-0.47651	-0.24918	-2.52538
H	-0.17233	-1.16686	-2.34121
H	0.313454	0.303106	-2.24961
O	0.563977	4.760916	3.545764
H	0.846661	3.974916	4.063865
H	1.313219	4.87043	2.924011
O	0.473257	-2.44304	-1.20151
H	0.930982	-3.30376	-1.36497
H	1.181045	-1.76784	-0.95513
H	-0.95746	0.742534	2.270139
H	-4.07379	-2.73186	0.831345
H	-1.35826	-3.04001	2.228058
H	-2.74281	-3.983	2.720024

[5]**TS2**$_{A,C3}$

C	0.120011	2.519814	-5.16298
H	-0.72463	2.671304	-5.84938
C	0.224922	3.671753	-4.15796
H	1.0882	3.49811	-3.503
H	0.397033	4.618528	-4.68165
C	-1.03499	3.82098	-3.29519
H	-1.93112	3.775041	-3.933
H	-1.12223	2.992539	-2.58487
C	-1.11152	5.152645	-2.55457
O	-0.64309	6.192314	-3.05149
N	-1.75589	5.147757	-1.36979
H	-2.09767	4.28298	-0.95049
H	-1.91105	6.035233	-0.89439
C	-5.83865	5.565504	-0.71982
H	-6.50128	4.72223	-0.94698
C	-5.32844	5.506033	0.724484
H	-4.72696	4.599011	0.865013
H	-4.6417	6.346133	0.894175
C	-6.42009	5.525533	1.758827
N	-6.17773	5.236758	3.099347
C	-7.33895	5.341795	3.716961
H	-7.51358	5.176292	4.771506
N	-8.3275	5.687088	2.852228
H	-9.30327	5.813099	3.077659
C	-7.75612	5.804171	1.597518
H	-8.33929	6.061858	0.727624
C	3.227083	-6.73187	3.219693
C	4.371704	-5.75225	3.489914
C	2.197182	-6.37006	2.12841
O	4.855156	-5.66628	4.61589
C	1.590642	-5.0043	2.285286
C	0.796985	-4.43365	3.254696
N	1.833349	-3.99292	1.373671
C	1.205848	-2.87866	1.804264
N	0.566045	-3.11127	2.947209
H	3.679147	-7.70329	2.973555
H	2.658268	-6.42926	1.138272
H	1.410628	-7.1354	2.150805
H	0.378863	-4.87926	4.144934
H	1.227895	-1.93893	1.271129
H	2.453587	-4.11641	0.536221
N	4.839745	-5.01085	2.436131
H	4.472145	-5.13169	1.492357
C	5.99155	-4.14185	2.605468
H	6.541567	-4.47787	3.488772

C	5.718634	-2.6372	2.77959
O	6.659717	-1.84498	2.703859
N	4.430631	-2.28082	2.993184
C	3.922942	-0.90424	3.052074
C	4.637296	-0.00971	2.018111
C	3.906307	-0.28937	4.460453
O	5.079465	1.099518	2.291664
C	2.956653	0.917742	4.57429
C	1.472073	0.647324	4.258513
O	0.743902	1.594087	3.896809
O	1.076464	-0.56377	4.420694
H	3.751981	-3.02928	3.04197
H	2.881839	-0.98127	2.722287
H	3.595832	-1.07519	5.156951
H	4.92172	0.01972	4.728921
H	3.304215	1.729381	3.93101
H	2.993543	1.304418	5.603353
N	4.707214	-0.56804	0.771185
H	4.008704	-1.27201	0.558809
C	5.019561	0.239042	-0.40278
H	5.096746	-0.48011	-1.23
C	3.903513	1.233112	-0.74466
H	3.731257	1.890593	0.115758
H	4.215047	1.87723	-1.56925
C	2.585138	0.539472	-1.13042
O	1.810059	1.198526	-1.8757
O	2.365672	-0.63249	-0.67544
C	6.414863	0.874814	-0.32358
O	6.712002	1.85413	-1.01445
N	7.339151	0.196822	0.413424
H	7.012882	-0.50214	1.079033
C	8.682372	0.737856	0.605265
H	8.603074	1.825172	0.721908
C	9.327663	0.138359	1.858288
H	9.369005	-0.95474	1.796136
H	8.747196	0.401234	2.747236
H	10.34462	0.525924	1.955656
C	9.604672	0.554088	-0.62027
O	10.72167	1.061107	-0.64273
N	9.0805	-0.18028	-1.64112
H	8.155235	-0.56572	-1.5061
C	9.69933	-0.32157	-2.9424
H	10.76092	-0.08874	-2.80786
C	9.570261	-1.77503	-3.46841
H	10.16035	-2.42333	-2.81042
H	10.02428	-1.79264	-4.46267
C	8.137093	-2.26491	-3.53057

C	7.535785	-2.87829	-2.41989
H	8.129576	-3.06155	-1.52638
C	6.188382	-3.25045	-2.44078
H	5.731863	-3.70761	-1.56678
C	5.421092	-3.03328	-3.5888
H	4.373191	-3.32104	-3.60196
C	6.015664	-2.44881	-4.71045
H	5.429429	-2.28216	-5.61069
C	7.35768	-2.06411	-4.68073
H	7.809131	-1.58644	-5.5459
C	9.185277	0.686206	-4.00444
O	9.567625	0.572769	-5.17288
N	8.359708	1.660969	-3.56105
H	8.050627	1.663794	-2.59179
C	7.868244	2.735636	-4.41449
H	8.36052	2.617973	-5.38184
C	6.337114	2.755825	-4.55083
H	5.904629	2.865093	-3.54749
H	6.065723	3.664839	-5.11007
C	5.703515	1.532123	-5.23936
H	5.97301	0.643121	-4.65303
C	4.171236	1.651948	-5.2326
H	3.705815	0.773754	-5.69676
H	3.767099	1.738945	-4.21711
H	3.845081	2.535817	-5.79866
C	6.230456	1.326734	-6.66861
H	7.305812	1.120523	-6.67943
H	5.724244	0.480436	-7.15049
H	6.043729	2.215722	-7.28789
C	-5.1908	-2.32913	-6.00148
H	-4.52515	-3.03654	-6.50634
C	-4.5274	-1.79987	-4.72127
H	-3.66675	-1.17215	-4.98137
H	-5.22939	-1.14998	-4.18557
C	-4.0423	-2.8811	-3.75155
O	-4.36951	-4.07739	-3.95322
O	-3.32552	-2.47646	-2.77122
C	-6.55842	-2.95118	-5.74312
O	-7.52397	-2.28488	-5.36666
N	-6.65108	-4.29554	-5.97611
H	-5.77889	-4.80686	-6.00194
C	-7.87163	-5.02256	-5.67889
H	-8.71759	-4.47856	-6.10568
C	-8.21362	-5.24572	-4.19418
O	-9.29344	-5.73797	-3.89062
N	-7.26875	-4.85664	-3.29438
H	-6.37151	-4.50802	-3.62413

C	-7.48337	-4.98173	-1.86061
H	-8.12103	-5.8552	-1.6942
C	-6.14899	-5.19481	-1.12344
H	-6.3736	-5.20848	-0.05406
H	-5.47511	-4.35416	-1.32759
C	-5.50409	-6.53012	-1.49319
O	-5.96403	-7.59886	-1.11661
N	-4.35983	-6.43164	-2.25043
H	-4.21651	-5.59716	-2.828
H	-4.03434	-7.3132	-2.63018
C	-8.23457	-3.79504	-1.21401
O	-8.52106	-3.84354	-0.01956
N	-8.53729	-2.7343	-2.00604
H	-8.33443	-2.77162	-2.99883
C	-9.37605	-1.66106	-1.48667
H	-9.03538	-1.43875	-0.47086
C	-9.35633	-0.40334	-2.36559
H	-9.66338	-0.67041	-3.38487
H	-10.1428	0.260967	-1.97856
C	-8.03723	0.344426	-2.42268
C	-7.42471	0.616476	-3.65368
H	-7.87761	0.244205	-4.56787
C	-6.21965	1.312671	-3.72806
H	-5.74629	1.515839	-4.6843
C	-5.58515	1.743175	-2.55827
O	-4.37004	2.357279	-2.68561
H	-3.96652	2.546672	-1.79745
C	-6.1921	1.502313	-1.31679
H	-5.71324	1.851674	-0.40524
C	-7.40596	0.814166	-1.26281
H	-7.85881	0.633886	-0.29017
C	4.734966	-7.33239	-1.27656
H	5.429683	-6.79156	-0.62735
C	4.02655	-6.3756	-2.23687
H	4.765044	-5.90658	-2.90167
H	3.33002	-6.91044	-2.89196
C	3.249341	-5.22837	-1.57193
O	2.311302	-4.72168	-2.24006
O	3.622878	-4.85053	-0.40956
C	-0.16029	-5.27738	8.685546
C	0.017548	-4.17117	7.69826
C	-0.68485	-3.81351	6.574987
N	1.039625	-3.23629	7.78166
C	0.922181	-2.3786	6.73335
N	-0.11399	-2.70148	5.985881
H	-0.32994	-4.89998	9.702698
H	-1.02827	-5.88223	8.408669

H	-1.55793	-4.27795	6.140842
H	1.590292	-1.55583	6.532666
H	1.769901	-3.2147	8.47886
C	-1.96642	9.360278	1.89416
H	-1.40731	10.28973	2.055224
C	-1.62313	8.349716	3.01003
H	-0.5658	8.061811	2.934381
H	-1.73171	8.868942	3.972834
C	-2.50925	7.099039	2.996687
H	-3.56465	7.405909	2.967142
H	-2.33088	6.533079	2.079121
C	-2.30395	6.176908	4.204172
H	-1.26755	5.839827	4.288558
H	-2.53281	6.704672	5.138299
N	-3.18692	5.00716	4.144328
H	-4.18307	5.223252	4.136049
C	-2.89805	3.886698	3.429725
N	-1.6553	3.554405	3.116964
H	-0.81769	3.967729	3.558687
H	-1.50228	2.786809	2.45927
N	-3.92127	3.104664	3.045131
H	-4.85947	3.46239	3.182111
H	-3.78115	2.092263	2.881471
C	-1.71655	8.825251	0.485684
O	-2.33509	7.849252	0.045579
N	-0.80369	9.523186	-0.23955
H	-0.16963	10.14171	0.253753
C	-0.28323	9.063461	-1.51764
H	-0.86521	8.209946	-1.86629
C	1.20418	8.723814	-1.33886
O	1.905762	9.418484	-0.59753
N	1.629969	7.652431	-2.04119
H	0.921931	7.08767	-2.51707
C	2.979012	7.115567	-1.90601
H	3.592953	7.907129	-1.46818
C	3.036654	5.841553	-1.0421
H	2.43258	5.056697	-1.51463
H	4.070272	5.474702	-1.0294
C	2.557331	6.07355	0.394014
H	3.174794	6.833646	0.885017
H	1.525121	6.438725	0.405071
S	2.658606	4.593042	1.474329
C	1.299752	3.561477	0.80824
H	1.143493	2.753924	1.527234
H	0.375685	4.141367	0.730874
H	1.546214	3.126568	-0.16295
O	-2.53422	-1.17651	4.928643

C	-3.1076	-2.29182	4.644686
O	-2.49037	-3.20501	4.041812
C	-4.57253	-2.46523	5.025554
C	-4.90065	-1.97386	6.440431
H	-5.15677	-1.89195	4.291396
H	-4.83936	-3.51835	4.894418
H	-4.62413	-0.92221	6.557896
H	-5.9709	-2.08058	6.651108
O	-1.20259	-0.72911	2.288929
O	-3.66623	0.310348	2.867922
Fe	-0.62986	-1.66137	4.0541
C	-2.04828	-1.11997	0.106743
H	-1.01389	-1.2647	-0.19395
C	-2.72277	0.115418	-0.40989
H	-3.79218	0.069715	-0.18153
C	-2.23514	1.547596	0.075051
O	-2.83213	2.489999	-0.52234
O	-1.38039	1.62684	0.968422
H	-0.52006	-3.40132	-1.40318
N	-2.58397	0.066412	-1.91155
H	-1.53652	0.024834	-2.2426
H	-3.04336	0.894376	-2.32401
H	-3.02008	-0.80112	-2.30778
C	-2.89158	-2.2845	0.485694
H	-3.52713	-1.96034	1.321122
C	-2.12653	-3.56656	0.853077
C	-1.93778	-4.59757	-0.26016
H	-1.23482	-5.36531	0.074014
H	-2.87261	-5.09154	-0.53732
N	-1.38409	-4.0136	-1.53252
H	-1.09201	-4.7626	-2.16627
H	-2.12859	-3.45941	-2.03918
H	0.711673	-5.94371	8.719683
H	2.730138	-6.85982	4.185809
H	5.301687	-8.09199	-1.82826
H	4.018461	-7.85644	-0.63179
H	6.653201	-4.22841	1.738381
H	8.197747	3.693578	-3.98935
H	-4.34941	-2.54756	7.194797
H	-5.35822	-1.48635	-6.68189
H	-7.8299	-6.00159	-6.16563
H	-10.4134	-2.01477	-1.39282
H	-4.99536	5.528477	-1.41594
H	-6.38806	6.494786	-0.91513
H	-3.03317	9.612039	1.955138
H	-0.37043	9.86754	-2.26066
H	3.372068	6.894206	-2.90568

H	1.031832	2.448161	-5.76736
H	-0.0172	1.557233	-4.65835
H	-3.58711	0.051601	3.808402
H	-2.77438	0.001384	2.558949
O	-0.16329	-0.20284	-2.85229
H	0.172606	-1.12033	-2.74517
H	0.578309	0.373829	-2.45879
O	0.777716	4.314631	4.266342
H	0.93056	3.336263	4.260672
H	1.378294	4.645746	3.568436
O	0.851554	-2.53999	-1.70421
H	1.501487	-3.27578	-1.91436
H	1.385953	-1.80212	-1.2742
H	-0.82972	0.153216	2.084414
H	-3.59334	-2.45439	-0.35078
H	-1.16008	-3.28403	1.267194
H	-2.64751	-4.07035	1.67195

^{5}**Pr**$_{A,C3}$

C	-1.44014	1.751338	-5.19461
H	-2.50681	1.539206	-5.03709
C	-1.07601	3.138905	-4.65562
H	-0.04326	3.389701	-4.92081
H	-1.70476	3.902124	-5.1321
C	-1.22933	3.232677	-3.13165
H	-2.21628	2.867478	-2.81938
H	-0.48267	2.586961	-2.64638
C	-1.03917	4.649653	-2.60478
O	-0.36906	5.491582	-3.22228
N	-1.65081	4.93691	-1.43236
H	-2.13276	4.216863	-0.90035
H	-1.54509	5.872284	-1.04593
C	-5.25596	7.668238	-1.34571
H	-6.11504	7.172402	-1.81292
C	-4.85793	6.989965	-0.03158
H	-4.57938	5.946746	-0.23433
H	-3.94779	7.466259	0.351961
C	-5.93009	7.009373	1.020446
N	-5.75258	6.402527	2.259669
C	-6.87774	6.581584	2.926421
H	-7.08554	6.226586	3.926629
N	-7.77795	7.28013	2.189456
H	-8.71394	7.532057	2.471377
C	-7.18828	7.558276	0.968871
H	-7.70847	8.099725	0.194684
C	3.24824	-6.54206	3.903383
C	4.38106	-5.52466	4.060555
C	2.206138	-6.304	2.789158
O	4.853431	-5.29405	5.171618
C	1.593349	-4.93199	2.797348
C	0.775673	-4.26654	3.683661
N	1.855025	-4.02029	1.791583
C	1.209297	-2.87335	2.08537
N	0.542999	-2.98552	3.231086
H	3.709225	-7.52869	3.752813
H	2.659042	-6.46906	1.807149
H	1.422821	-7.06446	2.901411
H	0.342123	-4.61778	4.609005
H	1.231704	-2.00197	1.444633
H	2.481169	-4.23398	0.973726
N	4.847658	-4.91282	2.927463
H	4.481507	-5.13931	2.001126
C	5.971163	-3.99586	2.996948
H	6.526611	-4.2094	3.914636

C	5.647038	-2.49141	2.994815
O	6.551977	-1.68089	2.785272
N	4.354981	-2.15534	3.220761
C	3.800411	-0.79832	3.144194
C	4.443372	-0.00996	1.986876
C	3.817547	-0.03426	4.477722
O	4.874515	1.129823	2.117064
C	2.892985	1.197544	4.495709
C	1.38694	0.96931	4.258001
O	0.658952	1.956784	4.039555
O	0.971058	-0.24764	4.317139
H	3.705025	-2.91508	3.373804
H	2.749457	-0.9391	2.877539
H	3.516532	-0.74188	5.258657
H	4.842337	0.28546	4.69424
H	3.242561	1.928304	3.761279
H	2.97854	1.693754	5.473319
N	4.466212	-0.6934	0.80243
H	3.81241	-1.46067	0.699441
C	4.720499	-0.01492	-0.46286
H	4.811898	-0.81817	-1.20667
C	3.568514	0.900617	-0.88008
H	3.421637	1.656864	-0.10148
H	3.839972	1.446007	-1.7863
C	2.241263	0.167442	-1.13786
O	1.402784	0.811887	-1.8327
O	2.072398	-0.99519	-0.65156
C	6.081783	0.696985	-0.48186
O	6.294646	1.651309	-1.23683
N	7.074151	0.115514	0.24916
H	6.81005	-0.53673	0.98628
C	8.385221	0.753686	0.35283
H	8.240079	1.835319	0.458429
C	9.143077	0.221412	1.572989
H	9.274635	-0.86479	1.5117
H	8.590298	0.44095	2.490786
H	10.12773	0.693384	1.614861
C	9.24642	0.605804	-0.92119
O	10.30486	1.216129	-1.03278
N	8.742009	-0.22804	-1.87241
H	7.869792	-0.69525	-1.66267
C	9.305055	-0.39659	-3.19565
H	10.34646	-0.06277	-3.13665
C	9.279501	-1.88761	-3.6232
H	9.966262	-2.43668	-2.96866
H	9.669895	-1.93178	-4.64329
C	7.894439	-2.50025	-3.55451

C	7.41975	-3.07566	-2.36501
H	8.083788	-3.14547	-1.50557
C	6.108507	-3.54946	-2.26408
H	5.748428	-3.96694	-1.32736
C	5.251779	-3.47856	-3.36646
H	4.233044	-3.84732	-3.28307
C	5.722533	-2.93393	-4.56458
H	5.06677	-2.88031	-5.4301
C	7.027136	-2.44428	-4.65669
H	7.379238	-1.99668	-5.58212
C	8.64828	0.489844	-4.28729
O	8.980297	0.334192	-5.46606
N	7.761738	1.416302	-3.85805
H	7.510011	1.458954	-2.87305
C	7.150739	2.400768	-4.7421
H	7.602576	2.262009	-5.72628
C	5.616941	2.308677	-4.80106
H	5.225282	2.447667	-3.78448
H	5.257994	3.163968	-5.39535
C	5.03424	1.009306	-5.38908
H	5.381844	0.175085	-4.76452
C	3.498469	1.038235	-5.32465
H	3.072125	0.105505	-5.71394
H	3.126674	1.167183	-4.30113
H	3.096897	1.86184	-5.93175
C	5.516423	0.750306	-6.82524
H	6.601556	0.611518	-6.87085
H	5.047073	-0.15384	-7.23342
H	5.247953	1.586326	-7.48688
C	-5.36213	-1.75584	-5.65927
H	-4.66796	-2.28172	-6.3218
C	-4.68414	-1.47156	-4.30927
H	-3.83942	-0.78597	-4.45832
H	-5.38314	-0.96017	-3.6392
C	-4.14801	-2.70747	-3.58422
O	-4.24228	-3.8311	-4.14789
O	-3.63671	-2.51732	-2.43039
C	-6.6681	-2.53149	-5.50663
O	-7.68954	-2.02026	-5.04948
N	-6.62965	-3.83539	-5.92702
H	-5.70664	-4.23967	-6.02282
C	-7.7558	-4.72338	-5.71228
H	-8.67294	-4.15742	-5.89009
C	-7.90001	-5.35983	-4.31691
O	-8.86331	-6.07566	-4.07286
N	-6.9199	-5.06493	-3.41494
H	-6.10272	-4.54073	-3.72053

C	-6.91566	-5.64134	-2.07745
H	-7.39728	-6.62237	-2.1291
C	-5.47579	-5.81074	-1.55486
H	-5.56134	-6.13811	-0.51487
H	-4.96802	-4.83993	-1.57502
C	-4.6815	-6.87771	-2.30943
O	-4.93955	-8.06706	-2.2119
N	-3.6214	-6.3982	-3.06282
H	-3.73873	-5.46178	-3.47609
H	-3.23281	-7.10091	-3.68443
C	-7.71462	-4.84057	-1.02234
O	-7.84637	-5.30005	0.110297
N	-8.216	-3.63525	-1.38888
H	-8.10799	-3.31451	-2.34269
C	-9.01522	-2.8679	-0.43997
H	-8.4495	-2.78259	0.494791
C	-9.40244	-1.47827	-0.96591
H	-9.95068	-1.58971	-1.91047
H	-10.1209	-1.0622	-0.24569
C	-8.2517	-0.50919	-1.16271
C	-7.93889	-0.01551	-2.43602
H	-8.50413	-0.34872	-3.30131
C	-6.87752	0.867042	-2.62867
H	-6.63605	1.240878	-3.61926
C	-6.08614	1.265632	-1.54622
O	-5.01069	2.066246	-1.82009
H	-4.47643	2.26534	-1.00838
C	-6.40043	0.808828	-0.25704
H	-5.81473	1.125346	0.603503
C	-7.47475	-0.06609	-0.08254
H	-7.70036	-0.41378	0.923686
C	4.938896	-7.51567	-0.45408
H	5.616208	-6.86878	0.111369
C	4.18571	-6.72264	-1.5228
H	4.897956	-6.31502	-2.25337
H	3.511098	-7.36932	-2.09509
C	3.354067	-5.53331	-1.01042
O	2.442007	-5.12294	-1.76703
O	3.667084	-5.03552	0.127415
C	-0.52703	-4.29483	9.25166
C	-0.25224	-3.35319	8.125651
C	-0.90919	-3.10722	6.945952
N	0.833227	-2.48939	8.106165
C	0.793811	-1.77924	6.94679
N	-0.25091	-2.13033	6.224865
H	-0.68845	-3.7676	10.20128
H	-1.43066	-4.8719	9.036608

H	-1.80726	-3.56326	6.5564
H	1.525913	-1.04141	6.658152
H	1.549213	-2.41827	8.814757
C	-0.64246	9.565842	1.140795
H	0.177452	10.29279	1.174969
C	-0.57778	8.655424	2.388266
H	0.347318	8.064136	2.366024
H	-0.50852	9.304163	3.272477
C	-1.78448	7.719901	2.505637
H	-2.71109	8.310222	2.450552
H	-1.79259	7.065501	1.632031
C	-1.834	6.843779	3.765792
H	-0.87732	6.355605	3.968642
H	-2.08038	7.43056	4.657709
N	-2.87714	5.815246	3.61376
H	-3.81516	6.192181	3.484753
C	-2.66651	4.708677	2.853766
N	-1.46545	4.20531	2.652301
H	-0.61866	4.50743	3.172437
H	-1.38617	3.310893	2.144888
N	-3.76049	4.090899	2.325263
H	-4.58611	4.687495	2.24614
H	-3.57865	3.489332	1.512853
C	-0.62912	8.811994	-0.19089
O	-1.47768	7.951474	-0.46181
N	0.341795	9.190573	-1.05825
H	1.130634	9.726649	-0.71345
C	0.620216	8.487896	-2.3031
H	-0.11685	7.697971	-2.45059
C	2.054138	7.945151	-2.21854
O	2.930458	8.627233	-1.67883
N	2.236991	6.717488	-2.75048
H	1.40482	6.2004	-3.04066
C	3.48479	5.978266	-2.57656
H	4.261028	6.713742	-2.35113
C	3.391103	4.918156	-1.46258
H	2.635156	4.174079	-1.74294
H	4.345667	4.381741	-1.39888
C	3.045116	5.524186	-0.09817
H	3.833809	6.209469	0.230206
H	2.117139	6.102094	-0.15514
S	2.873285	4.292084	1.250144
C	1.344749	3.426838	0.738056
H	1.072248	2.763674	1.561602
H	0.53164	4.139858	0.573931
H	1.487502	2.824223	-0.16156
O	-2.70489	-0.42319	4.67893

C	-3.20207	-1.58701	4.613972
O	-2.47662	-2.58657	4.292301
C	-4.67955	-1.8298	4.895413
C	-5.39693	-0.67942	5.603704
H	-5.15872	-2.03539	3.927646
H	-4.75321	-2.76495	5.463452
H	-5.37828	0.21869	4.981352
H	-6.44242	-0.94485	5.80093
O	-1.30702	-0.70418	2.017005
O	-4.29874	1.116702	2.872865
Fe	-0.66999	-1.42754	4.124734
C	-1.81692	-1.04458	0.72608
H	-0.97385	-1.15065	0.028058
C	-2.74895	0.075608	0.181641
H	-3.74531	-0.03996	0.618211
C	-2.35086	1.542275	0.492475
O	-3.0818	2.451956	-0.02168
O	-1.3861	1.756115	1.258918
H	-0.83626	-3.91377	-1.52616
N	-2.87456	-0.08558	-1.30822
H	-1.91984	-0.19548	-1.80025
H	-3.39096	0.710103	-1.70775
H	-3.37373	-0.95703	-1.60347
C	-2.60289	-2.36158	0.821451
H	-3.25945	-2.28341	1.693658
C	-1.73236	-3.63154	0.93649
C	-2.0982	-4.7484	-0.0336
H	-1.50283	-5.64199	0.172941
H	-3.15558	-5.02169	0.015357
N	-1.80205	-4.346	-1.45134
H	-1.89494	-5.16212	-2.06876
H	-2.50358	-3.64418	-1.81462
H	0.294049	-5.00789	9.40291
H	2.76036	-6.57519	4.881934
H	5.532182	-8.31853	-0.90849
H	4.249024	-7.97801	0.262682
H	6.639201	-4.16088	2.146654
H	7.429648	3.403259	-4.38914
H	-4.91707	-0.44321	6.559771
H	-5.61972	-0.8007	-6.12991
H	-7.7226	-5.53902	-6.44144
H	-9.92577	-3.4324	-0.19719
H	-4.41888	7.631868	-2.04845
H	-5.51421	8.722771	-1.18853
H	-1.57801	10.14099	1.168102
H	0.556114	9.188487	-3.14648
H	3.742203	5.490393	-3.52385

H	-1.24791	1.686789	-6.27237
H	-0.87117	0.959886	-4.69479
H	-3.67127	0.653409	3.470387
H	-4.18947	2.057932	3.08926
O	-0.62398	-0.59691	-2.6517
H	-0.30381	-1.51995	-2.62139
H	0.172656	-0.04925	-2.33392
O	0.918625	4.675676	3.993792
H	0.961699	3.695336	4.148229
H	1.596747	4.834475	3.306469
O	0.640815	-3.08359	-1.676
H	1.360427	-3.77277	-1.73601
H	1.079311	-2.29575	-1.25258
H	-1.07819	0.260915	1.967837
H	-3.26087	-2.44042	-0.05073
H	-0.68376	-3.38765	0.758887
H	-1.78539	-4.02762	1.953822

[5]**TS1**$_{A,C4}$

C	-3.09533	0.249817	-5.28247
H	-3.99417	-0.34169	-5.06033
C	-3.3325	1.73854	-5.00911
H	-2.45791	2.32378	-5.31174
H	-4.17313	2.106381	-5.61354
C	-3.60644	2.013852	-3.5252
H	-4.49098	1.463556	-3.18188
H	-2.75733	1.635393	-2.93912
C	-3.75499	3.496263	-3.20751
O	-3.09584	4.355235	-3.81219
N	-4.62942	3.813813	-2.22759
H	-5.13551	3.089606	-1.7269
H	-4.69491	4.781309	-1.91427
C	-8.25142	4.752905	-0.79901
H	-9.00081	3.952837	-0.78454
C	-7.39126	4.738854	0.468846
H	-6.85731	3.781004	0.529206
H	-6.617	5.512469	0.384644
C	-8.17351	4.936032	1.736376
N	-7.57308	4.871152	2.989381
C	-8.53457	5.063837	3.873065
H	-8.41562	5.067898	4.948034
N	-9.73086	5.257021	3.261882
H	-10.6179	5.409944	3.718812
C	-9.51666	5.175545	1.897141
H	-10.3205	5.284479	1.186419
C	7.038538	-4.14104	3.01367
C	7.663711	-2.74773	3.130069
C	5.81014	-4.28189	2.096096
O	8.167231	-2.36004	4.179672
C	4.737964	-3.27968	2.406308
C	4.469617	-2.54237	3.535963
N	3.798422	-2.89006	1.473825
C	3.004105	-1.96395	2.047754
N	3.381097	-1.72415	3.303029
H	7.822311	-4.8323	2.676187
H	6.090154	-4.19744	1.038287
H	5.420558	-5.30413	2.191412
H	4.972263	-2.54948	4.490517
H	2.192843	-1.46942	1.536896
H	3.658226	-3.38639	0.553118
N	7.654203	-1.98089	1.993881
H	7.192295	-2.33106	1.166117
C	8.235842	-0.65404	1.936604
H	8.721584	-0.48736	2.903282

C	7.252561	0.495693	1.662399
O	7.634422	1.475215	1.018643
N	6.005353	0.365476	2.171115
C	4.949147	1.382219	2.044471
C	4.895023	1.942952	0.604707
C	5.043023	2.51023	3.087588
O	4.9293	3.142118	0.362707
C	3.694993	3.203511	3.34225
C	2.594569	2.347	3.993104
O	1.438427	2.763503	4.065346
O	3.007319	1.201438	4.473329
H	5.778839	-0.47363	2.692797
H	4.017716	0.8347	2.215412
H	5.420328	2.076687	4.018995
H	5.769919	3.248668	2.737091
H	3.294386	3.613484	2.409008
H	3.846665	4.069541	4.000993
N	4.814265	0.979734	-0.36558
H	4.421227	0.086999	-0.08928
C	4.497154	1.321161	-1.75134
H	4.504617	0.362007	-2.28456
C	3.099658	1.927603	-1.91209
H	2.996962	2.789754	-1.2451
H	2.978157	2.304927	-2.93035
C	1.964703	0.93237	-1.62125
O	0.811375	1.317936	-1.9701
O	2.261606	-0.16981	-1.06731
C	5.588413	2.151812	-2.44304
O	5.359138	2.698332	-3.52795
N	6.836895	2.131708	-1.90117
H	6.956614	1.825314	-0.93802
C	7.906137	2.931546	-2.49594
H	7.489881	3.900571	-2.79398
C	9.040128	3.154461	-1.49059
H	9.449165	2.201918	-1.13696
H	8.675065	3.704649	-0.61875
H	9.836491	3.724459	-1.97553
C	8.460702	2.341252	-3.81054
O	9.219381	3.000649	-4.51365
N	8.048904	1.077677	-4.11372
H	7.44416	0.611894	-3.44996
C	8.321983	0.42651	-5.37909
H	9.138113	0.994514	-5.83915
C	8.791522	-1.03605	-5.18208
H	9.790788	-1.01102	-4.73313
H	8.877547	-1.47317	-6.18087
C	7.867004	-1.86718	-4.3141

C	8.173456	-2.10668	-2.96654
H	9.104782	-1.72	-2.55733
C	7.305679	-2.83812	-2.15069
H	7.580843	-3.02818	-1.11409
C	6.104887	-3.33752	-2.65893
H	5.415651	-3.88914	-2.02366
C	5.795922	-3.10951	-4.00393
H	4.867996	-3.49735	-4.41627
C	6.66671	-2.39021	-4.82405
H	6.421627	-2.22609	-5.86958
C	7.153002	0.500214	-6.39779
O	7.205081	-0.18575	-7.42318
N	6.156534	1.367773	-6.11168
H	6.160662	1.876953	-5.23087
C	5.066102	1.655213	-7.03713
H	5.275899	1.096916	-7.95139
C	3.674774	1.32567	-6.47364
H	3.51968	1.92437	-5.56655
H	2.932082	1.673323	-7.2084
C	3.402816	-0.15489	-6.146
H	4.12416	-0.46318	-5.37541
C	1.988925	-0.31499	-5.56417
H	1.80372	-1.35072	-5.25483
H	1.824309	0.326389	-4.69051
H	1.228763	-0.05301	-6.31309
C	3.597448	-1.07306	-7.36339
H	4.629455	-1.05473	-7.72924
H	3.350495	-2.11148	-7.10815
H	2.936434	-0.77328	-8.18897
C	-5.17632	-4.07442	-4.4097
H	-4.25579	-4.44791	-4.86995
C	-4.84006	-3.30525	-3.11594
H	-4.3342	-2.36738	-3.37185
H	-5.76684	-3.02048	-2.60035
C	-3.95661	-4.05895	-2.11381
O	-3.79107	-5.3025	-2.24767
O	-3.45019	-3.37984	-1.16262
C	-6.17432	-5.20771	-4.19429
O	-7.38379	-4.99941	-4.06236
N	-5.64428	-6.46718	-4.14933
H	-4.65336	-6.49917	-3.93542
C	-6.46548	-7.59302	-3.73305
H	-7.36361	-7.63312	-4.353
C	-6.96253	-7.57435	-2.27122
O	-7.87138	-8.31708	-1.92176
N	-6.33732	-6.6806	-1.45599
H	-5.53961	-6.16042	-1.81574

C	-6.76314	-6.4161	-0.09091
H	-7.18136	-7.34359	0.314182
C	-5.56717	-5.98424	0.780002
H	-5.95971	-5.75877	1.773844
H	-5.11634	-5.07052	0.374399
C	-4.53236	-7.10146	0.915348
O	-4.71571	-8.06809	1.643387
N	-3.38187	-6.9261	0.183492
H	-3.39782	-6.32707	-0.64703
H	-2.78917	-7.74668	0.137952
C	-7.89299	-5.35848	0.02353
O	-8.27119	-4.99109	1.136622
N	-8.41212	-4.89461	-1.14057
H	-8.05101	-5.23619	-2.02537
C	-9.5977	-4.05145	-1.19245
H	-9.81455	-3.73908	-0.16851
C	-9.44834	-2.85676	-2.14207
H	-9.13774	-3.23416	-3.12552
H	-10.4527	-2.43081	-2.28589
C	-8.49962	-1.74661	-1.71955
C	-8.25288	-0.69211	-2.61476
H	-8.72911	-0.70558	-3.5935
C	-7.41444	0.370208	-2.28917
H	-7.23361	1.178258	-2.99251
C	-6.78514	0.394675	-1.03999
O	-5.93297	1.437848	-0.75978
H	-5.46952	1.269618	0.100138
C	-7.01889	-0.63986	-0.12866
H	-6.53335	-0.61816	0.842446
C	-7.868	-1.69711	-0.47064
H	-8.02204	-2.48941	0.256597
C	4.085081	-7.35738	-1.1336
H	4.82244	-6.80353	-1.72198
C	2.704654	-6.71064	-1.23945
H	2.347468	-6.73475	-2.2787
H	1.958595	-7.27307	-0.66345
C	2.63326	-5.24729	-0.77881
O	1.474528	-4.74132	-0.67044
O	3.714812	-4.63451	-0.55408
C	6.004901	-2.59843	8.673966
C	5.244923	-1.74289	7.714997
C	4.12842	-1.97653	6.95319
N	5.606623	-0.43766	7.412684
C	4.727218	0.052543	6.502096
N	3.820293	-0.85709	6.202756
H	6.044774	-2.15632	9.678059
H	5.519499	-3.57429	8.761198

H	3.517747	-2.86418	6.887761
H	4.761971	1.046779	6.085756
H	6.406076	0.05665	7.782082
C	-4.22301	8.707626	0.18307
H	-3.80613	9.698128	-0.03433
C	-3.52152	8.115204	1.427304
H	-2.45716	7.952978	1.211135
H	-3.5629	8.872613	2.222583
C	-4.16945	6.817508	1.919758
H	-5.25804	6.963768	1.962732
H	-4.00304	6.01698	1.192442
C	-3.70348	6.34787	3.30587
H	-2.6383	6.106531	3.330513
H	-3.86025	7.136729	4.0521
N	-4.48281	5.176713	3.724494
H	-5.49047	5.315652	3.644591
C	-4.13355	3.895476	3.43299
N	-2.87517	3.508353	3.274369
H	-2.04666	4.094094	3.47853
H	-2.72507	2.497871	3.170426
N	-5.13073	2.990616	3.352242
H	-6.06555	3.370631	3.223669
H	-4.92522	2.105084	2.879824
C	-4.13077	7.810335	-1.05046
O	-4.75646	6.745331	-1.12496
N	-3.32978	8.286804	-2.03655
H	-2.68969	9.041834	-1.81304
C	-2.88687	7.494636	-3.17318
H	-3.38776	6.527304	-3.17651
C	-1.36033	7.360932	-3.07691
O	-0.69403	8.312988	-2.65656
N	-0.85705	6.172465	-3.46966
H	-1.51634	5.423495	-3.68547
C	0.562596	5.857425	-3.3424
H	1.089903	6.813065	-3.28648
C	0.897413	4.98874	-2.11466
H	0.490716	3.978285	-2.24079
H	1.987298	4.876893	-2.07629
C	0.40533	5.606082	-0.80113
H	0.790136	6.62552	-0.68941
H	-0.68778	5.671953	-0.78485
S	0.951044	4.721429	0.713092
C	-0.0968	3.219159	0.678863
H	0.148082	2.665017	1.589721
H	-1.15795	3.488793	0.702055
H	0.123882	2.601975	-0.19539
O	0.852463	-0.42607	6.356865

C	0.73199	-1.69794	6.425961
O	1.411513	-2.42834	5.643195
C	-0.2555	-2.31198	7.39567
C	-0.44344	-1.51049	8.686939
H	-1.20742	-2.35079	6.849596
H	0.055833	-3.34357	7.592414
H	-0.78354	-0.49747	8.45817
H	-1.19151	-1.99008	9.32817
O	0.934005	-0.42746	3.502404
O	-2.01059	-0.39985	5.466542
Fe	2.172002	-0.54074	4.694894
C	-1.31088	-0.95067	1.293257
H	-0.92594	0.063292	1.436342
H	-0.81863	-1.32403	0.394038
C	-2.83383	-0.8448	1.077933
H	-3.33927	-1.74661	1.442112
C	-3.44812	0.341634	1.868197
O	-4.51536	0.861082	1.420554
O	-2.85851	0.685027	2.914398
H	-0.60305	-3.17024	-0.22411
N	-3.18813	-0.7413	-0.38237
H	-2.36474	-0.43864	-1.00677
H	-3.97075	-0.08803	-0.49145
H	-3.46356	-1.68173	-0.75964
C	-0.9329	-1.78964	2.517095
H	-1.6843	-1.73532	3.310361
H	-0.0332	-1.18515	3.046254
C	-0.39665	-3.20989	2.327302
H	-0.22699	-3.63642	3.322016
H	0.58238	-3.19748	1.834266
C	-1.29838	-4.17112	1.532767
H	-1.10498	-5.20949	1.809037
H	-2.35981	-3.98377	1.711854
N	-1.0678	-4.06476	0.049207
H	-0.36715	-4.75478	-0.28033
H	-1.97061	-4.10115	-0.47965
H	7.036851	-2.76805	8.340674
H	6.789328	-4.43442	4.037493
H	4.061961	-8.39334	-1.49326
H	4.437151	-7.36603	-0.09604
H	8.999996	-0.59098	1.15563
H	5.099385	2.724312	-7.28802
H	0.492333	-1.43501	9.253606
H	-5.64762	-3.37914	-5.11184
H	-5.90428	-8.51745	-3.90123
H	-10.4513	-4.65444	-1.5334
H	-7.62127	4.606723	-1.68121

H	-8.77248	5.710603	-0.91891
H	-5.28864	8.844232	0.40398
H	-3.13553	8.013659	-4.1087
H	0.89017	5.341102	-4.2521
H	-2.83821	0.076471	-6.33415
H	-2.28076	-0.13442	-4.65817
H	-1.09269	-0.09431	5.569728
H	-2.30542	-0.0016	4.628005
O	-1.21985	-0.36355	-2.15133
H	-0.71902	-1.20765	-2.11096
H	-0.4826	0.318091	-2.05143
O	-0.43995	4.875006	3.697989
H	0.203537	4.184598	3.974501
H	-0.09107	5.147575	2.825125
O	0.57307	-2.23174	-1.1899
H	1.143149	-3.03875	-1.18668
H	1.182672	-1.43691	-1.09686

^{5}**IM1**$_{A,C4}$

C	-2.077	1.654779	-4.69414
H	-3.05501	1.541601	-4.20969
C	-1.5624	3.089884	-4.54593
H	-0.64789	3.236427	-5.13081
H	-2.30482	3.791035	-4.95225
C	-1.25874	3.46524	-3.08244
H	-2.11815	3.235958	-2.43922
H	-0.41554	2.860588	-2.72273
C	-0.8722	4.931794	-2.96599
O	0.016023	5.42591	-3.67478
N	-1.57162	5.678799	-2.07782
H	-2.26097	5.261042	-1.46935
H	-1.37483	6.675481	-1.98471
C	-5.08252	8.725662	-2.44229
H	-6.01214	8.33232	-2.87065
C	-4.67575	7.960231	-1.17965
H	-4.50617	6.904353	-1.43079
H	-3.70585	8.336766	-0.83065
C	-5.68501	8.032902	-0.06882
N	-5.50673	7.348362	1.127758
C	-6.56596	7.612271	1.869485
H	-6.75165	7.233286	2.865273
N	-7.42594	8.4369	1.21905
H	-8.31003	8.775121	1.569859
C	-6.87778	8.713011	-0.02135
H	-7.3775	9.346868	-0.73673
C	2.633822	-5.89148	4.438553
C	3.978781	-5.16988	4.330235
C	1.538156	-5.53238	3.413839
O	4.650582	-4.96077	5.337068
C	1.254785	-4.06035	3.300439
C	0.811508	-3.12439	4.204139
N	1.446331	-3.36316	2.119374
C	1.122098	-2.06945	2.329812
N	0.73633	-1.89051	3.592777
H	2.831757	-6.97119	4.382872
H	1.811069	-5.90549	2.423079
H	0.624844	-6.06717	3.706494
H	0.539195	-3.25338	5.239724
H	1.18757	-1.2986	1.574229
H	1.847891	-3.80419	1.263567
N	4.39664	-4.79064	3.081733
H	3.859706	-5.02126	2.245848
C	5.694915	-4.16422	2.902145
H	6.335129	-4.45764	3.738723

C	5.72629	-2.62715	2.822359
O	6.763455	-2.06614	2.463876
N	4.578204	-1.98504	3.141973
C	4.362627	-0.53613	3.041227
C	4.992285	0.016685	1.744904
C	4.784105	0.248302	4.294453
O	5.650692	1.049566	1.712335
C	4.16229	1.652423	4.376773
C	2.635845	1.752046	4.53075
O	2.099619	2.858	4.463683
O	2.010363	0.628774	4.771794
H	3.80064	-2.55552	3.446065
H	3.279686	-0.42019	2.931012
H	4.488244	-0.34358	5.167794
H	5.875342	0.337405	4.304831
H	4.440412	2.237993	3.495702
H	4.590422	2.188567	5.234992
N	4.730155	-0.74943	0.643904
H	3.918794	-1.35591	0.690205
C	4.936434	-0.24961	-0.70956
H	4.786117	-1.12362	-1.35855
C	3.899109	0.804931	-1.10631
H	3.92323	1.628768	-0.38262
H	4.148903	1.232866	-2.07966
C	2.473842	0.226815	-1.16496
O	1.622359	0.876437	-1.82752
O	2.261228	-0.86068	-0.53176
C	6.390455	0.162736	-0.9666
O	6.685384	0.964529	-1.85841
N	7.333047	-0.5459	-0.28258
H	7.044166	-1.06049	0.547688
C	8.752102	-0.23914	-0.43352
H	8.866611	0.848899	-0.50325
C	9.543244	-0.7583	0.770747
H	9.408277	-1.83867	0.895206
H	9.202697	-0.26961	1.688243
H	10.60513	-0.55104	0.617719
C	9.357999	-0.77104	-1.75146
O	10.5044	-0.47738	-2.07442
N	8.532174	-1.55494	-2.50086
H	7.615323	-1.75513	-2.12387
C	8.843099	-2.01224	-3.83914
H	9.928241	-1.91535	-3.95156
C	8.456776	-3.50097	-4.03176
H	9.085498	-4.09789	-3.36097
H	8.713449	-3.76257	-5.06166
C	6.992452	-3.79158	-3.76504

C	6.539698	-4.10293	-2.4727
H	7.260494	-4.18664	-1.66144
C	5.181177	-4.30824	-2.21252
H	4.842077	-4.53063	-1.204
C	4.250371	-4.22523	-3.25153
H	3.19521	-4.3886	-3.04919
C	4.690697	-3.93472	-4.54561
H	3.974823	-3.87181	-5.36133
C	6.045882	-3.71404	-4.79898
H	6.3812	-3.46634	-5.8022
C	8.230714	-1.1417	-4.968
O	8.336865	-1.51737	-6.13902
N	7.638429	0.012181	-4.5854
H	7.562946	0.240642	-3.59701
C	7.109219	0.986694	-5.53166
H	7.379047	0.635315	-6.52936
C	5.59452	1.214292	-5.39822
H	5.388459	1.554171	-4.37449
H	5.326268	2.048958	-6.06478
C	4.692752	0.006771	-5.71876
H	4.960672	-0.80454	-5.02777
C	3.217471	0.365002	-5.47632
H	2.567251	-0.49543	-5.67655
H	3.028402	0.684224	-4.44461
H	2.898631	1.179927	-6.1412
C	4.898603	-0.51259	-7.15111
H	5.916509	-0.88437	-7.30781
H	4.205905	-1.33609	-7.36643
H	4.703871	0.280245	-7.88745
C	-5.3456	-3.48981	-5.18031
H	-4.60417	-4.17655	-5.60153
C	-4.7699	-2.79799	-3.93258
H	-3.96764	-2.11244	-4.22993
H	-5.54257	-2.17552	-3.46619
C	-4.20701	-3.73447	-2.85797
O	-4.44614	-4.97067	-2.93745
O	-3.53407	-3.18574	-1.92624
C	-6.66607	-4.21012	-4.92335
O	-7.73098	-3.60725	-4.78119
N	-6.58933	-5.57674	-4.88476
H	-5.66776	-5.95166	-4.6935
C	-7.74476	-6.37553	-4.52249
H	-8.61365	-5.99681	-5.06538
C	-8.14443	-6.40195	-3.03574
O	-9.17907	-6.96239	-2.6942
N	-7.29955	-5.76534	-2.17661
H	-6.41344	-5.40212	-2.52313

C	-7.55152	-5.72494	-0.74343
H	-8.10002	-6.63357	-0.4746
C	-6.22942	-5.69519	0.043602
H	-6.48938	-5.55278	1.095378
H	-5.61778	-4.84565	-0.28383
C	-5.46754	-7.0151	-0.08106
O	-5.85044	-8.03614	0.475051
N	-4.31914	-6.95628	-0.83188
H	-4.21882	-6.22472	-1.54522
H	-3.90934	-7.85909	-1.04192
C	-8.45227	-4.55581	-0.27989
O	-8.73854	-4.44834	0.910924
N	-8.89353	-3.69352	-1.23094
H	-8.6586	-3.86327	-2.20153
C	-9.87423	-2.67003	-0.89601
H	-9.61777	-2.28757	0.096302
C	-9.93267	-1.53458	-1.92798
H	-10.178	-1.9527	-2.91269
H	-10.7845	-0.89809	-1.64808
C	-8.67356	-0.69751	-2.04029
C	-7.97547	-0.61292	-3.25224
H	-8.32142	-1.18019	-4.11142
C	-6.80867	0.141108	-3.36558
H	-6.2671	0.198131	-4.30521
C	-6.29857	0.815369	-2.25223
O	-5.10707	1.472751	-2.39616
H	-4.79643	1.844346	-1.52634
C	-6.99741	0.767939	-1.03749
H	-6.61651	1.311503	-0.17659
C	-8.17257	0.020806	-0.94586
H	-8.69906	-0.00996	0.005992
C	3.541152	-7.78515	0.193052
H	4.390788	-7.17349	0.510332
C	2.726044	-7.07387	-0.88745
H	3.346492	-6.91922	-1.78143
H	1.879693	-7.68717	-1.21733
C	2.17638	-5.69217	-0.5037
O	1.213323	-5.25493	-1.19569
O	2.738584	-5.0621	0.449777
C	-0.24553	-2.93254	9.59346
C	0.203556	-2.08862	8.446176
C	-0.3954	-1.74778	7.259479
N	1.43593	-1.45273	8.407401
C	1.532533	-0.77566	7.232824
N	0.438177	-0.93359	6.51503
H	-0.29771	-2.36118	10.52955
H	-1.24475	-3.32817	9.39164

H	-1.3664	-2.03348	6.883901
H	2.389939	-0.19731	6.927368
H	2.151322	-1.49766	9.118887
C	-0.05446	9.97041	0.07448
H	0.825714	10.61948	0.153777
C	-0.11983	9.032684	1.305298
H	0.799538	8.435588	1.365636
H	-0.13769	9.66426	2.204183
C	-1.34136	8.108895	1.283162
H	-2.23809	8.705396	1.064409
H	-1.24201	7.403244	0.454133
C	-1.59997	7.303858	2.563779
H	-0.73639	6.708404	2.869276
H	-1.83674	7.965012	3.405765
N	-2.75765	6.423159	2.358643
H	-3.62235	6.912461	2.127425
C	-2.6789	5.2108	1.759017
N	-1.57001	4.473127	1.779807
H	-0.7888	4.674718	2.430193
H	-1.68984	3.489419	1.531402
N	-3.75635	4.779835	1.080854
H	-4.57088	5.384613	1.050469
H	-3.81045	3.82254	0.710854
C	-0.05358	9.216433	-1.25384
O	-1.07023	8.639034	-1.66683
N	1.117536	9.223738	-1.93019
H	1.95744	9.582779	-1.48816
C	1.388947	8.387722	-3.09073
H	0.555771	7.705275	-3.26029
C	2.706542	7.65056	-2.81583
O	3.624848	8.247752	-2.24544
N	2.748419	6.366398	-3.22932
H	1.872622	5.937283	-3.53164
C	3.875546	5.493263	-2.91106
H	4.720131	6.145564	-2.67639
C	3.586727	4.535419	-1.73944
H	2.802973	3.826115	-2.03228
H	4.489873	3.942022	-1.55384
C	3.17877	5.272726	-0.45951
H	3.968141	5.965809	-0.14891
H	2.275438	5.868113	-0.63077
S	2.888078	4.191758	0.996194
C	1.366865	3.310289	0.485247
H	1.064371	2.689077	1.334045
H	0.5693	4.025083	0.259274
H	1.54121	2.661248	-0.37483
O	-1.30372	1.238004	5.416963

C	-2.17315	0.301732	5.354688
O	-1.8789	-0.77386	4.74978
C	-3.53257	0.48965	5.995406
C	-3.44354	1.02397	7.431824
H	-4.06317	1.218535	5.36998
H	-4.06951	-0.46303	5.951208
H	-2.91858	1.983052	7.451068
H	-4.44608	1.169077	7.849433
O	-0.05288	0.768522	2.741136
O	-3.22885	3.013641	4.02849
Fe	0.17446	0.043103	4.39264
C	-2.68572	-1.11084	1.211432
H	-2.7995	-0.71812	2.234734
H	-1.60978	-1.01684	1.006007
C	-3.46359	-0.18109	0.281028
H	-4.54031	-0.35868	0.393755
C	-3.23929	1.331	0.576657
O	-3.89178	2.15676	-0.13212
O	-2.47293	1.651654	1.519161
H	-0.81535	-3.5552	-0.43229
N	-3.15328	-0.46732	-1.16407
H	-2.11382	-0.34357	-1.42949
H	-3.7023	0.155959	-1.77587
H	-3.38852	-1.45549	-1.428
C	-3.1131	-2.54339	1.165116
C	-2.26492	-3.609	1.786596
C	-1.97047	-4.81107	0.871748
H	-1.18646	-5.43495	1.3098
H	-2.85195	-5.43437	0.702848
N	-1.49266	-4.36159	-0.47323
H	-0.94367	-5.09144	-0.94638
H	-2.30007	-4.04583	-1.07336
H	0.422559	-3.78716	9.762458
H	2.287006	-5.69536	5.457422
H	3.927656	-8.74236	-0.17644
H	2.933201	-7.9935	1.08201
H	6.154436	-4.52597	1.97781
H	7.625126	1.942431	-5.36677
H	-2.90031	0.329935	8.084157
H	-5.55829	-2.72407	-5.93436
H	-7.58049	-7.40954	-4.84157
H	-10.8723	-3.12436	-0.80657
H	-4.29758	8.643042	-3.19949
H	-5.22942	9.791872	-2.23118
H	-0.94044	10.61664	0.070859
H	1.515195	9.011088	-3.98676
H	4.132822	4.910249	-3.80296

H	-2.19878	1.395646	-5.75321
H	-1.39267	0.930016	-4.23913
H	-2.44423	2.760209	4.548558
H	-3.09026	2.537718	3.189789
O	-0.70964	-0.40888	-2.19197
H	-0.37394	-1.32818	-2.10037
H	0.104259	0.146863	-2.00072
O	0.700016	4.980183	3.363124
H	0.97498	4.208821	3.906368
H	1.437678	5.049017	2.722429
O	0.443746	-2.64251	-1.09999
H	0.947272	-3.47856	-1.26823
H	1.113104	-1.93398	-0.84511
H	-0.92917	0.954028	2.345585
H	-4.1233	-2.79372	0.848004
H	-1.31746	-3.17201	2.11596
H	-2.74323	-4.01468	2.693507

[5]**TS2**$_{A,C4}$

C	-1.76896	2.008027	-4.89478
H	-2.80151	1.66174	-4.75059
C	-1.56891	3.391679	-4.26712
H	-0.57504	3.782417	-4.51052
H	-2.2901	4.101258	-4.69429
C	-1.72496	3.362616	-2.73944
H	-2.65752	2.858084	-2.45963
H	-0.90026	2.781357	-2.30198
C	-1.69992	4.753541	-2.12133
O	-0.98453	5.6581	-2.57746
N	-2.51049	4.947137	-1.05466
H	-3.05507	4.183597	-0.66241
H	-2.51998	5.86019	-0.60753
C	-6.38241	8.03535	-0.66645
H	-7.27308	7.650739	-1.1776
C	-5.94458	7.115204	0.476857
H	-5.69758	6.12398	0.072666
H	-5.00786	7.49671	0.900302
C	-6.97235	6.95056	1.560086
N	-6.76536	6.100682	2.640749
C	-7.85368	6.16996	3.383435
H	-8.03182	5.623818	4.299821
N	-8.75932	7.02744	2.846864
H	-9.67188	7.243737	3.220224
C	-8.21138	7.531845	1.680295
H	-8.74407	8.233067	1.057602
C	4.811209	-6.52659	3.066607
C	5.736221	-5.3112	3.170815
C	3.572721	-6.42476	2.151453
O	6.334547	-5.08223	4.219595
C	2.716618	-5.21757	2.408384
C	1.961696	-4.8068	3.48217
N	2.604345	-4.20557	1.471718
C	1.814335	-3.24571	1.979135
N	1.404179	-3.57488	3.206003
H	5.424171	-7.37823	2.738853
H	3.877648	-6.40555	1.10098
H	2.981929	-7.33947	2.287361
H	1.781548	-5.30406	4.423253
H	1.549691	-2.33937	1.455475
H	3.130671	-4.21303	0.552945
N	5.885297	-4.52526	2.058559
H	5.425742	-4.74695	1.172822
C	6.837536	-3.42928	2.061424
H	7.576851	-3.62021	2.844197

C	6.284684	-2.01314	2.295331
O	7.023711	-1.04379	2.114728
N	4.988875	-1.92657	2.679614
C	4.225137	-0.68035	2.815761
C	4.62235	0.330051	1.716585
C	4.268019	-0.05784	4.221716
O	4.919889	1.491434	1.966346
C	3.120383	0.941987	4.473513
C	1.709622	0.348819	4.371761
O	0.764541	1.019542	3.915568
O	1.592815	-0.87255	4.797358
H	4.485313	-2.79841	2.777813
H	3.188531	-0.96792	2.606929
H	4.215002	-0.87319	4.949989
H	5.226043	0.454435	4.352175
H	3.193491	1.786823	3.785492
H	3.223107	1.347342	5.490427
N	4.603388	-0.1993	0.456874
H	4.000054	-1.00261	0.306779
C	4.629592	0.658123	-0.72337
H	4.760797	-0.02788	-1.57177
C	3.315467	1.415669	-0.91834
H	3.136196	2.048429	-0.04159
H	3.400124	2.088751	-1.7736
C	2.095228	0.503868	-1.13854
O	1.107064	1.062886	-1.69507
O	2.154772	-0.70539	-0.75177
C	5.864197	1.569158	-0.76619
O	5.859672	2.623901	-1.41058
N	7.001503	1.067865	-0.20777
H	6.917163	0.305152	0.462529
C	8.221474	1.871277	-0.16639
H	7.948981	2.908304	0.061819
C	9.172583	1.34566	0.913086
H	9.431061	0.296106	0.73311
H	8.703578	1.410994	1.898967
H	10.08888	1.940961	0.901633
C	8.945996	1.955454	-1.52874
O	9.898185	2.713017	-1.68467
N	8.445346	1.145817	-2.50269
H	7.671566	0.545146	-2.25041
C	8.867527	1.16812	-3.88721
H	9.860886	1.628986	-3.90448
C	8.972099	-0.27113	-4.45695
H	9.793876	-0.77889	-3.93892
H	9.240332	-0.17526	-5.51235
C	7.690077	-1.06551	-4.30089

C	7.443912	-1.81391	-3.13874
H	8.216524	-1.8801	-2.37507
C	6.222452	-2.46543	-2.94421
H	6.038628	-3.02217	-2.02887
C	5.22812	-2.39746	-3.92437
H	4.27944	-2.90351	-3.76702
C	5.47292	-1.67838	-5.09778
H	4.709297	-1.62502	-5.86969
C	6.688041	-1.01546	-5.2827
H	6.863289	-0.43635	-6.18532
C	7.986926	2.052185	-4.81002
O	8.200646	2.046803	-6.02564
N	7.045564	2.809259	-4.20208
H	6.906659	2.734335	-3.19715
C	6.221712	3.77689	-4.91606
H	6.570886	3.782951	-5.95041
C	4.714047	3.488554	-4.82614
H	4.428523	3.478183	-3.76562
H	4.187945	4.340943	-5.28437
C	4.228223	2.184916	-5.48817
H	4.745514	1.348513	-4.99833
C	2.718671	2.003024	-5.26077
H	2.364326	1.066217	-5.70784
H	2.457903	1.976334	-4.19613
H	2.15198	2.823353	-5.72347
C	4.565856	2.130527	-6.98686
H	5.646704	2.146035	-7.16213
H	4.16809	1.213519	-7.43999
H	4.118589	2.980608	-7.5217
C	-5.17667	-2.59224	-5.77225
H	-4.37804	-3.06128	-6.355
C	-4.62404	-2.10357	-4.42318
H	-3.87405	-1.32021	-4.59439
H	-5.42333	-1.63998	-3.83653
C	-3.96989	-3.18452	-3.55888
O	-3.87766	-4.35615	-4.02198
O	-3.56684	-2.82849	-2.40495
C	-6.36301	-3.53916	-5.61314
O	-7.47766	-3.15075	-5.26241
N	-6.10539	-4.85396	-5.90023
H	-5.12767	-5.11736	-5.88549
C	-7.10012	-5.8804	-5.65329
H	-8.07203	-5.50202	-5.97765
C	-7.28279	-6.35072	-4.19734
O	-8.16981	-7.15	-3.92331
N	-6.42508	-5.81539	-3.28294
H	-5.65777	-5.22817	-3.60321

C	-6.47753	-6.17836	-1.8741
H	-6.84753	-7.20615	-1.80579
C	-5.0781	-6.10405	-1.2304
H	-5.21711	-6.29583	-0.16314
H	-4.67056	-5.09395	-1.35374
C	-4.12559	-7.16948	-1.77466
O	-4.24335	-8.35113	-1.48829
N	-3.10321	-6.69307	-2.57865
H	-3.29462	-5.83961	-3.12352
H	-2.61573	-7.43126	-3.07674
C	-7.45723	-5.3324	-1.02573
O	-7.61641	-5.60491	0.162381
N	-8.09818	-4.30605	-1.64015
H	-7.96795	-4.15747	-2.63336
C	-9.11979	-3.5542	-0.921
H	-8.7228	-3.31687	0.071383
C	-9.55717	-2.278	-1.65514
H	-9.92118	-2.54423	-2.65601
H	-10.4289	-1.888	-1.11029
C	-8.49855	-1.19847	-1.77635
C	-8.05481	-0.76525	-3.03285
H	-8.45051	-1.22668	-3.93269
C	-7.0668	0.210318	-3.15633
H	-6.71957	0.534027	-4.13315
C	-6.47919	0.766691	-2.01534
O	-5.44928	1.645729	-2.194
H	-5.06797	1.937953	-1.31988
C	-6.94021	0.376843	-0.74867
H	-6.51337	0.830887	0.141871
C	-7.93862	-0.59248	-0.64285
H	-8.27563	-0.88859	0.348736
C	5.819402	-6.75818	-1.64216
H	6.474536	-6.10443	-1.05844
C	4.82507	-5.94355	-2.47046
H	5.367185	-5.32345	-3.19749
H	4.175245	-6.5963	-3.06418
C	3.911706	-4.99432	-1.67488
O	2.865049	-4.6114	-2.24374
O	4.297273	-4.64681	-0.50079
C	1.347493	-5.86259	8.843113
C	1.366565	-4.73478	7.864606
C	0.545944	-4.40939	6.814386
N	2.327511	-3.73295	7.87909
C	2.062568	-2.86789	6.866638
N	0.989066	-3.25161	6.204036
H	1.237329	-5.50933	9.876749
H	0.503119	-6.523	8.627581

H	-0.33442	-4.9187	6.451602
H	2.646504	-1.99051	6.636768
H	3.106569	-3.66575	8.518468
C	-1.8493	9.533931	1.92821
H	-1.09626	10.32398	2.031642
C	-1.72316	8.541708	3.107702
H	-0.75524	8.025479	3.049216
H	-1.7083	9.123197	4.039955
C	-2.85734	7.513647	3.135255
H	-3.82353	8.038346	3.174358
H	-2.84671	6.970836	2.187506
C	-2.80909	6.488509	4.276314
H	-1.81954	6.035759	4.376015
H	-3.04291	6.943128	5.245302
N	-3.79583	5.42061	4.037885
H	-4.75251	5.76387	3.991952
C	-3.55141	4.481805	3.074164
N	-2.32547	4.04014	2.838388
H	-1.55158	4.097676	3.521819
H	-2.24176	3.272823	2.15295
N	-4.59314	4.022286	2.363791
H	-5.45259	4.572687	2.372744
H	-4.43688	3.362648	1.598355
C	-1.73993	8.869592	0.554038
O	-2.57227	8.039896	0.162676
N	-0.68897	9.277318	-0.19742
H	0.06618	9.795244	0.239885
C	-0.29577	8.636559	-1.44257
H	-0.959	7.797079	-1.65413
C	1.168981	8.199783	-1.29507
O	1.947784	8.893114	-0.63292
N	1.489809	7.050598	-1.92585
H	0.723538	6.493532	-2.30779
C	2.790168	6.407328	-1.7569
H	3.470667	7.165885	-1.36236
C	2.722101	5.184322	-0.82381
H	2.03557	4.448186	-1.25934
H	3.70893	4.707838	-0.78906
C	2.264106	5.548322	0.59242
H	2.995958	6.203301	1.077392
H	1.311824	6.087807	0.564916
S	2.074726	4.111985	1.720271
C	0.622675	3.273157	0.988147
H	0.340494	2.483731	1.687395
H	-0.21351	3.96958	0.878682
H	0.852616	2.811261	0.02555
O	-1.49246	-1.52271	5.53116

C	-2.10412	-2.60838	5.278448
O	-1.48712	-3.53571	4.654464
C	-3.54164	-2.82369	5.707672
C	-4.24636	-1.56768	6.222943
H	-4.06943	-3.25325	4.847526
H	-3.53088	-3.61516	6.470857
H	-4.25493	-0.78517	5.458632
H	-5.28091	-1.80072	6.500282
O	-0.43165	-1.42062	2.807014
O	-2.84572	0.727459	4.051332
Fe	0.190863	-2.22479	4.340923
C	-2.23122	-0.86905	0.866716
H	-2.23463	-0.45102	1.876747
H	-1.23873	-0.65497	0.457807
C	-3.28315	-0.05625	0.111627
H	-4.29373	-0.36614	0.403134
C	-3.16359	1.458242	0.462611
O	-4.01792	2.264318	-0.04665
O	-2.27454	1.788073	1.272409
H	-0.59562	-3.77537	-1.43023
N	-3.20031	-0.26827	-1.37607
H	-2.19455	-0.25757	-1.75388
H	-3.77528	0.421662	-1.87981
H	-3.53257	-1.21979	-1.66235
C	-2.47298	-2.3298	0.995345
C	-1.42261	-3.38448	1.057454
C	-1.67837	-4.61409	0.182562
H	-0.95632	-5.40187	0.416762
H	-2.68475	-5.02009	0.316982
N	-1.50971	-4.28759	-1.27075
H	-1.55148	-5.15244	-1.82365
H	-2.29887	-3.69083	-1.63692
H	2.262995	-6.46636	8.793604
H	4.509787	-6.74176	4.096024
H	6.448178	-7.38215	-2.28905
H	5.304351	-7.42345	-0.93816
H	7.36	-3.39171	1.100813
H	6.415388	4.77344	-4.49572
H	-3.736	-1.16528	7.103654
H	-5.54196	-1.72671	-6.33591
H	-6.86915	-6.7623	-6.25913
H	-9.99864	-4.19432	-0.75658
H	-5.57789	8.121686	-1.40226
H	-6.60878	9.044971	-0.30179
H	-2.83305	10.01897	1.971885
H	-0.36844	9.351408	-2.27401
H	3.162146	6.093666	-2.7395

H	-1.57738	2.035436	-5.97425
H	-1.10654	1.259082	-4.44687
H	-2.20671	0.158508	4.516104
H	-2.36717	1.071282	3.279475
O	-0.7979	-0.52011	-2.53319
H	-0.38555	-1.40609	-2.52953
H	-0.06352	0.089376	-2.19761
O	0.041043	3.830319	4.348751
H	0.222687	2.865744	4.324517
H	0.720783	4.195239	3.746534
O	0.806398	-2.8602	-1.69442
H	1.58423	-3.43836	-1.91545
H	1.203975	-2.04009	-1.28814
H	-0.2577	-0.45896	2.894084
H	-3.49069	-2.64028	1.244447
H	-0.45132	-2.97022	0.787442
H	-1.32444	-3.74747	2.091264

[5]**Pr**$_{A,C4}$

C	2.712173	0.113258	-5.91315
H	3.531206	0.743424	-5.53981
C	3.011349	-1.371	-5.67823
H	2.226323	-1.99203	-6.1224
H	3.944384	-1.65295	-6.18367
C	3.120461	-1.71185	-4.18708
H	3.8943	-1.09925	-3.70907
H	2.170089	-1.47209	-3.68966
C	3.414662	-3.18505	-3.93525
O	3.095191	-4.05881	-4.75519
N	4.041259	-3.48285	-2.77368
H	4.223096	-2.77043	-2.07389
H	4.252232	-4.4585	-2.57238
C	8.305499	-4.67809	-2.97337
H	8.955883	-3.83983	-3.2508
C	7.681353	-4.47667	-1.58966
H	7.067259	-3.56574	-1.59672
H	6.98219	-5.29858	-1.39345
C	8.680875	-4.37606	-0.4728
N	8.28896	-4.13996	0.84021
C	9.39638	-4.09199	1.556362
H	9.455059	-3.9159	2.621861
N	10.49177	-4.28968	0.779105
H	11.45244	-4.28985	1.089075
C	10.05065	-4.47046	-0.52021
H	10.7354	-4.63845	-1.33638
C	-5.45381	2.331331	6.222321
C	-6.08899	1.118846	5.533039
C	-4.37486	3.134346	5.459061
O	-6.39424	0.12546	6.190015
C	-3.2544	2.29972	4.910501
C	-2.16636	1.669473	5.474215
N	-3.2146	1.958688	3.57066
C	-2.14688	1.160642	3.376727
N	-1.48311	0.960067	4.507599
H	-6.26935	3.014403	6.500476
H	-4.83056	3.684226	4.62926
H	-3.97502	3.887666	6.14866
H	-1.82964	1.678589	6.501271
H	-1.87182	0.761565	2.413695
H	-3.89333	2.31333	2.853372
N	-6.3327	1.207306	4.188907
H	-6.07632	2.030879	3.640862
C	-7.04198	0.149728	3.493606
H	-7.64672	-0.39663	4.222955

C	-6.19903	-0.90086	2.750281
O	-6.76708	-1.72746	2.031643
N	-4.86046	-0.83782	2.930655
C	-3.88031	-1.80259	2.416985
C	-4.17227	-2.17967	0.95219
C	-3.70944	-3.03716	3.32238
O	-4.17465	-3.34144	0.561656
C	-2.38538	-3.79655	3.116823
C	-1.10161	-3.07825	3.555488
O	-0.06352	-3.77573	3.726597
O	-1.15333	-1.81318	3.711422
H	-4.52111	-0.12531	3.563865
H	-2.92445	-1.27332	2.422258
H	-3.77785	-2.69431	4.36178
H	-4.54491	-3.72177	3.145129
H	-2.28047	-4.06719	2.058753
H	-2.41854	-4.74877	3.65879
N	-4.37399	-1.11761	0.116981
H	-4.2698	-0.18298	0.488003
C	-4.44467	-1.2559	-1.33581
H	-4.59601	-0.24067	-1.72092
C	-3.13765	-1.77341	-1.94319
H	-2.82017	-2.68731	-1.4363
H	-3.32516	-2.04445	-2.98814
C	-1.99493	-0.74936	-1.93465
O	-0.83459	-1.25113	-2.10061
O	-2.25769	0.480497	-1.83763
C	-5.65374	-2.07823	-1.81773
O	-5.70025	-2.48155	-2.98661
N	-6.69555	-2.24455	-0.96079
H	-6.57209	-2.01694	0.025249
C	-7.7945	-3.14675	-1.308
H	-7.37043	-4.02591	-1.80713
C	-8.54277	-3.59129	-0.04726
H	-8.93236	-2.73006	0.505603
H	-7.87145	-4.13961	0.619835
H	-9.37611	-4.23548	-0.3382
C	-8.78238	-2.57048	-2.34473
O	-9.65255	-3.28397	-2.83412
N	-8.60674	-1.25806	-2.66207
H	-7.87161	-0.75717	-2.18009
C	-9.32334	-0.5791	-3.72285
H	-10.1897	-1.20711	-3.9559
C	-9.82451	0.814508	-3.26258
H	-10.5753	0.658609	-2.47928
H	-10.3168	1.275666	-4.1226
C	-8.70917	1.701244	-2.74766

C	-8.30679	1.643238	-1.4038
H	-8.85589	1.008701	-0.71084
C	-7.20714	2.374608	-0.94788
H	-6.89678	2.299678	0.091105
C	-6.5005	3.199905	-1.82719
H	-5.63682	3.75312	-1.46912
C	-6.91443	3.292547	-3.15854
H	-6.37756	3.93792	-3.84954
C	-8.00262	2.547036	-3.61661
H	-8.29904	2.595545	-4.66073
C	-8.5208	-0.45596	-5.04564
O	-8.96862	0.239383	-5.96216
N	-7.37047	-1.16297	-5.11332
H	-7.04864	-1.68859	-4.30358
C	-6.52332	-1.19963	-6.29836
H	-7.07524	-0.70205	-7.09841
C	-5.14102	-0.56284	-6.0733
H	-4.65638	-1.09165	-5.24208
H	-4.53032	-0.75987	-6.96827
C	-5.13748	0.949402	-5.78078
H	-5.77602	1.121513	-4.90426
C	-3.72015	1.422481	-5.42014
H	-3.70764	2.502012	-5.22324
H	-3.33773	0.922679	-4.52243
H	-3.01731	1.228659	-6.24257
C	-5.71374	1.769689	-6.94586
H	-6.7661	1.528869	-7.12874
H	-5.6528	2.844079	-6.73018
H	-5.15061	1.588444	-7.87256
C	3.433766	5.756243	-4.69525
H	2.46941	6.096363	-5.08576
C	3.227355	4.603271	-3.70343
H	2.809873	3.733557	-4.22608
H	4.195894	4.28685	-3.29993
C	2.308065	4.932249	-2.52188
O	1.887435	6.113222	-2.39504
O	2.039531	3.971318	-1.72852
C	4.217888	6.912409	-4.08519
O	5.37713	6.783	-3.68617
N	3.561253	8.109871	-4.02778
H	2.555128	8.079429	-4.12061
C	4.160936	9.268408	-3.39138
H	5.18081	9.386938	-3.76551
C	4.27242	9.25144	-1.85529
O	4.876658	10.1524	-1.286
N	3.689574	8.19925	-1.21825
H	3.178993	7.497823	-1.75244

C	3.813609	8.024529	0.220382
H	3.79041	9.012874	0.687998
C	2.653972	7.177562	0.776045
H	2.860936	7.023461	1.838598
H	2.644451	6.202595	0.276544
C	1.298576	7.874262	0.661098
O	1.008868	8.850969	1.331605
N	0.407587	7.275131	-0.22082
H	0.823076	6.819313	-1.04669
H	-0.40824	7.850455	-0.4089
C	5.142984	7.37877	0.679447
O	5.392017	7.308762	1.881142
N	5.965971	6.879464	-0.27785
H	5.749803	7.018833	-1.25906
C	7.276005	6.361625	0.097609
H	7.14569	5.751054	0.996425
C	7.950791	5.562859	-1.02479
H	8.0145	6.188577	-1.92402
H	8.988617	5.390431	-0.70413
C	7.307702	4.236377	-1.38826
C	6.986016	3.944881	-2.72105
H	7.138611	4.704175	-3.4825
C	6.431634	2.718862	-3.08527
H	6.180838	2.501281	-4.11958
C	6.168755	1.751552	-2.10997
O	5.572213	0.586706	-2.51259
H	5.301011	0.024228	-1.73473
C	6.493352	2.017183	-0.77269
H	6.304181	1.262946	-0.01269
C	7.060628	3.245586	-0.42809
H	7.308308	3.429894	0.614998
C	-6.99604	5.224606	2.661641
H	-7.53537	4.287327	2.828988
C	-6.12362	5.140475	1.408855
H	-6.75685	4.991707	0.523642
H	-5.58828	6.080928	1.234967
C	-5.0783	4.012431	1.397232
O	-4.14213	4.120542	0.569088
O	-5.24557	3.034184	2.20545
C	0.078724	-2.75583	9.914884
C	-0.27159	-2.18706	8.579298
C	0.495207	-1.64909	7.576179
N	-1.57353	-2.11796	8.10193
C	-1.54238	-1.5588	6.862882
N	-0.30505	-1.26249	6.518186
H	-0.21635	-3.80971	10.00587
H	1.160765	-2.70204	10.06327

H	1.566931	-1.51486	7.527615
H	-2.41929	-1.39407	6.255451
H	-2.40352	-2.43601	8.581362
C	4.725733	-8.58854	-1.26871
H	4.247899	-9.55821	-1.45153
C	4.333905	-8.07546	0.135571
H	3.248068	-7.91979	0.184045
H	4.56325	-8.87299	0.855893
C	5.059816	-6.78426	0.525759
H	6.140136	-6.91257	0.36379
H	4.747131	-5.99058	-0.15612
C	4.835798	-6.29707	1.964108
H	3.775722	-6.26339	2.227785
H	5.319733	-6.9584	2.692028
N	5.423932	-4.95723	2.129217
H	6.424512	-4.92856	1.939231
C	4.794298	-3.84418	1.657507
N	3.476956	-3.75387	1.581889
H	2.826855	-4.46312	1.963518
H	3.087718	-2.83569	1.332686
N	5.565188	-2.79818	1.290629
H	6.548036	-3.00365	1.119958
H	5.135665	-2.06507	0.72078
C	4.411309	-7.61697	-2.40709
O	4.892078	-6.47607	-2.43996
N	3.618827	-8.11356	-3.38966
H	3.078162	-8.95152	-3.20677
C	3.083725	-7.29796	-4.47096
H	3.530244	-6.30419	-4.43255
C	1.55591	-7.2653	-4.32313
O	0.957721	-8.291	-3.98494
N	0.97574	-6.07123	-4.57337
H	1.590343	-5.27116	-4.72993
C	-0.44397	-5.8369	-4.32168
H	-0.93161	-6.81439	-4.35444
C	-0.72181	-5.14603	-2.97097
H	-0.35919	-4.11104	-2.99307
H	-1.80863	-5.08624	-2.83575
C	-0.09744	-5.90124	-1.79284
H	-0.45357	-6.93652	-1.76935
H	0.992192	-5.93755	-1.89524
S	-0.49079	-5.21674	-0.13831
C	0.410312	-3.62232	-0.18382
H	0.410044	-3.24104	0.84054
H	1.447622	-3.7808	-0.49566
H	-0.07582	-2.89778	-0.84167
O	3.141007	-0.66612	6.007201

C	2.732663	0.506117	5.842955
O	1.646088	0.847086	5.247212
C	3.558154	1.675948	6.391184
C	4.888684	1.272815	7.024588
H	3.711311	2.382697	5.565284
H	2.925235	2.20917	7.113863
H	5.528922	0.76183	6.298287
H	5.425728	2.152465	7.399894
O	0.818964	0.253203	2.356858
O	1.811698	-1.86701	3.973715
Fe	0.236977	-0.41291	4.480538
C	1.356838	1.221883	0.186687
H	0.677725	0.406531	-0.07858
H	0.978573	2.104959	-0.32645
C	2.758449	0.944212	-0.36531
H	3.483558	1.622975	0.103249
C	3.275659	-0.48992	-0.07528
O	4.33298	-0.88182	-0.67609
O	2.660102	-1.1415	0.793401
H	-0.74309	3.638197	0.162481
N	2.777975	1.289903	-1.82487
H	1.909941	0.914647	-2.34275
H	3.648564	0.983855	-2.28216
H	2.683763	2.331217	-1.90751
C	1.335989	1.426791	1.720688
C	0.487687	2.608757	2.203186
C	0.810433	3.985251	1.614454
H	0.467454	4.755873	2.308359
H	1.881864	4.140295	1.454799
N	0.116243	4.260224	0.296906
H	-0.1516	5.256552	0.252759
H	0.779443	4.131737	-0.50896
H	-0.39743	-2.20601	10.73787
H	-5.04125	1.940595	7.156819
H	-7.73341	6.031383	2.570111
H	-6.39409	5.424396	3.556366
H	-7.71698	0.585984	2.751124
H	-6.38663	-2.24898	-6.59187
H	4.731482	0.580875	7.857993
H	4.024594	5.389881	-5.54329
H	3.599253	10.16214	-3.67886
H	7.933124	7.194825	0.386323
H	7.51729	-4.76114	-3.72694
H	8.901156	-5.59843	-3.0117
H	5.810534	-8.75852	-1.29472
H	3.333529	-7.75781	-5.43637
H	-0.84941	-5.22687	-5.13695

H	2.593719	0.32611	-6.98231
H	1.794789	0.415175	-5.39552
H	2.37816	-1.71545	4.772384
H	1.291723	-2.70757	4.052319
O	0.554169	0.63241	-3.12121
H	-0.12169	1.321972	-3.00192
H	0.063411	-0.17525	-2.71398
O	1.545514	-5.62713	2.451656
H	0.922631	-5.0959	3.002789
H	1.022224	-5.81129	1.644068
O	-1.99852	2.519664	0.128816
H	-2.86578	2.997128	0.253832
H	-2.10177	1.861383	-0.59832
H	1.402614	-0.48561	2.073079
H	2.366275	1.591653	2.075391
H	-0.56286	2.399345	2.005758
H	0.611355	2.665655	3.289386

^{5}**TS1**$_{A,C5}$

C	-2.11915	1.28553	-5.27841
H	-3.09666	0.787304	-5.3345
C	-2.27008	2.731913	-4.79433
H	-1.29606	3.233877	-4.80571
H	-2.91153	3.297491	-5.48161
C	-2.85535	2.811422	-3.38051
H	-3.78959	2.237923	-3.31949
H	-2.16165	2.349665	-2.66367
C	-3.1303	4.238017	-2.91638
O	-2.71539	5.224139	-3.54469
N	-3.86145	4.363164	-1.78573
H	-4.15253	3.548394	-1.24851
H	-4.05759	5.298165	-1.43832
C	-8.26427	6.688214	-1.27348
H	-9.10684	6.102018	-1.65963
C	-7.5573	5.971318	-0.11952
H	-7.1636	5.009	-0.47437
H	-6.67585	6.554227	0.172611
C	-8.4323	5.722573	1.076114
N	-7.96481	5.044532	2.196019
C	-8.97597	4.978232	3.040729
H	-8.95972	4.505635	4.013446
N	-10.0798	5.584398	2.532707
H	-10.9812	5.658068	2.98112
C	-9.74823	6.062787	1.276986
H	-10.4633	6.580895	0.657763
C	6.443876	-4.25762	4.082069
C	6.876139	-2.80096	4.264867
C	5.537208	-4.57427	2.880796
O	7.039161	-2.31767	5.38057
C	4.284713	-3.74851	2.854421
C	3.597012	-3.08359	3.843942
N	3.571953	-3.53341	1.69378
C	2.50046	-2.7796	1.997338
N	2.477682	-2.48269	3.298817
H	7.356158	-4.8657	4.015236
H	6.077268	-4.44762	1.932794
H	5.287786	-5.64382	2.912864
H	3.818893	-3.00994	4.896879
H	1.78604	-2.43494	1.265403
H	3.774742	-3.98072	0.750671
N	7.10775	-2.09017	3.115409
H	6.931036	-2.52512	2.220742
C	7.624625	-0.73594	3.1179
H	7.88672	-0.50195	4.154404

C	6.675383	0.355952	2.601964
O	7.153914	1.394519	2.142948
N	5.349974	0.106811	2.71015
C	4.290455	1.084751	2.418433
C	4.594088	1.871331	1.120917
C	4.00579	2.031009	3.597687
O	4.647058	3.093614	1.089599
C	2.593	2.630224	3.559844
C	1.424609	1.64755	3.740045
O	0.258383	2.047698	3.646635
O	1.774074	0.421236	4.003312
H	5.062698	-0.76817	3.133794
H	3.395241	0.48314	2.233844
H	4.148655	1.46692	4.525362
H	4.741586	2.840366	3.577381
H	2.441301	3.167779	2.617189
H	2.488176	3.389698	4.346144
N	4.79676	1.071862	0.030301
H	4.373682	0.151	0.062843
C	4.818277	1.603761	-1.33289
H	4.949189	0.719408	-1.96761
C	3.484631	2.245569	-1.74246
H	3.235795	3.064932	-1.06064
H	3.576197	2.681116	-2.74227
C	2.332169	1.220389	-1.74331
O	1.193263	1.647034	-2.06913
O	2.634997	0.028832	-1.41573
C	6.044426	2.466912	-1.64377
O	6.125	3.05559	-2.72935
N	7.083489	2.434424	-0.76678
H	6.943225	2.055349	0.167241
C	8.274929	3.243266	-1.0069
H	7.958665	4.212566	-1.40796
C	9.055039	3.459465	0.293853
H	9.332203	2.504501	0.752633
H	8.445659	4.011478	1.015526
H	9.96264	4.027513	0.074612
C	9.199596	2.672162	-2.10353
O	10.12033	3.348587	-2.54999
N	8.915953	1.405153	-2.52275
H	8.151974	0.92241	-2.06803
C	9.552994	0.784266	-3.6673
H	10.41223	1.420689	-3.90628
C	10.07817	-0.63705	-3.34531
H	10.90088	-0.53294	-2.62896
H	10.48802	-1.0359	-4.27755
C	9.032292	-1.58141	-2.78471

C	8.947139	-1.82435	-1.40601
H	9.661626	-1.34798	-0.73751
C	7.967082	-2.67423	-0.8851
H	7.94187	-2.8637	0.187079
C	7.040362	-3.29189	-1.72694
H	6.259392	-3.93535	-1.32783
C	7.124965	-3.06203	-3.10357
H	6.415382	-3.54057	-3.77369
C	8.111151	-2.22515	-3.62821
H	8.173082	-2.05741	-4.69949
C	8.67285	0.773811	-4.94581
O	9.015903	0.084548	-5.91108
N	7.582495	1.573046	-4.9309
H	7.352785	2.111625	-4.09894
C	6.694875	1.730084	-6.0776
H	7.191549	1.262674	-6.93013
C	5.291208	1.145775	-5.84503
H	4.863667	1.635953	-4.96027
H	4.659928	1.439875	-6.69769
C	5.217689	-0.38311	-5.66819
H	5.881513	-0.65603	-4.83593
C	3.792023	-0.81061	-5.2848
H	3.727519	-1.89967	-5.16923
H	3.469642	-0.36093	-4.33856
H	3.070085	-0.51751	-6.05918
C	5.701053	-1.13643	-6.91805
H	6.754476	-0.93004	-7.13374
H	5.596455	-2.2204	-6.78188
H	5.105401	-0.85809	-7.79896
C	-4.48055	-4.77214	-4.99517
H	-3.58304	-5.29799	-5.33695
C	-4.11677	-3.78235	-3.87686
H	-3.50267	-2.97187	-4.28623
H	-5.02926	-3.31144	-3.49175
C	-3.36264	-4.38932	-2.6883
O	-3.32664	-5.64284	-2.56579
O	-2.82738	-3.56301	-1.8795
C	-5.57086	-5.76083	-4.59557
O	-6.74397	-5.41846	-4.43464
N	-5.16484	-7.05994	-4.45674
H	-4.17436	-7.19407	-4.29771
C	-6.08008	-8.08267	-3.98631
H	-7.0166	-7.99553	-4.54224
C	-6.46712	-8.0567	-2.49635
O	-7.33071	-8.8232	-2.08522
N	-5.81851	-7.14632	-1.71971
H	-5.06367	-6.58795	-2.11571

C	-6.13251	-6.99425	-0.30648
H	-6.4128	-7.97781	0.082965
C	-4.91041	-6.48356	0.476933
H	-5.24285	-6.30371	1.502381
H	-4.56293	-5.53413	0.053134
C	-3.78809	-7.52121	0.520891
O	-3.87946	-8.54346	1.187684
N	-2.67082	-7.2009	-0.21209
H	-2.76494	-6.56816	-1.01513
H	-2.003	-7.95758	-0.30593
C	-7.3465	-6.08137	-0.01386
O	-7.74242	-5.95723	1.143379
N	-7.91997	-5.44554	-1.06809
H	-7.5829	-5.62821	-2.00653
C	-9.1566	-4.69789	-0.8873
H	-9.09125	-4.19185	0.080319
C	-9.42473	-3.69834	-2.02151
H	-9.46487	-4.23857	-2.97598
H	-10.4364	-3.29941	-1.85675
C	-8.43277	-2.55705	-2.13706
C	-7.73325	-2.33773	-3.33122
H	-7.87377	-3.02014	-4.16456
C	-6.81998	-1.29195	-3.45626
H	-6.27662	-1.12917	-4.38267
C	-6.571	-0.44555	-2.37125
O	-5.61339	0.520163	-2.52023
H	-5.43497	0.993446	-1.65644
C	-7.27623	-0.63387	-1.17452
H	-7.09373	0.031058	-0.33414
C	-8.19752	-1.67692	-1.07178
H	-8.73195	-1.81087	-0.13359
C	4.324601	-7.08445	-2.30433
H	5.140203	-6.41968	-2.60495
C	3.01332	-6.31335	-2.16039
H	2.718249	-5.86287	-3.11801
H	2.188209	-6.98664	-1.8921
C	3.031971	-5.18463	-1.12098
O	1.935154	-4.57863	-0.90842
O	4.120942	-4.91584	-0.54076
C	3.267087	-3.21349	9.184155
C	2.87703	-2.3996	7.994565
C	1.981522	-2.62002	6.978641
N	3.426096	-1.15638	7.71285
C	2.864258	-0.68799	6.569352
N	1.986123	-1.55326	6.100659
H	3.045247	-2.697	10.12718
H	2.711372	-4.15513	9.183657

H	1.324609	-3.46093	6.81597
H	3.104983	0.259019	6.113186
H	4.149978	-0.69266	8.243144
C	-3.97834	9.29762	0.63675
H	-3.41661	10.23779	0.584991
C	-3.54035	8.502097	1.888738
H	-2.48646	8.209742	1.786891
H	-3.59228	9.177984	2.753642
C	-4.40273	7.259677	2.127601
H	-5.45985	7.558136	2.188298
H	-4.32175	6.614274	1.250051
C	-4.05819	6.425193	3.368614
H	-2.99014	6.204425	3.4347
H	-4.32705	6.943775	4.295667
N	-4.80476	5.154726	3.342902
H	-5.81322	5.285029	3.363976
C	-4.44466	4.177698	2.454677
N	-3.17601	3.977888	2.134815
H	-2.39086	4.310015	2.725142
H	-2.96368	3.172551	1.526077
N	-5.42054	3.42927	1.916622
H	-6.37203	3.79577	1.959977
H	-5.18969	2.749909	1.186975
C	-3.83185	8.515148	-0.66982
O	-4.49867	7.496899	-0.8975
N	-2.94589	9.035708	-1.55414
H	-2.28709	9.740698	-1.2408
C	-2.51624	8.345279	-2.76036
H	-3.01929	7.380702	-2.83587
C	-0.98886	8.20485	-2.69485
O	-0.31579	9.107638	-2.18701
N	-0.49205	7.062829	-3.2171
H	-1.15861	6.332841	-3.47515
C	0.920841	6.712947	-3.10145
H	1.458079	7.643977	-2.90471
C	1.201549	5.681457	-1.99126
H	0.746516	4.718643	-2.25339
H	2.283553	5.507327	-1.94932
C	0.703434	6.145703	-0.61867
H	1.19425	7.080548	-0.32744
H	-0.3739	6.339302	-0.64526
S	1.034106	4.968234	0.748614
C	-0.06484	3.576873	0.285378
H	-0.11743	2.912973	1.151513
H	-1.07088	3.946065	0.065512
H	0.332417	3.018714	-0.56524
O	-0.75781	-0.6187	5.400754

C	-1.14956	-1.80934	5.622082
O	-0.46542	-2.78658	5.180965
C	-2.42023	-2.07078	6.410786
C	-3.26028	-0.82343	6.691385
H	-2.99495	-2.82044	5.852646
H	-2.12055	-2.56722	7.344827
H	-3.54464	-0.32628	5.759487
H	-4.17073	-1.09351	7.23922
O	-0.03405	-1.48503	2.673974
O	-2.63925	0.877073	3.68522
Fe	0.859762	-1.30266	4.166209
C	-2.52205	-1.13361	0.891441
H	-2.48495	-0.55682	1.816652
H	-1.52153	-1.04795	0.453326
C	-3.52833	-0.47722	-0.05257
H	-4.53803	-0.8724	0.103083
C	-3.59756	1.067417	0.128798
O	-4.63251	1.663462	-0.34569
O	-2.66853	1.62493	0.739174
H	-0.17771	-2.99101	-0.27856
N	-3.17796	-0.78105	-1.48969
H	-2.19973	-0.42743	-1.75826
H	-3.87779	-0.35851	-2.12019
H	-3.15588	-1.81427	-1.66958
C	-2.85106	-2.59527	1.268203
H	-3.14188	-3.17101	0.381112
H	-3.72068	-2.58847	1.936134
C	-1.67579	-3.28327	1.974115
H	-1.91952	-3.67685	2.965151
H	-0.86854	-2.40759	2.296645
C	-0.91412	-4.34232	1.196996
H	0.03899	-4.56805	1.683399
H	-1.4931	-5.27228	1.134696
N	-0.60669	-3.9408	-0.21057
H	0.148323	-4.53689	-0.61041
H	-1.45281	-3.9503	-0.83054
H	4.337202	-3.45767	9.181147
H	5.953519	-4.54072	5.017943
H	4.232289	-7.87802	-3.05594
H	4.616094	-7.5434	-1.3539
H	8.534209	-0.66322	2.513548
H	6.602149	2.802587	-6.29127
H	-2.69884	-0.10064	7.291562
H	-4.8781	-4.20648	-5.84553
H	-5.65701	-9.06766	-4.20608
H	-10.0068	-5.39304	-0.81739
H	-7.5601	6.855024	-2.09348

H	-8.64696	7.667252	-0.9595
H	-5.03943	9.561229	0.73344
H	-2.7761	8.94049	-3.64684
H	1.267107	6.31689	-4.06339
H	-1.67394	1.253019	-6.28011
H	-1.48369	0.702067	-4.60253
H	-1.84521	0.641503	4.198448
H	-2.27535	1.217852	2.852095
O	-0.71743	-0.23525	-2.39309
H	-0.17917	-1.02834	-2.17624
H	-0.07229	0.517326	-2.23687
O	-0.83942	4.668548	3.517779
H	-0.45795	3.771591	3.669455
H	-0.2772	5.039181	2.807456
O	1.005263	-1.97676	-1.10676
H	1.580012	-2.75544	-1.27515
H	1.599841	-1.15792	-1.16272

^{5}**IM1**$_{A,C5}$

C	-1.9963	1.931576	-4.73679
H	-2.97716	1.830816	-4.25501
C	-1.43551	3.342892	-4.53734
H	-0.51689	3.480268	-5.11806
H	-2.15496	4.081939	-4.9171
C	-1.11897	3.656735	-3.06169
H	-1.98646	3.437321	-2.42623
H	-0.29807	3.010815	-2.72322
C	-0.67955	5.104038	-2.90047
O	0.249198	5.576519	-3.57103
N	-1.38003	5.860798	-2.02157
H	-2.09923	5.458183	-1.43695
H	-1.14837	6.847445	-1.90417
C	-4.7818	9.034853	-2.39561
H	-5.71053	8.677892	-2.85656
C	-4.405	8.199845	-1.16814
H	-4.24543	7.156205	-1.47147
H	-3.43555	8.543741	-0.78553
C	-5.43058	8.229506	-0.07033
N	-5.27733	7.487087	1.094641
C	-6.34584	7.725352	1.831762
H	-6.55041	7.301053	2.80533
N	-7.18815	8.588143	1.208219
H	-8.07439	8.918083	1.561472
C	-6.61779	8.917993	-0.0089
H	-7.10007	9.590481	-0.7006
C	2.31041	-6.05273	4.331443
C	3.682026	-5.37708	4.275653
C	1.2603	-5.63838	3.280788
O	4.33355	-5.21584	5.304273
C	1.026178	-4.15582	3.188709
C	0.604927	-3.22042	4.10352
N	1.248276	-3.44657	2.02006
C	0.96169	-2.14734	2.248073
N	0.573666	-1.97495	3.510481
H	2.472397	-7.13761	4.262735
H	1.553494	-6.00051	2.29162
H	0.321948	-6.15032	3.532711
H	0.322959	-3.35614	5.135658
H	1.059962	-1.36603	1.507094
H	1.663475	-3.8828	1.168575
N	4.144745	-4.98185	3.04806
H	3.623341	-5.17397	2.192892
C	5.464676	-4.39004	2.918241
H	6.072068	-4.71962	3.765744

C	5.540498	-2.85333	2.87416
O	6.603253	-2.31411	2.559208
N	4.401516	-2.18634	3.173939
C	4.233473	-0.72902	3.110786
C	4.91472	-0.15971	1.847749
C	4.64892	0.001407	4.398388
O	5.603881	0.853384	1.862763
C	4.077494	1.424344	4.509739
C	2.552414	1.574382	4.627492
O	2.05531	2.698326	4.557651
O	1.882966	0.471406	4.846188
H	3.60115	-2.7402	3.448326
H	3.157876	-0.57516	2.976778
H	4.306675	-0.60442	5.244608
H	5.742033	0.048454	4.43977
H	4.400505	2.027528	3.656485
H	4.50128	1.915536	5.396746
N	4.664149	-0.88833	0.719266
H	3.836767	-1.47447	0.723329
C	4.937149	-0.3681	-0.61452
H	4.78604	-1.22429	-1.28666
C	3.947485	0.724484	-1.02884
H	3.966234	1.531941	-0.28688
H	4.249414	1.16562	-1.98139
C	2.510319	0.189226	-1.15978
O	1.704359	0.883024	-1.83445
O	2.243966	-0.91119	-0.5707
C	6.411216	0.005822	-0.80936
O	6.761447	0.81505	-1.67428
N	7.307965	-0.74277	-0.10682
H	6.975707	-1.26687	0.701129
C	8.739395	-0.47249	-0.20004
H	8.884534	0.613065	-0.24841
C	9.470832	-1.03015	1.024556
H	9.300905	-2.1077	1.128513
H	9.1104	-0.54414	1.93585
H	10.54301	-0.85111	0.913398
C	9.380107	-1.00044	-1.50299
O	10.54605	-0.73568	-1.77742
N	8.560386	-1.7456	-2.29732
H	7.624342	-1.92346	-1.95821
C	8.90744	-2.18768	-3.6318
H	9.998312	-2.11983	-3.70252
C	8.487265	-3.6611	-3.86646
H	9.071821	-4.28719	-3.18247
H	8.776999	-3.91252	-4.89009
C	7.005885	-3.91352	-3.66265

C	6.493295	-4.23094	-2.39439
H	7.178382	-4.34796	-1.55678
C	5.120172	-4.39995	-2.1913
H	4.734995	-4.6273	-1.20061
C	4.234341	-4.27377	-3.26468
H	3.167626	-4.40881	-3.10687
C	4.733978	-3.97702	-4.53559
H	4.053211	-3.88057	-5.37767
C	6.103992	-3.79264	-4.73177
H	6.486171	-3.53923	-5.71664
C	8.361774	-1.27978	-4.76531
O	8.500407	-1.63709	-5.93872
N	7.789105	-0.11604	-4.38263
H	7.682959	0.096529	-3.39348
C	7.322142	0.889682	-5.329
H	7.617127	0.547727	-6.32284
C	5.810854	1.159697	-5.24497
H	5.578231	1.488862	-4.22345
H	5.591223	2.01282	-5.90598
C	4.885947	-0.01476	-5.61802
H	5.102444	-0.84387	-4.93014
C	3.414059	0.385364	-5.42569
H	2.746145	-0.45192	-5.66245
H	3.195968	0.696493	-4.39725
H	3.145389	1.218461	-6.09019
C	5.130388	-0.51975	-7.04943
H	6.141628	-0.92099	-7.17307
H	4.421322	-1.31786	-7.30359
H	4.988702	0.289228	-7.78033
C	-5.47447	-3.38551	-5.29347
H	-4.76203	-4.09916	-5.72
C	-4.84434	-2.67234	-4.0857
H	-4.03476	-2.0161	-4.42582
H	-5.5882	-2.01901	-3.61423
C	-4.27434	-3.59478	-3.00277
O	-4.56232	-4.82178	-3.03157
O	-3.54314	-3.04125	-2.11792
C	-6.79964	-4.06802	-4.96991
O	-7.83619	-3.43336	-4.76756
N	-6.76504	-5.43608	-4.94672
H	-5.84935	-5.84622	-4.81103
C	-7.92869	-6.20438	-4.54678
H	-8.80623	-5.79319	-5.05091
C	-8.27223	-6.23679	-3.04647
O	-9.30676	-6.77779	-2.67416
N	-7.38335	-5.62925	-2.21197
H	-6.50738	-5.26696	-2.58507

C	-7.59864	-5.58296	-0.77292
H	-8.15349	-6.48287	-0.48933
C	-6.25829	-5.56906	-0.01696
H	-6.49182	-5.43674	1.042359
H	-5.6509	-4.71834	-0.34762
C	-5.50321	-6.88915	-0.17422
O	-5.87981	-7.91853	0.369807
N	-4.36279	-6.81907	-0.93883
H	-4.28371	-6.08296	-1.64984
H	-3.96212	-7.72051	-1.17206
C	-8.46942	-4.39896	-0.29084
O	-8.74995	-4.30081	0.902
N	-8.88626	-3.50783	-1.22671
H	-8.66712	-3.67126	-2.20226
C	-9.83609	-2.46288	-0.8693
H	-9.54724	-2.08091	0.114431
C	-9.89877	-1.33191	-1.90527
H	-10.1646	-1.75202	-2.88375
H	-10.7394	-0.68517	-1.61502
C	-8.63526	-0.50456	-2.04373
C	-7.97399	-0.40789	-3.27543
H	-8.3473	-0.96638	-4.12889
C	-6.81098	0.347486	-3.41707
H	-6.29824	0.414089	-4.37217
C	-6.26913	1.013865	-2.31394
O	-5.08712	1.681176	-2.49028
H	-4.7377	2.027509	-1.62482
C	-6.92811	0.950347	-1.07809
H	-6.52107	1.484502	-0.22318
C	-8.09909	0.200538	-0.95748
H	-8.59526	0.159695	0.010061
C	3.331464	-7.87154	0.068452
H	4.185548	-7.28429	0.418287
C	2.558572	-7.12358	-1.01822
H	3.2045	-6.96356	-1.89295
H	1.709646	-7.71385	-1.38193
C	2.024369	-5.73963	-0.62109
O	1.094516	-5.26637	-1.33515
O	2.563618	-5.14401	0.366548
C	-0.81001	-3.09253	9.43263
C	-0.2599	-2.23905	8.337636
C	-0.75058	-1.88724	7.10534
N	0.971028	-1.60355	8.414694
C	1.172034	-0.91568	7.259779
N	0.146103	-1.06677	6.445972
H	-0.95074	-2.52795	10.36371
H	-1.78472	-3.48974	9.136351

H	-1.68462	-2.16769	6.642078
H	2.05229	-0.33364	7.037331
H	1.618704	-1.65336	9.187988
C	0.261875	10.02125	0.26595
H	1.159591	10.63966	0.384194
C	0.141123	9.047807	1.464958
H	1.04628	8.430299	1.533616
H	0.108281	9.651582	2.382304
C	-1.09688	8.150613	1.375606
H	-1.97371	8.771657	1.145271
H	-0.98346	7.467616	0.529566
C	-1.41628	7.311543	2.620103
H	-0.57633	6.688622	2.936474
H	-1.67141	7.94982	3.474217
N	-2.58263	6.462637	2.341378
H	-3.42734	6.977735	2.091625
C	-2.50359	5.269399	1.705833
N	-1.41805	4.501274	1.757437
H	-0.66069	4.662921	2.446832
H	-1.5477	3.530345	1.466915
N	-3.5531	4.89373	0.950542
H	-4.36452	5.503504	0.938262
H	-3.63095	3.935302	0.586034
C	0.268119	9.307499	-1.0845
O	-0.76146	8.79097	-1.54351
N	1.458305	9.276181	-1.72562
H	2.300544	9.586382	-1.25211
C	1.724195	8.458175	-2.90027
H	0.871039	7.810411	-3.10344
C	3.008024	7.6673	-2.61633
O	3.934219	8.216146	-2.01113
N	3.014618	6.393507	-3.06331
H	2.131395	6.002514	-3.39388
C	4.107917	5.476039	-2.75179
H	4.969403	6.09449	-2.48867
C	3.772859	4.498036	-1.6096
H	2.970631	3.822468	-1.93087
H	4.653916	3.87078	-1.42872
C	3.372058	5.214264	-0.31549
H	4.178024	5.874841	0.022026
H	2.488927	5.84074	-0.48135
S	3.032745	4.103683	1.10706
C	1.502768	3.26975	0.545999
H	1.170476	2.63477	1.373221
H	0.726712	4.008509	0.323205
H	1.677468	2.63905	-0.32738
O	-1.47081	1.155432	5.287826

C	-2.35329	0.241872	5.13465
O	-2.04023	-0.82161	4.518533
C	-3.74955	0.441861	5.687397
C	-3.74582	0.927694	7.143723
H	-4.22091	1.203365	5.053558
H	-4.30281	-0.49585	5.57593
H	-3.20189	1.87244	7.229266
H	-4.77059	1.083826	7.498675
O	-0.02361	0.745359	2.703069
O	-3.25155	3.037321	3.861186
Fe	0.056632	-0.04415	4.33781
C	-2.56388	-1.07981	0.902541
H	-2.36406	-0.65631	1.887594
H	-1.58755	-1.22782	0.426588
C	-3.36748	-0.0723	0.076988
H	-4.44226	-0.2296	0.234324
C	-3.11239	1.425309	0.419984
O	-3.7475	2.281167	-0.26983
O	-2.35544	1.707849	1.381461
H	-0.8573	-3.48421	-0.60954
N	-3.12951	-0.30016	-1.39391
H	-2.09289	-0.19214	-1.67431
H	-3.6888	0.355764	-1.96039
H	-3.38669	-1.27463	-1.68034
C	-3.30394	-2.43433	1.090232
H	-4.12327	-2.26495	1.805633
C	-2.39814	-3.50673	1.604251
C	-1.98602	-4.68037	0.780675
H	-1.14309	-5.20159	1.239852
H	-2.79602	-5.40922	0.632026
N	-1.54673	-4.28223	-0.60875
H	-1.01154	-5.0372	-1.06061
H	-2.35468	-3.98336	-1.21078
H	-0.15732	-3.94632	9.656919
H	1.939678	-5.86203	5.342928
H	3.707162	-8.82973	-0.30955
H	2.69729	-8.08315	0.938038
H	5.940255	-4.74494	1.999355
H	7.85904	1.82693	-5.12874
H	-3.26287	0.200166	7.806878
H	-5.6971	-2.63692	-6.06201
H	-7.80862	-7.23869	-4.88376
H	-10.8402	-2.89673	-0.75116
H	-3.98717	8.978552	-3.14509
H	-4.91646	10.09107	-2.13255
H	-0.60403	10.69386	0.263674
H	1.892355	9.096837	-3.77854

H	4.359303	4.908101	-3.65507
H	-2.12873	1.715841	-5.80426
H	-1.3334	1.169003	-4.3127
H	-2.51789	2.747838	4.434419
H	-3.05673	2.57744	3.024381
O	-0.64868	-0.30258	-2.35431
H	-0.34933	-1.23601	-2.28695
H	0.174916	0.215642	-2.10527
O	0.79609	4.898806	3.439761
H	1.021117	4.105338	3.974189
H	1.556882	4.95333	2.825336
O	0.410836	-2.61082	-1.30339
H	0.893262	-3.46153	-1.45168
H	1.088505	-1.93391	-0.98719
H	-0.86089	0.968965	2.247393
H	-3.76741	-2.74775	0.148718
H	-1.97595	-3.4031	2.599268

^{5}**TS2**$_{A,C5}$

C	2.286915	-1.85574	-5.79517
H	3.059044	-1.07768	-5.86462
C	2.84814	-3.12474	-5.1437
H	2.090209	-3.9157	-5.13946
H	3.687458	-3.512	-5.73599
C	3.317503	-2.87692	-3.70625
H	4.021855	-2.0368	-3.67194
H	2.463861	-2.59017	-3.07643
C	3.962578	-4.09646	-3.06038
O	3.74235	-5.24678	-3.4703
N	4.793814	-3.84523	-2.02393
H	4.859278	-2.90863	-1.62978
H	5.183492	-4.6357	-1.51676
C	9.435595	-4.7033	-1.37548
H	10.07125	-3.96713	-1.88233
C	8.582501	-4.05692	-0.28009
H	7.924154	-3.30065	-0.72895
H	7.910805	-4.81641	0.137389
C	9.379367	-3.41144	0.817767
N	8.76674	-2.71654	1.854747
C	9.737602	-2.26548	2.625541
H	9.61233	-1.67412	3.522447
N	10.95445	-2.63418	2.148619
H	11.84995	-2.40709	2.555269
C	10.74146	-3.3651	0.992763
H	11.55828	-3.76804	0.415075
C	-6.16831	3.687588	4.960277
C	-6.57231	2.244547	4.646505
C	-5.06723	4.345801	4.099004
O	-6.94017	1.496163	5.546019
C	-3.82171	3.519573	3.961235
C	-2.78847	3.194747	4.811804
N	-3.55486	2.831879	2.792248
C	-2.40949	2.139232	2.958454
N	-1.91622	2.339212	4.175174
H	-7.07811	4.302662	4.906948
H	-5.44678	4.565952	3.095004
H	-4.82514	5.314071	4.553882
H	-2.61111	3.51962	5.826902
H	-1.95807	1.512909	2.20357
H	-4.14742	2.883395	1.934865
N	-6.53614	1.84084	3.334953
H	-6.31022	2.492531	2.589228
C	-7.04054	0.53232	2.9614
H	-7.74848	0.204298	3.726414
C	-6.03206	-0.61632	2.788901

O	-6.47253	-1.72849	2.485274
N	-4.72184	-0.33108	2.939136
C	-3.61834	-1.30085	2.821522
C	-3.85434	-2.26993	1.632132
C	-3.33522	-2.05012	4.136278
O	-3.82652	-3.48721	1.760609
C	-1.93819	-2.69115	4.194211
C	-0.74982	-1.71876	4.302005
O	0.373936	-2.11501	3.900362
O	-0.98122	-0.58278	4.839938
H	-4.48552	0.607793	3.234161
H	-2.73914	-0.69746	2.567867
H	-3.43547	-1.33317	4.956988
H	-4.09699	-2.82567	4.260735
H	-1.78066	-3.34902	3.335699
H	-1.88681	-3.33972	5.081085
N	-4.08411	-1.64108	0.44062
H	-3.83844	-0.65648	0.385927
C	-4.17242	-2.35275	-0.83551
H	-4.35799	-1.57165	-1.58252
C	-2.87356	-3.05726	-1.24781
H	-2.45403	-3.60342	-0.39797
H	-3.10914	-3.80861	-2.00797
C	-1.80359	-2.13459	-1.84406
O	-0.71366	-2.68787	-2.14773
O	-2.08093	-0.89884	-2.03672
C	-5.3805	-3.29984	-0.95724
O	-5.4505	-4.07783	-1.9184
N	-6.40229	-3.16648	-0.07446
H	-6.26926	-2.62111	0.777124
C	-7.51899	-4.11103	-0.10227
H	-7.11614	-5.10765	-0.31717
C	-8.24359	-4.13852	1.247332
H	-8.60406	-3.14276	1.523896
H	-7.5652	-4.47669	2.035788
H	-9.09343	-4.82192	1.177712
C	-8.52345	-3.86598	-1.24848
O	-9.42064	-4.67391	-1.46768
N	-8.33235	-2.73032	-1.97809
H	-7.5713	-2.12208	-1.70549
C	-9.05075	-2.43778	-3.20221
H	-9.93707	-3.08099	-3.19303
C	-9.51253	-0.95884	-3.25137
H	-10.2519	-0.81169	-2.45581
H	-10.0114	-0.81578	-4.21355
C	-8.37433	0.028373	-3.09051
C	-8.0032	0.500234	-1.82193

H	-8.58781	0.202685	-0.95338
C	-6.89496	1.335558	-1.65733
H	-6.61888	1.691721	-0.66853
C	-6.14147	1.721849	-2.76874
H	-5.26277	2.347371	-2.63721
C	-6.51946	1.285099	-4.04108
H	-5.9439	1.587047	-4.91247
C	-7.62446	0.447278	-4.20111
H	-7.90158	0.089074	-5.18855
C	-8.2785	-2.8112	-4.49623
O	-8.737	-2.46884	-5.59061
N	-7.14899	-3.53444	-4.33254
H	-6.81284	-3.7511	-3.39662
C	-6.35046	-4.03271	-5.4458
H	-6.93472	-3.87028	-6.35378
C	-4.95993	-3.38176	-5.53666
H	-4.44485	-3.54857	-4.58154
H	-4.38334	-3.9249	-6.30153
C	-4.94354	-1.87687	-5.86547
H	-5.55351	-1.36568	-5.10881
C	-3.51459	-1.32082	-5.76148
H	-3.49143	-0.25218	-6.01081
H	-3.10639	-1.43065	-4.75032
H	-2.83746	-1.83542	-6.45709
C	-5.55594	-1.57365	-7.24235
H	-6.61376	-1.85284	-7.28484
H	-5.48694	-0.50183	-7.46883
H	-5.0222	-2.11154	-8.03881
C	1.644689	5.167186	-5.67215
H	0.584037	5.381573	-5.83965
C	1.801336	4.101541	-4.57681
H	1.419584	3.139519	-4.93686
H	2.866932	3.951515	-4.362
C	1.095647	4.424243	-3.25538
O	0.710779	5.602183	-3.04105
O	0.962507	3.450846	-2.43815
C	2.422264	6.445548	-5.38133
O	3.654271	6.474851	-5.35391
N	1.66404	7.565522	-5.18171
H	0.692898	7.414065	-4.94438
C	2.271683	8.833662	-4.82464
H	3.113783	9.019475	-5.49595
C	2.829623	8.973932	-3.39678
O	3.439746	9.990092	-3.08641
N	2.621764	7.920642	-2.55949
H	2.062621	7.127499	-2.86901
C	3.170503	7.907421	-1.21197

H	3.162981	8.934661	-0.8355
C	2.31686	7.032201	-0.27907
H	2.833819	6.997439	0.683611
H	2.257319	6.013928	-0.68073
C	0.926125	7.62247	-0.04857
O	0.75437	8.646159	0.59571
N	-0.11262	6.887014	-0.5831
H	0.076252	6.325476	-1.42308
H	-1.00457	7.370336	-0.56512
C	4.64599	7.449109	-1.12053
O	5.218098	7.482735	-0.03281
N	5.238786	7.01437	-2.26174
H	4.730269	7.055772	-3.13803
C	6.666615	6.723927	-2.28133
H	6.915957	6.257513	-1.32392
C	7.081601	5.832704	-3.46013
H	6.777226	6.312004	-4.39923
H	8.181111	5.817627	-3.47098
C	6.550936	4.412315	-3.42978
C	5.771987	3.917065	-4.48414
H	5.505611	4.576667	-5.30524
C	5.293409	2.60762	-4.48003
H	4.693228	2.227834	-5.30224
C	5.572678	1.761558	-3.40133
O	5.027413	0.50769	-3.41699
H	5.150843	0.042571	-2.53985
C	6.361695	2.23291	-2.34277
H	6.585745	1.574092	-1.50768
C	6.843733	3.542386	-2.37026
H	7.455045	3.890737	-1.54014
C	-6.39336	5.848989	-0.08348
H	-7.18458	5.193286	0.290363
C	-5.37251	5.050934	-0.89523
H	-5.85749	4.61962	-1.78232
H	-4.56998	5.692717	-1.27684
C	-4.72296	3.881327	-0.14669
O	-3.61577	3.450411	-0.59565
O	-5.33452	3.388181	0.848603
C	-0.94321	3.175859	10.17893
C	-0.88605	2.365144	8.926065
C	-0.35264	2.615829	7.686727
N	-1.42659	1.090275	8.821176
C	-1.20593	0.633201	7.559533
N	-0.5573	1.535591	6.851832
H	-0.42596	2.684156	11.01348
H	-0.45917	4.142462	10.01404
H	0.168379	3.493714	7.33238

H	-1.52428	-0.32759	7.183776
H	-1.91388	0.589772	9.550422
C	6.087232	-8.09575	1.243827
H	5.789315	-9.14175	1.383151
C	5.482457	-7.22998	2.374559
H	4.386261	-7.25611	2.310891
H	5.748061	-7.69362	3.334752
C	5.969118	-5.77935	2.326147
H	7.068746	-5.76381	2.354129
H	5.686508	-5.35775	1.358843
C	5.435372	-4.85068	3.425899
H	4.352474	-4.92863	3.549447
H	5.877308	-5.08245	4.401508
N	5.781746	-3.45199	3.114762
H	6.784641	-3.28227	3.099918
C	5.151711	-2.79967	2.091503
N	3.886098	-3.05084	1.794794
H	3.253807	-3.51241	2.472624
H	3.438007	-2.47593	1.069935
N	5.868518	-1.89515	1.40199
H	6.883754	-1.96651	1.466651
H	5.473304	-1.45721	0.5655
C	5.711865	-7.61531	-0.15943
O	6.126634	-6.53938	-0.61114
N	4.917428	-8.4592	-0.862
H	4.456018	-9.22554	-0.3833
C	4.286668	-8.1056	-2.12414
H	4.541383	-7.08056	-2.39454
C	2.774709	-8.31246	-1.96132
O	2.36026	-9.25523	-1.2783
N	1.995537	-7.41534	-2.60174
H	2.453572	-6.60998	-3.03145
C	0.541033	-7.42337	-2.46903
H	0.261437	-8.43749	-2.1721
C	0.003618	-6.39971	-1.44899
H	0.168893	-5.3794	-1.81499
H	-1.08418	-6.5272	-1.38299
C	0.62389	-6.57517	-0.05886
H	0.449356	-7.58945	0.315602
H	1.708446	-6.42985	-0.10139
S	-0.05331	-5.45397	1.226527
C	0.567189	-3.83654	0.626375
H	0.393801	-3.11319	1.427414
H	1.640928	-3.89574	0.43036
H	0.054462	-3.51469	-0.28109
O	2.01892	1.301373	5.216539
C	2.192803	2.566449	5.099637

O	1.235975	3.335979	4.822168
C	3.601513	3.124733	5.273705
C	4.417667	2.418498	6.360975
H	4.111579	3.016741	4.304881
H	3.519176	4.199426	5.464876
H	4.48832	1.347174	6.154573
H	5.430745	2.833253	6.418804
O	0.243955	1.126277	2.749662
O	2.506657	-0.49281	3.125912
Fe	-0.08103	1.303619	4.69322
C	2.074406	1.275912	0.201848
H	2.381963	0.766415	1.117007
H	1.065731	0.914316	-0.01505
C	3.032921	0.886367	-0.92745
H	3.89597	1.558304	-0.94753
C	3.552924	-0.5705	-0.74762
O	4.772166	-0.78742	-1.09625
O	2.769425	-1.39871	-0.26173
H	-1.13331	2.219286	-0.19284
N	2.375085	1.004247	-2.27452
H	1.56309	0.303067	-2.44638
H	3.088533	0.834226	-3.00036
H	1.954935	1.950333	-2.42454
C	2.086175	2.817399	0.468016
H	3.063662	3.072383	0.893099
C	1.025034	3.307384	1.37751
C	-0.28807	3.823343	0.929498
H	-1.02601	3.722181	1.726609
H	-0.16847	4.907102	0.73234
N	-0.85428	3.227787	-0.31757
H	-1.7774	3.663416	-0.52918
H	-0.214	3.351038	-1.14584
H	-1.97552	3.371864	10.49773
H	-5.86636	3.671899	6.011348
H	-6.85272	6.633584	-0.6961
H	-5.92479	6.330763	0.782875
H	-7.58058	0.606849	2.011749
H	-6.22723	-5.11613	-5.31817
H	3.947628	2.53644	7.344308
H	2.051973	4.767081	-6.60795
H	1.543298	9.63473	-4.98152
H	7.236322	7.664136	-2.32764
H	8.787702	-5.16839	-2.12377
H	10.08396	-5.48743	-0.96518
H	7.181835	-8.05859	1.311079
H	4.644577	-8.77028	-2.92298
H	0.100908	-7.22297	-3.45284

H	1.930273	-2.0611	-6.81174
H	1.451875	-1.44475	-5.21628
H	2.604834	0.078278	3.915281
H	1.816028	-1.13614	3.412578
O	0.390322	-0.50653	-3.09573
H	-0.53054	-0.22513	-2.87513
H	0.273375	-1.45102	-2.78215
O	2.05062	-4.32526	3.547568
H	1.415481	-3.61006	3.786879
H	1.518087	-4.924	2.983749
O	-2.43301	0.974533	-0.11933
H	-3.08434	1.658444	-0.40396
H	-2.33218	0.306972	-0.85472
H	1.022379	0.538682	2.627094
H	1.979039	3.335123	-0.49526
H	1.27064	3.576986	2.402921

[5]**Pr**$_{A,C5}$

C	-0.29965	2.945358	-4.97702
H	-1.31963	2.565371	-5.12216
C	-0.30546	4.267329	-4.20365
H	0.712471	4.663969	-4.12657
H	-0.88654	5.021322	-4.75204
C	-0.87812	4.112208	-2.7889
H	-1.89053	3.690537	-2.82541
H	-0.26057	3.400856	-2.22159
C	-0.90951	5.422891	-2.0145
O	-0.07107	6.317318	-2.20906
N	-1.9027	5.560338	-1.10777
H	-2.54684	4.796861	-0.91057
H	-1.91493	6.396984	-0.5306
C	-5.4134	8.980307	-0.31534
H	-6.30099	8.866833	-0.94957
C	-5.20849	7.767871	0.597373
H	-5.07594	6.866644	-0.01716
H	-4.26448	7.891564	1.140952
C	-6.33297	7.529943	1.564422
N	-6.31975	6.458347	2.45006
C	-7.44764	6.5237	3.131367
H	-7.7646	5.833412	3.901334
N	-8.19541	7.587566	2.740632
H	-9.09994	7.84855	3.104808
C	-7.49706	8.238785	1.738838
H	-7.88702	9.118777	1.252231
C	3.629824	-7.30228	2.476803
C	4.66022	-6.19797	2.72417
C	2.454265	-7.00273	1.524424
O	5.231311	-6.12014	3.809324
C	1.674473	-5.76858	1.87772
C	0.87233	-5.42938	2.943849
N	1.693771	-4.65101	1.064841
C	0.92804	-3.70304	1.637685
N	0.411322	-4.13887	2.785167
H	4.176381	-8.17893	2.100515
H	2.814148	-6.89666	0.496316
H	1.790758	-7.87685	1.530653
H	0.598488	-6.01748	3.807873
H	0.750809	-2.73165	1.202335
H	2.284057	-4.58459	0.188534
N	4.941173	-5.3449	1.689602
H	4.494878	-5.43272	0.775401
C	6.007842	-4.36962	1.822577
H	6.71119	-4.72445	2.579938

C	5.616934	-2.93563	2.214957
O	6.498968	-2.07809	2.296655
N	4.29947	-2.69926	2.413999
C	3.711188	-1.3788	2.669376
C	4.401164	-0.29711	1.806851
C	3.670983	-0.99436	4.157785
O	4.80185	0.762305	2.2736
C	2.682465	0.141033	4.472788
C	1.19158	-0.16326	4.270769
O	0.39644	0.805097	4.200515
O	0.850011	-1.39718	4.210476
H	3.671631	-3.48603	2.310466
H	2.678265	-1.45406	2.309344
H	3.404457	-1.89381	4.721668
H	4.676179	-0.68897	4.46443
H	2.936217	1.031766	3.892166
H	2.79268	0.432254	5.527782
N	4.510973	-0.64211	0.489604
H	3.820181	-1.30195	0.142778
C	4.820035	0.35367	-0.53117
H	4.954196	-0.2247	-1.45595
C	3.671728	1.340623	-0.76119
H	3.477954	1.887086	0.169228
H	3.96515	2.083909	-1.5055
C	2.370547	0.657515	-1.22395
O	1.533693	1.39632	-1.81498
O	2.226946	-0.57817	-0.96685
C	6.180079	1.025866	-0.30832
O	6.446673	2.119232	-0.82022
N	7.123526	0.270089	0.319537
H	6.822805	-0.53387	0.868683
C	8.468811	0.789938	0.543149
H	8.390768	1.848845	0.815526
C	9.162789	0.02255	1.672083
H	9.203097	-1.05037	1.453667
H	8.617349	0.151904	2.611337
H	10.18245	0.398932	1.78553
C	9.341811	0.790019	-0.73398
O	10.44651	1.323216	-0.73792
N	8.783718	0.179021	-1.81614
H	7.877163	-0.24989	-1.68442
C	9.330242	0.219167	-3.15622
H	10.40286	0.415444	-3.05411
C	9.13669	-1.14291	-3.87325
H	9.771324	-1.88158	-3.37035
H	9.504466	-1.01568	-4.895
C	7.696525	-1.61806	-3.87591

C	7.208947	-2.43993	-2.8472
H	7.894644	-2.79268	-2.079
C	5.860482	-2.80541	-2.7906
H	5.493747	-3.43081	-1.98054
C	4.974032	-2.36366	-3.77691
H	3.924493	-2.64056	-3.72482
C	5.453195	-1.56699	-4.82097
H	4.773411	-1.22482	-5.59709
C	6.798637	-1.19562	-4.86899
H	7.161486	-0.56066	-5.67276
C	8.786553	1.378004	-4.03547
O	9.120965	1.434985	-5.22245
N	7.994898	2.284182	-3.41935
H	7.721222	2.144022	-2.44974
C	7.504044	3.489491	-4.07597
H	7.976585	3.527144	-5.05952
C	5.971429	3.555221	-4.17813
H	5.556881	3.492797	-3.16309
H	5.70873	4.55273	-4.56438
C	5.303363	2.48331	-5.06027
H	5.562112	1.500479	-4.6432
C	3.773809	2.626466	-5.00621
H	3.285302	1.860567	-5.62093
H	3.383852	2.523403	-3.98673
H	3.45761	3.606801	-5.38967
C	5.806901	2.523253	-6.51207
H	6.877464	2.30262	-6.57706
H	5.276583	1.783906	-7.12579
H	5.629692	3.510068	-6.96318
C	-4.66337	-2.78538	-6.06582
H	-3.86083	-3.45713	-6.38526
C	-4.22927	-2.00069	-4.81953
H	-3.36961	-1.36409	-5.06601
H	-5.03401	-1.3288	-4.50247
C	-3.83889	-2.86979	-3.61836
O	-3.89976	-4.12364	-3.74049
O	-3.48938	-2.25312	-2.5605
C	-5.96352	-3.55135	-5.84511
O	-7.0373	-2.98331	-5.63709
N	-5.86388	-4.91441	-5.91067
H	-4.93426	-5.29801	-5.80282
C	-6.99865	-5.76593	-5.60556
H	-7.8773	-5.37353	-6.12299
C	-7.40203	-5.90366	-4.12506
O	-8.41143	-6.53313	-3.8311
N	-6.5958	-5.28279	-3.22074
H	-5.7415	-4.82281	-3.53227

C	-6.90208	-5.28775	-1.79843
H	-7.381	-6.24127	-1.55732
C	-5.6201	-5.14156	-0.9566
H	-5.94066	-5.07355	0.086815
H	-5.11135	-4.20988	-1.2281
C	-4.68666	-6.34717	-1.08074
O	-4.96968	-7.43701	-0.60819
N	-3.48581	-6.09308	-1.72332
H	-3.50611	-5.37341	-2.46095
H	-2.976	-6.9401	-1.95501
C	-7.90508	-4.19612	-1.35301
O	-8.30751	-4.18639	-0.19127
N	-8.27901	-3.27262	-2.2748
H	-7.96333	-3.36312	-3.23371
C	-9.31785	-2.30193	-1.95202
H	-9.11977	-1.92778	-0.94277
C	-9.40041	-1.14992	-2.96354
H	-9.52855	-1.56463	-3.9716
H	-10.33	-0.60607	-2.74123
C	-8.23356	-0.17969	-2.96617
C	-7.4948	0.049154	-4.13491
H	-7.72737	-0.5178	-5.03192
C	-6.4332	0.952774	-4.15835
H	-5.86272	1.12262	-5.06722
C	-6.06993	1.642921	-2.99629
O	-4.97922	2.463939	-3.05573
H	-4.70473	2.769517	-2.14316
C	-6.81087	1.443892	-1.82201
H	-6.54075	1.987902	-0.9206
C	-7.87888	0.545187	-1.81984
H	-8.43942	0.40392	-0.89781
C	4.577361	-7.04887	-2.45185
H	5.320923	-6.6368	-1.76324
C	3.735053	-5.93544	-3.07622
H	4.379006	-5.27447	-3.67235
H	2.993799	-6.34007	-3.7742
C	2.983007	-5.03908	-2.07871
O	1.976652	-4.43022	-2.50791
O	3.447052	-4.95998	-0.88449
C	0.646894	-6.54132	8.441942
C	0.656163	-5.37636	7.507636
C	-0.227	-4.95769	6.543699
N	1.677987	-4.4395	7.474046
C	1.385635	-3.52131	6.514789
N	0.23784	-3.80817	5.935188
H	0.64765	-6.22674	9.493857
H	-0.25308	-7.13986	8.276125

H	-1.16615	-5.39764	6.241266
H	2.015991	-2.68172	6.266735
H	2.517528	-4.45477	8.035355
C	-1.10978	9.402791	2.800423
H	-0.31373	10.08645	3.118116
C	-1.17532	8.199712	3.770863
H	-0.2373	7.631064	3.714717
H	-1.23751	8.594566	4.794436
C	-2.36108	7.275335	3.48256
H	-3.28952	7.865176	3.48675
H	-2.25524	6.888321	2.466265
C	-2.53607	6.084107	4.434802
H	-1.61052	5.518456	4.568105
H	-2.85105	6.406089	5.433676
N	-3.58038	5.178249	3.918806
H	-4.49242	5.62462	3.858718
C	-3.32635	4.420612	2.806316
N	-2.13501	3.886697	2.600921
H	-1.40817	3.819006	3.335142
H	-1.9977	3.323146	1.749224
N	-4.33813	4.226326	1.942093
H	-5.12197	4.878912	1.995449
H	-4.1665	3.761036	1.046306
C	-0.9155	9.00085	1.336492
O	-1.7844	8.378216	0.710932
N	0.258168	9.39437	0.786116
H	1.003569	9.735529	1.384152
C	0.726125	8.95517	-0.51909
H	0.025232	8.235534	-0.94367
C	2.128089	8.358905	-0.32726
O	2.889327	8.849657	0.512834
N	2.417571	7.30653	-1.12161
H	1.650626	6.897076	-1.65855
C	3.634327	6.517516	-0.94989
H	4.345702	7.143012	-0.40487
C	3.379132	5.19461	-0.20289
H	2.687118	4.581114	-0.79302
H	4.319993	4.634043	-0.14385
C	2.811014	5.413631	1.203082
H	3.531034	5.94972	1.830612
H	1.900916	6.02057	1.162007
S	2.424562	3.868733	2.115577
C	1.005805	3.233477	1.14773
H	0.586088	2.402509	1.719394
H	0.239801	4.004771	1.031939
H	1.302215	2.860608	0.16562
O	-2.3548	-1.86599	5.418105

C	-2.95361	-2.95849	5.20663
O	-2.42048	-3.86959	4.484832
C	-4.32012	-3.23436	5.819415
C	-4.95159	-2.03733	6.529592
H	-4.97002	-3.61436	5.021184
H	-4.19204	-4.07639	6.513867
H	-5.11659	-1.20796	5.833933
H	-5.91861	-2.31298	6.965832
O	-1.57747	-1.91047	2.214974
O	-2.1946	0.232737	3.598531
Fe	-0.6101	-2.80094	4.129663
C	-2.50142	-0.38945	-0.00734
H	-1.82747	0.135473	0.66938
H	-1.86005	-0.97397	-0.66966
C	-3.25321	0.666482	-0.83136
H	-4.33592	0.53581	-0.73828
C	-2.94184	2.127051	-0.3903
O	-3.79275	3.026893	-0.73777
O	-1.93312	2.327811	0.304203
H	-0.9825	-3.09623	-0.68137
N	-2.9529	0.468634	-2.29011
H	-1.89119	0.419581	-2.49714
H	-3.43214	1.179606	-2.86121
H	-3.2956	-0.48696	-2.55185
C	-3.46615	-1.2791	0.789483
H	-4.01373	-0.64044	1.493352
C	-2.77588	-2.39265	1.59674
C	-2.48884	-3.67785	0.814625
H	-1.79468	-4.29634	1.384684
H	-3.42544	-4.22812	0.704443
N	-1.93904	-3.54208	-0.58011
H	-1.88795	-4.49047	-0.97442
H	-2.58159	-3.03056	-1.23909
H	1.51442	-7.19664	8.290137
H	3.257819	-7.57301	3.469302
H	5.105102	-7.62112	-3.2245
H	3.954497	-7.75123	-1.88419
H	6.544845	-4.28606	0.872161
H	7.856886	4.359652	-3.50565
H	-4.30159	-1.66861	7.328614
H	-4.85084	-2.07689	-6.88127
H	-6.80749	-6.77028	-5.99543
H	-10.2931	-2.80854	-1.90738
H	-4.54315	9.106184	-0.9656
H	-5.52776	9.902581	0.267605
H	-2.05445	9.958559	2.85611
H	0.798325	9.812033	-1.20362

H	4.058264	6.301046	-1.93746
H	0.148442	3.074479	-5.96952
H	0.266194	2.17266	-4.44635
H	-2.4446	-0.31105	4.375255
H	-1.27213	0.525672	3.823915
O	-0.42669	-0.02108	-2.9026
H	-0.22062	-0.94435	-2.64455
H	0.303049	0.525646	-2.46889
O	0.026671	3.607848	4.401375
H	0.203813	2.64166	4.464477
H	0.799222	3.945774	3.903025
O	0.393854	-2.43704	-1.46374
H	0.986895	-3.14264	-1.84464
H	1.017141	-1.69313	-1.21191
H	-1.80182	-1.01655	2.635769
H	-4.21664	-1.7307	0.125984
H	-3.46555	-2.72786	2.383323

Model 2 structures:

^{5}**Re**$_{B}$

C	-2.58559	-3.25979	5.185932
H	-2.14366	-2.80753	4.289227
C	-1.47472	-3.64232	6.169501
H	-1.90848	-3.89286	7.144703
H	-0.98137	-4.54754	5.809683
C	-0.42981	-2.53403	6.351205
H	-0.88527	-1.62438	6.764461
H	0.324286	-2.86042	7.080538
C	0.299166	-2.19615	5.048072
O	0.39928	-3.0381	4.141597
N	0.834488	-0.96462	4.950951
H	0.658724	-0.27725	5.669806
H	1.182593	-0.64191	4.030268
C	-3.4122	-4.4514	4.706963
O	-3.05918	-5.62331	4.897382
N	-4.54365	-4.13569	4.02953
H	-4.76406	-3.15585	3.870711
C	-5.37489	-5.16416	3.414785
H	-6.41142	-4.8069	3.412479
C	-4.93977	-5.55327	1.991375
H	-3.86168	-5.75778	1.999445
H	-5.43409	-6.50193	1.735821
C	-5.28638	-4.51641	0.91425
H	-4.85494	-3.53594	1.148134
H	-6.37758	-4.38684	0.888607
C	-4.80629	-4.95914	-0.47943
H	-3.7562	-4.70133	-0.63218
H	-4.88596	-6.05192	-0.56477
N	-5.59606	-4.37826	-1.56608
H	-6.52651	-4.77661	-1.71388
C	-5.12198	-3.66295	-2.60428
N	-3.95221	-3.0263	-2.56445
H	-3.57788	-2.62542	-1.67946
H	-3.6318	-2.62148	-3.45135
N	-5.86662	-3.60947	-3.72707
H	-6.6859	-4.20118	-3.79082
H	-5.74242	-2.81897	-4.36689
C	0.439325	-8.17975	0.118102
H	1.46922	-8.52255	0.279081
H	0.45228	-7.50367	-0.74603
C	-0.10602	-7.49813	1.334408
N	0.464764	-6.34427	1.844042
C	-0.25529	-6.02476	2.910542
H	-0.09871	-5.15701	3.539653

N	-1.25722	-6.91063	3.118024
H	-1.98192	-6.78211	3.82471
C	-1.17945	-7.85687	2.116152
H	-1.86465	-8.6892	2.05625
C	6.037314	5.189865	-5.05979
C	5.880519	5.612637	-3.59871
C	4.937347	4.274899	-5.64726
O	6.810982	5.463478	-2.79972
C	4.686021	3.032684	-4.84316
C	5.391248	1.866234	-4.65917
N	3.582024	2.933603	-4.02206
C	3.638137	1.760218	-3.3805
N	4.724438	1.075478	-3.74241
H	6.10387	6.098182	-5.67376
H	3.994175	4.82535	-5.74462
H	5.237996	4.008444	-6.66759
H	6.324029	1.55229	-5.10287
H	2.910359	1.440198	-2.65422
H	2.880114	3.712129	-3.84163
N	4.720164	6.231325	-3.23948
H	3.853739	6.062583	-3.74562
C	4.564032	6.791665	-1.90196
H	5.539907	7.172555	-1.58728
C	4.029688	5.799858	-0.85382
O	2.96499	6.034021	-0.27549
N	4.820715	4.722513	-0.65243
C	4.49987	3.468274	0.049574
C	3.429184	3.628508	1.162522
C	5.804167	2.921494	0.658245
O	3.746296	3.867167	2.326577
C	5.727953	1.495469	1.217424
C	5.71261	0.339399	0.219494
O	5.671264	-0.83517	0.646031
O	5.811054	0.64948	-1.02883
H	5.678091	4.708416	-1.2033
H	4.115411	2.757722	-0.6926
H	6.589563	2.963468	-0.10498
H	6.085039	3.596447	1.471936
H	4.860309	1.372769	1.876478
H	6.601384	1.312858	1.856021
N	2.154285	3.377891	0.773956
H	1.922739	3.331432	-0.21908
C	1.045321	3.316715	1.722284
H	0.138825	3.280445	1.107397
C	1.089651	2.042333	2.565953
H	2.016937	2.011312	3.143432
H	0.269647	2.077159	3.292779

C	0.954881	0.753523	1.753334
O	0.38615	0.779988	0.631617
O	1.420403	-0.29756	2.310884
C	0.915388	4.56247	2.625163
O	0.458857	4.468518	3.769767
N	1.197671	5.76211	2.049999
H	1.749283	5.754767	1.19141
C	1.28843	6.971415	2.872347
H	1.767621	6.700976	3.822057
C	2.135805	8.040106	2.175563
H	3.159263	7.683387	2.032217
H	2.144895	8.939287	2.795147
H	1.722815	8.292445	1.192778
C	-0.06706	7.570041	3.304872
O	-0.08873	8.559098	4.03039
N	-1.19351	6.931488	2.875268
H	-1.08362	6.103958	2.305223
C	-2.50456	7.232679	3.421458
H	-2.4622	8.274402	3.75521
C	-3.61725	7.107185	2.353502
H	-3.41702	7.84709	1.570227
H	-4.55396	7.381448	2.8465
C	-3.73036	5.723492	1.746439
C	-3.03993	5.389557	0.571359
H	-2.45813	6.153812	0.059463
C	-3.09585	4.094297	0.049019
H	-2.55764	3.849651	-0.86316
C	-3.84993	3.110204	0.690927
H	-3.87029	2.102299	0.288826
C	-4.56195	3.437099	1.847846
H	-5.16525	2.681452	2.34449
C	-4.5024	4.730144	2.370243
H	-5.04624	4.979169	3.277084
C	-2.87274	6.425462	4.69411
O	-3.9886	6.579182	5.198215
N	-1.91256	5.616468	5.198496
H	-1.04085	5.484921	4.690028
C	-2.05484	4.925894	6.472609
H	-3.02766	5.211249	6.877201
C	-1.90743	3.400965	6.362616
H	-0.9113	3.17953	5.956122
H	-1.92858	2.991964	7.385064
C	-2.96339	2.679058	5.504766
H	-2.89434	3.078491	4.484286
C	-2.64901	1.176235	5.440902
H	-3.34466	0.648884	4.776764
H	-1.63705	0.991573	5.059524

H	-2.71857	0.719459	6.439591
C	-4.39639	2.916407	6.006199
H	-5.11844	2.35243	5.401134
H	-4.50908	2.590294	7.050261
H	-4.67539	3.973303	5.948908
C	-6.46405	1.76067	-2.88143
H	-6.09436	2.261718	-3.78403
C	-5.40309	0.744842	-2.41595
H	-5.51982	0.522137	-1.35291
H	-5.50417	-0.2025	-2.95917
C	-3.95803	1.205631	-2.66342
O	-3.70494	1.850556	-3.7336
O	-3.09766	0.879663	-1.80538
C	-7.83036	1.16451	-3.18497
O	-8.86283	1.528661	-2.6143
N	-7.85732	0.197192	-4.1564
H	-6.98806	-0.17584	-4.54369
C	-9.11045	-0.29536	-4.69788
H	-9.8005	0.541675	-4.85294
C	-9.88346	-1.31402	-3.85461
O	-10.9069	-1.83707	-4.29859
N	-9.39016	-1.56772	-2.61643
H	-8.51267	-1.13546	-2.36083
C	-10.1253	-2.32316	-1.61452
H	-11.1183	-2.49248	-2.04475
C	-9.45414	-3.6692	-1.30749
H	-10.0825	-4.21522	-0.59547
H	-8.49158	-3.50496	-0.81331
C	-9.18831	-4.54854	-2.52273
O	-8.16635	-5.25127	-2.58771
N	-10.1256	-4.58575	-3.49898
H	-10.7595	-3.80549	-3.66325
H	-9.89301	-5.15007	-4.30722
C	-10.3752	-1.53248	-0.30603
O	-10.7178	-2.14072	0.702557
N	-10.233	-0.18623	-0.39318
H	-9.97865	0.242327	-1.27893
C	-10.5374	0.713423	0.71314
H	-10.8623	0.090835	1.5495
C	-9.33927	1.602771	1.11057
H	-8.94108	2.070624	0.201495
H	-9.72887	2.416082	1.735439
C	-8.2412	0.879272	1.85997
C	-7.19094	0.23382	1.190957
H	-7.1589	0.269903	0.105909
C	-6.17327	-0.43035	1.876741
H	-5.33974	-0.8893	1.350566

C	-6.21014	-0.4617	3.2723
O	-5.19614	-1.13233	3.931568
H	-5.23249	-0.90201	4.87384
C	-7.24432	0.168258	3.967113
H	-7.26858	0.143179	5.054949
C	-8.24442	0.834531	3.259356
H	-9.0376	1.332869	3.811703
C	0.229003	5.726548	-2.12156
H	0.751835	6.367237	-1.40281
H	-0.61782	5.257682	-1.61399
C	1.220443	4.683211	-2.61812
O	1.990561	5.021003	-3.57061
O	1.254826	3.544717	-2.05407
C	10.33676	-0.62622	-5.28683
C	9.271618	-0.45006	-4.25561
C	7.914677	-0.62597	-4.29462
N	9.532071	-0.03542	-2.95582
C	8.368029	0.032739	-2.27021
N	7.365058	-0.31609	-3.06254
H	11.09027	-1.3603	-4.97455
H	9.893271	-0.98399	-6.21985
H	7.297848	-0.98164	-5.10487
H	8.313246	0.250112	-1.21278
H	10.44164	0.15976	-2.56151
C	7.559132	-6.58598	-0.97826
H	8.342815	-6.51762	-1.7431
C	6.597762	-5.38378	-1.11973
H	7.141957	-4.46048	-0.87268
H	6.285729	-5.28128	-2.1645
C	5.349274	-5.49033	-0.23165
H	4.749337	-6.35149	-0.56086
H	5.626989	-5.68232	0.811329
C	4.512531	-4.21343	-0.32398
H	5.09716	-3.36969	0.052475
H	4.264472	-4.00432	-1.36958
N	3.285335	-4.32754	0.471472
H	2.823799	-5.22917	0.499635
C	2.608678	-3.30836	1.023553
N	3.097435	-2.08295	1.117405
H	4.026951	-1.81688	0.796995
H	2.481083	-1.34525	1.511006
N	1.344626	-3.54522	1.480499
H	1.065127	-4.53492	1.483977
H	1.077833	-3.0588	2.346102
C	8.181554	-6.63224	0.410049
O	7.703653	-7.2733	1.33991
N	9.31167	-5.85677	0.555627

H	9.52355	-5.17887	-0.1673
C	9.792671	-5.49449	1.869835
H	9.180677	-6.03054	2.601333
C	9.728849	-3.97355	2.054541
O	9.797834	-3.22896	1.069882
N	9.646208	-3.54257	3.332773
H	9.531976	-4.24199	4.053844
C	9.647236	-2.13332	3.735905
H	10.09484	-1.56622	2.918068
C	8.254437	-1.56067	4.041348
H	8.379397	-0.5	4.289681
H	7.654015	-1.59574	3.127714
C	7.530987	-2.26512	5.192625
H	7.341097	-3.32007	4.956562
H	8.143874	-2.24059	6.102733
S	5.946361	-1.48503	5.689459
C	4.891909	-1.86844	4.24471
H	3.899144	-1.47087	4.471767
H	5.252998	-1.39688	3.327345
H	4.807538	-2.95031	4.099024
O	3.857671	-0.83088	-1.95557
O	8.590985	-0.67571	0.773894
Fe	5.35598	-0.52585	-2.52448
C	-2.36006	0.102942	-7.00965
H	-2.31698	0.450629	-8.0508
H	-1.94508	-0.91015	-6.99701
C	-3.8431	-0.05314	-6.63797
H	-4.39598	-0.32871	-7.54394
C	-4.23014	-1.12348	-5.57208
O	-3.35587	-1.90993	-5.18247
O	-5.46294	-1.08334	-5.23791
N	-4.48556	1.229074	-6.15257
H	-4.15863	1.505088	-5.13651
H	-5.48794	1.034017	-6.07097
H	-4.33818	2.015424	-6.78995
C	-1.48164	1.043543	-6.16334
H	-1.97804	2.019947	-6.06176
H	-0.57115	1.24454	-6.74299
C	-1.08904	0.538917	-4.76776
H	-0.37234	-0.28656	-4.84433
H	-1.96327	0.144892	-4.24866
C	-0.49656	1.68473	-3.94775
H	0.496565	1.975103	-4.29989
H	-1.15151	2.557471	-3.99713
N	-0.37164	1.333832	-2.50357
H	10.83736	-5.80885	2.010697
H	10.30064	-2.04062	4.612382

H	-0.16598	-9.05244	-0.14701
H	7.019851	-7.52574	-1.13117
H	-5.32355	-6.04294	4.062454
H	-3.24845	-2.49832	5.617221
H	10.8579	0.315043	-5.50371
H	7.005039	4.685026	-5.11264
H	-0.12029	6.352893	-2.94719
H	3.851149	7.616494	-1.94502
H	-1.28842	5.302685	7.164129
H	-6.61211	2.535602	-2.12561
H	-8.91888	-0.75346	-5.67172
H	-11.3771	1.357329	0.420343
H	9.092772	-1.51523	0.797483
H	7.658319	-0.95624	0.822449
C	4.533574	-2.91054	-3.9775
O	4.581647	-4.10397	-3.72175
O	5.480592	-2.06785	-3.55814
C	3.424693	-2.31583	-4.82677
H	3.771685	-1.44165	-5.38417
H	2.604295	-1.99908	-4.17169
H	3.041022	-3.07936	-5.50788
H	0.099316	2.120343	-2.01557
H	-1.32555	1.176428	-2.1089
H	0.209598	0.463495	-2.38797
O	1.058714	-1.03211	-2.452
H	1.993856	-0.95472	-2.17472
H	0.588102	-1.45345	-1.68556
O	-3.10488	-1.59825	-0.34009
H	-3.18745	-0.7038	-0.7383
H	-2.13104	-1.69014	-0.18506
O	-0.32296	-1.66094	-0.19426
H	0.119553	-2.32888	0.371149
H	-0.04849	-0.76781	0.187516

[5]**TS1**$_{B,C3}$

C	-5.46435	1.576448	4.879669
H	-4.57762	1.611336	4.23431
C	-5.02781	1.485396	6.347984
H	-5.82305	1.866803	6.998654
H	-4.88823	0.434485	6.610554
C	-3.73262	2.254514	6.635273
H	-3.80549	3.295891	6.2925
H	-3.56016	2.303055	7.719217
C	-2.49494	1.612145	6.000888
O	-2.54829	0.498044	5.458537
N	-1.33896	2.298497	6.10108
H	-1.34077	3.255345	6.425347
H	-0.54088	1.987619	5.535725
C	-6.29322	0.382373	4.399848
O	-6.49918	-0.61651	5.099793
N	-6.74564	0.480981	3.123378
H	-6.47534	1.290732	2.571568
C	-7.2481	-0.67665	2.382181
H	-7.7245	-0.29771	1.473458
C	-6.1389	-1.69052	2.033775
H	-5.79942	-2.15883	2.964571
H	-6.58806	-2.48567	1.425701
C	-4.93608	-1.055	1.320886
H	-4.66245	-0.13579	1.852464
H	-5.18674	-0.75268	0.298571
C	-3.67076	-1.91699	1.30018
H	-2.81172	-1.28606	1.07636
H	-3.50151	-2.35385	2.294039
N	-3.68447	-3.01253	0.322445
H	-4.3016	-3.81137	0.487003
C	-2.72339	-3.22159	-0.59296
N	-1.88571	-2.24092	-0.9695
H	-2.24281	-1.26465	-1.02826
H	-1.17901	-2.51823	-1.64834
N	-2.56161	-4.45241	-1.10808
H	-3.20411	-5.18616	-0.82874
H	-1.96607	-4.53853	-1.93743
C	-2.92785	-6.01233	5.532379
H	-2.11157	-6.01967	6.26458
H	-2.49555	-6.28361	4.56
C	-3.59919	-4.67512	5.482667
N	-2.89657	-3.51285	5.20883
C	-3.7902	-2.53212	5.253132
H	-3.58537	-1.47683	5.12995
N	-5.02663	-3.00035	5.536677

H	-5.84728	-2.39741	5.564434
C	-4.92516	-4.36778	5.683028
H	-5.7788	-4.98873	5.909366
C	8.105048	2.104204	-3.99951
C	7.751211	2.824389	-2.69634
C	6.90554	1.591601	-4.83659
O	8.199815	2.431845	-1.61416
C	5.942422	0.765367	-4.03851
C	5.927352	-0.55181	-3.64042
N	4.846259	1.349988	-3.43458
C	4.222841	0.408605	-2.71098
N	4.842451	-0.76519	-2.81355
H	8.705921	2.778314	-4.62369
H	6.360111	2.433417	-5.27783
H	7.302265	1.001883	-5.67065
H	6.621628	-1.34424	-3.87471
H	3.33112	0.589245	-2.13451
H	4.536594	2.352659	-3.61518
N	6.98147	3.943228	-2.80182
H	6.317421	4.051936	-3.57327
C	6.658297	4.722829	-1.61355
H	7.528609	4.703876	-0.95179
C	5.416323	4.221995	-0.86331
O	4.376	4.886602	-0.83816
N	5.553016	3.01951	-0.25789
C	4.455803	2.338526	0.430219
C	3.80223	3.301348	1.457945
C	5.027971	1.094318	1.138361
O	4.45401	3.777259	2.380691
C	3.979132	0.109375	1.698536
C	3.324415	-0.79713	0.651779
O	2.082861	-0.80924	0.500773
O	4.136869	-1.54643	-0.01793
H	6.455546	2.556854	-0.34404
H	3.703006	2.045505	-0.31242
H	5.678026	0.551415	0.442056
H	5.650622	1.454956	1.963954
H	3.194427	0.636579	2.250062
H	4.495132	-0.55612	2.401953
N	2.48474	3.560575	1.242076
H	2.00699	3.073161	0.486563
C	1.678995	4.415844	2.110879
H	0.700289	4.482247	1.622017
C	1.412551	3.77876	3.480445
H	2.340355	3.436871	3.942585
H	0.974422	4.544364	4.130379
C	0.426569	2.61893	3.329464

O	-0.52152	2.784551	2.512161
O	0.62541	1.582196	4.041637
C	2.150988	5.873501	2.234234
O	1.73317	6.573701	3.164524
N	2.894105	6.39119	1.224803
H	3.316482	5.768846	0.535464
C	3.407176	7.756236	1.341979
H	3.804249	7.892088	2.353784
C	4.522029	8.01442	0.323593
H	5.366613	7.342149	0.500698
H	4.86016	9.04867	0.425567
H	4.168176	7.856505	-0.70084
C	2.323307	8.846998	1.221823
O	2.563827	9.989911	1.594332
N	1.133303	8.462551	0.676316
H	1.042407	7.506532	0.357645
C	-0.01454	9.346035	0.591448
H	0.361755	10.33521	0.875803
C	-0.58195	9.442052	-0.84549
H	0.175355	9.932929	-1.46685
H	-1.45524	10.09786	-0.79494
C	-0.95916	8.109896	-1.46182
C	-0.04481	7.408295	-2.26093
H	0.934894	7.841092	-2.45303
C	-0.37116	6.172822	-2.82724
H	0.366166	5.657242	-3.43803
C	-1.63331	5.62024	-2.59871
H	-1.9039	4.663383	-3.04058
C	-2.55606	6.30639	-1.80384
H	-3.54169	5.882814	-1.62661
C	-2.22321	7.539029	-1.23943
H	-2.93957	8.070995	-0.61987
C	-1.13207	9.018168	1.612073
O	-2.24535	9.535199	1.485351
N	-0.78972	8.201153	2.636393
H	0.155536	7.829452	2.694142
C	-1.6843	7.927863	3.754233
H	-2.48323	8.671454	3.713942
C	-2.25579	6.499749	3.7667
H	-1.41725	5.789865	3.780476
H	-2.79207	6.368999	4.720978
C	-3.20219	6.126161	2.608641
H	-2.64655	6.253224	1.669013
C	-3.59956	4.645679	2.721972
H	-4.24052	4.336456	1.886506
H	-2.71759	3.995306	2.730401
H	-4.16283	4.469234	3.651401

C	-4.44373	7.028979	2.547704
H	-5.12114	6.698379	1.749538
H	-5.00416	6.99134	3.493232
H	-4.17537	8.069489	2.341875
C	-4.24124	-0.7638	-5.74887
H	-3.49194	-1.01338	-6.50838
C	-3.51209	-0.48809	-4.42084
H	-4.1744	0.001429	-3.70297
H	-3.18652	-1.43419	-3.96835
C	-2.23795	0.361269	-4.59092
O	-1.48261	0.102954	-5.57971
O	-2.00113	1.2245	-3.70021
C	-5.26853	-1.88385	-5.66809
O	-6.48265	-1.69388	-5.79506
N	-4.75981	-3.13542	-5.44534
H	-3.7673	-3.26109	-5.24599
C	-5.56672	-4.32901	-5.60844
H	-6.23634	-4.20578	-6.4677
C	-6.46457	-4.72325	-4.43495
O	-7.05879	-5.80432	-4.43724
N	-6.58237	-3.80964	-3.44037
H	-6.00943	-2.97808	-3.49652
C	-7.57726	-3.91554	-2.38588
H	-8.22293	-4.75341	-2.67123
C	-6.94188	-4.18683	-1.01701
H	-7.74394	-4.24998	-0.27312
H	-6.31772	-3.33645	-0.728
C	-6.07017	-5.43122	-0.90082
O	-5.14501	-5.47154	-0.0694
N	-6.36718	-6.49842	-1.67237
H	-6.89324	-6.41688	-2.54318
H	-5.75218	-7.29764	-1.583
C	-8.5181	-2.68519	-2.30778
O	-9.19595	-2.51595	-1.29884
N	-8.55944	-1.89881	-3.41253
H	-7.97147	-2.11449	-4.21314
C	-9.50601	-0.79865	-3.56324
H	-10.1307	-0.7928	-2.66741
C	-8.82673	0.572186	-3.77494
H	-8.07704	0.46757	-4.56919
H	-9.59458	1.257387	-4.15706
C	-8.19884	1.161699	-2.53064
C	-6.83951	0.984399	-2.24147
H	-6.21916	0.431357	-2.94184
C	-6.25259	1.515473	-1.0928
H	-5.19139	1.382937	-0.90213
C	-7.04668	2.235089	-0.19754

O	-6.44835	2.715256	0.955539
H	-7.00443	3.418014	1.32888
C	-8.40429	2.433548	-0.46122
H	-9.02067	2.999112	0.235327
C	-8.96629	1.902313	-1.62217
H	-10.0226	2.06983	-1.82075
C	3.767665	6.151082	-4.25039
H	3.943631	6.513757	-3.22997
H	2.93515	6.71272	-4.68072
C	3.438019	4.664145	-4.16114
O	4.438603	3.865426	-4.12723
O	2.23079	4.329939	-4.10109
C	9.44454	-4.89071	-1.32633
C	8.180408	-4.12951	-1.09269
C	7.229799	-3.63788	-1.95332
N	7.751342	-3.73701	0.165435
C	6.591043	-3.05433	0.027062
N	6.244029	-2.9722	-1.24751
H	9.469703	-5.83091	-0.76079
H	9.540544	-5.13781	-2.38732
H	7.183732	-3.73195	-3.02809
H	6.03484	-2.65452	0.86129
H	8.068261	-4.08499	1.067086
C	2.009157	-7.05568	0.871632
H	2.105721	-7.51947	-0.12001
C	1.36612	-5.66576	0.705403
H	1.998531	-5.02338	0.083184
H	0.432721	-5.79728	0.142123
C	1.047188	-4.97986	2.038275
H	0.403669	-5.63901	2.639742
H	1.963462	-4.82664	2.62411
C	0.344501	-3.63148	1.840662
H	1.025018	-2.91215	1.378912
H	-0.50175	-3.74963	1.151461
N	-0.1263	-3.11076	3.124317
H	-0.69965	-3.73848	3.680398
C	-0.21106	-1.81684	3.504351
N	0.24916	-0.80325	2.756403
H	0.916978	-0.96118	1.997087
H	0.346224	0.119135	3.249058
N	-0.77148	-1.56192	4.692605
H	-1.32375	-2.31923	5.108262
H	-1.11288	-0.61755	4.900581
C	3.386413	-7.11483	1.529124
O	3.623126	-7.90668	2.451075
N	4.350274	-6.30082	1.01633
H	4.142065	-5.71586	0.201184

C	5.762336	-6.54092	1.308416
H	5.948778	-7.62235	1.308407
C	6.260058	-5.96394	2.648313
O	7.06949	-5.01567	2.69246
N	5.781954	-6.58345	3.738485
H	5.059785	-7.28748	3.567686
C	6.186704	-6.24387	5.102378
H	7.209533	-5.86143	5.049019
C	5.286054	-5.20703	5.79423
H	5.725579	-4.97315	6.771511
H	5.301708	-4.28344	5.206468
C	3.845446	-5.68749	5.993167
H	3.366854	-5.91279	5.031967
H	3.828686	-6.61287	6.582089
S	2.781016	-4.52852	6.938411
C	2.650071	-3.13085	5.766346
H	2.017607	-2.37864	6.244791
H	3.621818	-2.68487	5.540591
H	2.163712	-3.44669	4.838004
O	2.410302	-2.36269	-2.27675
O	5.382538	-2.75097	3.205118
Fe	4.106247	-2.47749	-1.76923
C	1.637478	-2.53268	-4.6691
H	2.482327	-3.18403	-4.90235
H	1.818016	-2.61027	-3.33446
C	0.328584	-3.12109	-5.18959
H	0.574769	-4.04077	-5.73484
C	-0.83721	-3.57517	-4.22017
O	-0.53269	-3.89443	-3.05585
O	-1.95938	-3.6239	-4.79636
N	-0.38386	-2.25514	-6.21167
H	-0.72871	-1.29414	-5.86642
H	-1.26449	-2.77851	-6.36334
H	0.137589	-2.13215	-7.08169
C	1.930677	-1.06422	-4.92394
H	1.700528	-0.80122	-5.97005
H	3.005707	-0.90584	-4.80454
C	1.164696	-0.12729	-3.97957
H	1.540053	-0.28609	-2.96671
H	0.105426	-0.38409	-3.9602
C	1.299955	1.32531	-4.4018
H	2.339244	1.650157	-4.47325
H	0.817404	1.489738	-5.36807
N	0.628442	2.237462	-3.42785
H	6.348714	-6.07838	0.514341
H	6.204013	-7.17781	5.676313
H	-3.63848	-6.79719	5.80959

H	1.365511	-7.70843	1.469037
H	-8.01777	-1.16746	2.984535
H	-6.01572	2.506142	4.685893
H	10.3289	-4.30924	-1.03697
H	8.738801	1.263038	-3.70791
H	4.678149	6.321155	-4.83272
H	6.453239	5.751341	-1.91475
H	-1.12134	8.091465	4.681663
H	-4.77022	0.130984	-6.08653
H	-4.90925	-5.17656	-5.81754
H	-10.1488	-1.01326	-4.4269
H	6.11397	-3.40023	3.179737
H	4.639903	-3.23634	2.814367
C	4.205055	-4.92841	-2.5368
O	3.940839	-4.6373	-1.31957
O	4.477871	-4.00831	-3.35796
C	4.155571	-6.37283	-2.98386
H	4.596876	-7.02552	-2.22477
H	4.67418	-6.50172	-3.93589
H	3.107548	-6.67086	-3.10889
H	1.02683	3.190296	-3.59835
H	-0.40226	2.149606	-3.56121
H	0.835095	1.971869	-2.4385
O	1.04471	1.56049	-0.67212
H	1.474373	0.729358	-0.37562
H	0.165259	1.498446	-0.21206
O	-2.94904	0.336777	-1.20708
H	-2.64735	0.755914	-2.04855
H	-2.44358	0.775967	-0.48342
O	-1.21361	1.00907	0.789576
H	-0.91977	0.181166	1.212696
H	-1.09958	1.696394	1.527953

^{5}**IM1**$_{B,C3}$

C	-4.04663	-1.656	5.659592
H	-3.30301	-1.40131	4.892529
C	-3.32634	-1.96876	6.975387
H	-4.0546	-2.02579	7.793086
H	-2.86191	-2.95462	6.900325
C	-2.25428	-0.93067	7.332055
H	-2.68346	0.078948	7.387053
H	-1.85582	-1.14752	8.332902
C	-1.06775	-0.92795	6.363304
O	-0.84478	-1.89734	5.622288
N	-0.2644	0.152085	6.390323
H	-0.51827	0.966856	6.930119
H	0.4574	0.253585	5.664117
C	-4.85442	-2.82526	5.096988
O	-4.65263	-3.99994	5.430008
N	-5.77945	-2.48681	4.164686
H	-5.84668	-1.51507	3.871477
C	-6.43813	-3.48545	3.32466
H	-7.31251	-3.00211	2.876271
C	-5.51609	-4.0582	2.229716
H	-4.72832	-4.64932	2.713072
H	-6.10576	-4.75271	1.615355
C	-4.87198	-2.96487	1.36728
H	-4.37267	-2.24582	2.027115
H	-5.6424	-2.39811	0.826483
C	-3.81333	-3.44863	0.372651
H	-3.2753	-2.5791	-0.01107
H	-3.08545	-4.09385	0.886565
N	-4.41027	-4.1716	-0.75312
H	-5.23429	-4.75135	-0.58874
C	-3.92555	-4.20256	-2.00274
N	-2.86767	-3.48183	-2.38915
H	-2.49941	-2.67836	-1.85402
H	-2.66703	-3.51469	-3.38751
N	-4.50387	-5.02253	-2.90444
H	-5.31488	-5.55624	-2.61291
H	-4.32007	-4.83851	-3.89095
C	0.252255	-8.06625	4.106209
H	1.095426	-8.06094	4.807904
H	0.667822	-7.95124	3.096208
C	-0.72709	-6.97984	4.425804
N	-0.3548	-5.64707	4.469004
C	-1.45404	-4.9751	4.781176
H	-1.51877	-3.90586	4.932457
N	-2.51368	-5.80055	4.938723

H	-3.44523	-5.46514	5.181315
C	-2.06797	-7.08629	4.71543
H	-2.72211	-7.94286	4.779106
C	6.703623	5.21291	-4.33953
C	6.264525	5.526156	-2.90725
C	5.699018	4.412953	-5.20532
O	6.997151	5.253652	-1.94972
C	5.206523	3.170746	-4.5236
C	5.79895	1.968321	-4.22092
N	3.962298	3.139418	-3.92457
C	3.836678	1.972787	-3.28525
N	4.932714	1.224234	-3.44353
H	6.934361	6.158403	-4.84815
H	4.834912	5.03431	-5.46503
H	6.194175	4.159149	-6.14989
H	6.780426	1.601798	-4.4788
H	2.984255	1.704561	-2.68531
H	3.265622	3.96347	-3.9417
N	5.079644	6.175342	-2.74841
H	4.333513	6.114964	-3.44516
C	4.650495	6.601385	-1.42326
H	5.536988	6.923408	-0.8694
C	3.900208	5.524703	-0.62458
O	2.692267	5.642994	-0.38889
N	4.652452	4.480788	-0.20787
C	4.113036	3.314188	0.499722
C	3.218495	3.775331	1.678767
C	5.288184	2.485557	1.053892
O	3.689383	4.363587	2.64653
C	4.903638	1.085646	1.562389
C	4.759703	0.008725	0.490108
O	4.042456	-1.00044	0.690391
O	5.463555	0.168251	-0.57781
H	5.631546	4.480144	-0.48877
H	3.519004	2.710478	-0.19914
H	6.048322	2.372525	0.274105
H	5.72614	3.061866	1.875107
H	3.99225	1.098054	2.169684
H	5.706425	0.719668	2.216857
N	1.900805	3.461969	1.562031
H	1.591794	2.968072	0.735053
C	0.908072	3.749658	2.59473
H	-0.05852	3.515798	2.135824
C	1.056732	2.808244	3.79492
H	2.062474	2.872615	4.216534
H	0.355486	3.117367	4.578014
C	0.754112	1.364857	3.376561

O	-0.06593	1.207662	2.432004
O	1.34807	0.439053	4.018537
C	0.820542	5.226416	3.014709
O	0.325874	5.530274	4.106654
N	1.169916	6.160971	2.094211
H	1.665777	5.871057	1.250836
C	1.226053	7.572156	2.476042
H	1.626286	7.632115	3.494266
C	2.136775	8.361095	1.530755
H	3.163413	7.987422	1.591737
H	2.120048	9.414227	1.821465
H	1.799726	8.274173	0.492266
C	-0.15417	8.254214	2.581877
O	-0.25129	9.368002	3.085822
N	-1.21218	7.541256	2.103214
H	-1.02083	6.647366	1.669176
C	-2.59173	7.958988	2.265073
H	-2.54715	9.004858	2.589193
C	-3.38072	7.890694	0.935718
H	-2.96598	8.652242	0.265648
H	-4.41258	8.168657	1.166639
C	-3.33686	6.53591	0.257323
C	-2.34956	6.244457	-0.69635
H	-1.63053	7.01426	-0.96955
C	-2.27991	4.99016	-1.30966
H	-1.5021	4.797243	-2.0458
C	-3.21042	4.002465	-0.97658
H	-3.16624	3.02643	-1.45293
C	-4.2026	4.281012	-0.03174
H	-4.93529	3.519244	0.225774
C	-4.26582	5.533917	0.581325
H	-5.03479	5.747494	1.318446
C	-3.33369	7.2031	3.397242
O	-4.56275	7.269195	3.481179
N	-2.54885	6.541289	4.280809
H	-1.54456	6.490957	4.129955
C	-3.07824	5.868219	5.457226
H	-4.12266	6.171789	5.553508
C	-2.93083	4.339523	5.393827
H	-1.8622	4.112089	5.282222
H	-3.24098	3.92808	6.367734
C	-3.71513	3.631387	4.273135
H	-3.4302	4.092521	3.317765
C	-3.32374	2.14722	4.209024
H	-3.90041	1.615738	3.443568
H	-2.26442	2.011954	3.964502
H	-3.51406	1.652957	5.174307

C	-5.23477	3.799311	4.431961
H	-5.76738	3.274433	3.627058
H	-5.57986	3.393139	5.395016
H	-5.53277	4.850972	4.377689
C	-5.54288	0.279676	-4.78245
H	-5.06657	0.08291	-5.7443
C	-4.54898	-0.03776	-3.65055
H	-4.96226	0.242129	-2.67769
H	-4.35313	-1.11919	-3.6185
C	-3.18841	0.662851	-3.82185
O	-2.72696	0.82446	-4.99261
O	-2.59138	1.016919	-2.76632
C	-6.84092	-0.5042	-4.6791
O	-7.79347	-0.10569	-3.98154
N	-6.89746	-1.66547	-5.37606
H	-6.04867	-2.00662	-5.84305
C	-8.13776	-2.38595	-5.62274
H	-8.91813	-1.66986	-5.91545
C	-8.7376	-3.22427	-4.48691
O	-9.40653	-4.22892	-4.74557
N	-8.54626	-2.75576	-3.2271
H	-8.05832	-1.86774	-3.14225
C	-9.31545	-3.24047	-2.0883
H	-10.112	-3.87015	-2.50002
C	-8.46394	-4.06428	-1.11131
H	-9.09701	-4.34086	-0.26033
H	-7.65209	-3.44887	-0.71107
C	-7.82721	-5.33148	-1.66442
O	-6.78267	-5.7831	-1.16028
N	-8.45827	-5.9843	-2.66416
H	-9.08426	-5.50638	-3.31511
H	-7.99545	-6.81616	-3.00979
C	-10.0533	-2.11312	-1.3214
O	-10.5449	-2.36172	-0.22564
N	-10.1529	-0.91632	-1.95622
H	-9.67649	-0.77068	-2.83873
C	-10.8376	0.23145	-1.37566
H	-11.2952	-0.11282	-0.44578
C	-9.89394	1.426748	-1.12002
H	-9.3765	1.67504	-2.05503
H	-10.5216	2.290836	-0.86732
C	-8.88632	1.183815	-0.01845
C	-7.60295	0.694985	-0.29986
H	-7.31531	0.530096	-1.3356
C	-6.68338	0.444013	0.720195
H	-5.67865	0.09654	0.500244
C	-7.04892	0.676228	2.047099

O	-6.12429	0.383444	3.039245
H	-6.25416	0.995773	3.782066
C	-8.31804	1.171239	2.351779
H	-8.59936	1.354879	3.386248
C	-9.22112	1.423825	1.320491
H	-10.2062	1.813975	1.566281
C	0.905739	6.893404	-3.32956
H	1.122766	7.085437	-2.27139
H	-0.17132	6.98949	-3.48664
C	1.379362	5.479291	-3.6432
O	2.606671	5.355176	-3.9875
O	0.561033	4.537733	-3.5163
C	10.87671	-1.35001	-3.55858
C	9.564101	-1.02394	-2.92427
C	8.340196	-0.70235	-3.45595
N	9.377524	-0.96385	-1.55074
C	8.091481	-0.62922	-1.3069
N	7.430692	-0.45738	-2.44178
H	11.26197	-2.3237	-3.23008
H	10.76671	-1.38557	-4.64597
H	8.052303	-0.64368	-4.49535
H	7.673697	-0.54454	-0.31488
H	9.982824	-1.33448	-0.82275
C	5.46286	-6.52621	-0.73366
H	5.495274	-6.79811	-1.79823
C	4.314255	-5.52801	-0.50724
H	4.516648	-4.58543	-1.02958
H	3.41174	-5.94298	-0.97551
C	4.022143	-5.2577	0.974287
H	3.697243	-6.19211	1.455028
H	4.933042	-4.93948	1.497831
C	2.947912	-4.18041	1.148295
H	3.35348	-3.21553	0.840364
H	2.085056	-4.39994	0.499833
N	2.517633	-4.09776	2.545499
H	2.247732	-4.98009	2.966829
C	1.918437	-3.01738	3.114828
N	2.033155	-1.79221	2.582397
H	2.828774	-1.5614	1.974974
H	1.688592	-0.98421	3.156744
N	1.218504	-3.21784	4.228575
H	0.869714	-4.16835	4.422342
H	0.678977	-2.45817	4.65642
C	6.869882	-6.06955	-0.35395
O	7.641742	-6.82273	0.254452
N	7.256697	-4.83475	-0.77192
H	6.618996	-4.22079	-1.2874

C	8.664685	-4.44988	-0.76616
H	9.268821	-5.31137	-1.07676
C	9.201979	-3.94357	0.585579
O	9.596175	-2.76897	0.731267
N	9.244839	-4.86348	1.561001
H	8.848056	-5.7778	1.332054
C	9.807506	-4.59577	2.884919
H	10.62411	-3.88072	2.750762
C	8.80535	-4.03486	3.907208
H	9.361599	-3.77181	4.815134
H	8.382698	-3.10511	3.513329
C	7.687388	-5.01501	4.274937
H	7.091945	-5.28965	3.395368
H	8.111017	-5.94412	4.67573
S	6.564284	-4.41437	5.59694
C	5.614705	-3.1304	4.704189
H	4.968496	-2.65266	5.445576
H	6.262113	-2.36934	4.260623
H	4.981518	-3.57894	3.931995
O	3.443021	-1.00435	-2.04952
O	7.381718	-1.33543	1.931597
Fe	5.214868	-0.55801	-2.42904
C	-0.93162	-2.16663	-5.5273
H	-0.90693	-3.00716	-4.84181
H	3.470574	-1.35263	-1.13023
C	-2.02833	-2.20832	-6.53214
H	-1.7281	-2.75457	-7.44482
C	-3.3607	-2.89801	-6.0401
O	-3.24072	-3.83487	-5.22472
O	-4.40066	-2.41776	-6.56967
N	-2.45982	-0.83969	-7.0062
H	-2.60883	-0.16482	-6.1681
H	-3.39569	-1.01288	-7.41369
H	-1.82362	-0.41589	-7.68288
C	0.222381	-1.21091	-5.52947
H	0.13996	-0.5048	-6.36749
H	1.156899	-1.76505	-5.70685
C	0.357947	-0.41067	-4.19226
H	1.258696	-0.72483	-3.65412
H	-0.49066	-0.63961	-3.53938
C	0.393488	1.102588	-4.43696
H	1.373553	1.434294	-4.79218
H	-0.36764	1.389494	-5.16446
N	0.06941	1.860026	-3.19189
H	8.790555	-3.64481	-1.49013
H	10.23752	-5.53642	3.247587
H	-0.22315	-9.05075	4.159098

H	5.286201	-7.45204	-0.17775
H	-6.79398	-4.29404	3.96901
H	-4.69203	-0.77423	5.764228
H	11.64004	-0.5977	-3.32367
H	7.637497	4.653617	-4.24209
H	1.444269	7.633015	-3.929
H	3.966086	7.443706	-1.53414
H	-2.54129	6.239594	6.339792
H	-5.81101	1.340024	-4.74611
H	-7.97892	-3.06407	-6.46255
H	-11.6405	0.545417	-2.05548
H	8.216914	-1.74821	1.633437
H	6.684536	-1.88951	1.547177
C	5.664108	-2.8391	-3.54788
O	5.755111	-2.85425	-2.2805
O	5.432411	-1.74604	-4.1538
C	5.79152	-4.11606	-4.34882
H	6.426936	-4.84051	-3.83262
H	6.18939	-3.90592	-5.34422
H	4.794834	-4.55764	-4.46948
H	0.280961	2.8842	-3.31701
H	-0.94864	1.71323	-2.99017
H	0.621059	1.502794	-2.37333
O	1.632686	0.892286	-1.07355
H	2.260421	0.222543	-1.43796
H	1.076145	0.382091	-0.42653
O	-1.98177	-1.17348	-1.1429
H	-2.25753	-0.32588	-1.55461
H	-1.25504	-0.9789	-0.50357
O	0.075414	-0.6033	0.601262
H	0.616466	-1.30022	1.016989
H	-0.08725	0.043039	1.367617

^{5}**TS2**$_{B,C3}$

C	-5.41712	0.55378	5.302027
H	-4.63827	0.90597	4.616709
C	-4.78236	0.03613	6.601861
H	-5.499	0.121514	7.425811
H	-4.55999	-1.02829	6.500211
C	-3.49747	0.786796	6.972614
H	-3.67049	1.871392	7.01047
H	-3.17059	0.496376	7.980436
C	-2.34225	0.504486	6.008023
O	-2.44601	-0.34104	5.107532
N	-1.1985	1.191553	6.210736
H	-1.17688	1.961684	6.864403
H	-0.49108	1.174376	5.467961
C	-6.2469	-0.4934	4.556913
O	-6.54861	-1.58886	5.043471
N	-6.62416	-0.12128	3.305682
H	-6.32986	0.788267	2.973082
C	-7.43954	-0.94848	2.418402
H	-8.07755	-0.27206	1.838614
C	-6.63632	-1.84323	1.456055
H	-6.15303	-2.63943	2.038144
H	-7.3588	-2.33733	0.795324
C	-5.5843	-1.07274	0.621407
H	-5.81387	-0.00104	0.615988
H	-5.62845	-1.37075	-0.43197
C	-4.13682	-1.24422	1.11369
H	-3.5173	-0.4113	0.781615
H	-4.09866	-1.24663	2.208289
N	-3.51016	-2.4919	0.651451
H	-3.8369	-3.38267	1.030673
C	-2.46031	-2.5812	-0.17172
N	-1.95454	-1.50821	-0.81134
H	-2.55593	-0.7155	-1.07469
H	-1.1563	-1.706	-1.41539
N	-1.8499	-3.76404	-0.34489
H	-2.23643	-4.58988	0.101206
H	-1.13531	-3.8262	-1.06774
C	-2.61571	-6.61814	3.777354
H	-1.65167	-6.68011	4.294449
H	-2.42402	-6.6481	2.697056
C	-3.34551	-5.36681	4.154445
N	-2.70635	-4.14204	4.230635
C	-3.64644	-3.26214	4.542877
H	-3.48704	-2.20557	4.711209
N	-4.85878	-3.85303	4.670429

H	-5.7104	-3.33615	4.884865
C	-4.68414	-5.19892	4.427346
H	-5.50297	-5.90166	4.462354
C	7.798006	4.200836	-3.26345
C	7.179348	4.744327	-1.97226
C	6.843	3.43298	-4.2135
O	7.639449	4.431075	-0.86943
C	6.094471	2.335491	-3.52098
C	6.403927	1.036484	-3.18897
N	4.864726	2.583152	-2.94163
C	4.483512	1.472397	-2.29105
N	5.387349	0.504535	-2.41972
H	8.243685	5.038121	-3.81617
H	6.119975	4.120524	-4.66702
H	7.44163	3.018754	-5.03236
H	7.278332	0.454857	-3.4404
H	3.561864	1.388109	-1.7408
H	4.304007	3.463018	-3.10792
N	6.160188	5.639485	-2.10645
H	5.53484	5.6002	-2.91621
C	5.594548	6.284563	-0.92778
H	6.405169	6.439566	-0.21044
C	4.45666	5.491526	-0.2714
O	3.293193	5.904188	-0.30037
N	4.82453	4.327968	0.315673
C	3.850529	3.374314	0.845289
C	2.89601	4.082605	1.842104
C	4.593102	2.229112	1.561539
O	3.322147	4.622192	2.857127
C	3.718542	0.987569	1.863376
C	3.563924	0.052991	0.659353
O	2.431291	-0.11177	0.109817
O	4.627687	-0.52533	0.269259
H	5.808618	4.073007	0.268188
H	3.259976	2.98272	0.008202
H	5.451119	1.910214	0.957757
H	4.981091	2.637014	2.500826
H	2.737173	1.276185	2.252508
H	4.222707	0.401523	2.640532
N	1.582234	4.024815	1.499362
H	1.323729	3.525396	0.64867
C	0.5113	4.563056	2.329686
H	-0.41197	4.403384	1.760114
C	0.325262	3.78066	3.640999
H	1.275922	3.675092	4.166029
H	-0.36905	4.347941	4.269055
C	-0.28151	2.417345	3.30507

O	-1.33813	2.437314	2.609926
O	0.312876	1.37087	3.716066
C	0.553599	6.083871	2.548842
O	-0.11417	6.5935	3.455543
N	1.198904	6.830956	1.618582
H	1.843829	6.383623	0.966968
C	1.259375	8.282897	1.780501
H	1.539988	8.510757	2.814421
C	2.290089	8.896406	0.827201
H	3.288067	8.498321	1.031856
H	2.299949	9.980415	0.966551
H	2.043612	8.676634	-0.21727
C	-0.10305	8.984212	1.600176
O	-0.26644	10.12053	2.02894
N	-1.06252	8.281991	0.929132
H	-0.82218	7.367453	0.569209
C	-2.41532	8.774669	0.748504
H	-2.38734	9.823379	1.064163
C	-2.86612	8.730826	-0.73275
H	-2.23571	9.432852	-1.28984
H	-3.89269	9.106032	-0.75713
C	-2.79471	7.360764	-1.37822
C	-1.66265	6.973055	-2.10959
H	-0.83716	7.672588	-2.22525
C	-1.58172	5.714165	-2.71168
H	-0.68919	5.457693	-3.27709
C	-2.64479	4.817134	-2.58362
H	-2.6003	3.837416	-3.05535
C	-3.7807	5.185081	-1.85544
H	-4.61165	4.490135	-1.75619
C	-3.85502	6.446317	-1.26014
H	-4.73925	6.735027	-0.6991
C	-3.46441	8.095127	1.662338
O	-4.66411	8.301328	1.461843
N	-2.98697	7.327957	2.671037
H	-1.9832	7.250772	2.814546
C	-3.85512	6.740016	3.685325
H	-4.80352	7.281141	3.650593
C	-4.07896	5.225403	3.526333
H	-3.10388	4.719726	3.539897
H	-4.62236	4.878879	4.420633
C	-4.84984	4.776293	2.269297
H	-4.29066	5.128664	1.392087
C	-4.89021	3.240027	2.20271
H	-5.36462	2.898796	1.273643
H	-3.88256	2.813016	2.260746
H	-5.47359	2.846619	3.051094

C	-6.26688	5.36413	2.196929
H	-6.80619	4.963965	1.329218
H	-6.84637	5.104127	3.094845
H	-6.24241	6.453119	2.098756
C	-3.28016	-1.86078	-5.73499
H	-2.41021	-2.09588	-6.35959
C	-2.78874	-1.22468	-4.42219
H	-3.62855	-0.81205	-3.86063
H	-2.327	-1.99442	-3.79228
C	-1.71294	-0.14353	-4.63196
O	-0.77219	-0.3968	-5.4485
O	-1.79498	0.912564	-3.94147
C	-4.10532	-3.12805	-5.54239
O	-5.28023	-3.22662	-5.90207
N	-3.44961	-4.1768	-4.94911
H	-2.50529	-4.0537	-4.5848
C	-4.00034	-5.5177	-4.96969
H	-4.5858	-5.6471	-5.88745
C	-4.92327	-5.91037	-3.81425
O	-5.16268	-7.09918	-3.58627
N	-5.47389	-4.89112	-3.1076
H	-5.18407	-3.94772	-3.332
C	-6.47768	-5.10328	-2.07416
H	-6.81233	-6.13952	-2.19706
C	-5.92686	-4.89416	-0.65714
H	-6.75471	-5.02675	0.047962
H	-5.59729	-3.85817	-0.54301
C	-4.77274	-5.7753	-0.19894
O	-4.00627	-5.37184	0.69486
N	-4.65141	-7.01001	-0.72876
H	-5.05904	-7.26152	-1.63
H	-3.86471	-7.56054	-0.40796
C	-7.74647	-4.23313	-2.26733
O	-8.49329	-4.04292	-1.31044
N	-7.97811	-3.77948	-3.52336
H	-7.2979	-3.96288	-4.25211
C	-9.18734	-3.03787	-3.87203
H	-9.72798	-2.88362	-2.93605
C	-8.90305	-1.69747	-4.57541
H	-8.37791	-1.8923	-5.51927
H	-9.87831	-1.27088	-4.84867
C	-8.10387	-0.71412	-3.74756
C	-6.79111	-0.3837	-4.1017
H	-6.35849	-0.82251	-4.99818
C	-6.02854	0.493432	-3.32966
H	-5.02585	0.770401	-3.63887
C	-6.55898	1.041389	-2.15369

O	-5.82629	1.84187	-1.33154
H	-4.86577	1.662623	-1.48997
C	-7.88693	0.751422	-1.80859
H	-8.30646	1.203642	-0.91458
C	-8.64181	-0.11116	-2.60009
H	-9.6677	-0.32876	-2.3092
C	2.688098	7.030858	-3.79583
H	2.721191	7.367106	-2.752
H	1.784885	7.426604	-4.26534
C	2.68164	5.505428	-3.79029
O	3.812213	4.935957	-3.61585
O	1.574541	4.926908	-3.92954
C	10.49329	-3.09682	0.017739
C	9.097914	-2.56294	0.005176
C	8.363848	-1.93072	-0.96733
N	8.260162	-2.60149	1.110489
C	7.086401	-2.01767	0.775139
N	7.113223	-1.59712	-0.48075
H	10.52603	-4.15974	0.289689
H	10.94119	-2.99371	-0.97464
H	8.653694	-1.69658	-1.98143
H	6.256793	-1.92513	1.461531
H	8.371796	-3.1551	1.960608
C	3.475316	-6.46408	-0.30817
H	3.88116	-6.617	-1.31689
C	2.758914	-5.09916	-0.26791
H	3.471804	-4.29564	-0.4726
H	2.039713	-5.06	-1.09506
C	2.015102	-4.83953	1.047665
H	1.157053	-5.52201	1.128604
H	2.667307	-5.05713	1.903226
C	1.52756	-3.39231	1.158067
H	2.394421	-2.72455	1.242192
H	0.980464	-3.10406	0.254505
N	0.657498	-3.25433	2.327315
H	0.603297	-4.04213	2.964874
C	0.15638	-2.09703	2.800153
N	0.385494	-0.92614	2.185339
H	1.130799	-0.84858	1.492965
H	0.213024	-0.05694	2.740957
N	-0.59183	-2.14487	3.911516
H	-1.11158	-3.01614	4.104041
H	-1.07319	-1.2964	4.226179
C	4.618212	-6.63116	0.691038
O	4.609755	-7.53067	1.54548
N	5.647953	-5.75456	0.574191
H	5.640168	-5.05435	-0.17548

C	6.895351	-5.94071	1.304615
H	7.179602	-6.99992	1.270443
C	6.852162	-5.49787	2.781045
O	7.476378	-4.4944	3.187289
N	6.134475	-6.30421	3.576742
H	5.588945	-7.02105	3.088886
C	6.002624	-6.14397	5.023864
H	6.938288	-5.71587	5.394935
C	4.820212	-5.26366	5.470097
H	4.777717	-5.28473	6.566622
H	5.017987	-4.23075	5.168021
C	3.483495	-5.72205	4.884588
H	3.524196	-5.68807	3.791549
H	3.268842	-6.76011	5.166054
S	2.006249	-4.78574	5.462524
C	2.581944	-3.04766	5.38973
H	1.682776	-2.43085	5.458479
H	3.242709	-2.8145	6.228378
H	3.100633	-2.82921	4.4527
O	3.380625	-1.97812	-1.95074
O	5.700914	-2.34494	3.713825
Fe	5.177602	-1.43057	-1.52388
C	2.619504	-2.08062	-4.12741
H	3.585032	-2.56468	-4.19752
H	2.698348	-1.50544	-1.42919
C	1.44562	-2.98871	-4.35625
H	1.827728	-3.99976	-4.53255
C	0.242395	-3.21216	-3.33741
O	0.331615	-2.8072	-2.16218
O	-0.70317	-3.84352	-3.88547
N	0.686945	-2.62463	-5.63589
H	0.125486	-1.69837	-5.55829
H	-0.03336	-3.36046	-5.69432
H	1.278539	-2.61261	-6.46966
C	2.590255	-0.6257	-4.45944
H	2.312689	-0.5029	-5.52367
H	3.606807	-0.23428	-4.36595
C	1.624559	0.221935	-3.62155
H	1.979225	0.248526	-2.58984
H	0.63406	-0.23008	-3.60404
C	1.517164	1.624416	-4.20274
H	2.481651	2.136838	-4.22513
H	1.122835	1.581566	-5.22086
N	0.586644	2.480522	-3.41285
H	7.664951	-5.35175	0.805403
H	5.904035	-7.15126	5.44421
H	-3.20019	-7.50757	4.035028

H	2.774057	-7.27952	-0.10622
H	-8.08684	-1.56369	3.048792
H	-6.05984	1.421255	5.506377
H	11.13056	-2.55632	0.728948
H	8.607737	3.539903	-2.94454
H	3.577257	7.418287	-4.30295
H	5.179604	7.250445	-1.22103
H	-3.39621	6.929425	4.663258
H	-3.90142	-1.1573	-6.29498
H	-3.18563	-6.24573	-4.98457
H	-9.81656	-3.66213	-4.52124
H	6.357138	-3.07148	3.672779
H	6.085497	-1.69676	4.323251
C	6.044349	-3.6229	-2.6827
O	5.617545	-3.80973	-1.50677
O	6.009615	-2.46321	-3.20915
C	6.648317	-4.76892	-3.47059
H	6.227898	-5.7266	-3.15278
H	7.729063	-4.79265	-3.2845
H	6.497253	-4.62395	-4.54317
H	0.836808	3.489044	-3.62156
H	-0.38394	2.207539	-3.6556
H	0.716348	2.333573	-2.38353
O	0.881253	2.062476	-0.63818
H	1.448297	1.311904	-0.33959
H	-0.01762	1.817028	-0.28737
O	-3.23921	0.947526	-1.60892
H	-2.76102	1.023759	-2.47637
H	-2.63611	1.303288	-0.91128
O	-1.43931	1.169666	0.412121
H	-1.22491	0.23341	0.57533
H	-1.47207	1.613297	1.334048

[5]**Pr**$_{B,C3}$

C	-5.33027	1.024229	5.407975
H	-4.55063	1.293079	4.686792
C	-4.69151	0.476422	6.693456
H	-5.36673	0.635304	7.541215
H	-4.5634	-0.60464	6.604478
C	-3.33539	1.122432	7.003088
H	-3.41548	2.218457	7.020999
H	-2.99878	0.826937	8.006132
C	-2.24236	0.726661	6.00622
O	-2.45729	-0.09607	5.104663
N	-1.02855	1.291308	6.180003
H	-0.90786	2.048321	6.838122
H	-0.34617	1.202579	5.419546
C	-6.27482	0.039789	4.715733
O	-6.63667	-1.02534	5.227595
N	-6.68262	0.431454	3.479864
H	-6.32783	1.308578	3.120683
C	-7.58419	-0.34577	2.632613
H	-8.20242	0.365706	2.073781
C	-6.87126	-1.29336	1.649408
H	-6.40309	-2.10311	2.224837
H	-7.64503	-1.76035	1.027564
C	-5.81939	-0.58699	0.757946
H	-5.93663	0.500982	0.822488
H	-5.98458	-0.82469	-0.29826
C	-4.36029	-0.92365	1.110198
H	-3.68759	-0.1456	0.748071
H	-4.22703	-0.97554	2.19701
N	-3.9184	-2.20674	0.544739
H	-4.33959	-3.07197	0.892088
C	-2.89319	-2.37382	-0.29908
N	-2.28837	-1.35114	-0.91537
H	-2.75694	-0.46293	-1.12517
H	-1.38648	-1.54947	-1.36512
N	-2.41341	-3.61545	-0.52337
H	-2.8719	-4.39383	-0.06069
H	-1.89751	-3.76665	-1.39119
C	-3.15921	-6.32157	3.755032
H	-2.18235	-6.4622	4.230971
H	-3.01379	-6.35124	2.66737
C	-3.77711	-5.02505	4.177054
N	-3.04278	-3.85503	4.253347
C	-3.90091	-2.91195	4.612573
H	-3.65516	-1.87318	4.787975
N	-5.15056	-3.41052	4.771361

H	-5.95213	-2.83356	5.021944
C	-5.08899	-4.76065	4.498716
H	-5.95802	-5.39915	4.550166
C	8.200662	3.728746	-3.2036
C	7.585203	4.273847	-1.91228
C	7.220674	3.038154	-4.18672
O	8.000857	3.908941	-0.80785
C	6.395279	1.975901	-3.52768
C	6.627488	0.659181	-3.20118
N	5.165816	2.280231	-2.97752
C	4.710655	1.180071	-2.35309
N	5.564372	0.167253	-2.46867
H	8.703221	4.552553	-3.72706
H	6.549719	3.776592	-4.64042
H	7.809082	2.603794	-5.0026
H	7.482721	0.040968	-3.43267
H	3.772725	1.139852	-1.82631
H	4.65963	3.189188	-3.13255
N	6.619396	5.226855	-2.04591
H	6.010215	5.241454	-2.86879
C	6.063326	5.871103	-0.86291
H	6.86319	5.956191	-0.12213
C	4.863003	5.133422	-0.25529
O	3.731041	5.626126	-0.28915
N	5.137745	3.92825	0.297211
C	4.090345	3.038244	0.799613
C	3.200413	3.794365	1.823231
C	4.742375	1.813249	1.4713
O	3.673883	4.262603	2.852897
C	3.774853	0.637829	1.760475
C	3.518894	-0.25895	0.544055
O	2.39296	-0.26025	-0.0319
O	4.497156	-0.99084	0.176021
H	6.104372	3.611495	0.270491
H	3.468571	2.724972	-0.04756
H	5.563666	1.444	0.84508
H	5.176893	2.160784	2.414644
H	2.826124	1.000755	2.168436
H	4.240539	-0.0004	2.519789
N	1.888131	3.870479	1.479547
H	1.58296	3.413803	0.619581
C	0.86849	4.488113	2.318579
H	-0.06136	4.429189	1.740301
C	0.598081	3.695792	3.60843
H	1.52981	3.491429	4.138365
H	-0.04978	4.307507	4.244285
C	-0.12295	2.402034	3.227984

O	-1.16256	2.534854	2.520019
O	0.372219	1.296451	3.615151
C	1.047281	5.992842	2.573305
O	0.425452	6.54021	3.491067
N	1.756926	6.699184	1.658939
H	2.356384	6.21092	0.993029
C	1.932056	8.138772	1.841823
H	2.226245	8.329194	2.879482
C	3.011021	8.682204	0.899428
H	3.974731	8.206838	1.103784
H	3.103501	9.760674	1.050979
H	2.753548	8.492934	-0.14831
C	0.629135	8.947395	1.669304
O	0.553415	10.08689	2.113688
N	-0.38085	8.331381	0.987236
H	-0.21085	7.40691	0.613023
C	-1.69072	8.930218	0.812292
H	-1.58347	9.966385	1.151717
C	-2.13622	8.95495	-0.67094
H	-1.45333	9.621853	-1.20892
H	-3.13242	9.404996	-0.68958
C	-2.16158	7.599295	-1.34963
C	-1.06043	7.152561	-2.09415
H	-0.18894	7.796114	-2.19563
C	-1.06704	5.906031	-2.72659
H	-0.19497	5.603773	-3.30125
C	-2.18875	5.081189	-2.61545
H	-2.21418	4.111423	-3.10864
C	-3.29539	5.508636	-1.87505
H	-4.17164	4.869544	-1.79109
C	-3.28235	6.757358	-1.24989
H	-4.1442	7.092868	-0.68006
C	-2.7937	8.314194	1.707354
O	-3.97328	8.613866	1.505123
N	-2.38229	7.494665	2.704022
H	-1.38798	7.341253	2.852621
C	-3.29862	6.963302	3.707247
H	-4.19722	7.584262	3.684803
C	-3.65233	5.476901	3.520611
H	-2.72464	4.888902	3.519209
H	-4.22069	5.161566	4.411018
C	-4.46388	5.118494	2.259949
H	-3.87807	5.433801	1.385969
C	-4.64003	3.593061	2.170101
H	-5.14929	3.3087	1.240167
H	-3.67415	3.077433	2.21453
H	-5.25172	3.239006	3.015994

C	-5.82365	5.830411	2.203245
H	-6.40029	5.491988	1.333163
H	-6.42039	5.610187	3.100519
H	-5.70278	6.914083	2.119793
C	-3.12437	-1.82935	-5.90109
H	-2.15983	-2.21261	-6.25322
C	-2.88169	-0.90508	-4.69562
H	-3.7217	-0.21972	-4.57618
H	-2.81482	-1.49146	-3.76941
C	-1.58908	-0.06905	-4.75554
O	-0.60854	-0.51559	-5.43662
O	-1.57345	0.994125	-4.07889
C	-4.04537	-3.0087	-5.61531
O	-5.15152	-3.14667	-6.13368
N	-3.55149	-3.95259	-4.73998
H	-2.64387	-3.80822	-4.30045
C	-4.12041	-5.28822	-4.71048
H	-4.50426	-5.53824	-5.70617
C	-5.27038	-5.55966	-3.73837
O	-5.63793	-6.72106	-3.54372
N	-5.84977	-4.48453	-3.14556
H	-5.43675	-3.57356	-3.30571
C	-6.95685	-4.62009	-2.20521
H	-7.34556	-5.63267	-2.35997
C	-6.51834	-4.43916	-0.74526
H	-7.40991	-4.52274	-0.11415
H	-6.14131	-3.42325	-0.60106
C	-5.46056	-5.38909	-0.2015
O	-4.68033	-5.00723	0.689176
N	-5.4437	-6.66237	-0.65187
H	-5.8131	-6.91222	-1.56855
H	-4.71576	-7.25718	-0.27543
C	-8.14365	-3.66764	-2.50152
O	-8.90724	-3.35781	-1.59208
N	-8.29398	-3.28598	-3.79401
H	-7.59739	-3.5706	-4.47237
C	-9.39344	-2.42917	-4.22952
H	-9.98761	-2.22436	-3.33677
C	-8.92681	-1.11339	-4.88872
H	-8.3587	-1.35601	-5.79717
H	-9.82905	-0.5841	-5.22084
C	-8.09577	-0.22812	-3.98793
C	-6.71182	-0.41701	-3.89772
H	-6.23691	-1.16732	-4.5272
C	-5.92731	0.355636	-3.04517
H	-4.86155	0.185801	-2.97044
C	-6.50499	1.374995	-2.27468

O	-5.76355	2.163138	-1.45188
H	-4.80798	1.934941	-1.55966
C	-7.88921	1.581153	-2.35699
H	-8.33865	2.374727	-1.7676
C	-8.66414	0.78288	-3.1983
H	-9.73676	0.960239	-3.24895
C	3.336442	6.916722	-3.78451
H	3.360011	7.237284	-2.73542
H	2.492065	7.400674	-4.2803
C	3.186848	5.398016	-3.80265
O	4.252252	4.720714	-3.61501
O	2.030443	4.933035	-3.97399
C	10.28381	-4.03525	0.155089
C	8.958266	-3.34823	0.096138
C	8.353476	-2.59884	-0.88336
N	8.060185	-3.33731	1.152594
C	6.97679	-2.61411	0.781317
N	7.120789	-2.14805	-0.44968
H	10.1855	-5.10562	0.377882
H	10.79481	-3.9422	-0.80741
H	8.72903	-2.35375	-1.86679
H	6.120992	-2.46037	1.423549
H	8.071109	-3.91769	1.991812
C	2.832119	-6.83347	-0.2627
H	3.208785	-7.06457	-1.26821
C	2.231123	-5.41323	-0.27324
H	3.004562	-4.68345	-0.53313
H	1.488516	-5.36843	-1.08184
C	1.550402	-5.02979	1.045918
H	0.663563	-5.66065	1.199901
H	2.225849	-5.22721	1.887892
C	1.141554	-3.55488	1.081595
H	2.044315	-2.93017	1.085756
H	0.565582	-3.28705	0.18952
N	0.336777	-3.29772	2.276144
H	0.223114	-4.05671	2.940593
C	-0.06298	-2.08755	2.707943
N	0.245003	-0.9596	2.044268
H	0.989785	-0.96483	1.347354
H	0.1689	-0.07294	2.595786
N	-0.79269	-2.03373	3.830557
H	-1.37802	-2.8523	4.062593
H	-1.18513	-1.13814	4.136399
C	3.970715	-7.06526	0.730544
O	3.907154	-7.96235	1.585275
N	5.053515	-6.25889	0.604189
H	5.084997	-5.55089	-0.14018

C	6.291997	-6.52281	1.32681
H	6.506825	-7.59831	1.294396
C	6.290187	-6.07271	2.801581
O	7.001304	-5.12707	3.202293
N	5.504878	-6.80754	3.60284
H	4.906424	-7.48615	3.122688
C	5.382973	-6.61871	5.047369
H	6.352768	-6.27547	5.419025
C	4.286542	-5.62493	5.475142
H	4.237696	-5.62563	6.571595
H	4.581915	-4.61984	5.15913
C	2.915569	-5.96538	4.888383
H	2.965736	-5.95533	3.795338
H	2.602588	-6.97343	5.186379
S	1.528873	-4.8843	5.437924
C	2.267023	-3.20921	5.350097
H	1.429139	-2.50959	5.396879
H	2.935014	-3.0276	6.195719
H	2.817121	-3.05401	4.41851
O	2.992665	-2.15826	-2.39156
O	5.424038	-2.81164	3.687766
Fe	5.238325	-1.7538	-1.54322
C	2.577744	-2.14526	-3.75476
H	3.376938	-2.68329	-4.2768
H	2.288263	-1.76671	-1.82817
C	1.29297	-3.01083	-3.91299
H	1.59688	-4.062	-3.85868
C	0.110694	-2.85783	-2.91014
O	0.269709	-2.1255	-1.90976
O	-0.91607	-3.53175	-3.2243
N	0.639159	-2.8275	-5.26474
H	0.125637	-1.85848	-5.3505
H	-0.10445	-3.53241	-5.31233
H	1.286376	-2.95036	-6.04772
C	2.53351	-0.72843	-4.34176
H	2.193557	-0.76515	-5.38634
H	3.571446	-0.37854	-4.37127
C	1.67735	0.262855	-3.56126
H	2.029228	0.322904	-2.52986
H	0.643272	-0.06935	-3.51548
C	1.710318	1.637701	-4.20718
H	2.71284	2.071496	-4.22505
H	1.334916	1.590479	-5.23242
N	0.835407	2.590063	-3.46139
H	7.093557	-5.98569	0.819463
H	5.188062	-7.607	5.478935
H	-3.7975	-7.16883	4.026868

H	2.066166	-7.57641	-0.02063
H	-8.24188	-0.9161	3.293618
H	-5.88675	1.948212	5.617574
H	10.93657	-3.59936	0.922088
H	8.9652	3.015469	-2.88601
H	4.276114	7.225673	-4.25284
H	5.714603	6.868412	-1.13668
H	-2.82509	7.093592	4.687803
H	-3.57049	-1.27095	-6.72746
H	-3.33543	-6.00937	-4.46691
H	-10.0227	-2.98644	-4.9362
H	6.014654	-3.59194	3.646564
H	5.872178	-2.19304	4.284392
C	5.604597	-4.14784	-2.56628
O	5.163467	-4.24964	-1.38961
O	5.732251	-3.00798	-3.12967
C	6.042128	-5.37874	-3.33953
H	5.57325	-6.28272	-2.94258
H	7.129795	-5.48458	-3.24681
H	5.813755	-5.26796	-4.40294
H	1.176854	3.57249	-3.6748
H	-0.14223	2.393263	-3.72826
H	0.91746	2.436795	-2.42845
O	1.001738	2.055546	-0.68236
H	1.519915	1.255433	-0.42375
H	0.088349	1.843661	-0.35244
O	-3.13153	1.277661	-1.7391
H	-2.63344	1.287846	-2.59491
H	-2.49895	1.531273	-1.02293
O	-1.38532	1.255085	0.335567
H	-1.22374	0.312799	0.522372
H	-1.38067	1.715676	1.24953

^{5}**TS1**$_{B,C4}$

C	-3.61334	-2.5395	6.160456
H	-3.18056	-1.99238	5.312639
C	-2.48222	-3.14711	6.997092
H	-2.86833	-3.45897	7.974374
H	-2.12647	-4.05253	6.50064
C	-1.30341	-2.1892	7.20488
H	-1.63036	-1.25514	7.681778
H	-0.57885	-2.64431	7.894233
C	-0.55741	-1.85529	5.908905
O	-0.71364	-2.53168	4.878563
N	0.281725	-0.80722	5.951465
H	0.384416	-0.2523	6.788525
H	0.789391	-0.52715	5.100909
C	-4.55472	-3.58028	5.553718
O	-4.29246	-4.78964	5.536442
N	-5.68172	-3.07576	4.988283
H	-5.78646	-2.06659	4.94426
C	-6.52762	-3.87937	4.103932
H	-7.48231	-3.35388	3.999738
C	-5.87953	-4.11589	2.725347
H	-4.95594	-4.68394	2.880782
H	-6.54326	-4.75511	2.127928
C	-5.56523	-2.80035	1.99546
H	-5.26702	-2.05073	2.734059
H	-6.46348	-2.39762	1.511602
C	-4.41224	-2.85145	0.995418
H	-4.20234	-1.83341	0.661503
H	-3.50829	-3.23687	1.49069
N	-4.71439	-3.67871	-0.17554
H	-5.32115	-4.49398	-0.0723
C	-4.04766	-3.60237	-1.33651
N	-3.19442	-2.6082	-1.60494
H	-3.1736	-1.7114	-1.08226
H	-2.79109	-2.63239	-2.53958
N	-4.21832	-4.57384	-2.25588
H	-4.90257	-5.29883	-2.06916
H	-3.89137	-4.38355	-3.20388
C	-1.7582	-7.04996	0.135693
H	-0.75056	-7.46741	0.018058
H	-1.88478	-6.27334	-0.62927
C	-1.96371	-6.50318	1.514408
N	-1.13308	-5.53174	2.052005
C	-1.60337	-5.28971	3.271603
H	-1.21359	-4.56388	3.975117
N	-2.6856	-6.05318	3.546577

H	-3.28596	-5.91178	4.361102
C	-2.93314	-6.82939	2.432825
H	-3.74954	-7.53465	2.393523
C	6.713325	4.328035	-5.35761
C	6.606652	4.666879	-3.86827
C	5.427224	3.810527	-6.05181
O	7.408653	4.183452	-3.05935
C	4.758171	2.688618	-5.31395
C	4.982487	1.332162	-5.22904
N	3.744721	2.954997	-4.41861
C	3.40113	1.803215	-3.83028
N	4.120612	0.783806	-4.29825
H	7.066326	5.219561	-5.89313
H	4.705355	4.626736	-6.17122
H	5.700372	3.488626	-7.06334
H	5.707165	0.728143	-5.75405
H	2.665714	1.724005	-3.0506
H	3.293731	3.91917	-4.28657
N	5.65339	5.560191	-3.49343
H	4.816315	5.725063	-4.05523
C	5.534017	5.97996	-2.10175
H	6.542337	6.10393	-1.6958
C	4.733907	5.009238	-1.216
O	3.629405	5.325608	-0.76055
N	5.338479	3.823022	-0.9837
C	4.719757	2.706864	-0.26595
C	4.111545	3.194506	1.072672
C	5.808882	1.657826	0.016564
O	4.814485	3.666851	1.960418
C	5.291447	0.325005	0.572359
C	4.737706	-0.69266	-0.43191
O	4.461976	-1.84275	-0.00948
O	4.638	-0.31351	-1.64845
H	6.232563	3.671295	-1.44906
H	3.933907	2.266255	-0.89027
H	6.364305	1.4681	-0.90882
H	6.49944	2.103247	0.740779
H	4.513385	0.485572	1.331071
H	6.101221	-0.19419	1.099949
N	2.763854	3.045549	1.173269
H	2.26742	2.559834	0.42909
C	2.004989	3.404836	2.366783
H	0.951893	3.38752	2.061628
C	2.123718	2.359567	3.484673
H	3.159295	2.037365	3.614759
H	1.80472	2.827208	4.42339
C	1.211038	1.162866	3.202183

O	0.099082	1.411072	2.661721
O	1.616534	0.001696	3.544144
C	2.232502	4.837969	2.875656
O	1.986815	5.121924	4.053455
N	2.541143	5.789423	1.956683
H	2.866283	5.505023	1.032057
C	2.868364	7.140356	2.413973
H	3.497028	7.060439	3.307676
C	3.626894	7.918998	1.334641
H	4.57622	7.426574	1.105102
H	3.825663	8.928618	1.702641
H	3.044293	7.987355	0.409671
C	1.64869	7.952634	2.890917
O	1.813268	8.982974	3.534636
N	0.421861	7.463152	2.546868
H	0.387559	6.622064	1.985603
C	-0.81526	8.046037	3.029743
H	-0.51881	8.978436	3.523335
C	-1.79124	8.40393	1.882788
H	-1.31392	9.183108	1.278052
H	-2.67972	8.836576	2.350375
C	-2.18643	7.240052	0.995797
C	-1.4725	6.953595	-0.17714
H	-0.63188	7.58477	-0.4574
C	-1.83511	5.884484	-1.00175
H	-1.2631	5.693842	-1.90714
C	-2.92508	5.079121	-0.66329
H	-3.22287	4.249737	-1.30056
C	-3.64164	5.351382	0.505953
H	-4.49387	4.732274	0.775796
C	-3.2776	6.419617	1.326992
H	-3.83563	6.629088	2.234927
C	-1.51967	7.202687	4.120787
O	-2.67918	7.472089	4.445468
N	-0.78019	6.224663	4.696106
H	0.182724	6.077728	4.402431
C	-1.2588	5.440533	5.828154
H	-2.11008	5.976019	6.254225
C	-1.63559	3.991689	5.471881
H	-0.75795	3.505216	5.025184
H	-1.84179	3.461163	6.415324
C	-2.83743	3.815679	4.523866
H	-2.61323	4.360089	3.59645
C	-3.00034	2.331138	4.15404
H	-3.80328	2.219072	3.411329
H	-2.08117	1.917346	3.725379
H	-3.26313	1.737367	5.043811

C	-4.13971	4.38672	5.104686
H	-4.98488	4.183715	4.434677
H	-4.37271	3.928142	6.076373
H	-4.07492	5.470699	5.235451
C	-5.80722	0.629602	-4.67936
H	-5.10642	0.381801	-5.47895
C	-5.0365	0.659337	-3.34893
H	-5.63481	1.112308	-2.55369
H	-4.81188	-0.36723	-3.02277
C	-3.68129	1.384017	-3.43585
O	-3.10474	1.433146	-4.56303
O	-3.20648	1.833725	-2.35088
C	-6.95799	-0.36121	-4.6637
O	-8.10279	-0.05166	-4.31254
N	-6.63706	-1.63836	-5.03304
H	-5.67709	-1.84295	-5.33084
C	-7.66677	-2.60747	-5.36169
H	-8.45536	-2.13196	-5.95843
C	-8.39868	-3.28921	-4.2052
O	-9.21072	-4.18881	-4.43971
N	-8.13076	-2.8418	-2.95287
H	-7.42747	-2.12241	-2.85057
C	-8.95689	-3.20127	-1.80886
H	-9.8206	-3.73064	-2.22529
C	-8.21931	-4.1074	-0.812
H	-8.9221	-4.38399	-0.01847
H	-7.41683	-3.54237	-0.33208
C	-7.58993	-5.37221	-1.37655
O	-6.5418	-5.82637	-0.88037
N	-8.22903	-6.01138	-2.37954
H	-8.8772	-5.52786	-3.00301
H	-7.7667	-6.83223	-2.75073
C	-9.5345	-1.98172	-1.04877
O	-10.0282	-2.1603	0.062076
N	-9.47616	-0.78571	-1.68274
H	-9.10667	-0.71583	-2.62881
C	-10.0542	0.420976	-1.09967
H	-10.7002	0.099241	-0.28025
C	-9.01106	1.446129	-0.61475
H	-8.35249	1.695541	-1.45601
H	-9.56138	2.36881	-0.37746
C	-8.17103	1.03931	0.583468
C	-6.77583	1.142822	0.546458
H	-6.28365	1.474721	-0.36445
C	-5.98253	0.8373	1.655116
H	-4.89985	0.889253	1.569269
C	-6.59351	0.429248	2.842381

O	-5.86532	0.095896	3.97068
H	-4.98473	0.503192	3.904075
C	-7.98412	0.307183	2.904896
H	-8.44801	-0.00965	3.8343
C	-8.75644	0.607544	1.784674
H	-9.83567	0.505013	1.855137
C	1.486136	7.025459	-3.12897
H	1.866819	7.09579	-2.1025
H	0.411631	7.226532	-3.11619
C	1.779483	5.616468	-3.63043
O	2.902027	5.439252	-4.21544
O	0.941141	4.703306	-3.42645
C	8.363683	-2.77783	-6.29801
C	7.445744	-2.37552	-5.19136
C	6.148926	-1.93534	-5.18501
N	7.817571	-2.38273	-3.85406
C	6.77853	-1.95896	-3.10025
N	5.750134	-1.67451	-3.88443
H	8.715007	-3.81096	-6.18291
H	7.842148	-2.70927	-7.2564
H	5.469147	-1.80402	-6.01254
H	6.808687	-1.94398	-2.01996
H	8.707942	-2.68102	-3.48068
C	5.525972	-6.94226	0.461444
H	6.206649	-7.30467	-0.32277
C	4.624077	-5.8505	-0.1377
H	5.216303	-4.9905	-0.46941
H	4.174445	-6.26384	-1.05101
C	3.499455	-5.37971	0.795284
H	2.963112	-6.24907	1.200739
H	3.918249	-4.84115	1.655204
C	2.509118	-4.47275	0.048678
H	3.012185	-3.58556	-0.3418
H	2.105276	-5.00125	-0.82145
N	1.360701	-4.06065	0.858735
H	0.603335	-4.73013	1.022057
C	1.274542	-2.95211	1.601191
N	2.264114	-2.08997	1.783213
H	3.145649	-2.14313	1.264311
H	2.066443	-1.24662	2.345618
N	0.050119	-2.66972	2.153172
H	-0.6062	-3.44403	2.075861
H	0.038137	-2.26029	3.095143
C	6.404231	-6.59716	1.665424
O	6.502901	-7.36665	2.618386
N	7.131515	-5.4373	1.583167
H	7.056699	-4.81869	0.77426

C	8.181327	-5.18638	2.547534
H	8.02081	-5.88841	3.369497
C	8.256063	-3.74006	3.037595
O	8.110685	-2.76405	2.291549
N	8.596562	-3.59828	4.340181
H	8.645907	-4.42739	4.914325
C	8.889922	-2.29836	4.951764
H	9.556278	-1.75386	4.274972
C	7.645017	-1.4425	5.229417
H	7.973842	-0.46808	5.609818
H	7.149468	-1.25563	4.2726
C	6.665873	-2.07256	6.224309
H	6.419363	-3.10192	5.937604
H	7.103736	-2.10999	7.228908
S	5.100023	-1.1309	6.405788
C	4.301255	-1.54318	4.814742
H	3.42691	-0.89856	4.697045
H	4.973997	-1.34817	3.974339
H	3.985869	-2.59132	4.79274
O	2.252254	-0.71581	-2.74733
O	6.99251	-3.08793	-0.21047
Fe	3.888069	-1.12004	-3.30064
C	-0.28927	-1.82096	-4.72955
H	0.467247	-2.46788	-5.18121
H	-0.65224	-2.33814	-3.83604
C	-1.49403	-1.74496	-5.72327
H	-1.21293	-2.24993	-6.65386
C	-2.83558	-2.42046	-5.25366
O	-2.74475	-3.29441	-4.36383
O	-3.85121	-2.00145	-5.87064
N	-1.91929	-0.36103	-6.16815
H	-2.2864	0.284807	-5.4009
H	-2.76609	-0.5441	-6.73088
H	-1.20836	0.132618	-6.71196
C	0.358852	-0.51148	-4.34615
H	0.896938	-0.00453	-5.15388
H	1.476449	-0.76247	-3.55852
C	-0.40458	0.378753	-3.3912
H	-0.10297	0.142389	-2.36503
H	-1.47458	0.159093	-3.42418
C	-0.18703	1.866809	-3.65768
H	0.838133	2.099854	-3.93551
H	-0.85444	2.222863	-4.44322
N	-0.49447	2.669378	-2.43883
H	9.171561	-5.41244	2.117091
H	9.442343	-2.4995	5.876139
H	-2.47759	-7.84685	-0.07721

H	4.925224	-7.80107	0.776172
H	-6.71976	-4.83582	4.597117
H	-4.19273	-1.80929	6.740729
H	9.247122	-2.12917	-6.35226
H	7.498554	3.570806	-5.42367
H	2.000976	7.775165	-3.73594
H	5.011507	6.936886	-2.07517
H	-0.46258	5.422938	6.582254
H	-6.23033	1.616523	-4.88724
H	-7.22165	-3.39481	-5.97517
H	-10.6856	0.896246	-1.85945
H	7.470505	-2.82358	0.609002
H	6.067885	-2.79638	-0.05238
C	3.198122	-3.35503	-4.23492
O	3.131964	-2.22724	-4.85453
O	3.604919	-3.39664	-3.04999
C	2.826644	-4.61841	-4.98166
H	2.433635	-5.36592	-4.28879
H	3.730161	-5.0295	-5.44825
H	2.100988	-4.41448	-5.77277
H	-0.17074	3.647484	-2.61483
H	-1.51965	2.585685	-2.25717
H	0.058333	2.276878	-1.65018
O	1.30946	1.149043	-0.73698
H	1.760214	0.501769	-1.32587
H	0.698696	0.604154	-0.17318
O	-3.14138	-0.12051	-0.32406
H	-3.23587	0.633176	-0.95053
H	-2.24316	-0.03654	0.085656
O	-0.61303	-0.15446	0.731311
H	-0.47099	-1.06887	1.058232
H	-0.45675	0.413455	1.555698

^{5}**IM1**$_{B,C4}$

C	-3.28379	-2.40628	5.699482
H	-2.77307	-2.0332	4.801688
C	-2.25826	-3.07493	6.620197
H	-2.72478	-3.31163	7.58374
H	-1.95481	-4.02875	6.182362
C	-1.01619	-2.2106	6.867671
H	-1.294	-1.24046	7.301336
H	-0.37269	-2.70271	7.610098
C	-0.16585	-1.97433	5.615318
O	-0.27485	-2.69662	4.611276
N	0.722141	-0.96791	5.680611
H	0.756082	-0.35823	6.484564
H	1.265124	-0.7091	4.845835
C	-4.38082	-3.35326	5.214618
O	-4.2714	-4.58542	5.2697
N	-5.47009	-2.74832	4.676049
H	-5.45586	-1.73977	4.549994
C	-6.4744	-3.49068	3.913783
H	-7.32911	-2.81998	3.779227
C	-5.95629	-3.98953	2.549244
H	-5.22219	-4.78303	2.726171
H	-6.79643	-4.44618	2.00836
C	-5.30363	-2.87327	1.722905
H	-4.6124	-2.3196	2.36819
H	-6.05804	-2.1478	1.391002
C	-4.47923	-3.33585	0.521712
H	-3.91372	-2.48091	0.142276
H	-3.75736	-4.10483	0.8319
N	-5.32647	-3.8558	-0.55413
H	-6.20519	-4.32226	-0.32289
C	-4.98098	-3.87562	-1.84784
N	-3.86585	-3.29439	-2.30186
H	-3.34393	-2.57805	-1.77554
H	-3.76478	-3.30391	-3.31619
N	-5.76145	-4.53566	-2.72799
H	-6.60763	-4.97236	-2.38117
H	-5.65509	-4.30434	-3.71562
C	-1.391	-7.21084	0.138314
H	-0.42283	-7.72493	0.192672
H	-1.32273	-6.46676	-0.6652
C	-1.74388	-6.57016	1.444763
N	-0.96189	-5.57564	2.008213
C	-1.55579	-5.24806	3.147584
H	-1.21852	-4.48292	3.835936
N	-2.67499	-5.98064	3.352279

H	-3.33162	-5.80181	4.113071
C	-2.81435	-6.82662	2.270414
H	-3.63119	-7.5279	2.188894
C	7.244425	4.329601	-4.99709
C	6.763805	4.773101	-3.61268
C	6.234604	3.572394	-5.89265
O	7.472491	4.5683	-2.6172
C	5.587122	2.403363	-5.2087
C	6.034967	1.165473	-4.81092
N	4.292577	2.497106	-4.74459
C	3.996124	1.370819	-4.08684
N	5.031189	0.526933	-4.10484
H	7.593667	5.220413	-5.53694
H	5.44684	4.250432	-6.23864
H	6.769865	3.240182	-6.79026
H	6.994433	0.697377	-4.97181
H	3.062298	1.192446	-3.58304
H	3.695464	3.369497	-4.8407
N	5.591825	5.452127	-3.53603
H	4.874795	5.398711	-4.26382
C	5.132056	6.015407	-2.27142
H	5.999768	6.422931	-1.74311
C	4.398686	5.017473	-1.3575
O	3.200385	5.156288	-1.08929
N	5.174547	4.018785	-0.88103
C	4.674253	2.886208	-0.10213
C	3.77191	3.377377	1.051841
C	5.879096	2.146112	0.507756
O	4.22923	4.063457	1.96268
C	5.551036	0.749482	1.052153
C	5.476438	-0.3686	0.013304
O	5.32648	-1.55512	0.388912
O	5.626368	-0.01424	-1.21272
H	6.142985	4.006939	-1.20001
H	4.110046	2.214417	-0.76033
H	6.662763	2.057441	-0.25244
H	6.264729	2.775125	1.316358
H	4.603029	0.741577	1.607843
H	6.310613	0.441662	1.781483
N	2.475364	2.97737	0.9992
H	2.168241	2.350806	0.255394
C	1.54718	3.266449	2.09007
H	0.545665	3.048377	1.701983
C	1.767875	2.325179	3.281725
H	2.814137	2.337381	3.594196
H	1.166422	2.681989	4.124545
C	1.318019	0.907177	2.918369

O	0.23882	0.807046	2.281959
O	2.052541	-0.06626	3.304939
C	1.525696	4.745973	2.513615
O	1.247438	5.059914	3.674212
N	1.675594	5.678336	1.529248
H	2.083798	5.376911	0.644547
C	1.884807	7.080027	1.906143
H	2.584587	7.115247	2.749096
C	2.469571	7.876422	0.735041
H	3.443561	7.47367	0.445535
H	2.58589	8.918891	1.041311
H	1.809825	7.837125	-0.13911
C	0.623528	7.791294	2.4317
O	0.725224	8.869479	3.007386
N	-0.56545	7.167075	2.19803
H	-0.54706	6.286911	1.698848
C	-1.82848	7.655581	2.717669
H	-1.63055	8.678669	3.056525
C	-2.92427	7.703545	1.625101
H	-2.63061	8.462959	0.891808
H	-3.84322	8.039128	2.112961
C	-3.14608	6.37541	0.931134
C	-2.45241	6.056437	-0.2462
H	-1.78818	6.795339	-0.68981
C	-2.60473	4.807657	-0.85347
H	-2.05619	4.577328	-1.76357
C	-3.46005	3.855008	-0.29721
H	-3.55568	2.880738	-0.76728
C	-4.16994	4.168178	0.865096
H	-4.84731	3.437452	1.299809
C	-4.01412	5.414503	1.474302
H	-4.55644	5.651382	2.385233
C	-2.32655	6.894836	3.972474
O	-3.48573	7.053768	4.365803
N	-1.41118	6.12217	4.602426
H	-0.47909	6.015411	4.208147
C	-1.68397	5.452811	5.86611
H	-2.64806	5.82509	6.218371
C	-1.67147	3.918973	5.761551
H	-0.68303	3.612815	5.39364
H	-1.76426	3.512065	6.780976
C	-2.75605	3.287753	4.867682
H	-2.6684	3.730667	3.8664
C	-2.51338	1.775896	4.7327
H	-3.29995	1.299864	4.135601
H	-1.5582	1.559242	4.241512
H	-2.50597	1.293442	5.722234

C	-4.17514	3.579477	5.381387
H	-4.92473	3.099154	4.737275
H	-4.31243	3.200509	6.405079
H	-4.39341	4.6521	5.380881
C	-5.9501	1.020445	-4.34458
H	-5.53253	0.609634	-5.26323
C	-4.99049	0.754404	-3.17426
H	-5.3238	1.273845	-2.27124
H	-4.98141	-0.31861	-2.93022
C	-3.5315	1.159101	-3.45728
O	-3.14761	1.27514	-4.65977
O	-2.78768	1.32184	-2.44829
C	-7.33703	0.451178	-4.10841
O	-8.12174	0.957464	-3.28457
N	-7.65969	-0.64891	-4.83365
H	-6.93709	-1.08095	-5.42219
C	-9.02029	-1.15186	-4.95445
H	-9.70163	-0.31038	-5.14229
C	-9.63125	-1.92558	-3.77914
O	-10.5188	-2.75809	-3.9876
N	-9.19821	-1.59105	-2.53691
H	-8.52958	-0.82916	-2.47909
C	-9.92303	-1.97137	-1.33022
H	-10.8776	-2.39254	-1.6636
C	-9.16764	-3.01275	-0.49218
H	-9.74236	-3.1887	0.424457
H	-8.19822	-2.60957	-0.18219
C	-8.89827	-4.35878	-1.14921
O	-7.94118	-5.06105	-0.77311
N	-9.75428	-4.79502	-2.09752
H	-10.3197	-4.15385	-2.65785
H	-9.5325	-5.69025	-2.51582
C	-10.3093	-0.7671	-0.43328
O	-10.7567	-0.98685	0.687853
N	-10.1662	0.469221	-0.97527
H	-9.75952	0.581762	-1.89798
C	-10.5079	1.686212	-0.24967
H	-11.0053	1.37546	0.671463
C	-9.27977	2.570832	0.056254
H	-8.74637	2.767425	-0.88186
H	-9.65455	3.538373	0.414373
C	-8.33182	1.982039	1.078193
C	-7.19304	1.263965	0.689239
H	-6.97608	1.14943	-0.37014
C	-6.31675	0.720293	1.630893
H	-5.41305	0.204521	1.322887
C	-6.58656	0.878541	2.991373

O	-5.71121	0.308364	3.903848
H	-5.72809	0.833528	4.720638
C	-7.71561	1.588871	3.404459
H	-7.92461	1.713997	4.464651
C	-8.57121	2.136979	2.450047
H	-9.44104	2.698096	2.783703
C	1.2565	6.009844	-3.91045
H	1.519063	6.188777	-2.86067
H	0.173655	5.869327	-3.97541
C	1.995221	4.757139	-4.36151
O	3.143026	4.904581	-4.89989
O	1.474249	3.628497	-4.15878
C	10.19031	-2.84616	-4.60567
C	9.082869	-2.16226	-3.8746
C	7.722153	-2.22037	-4.00354
N	9.301357	-1.26466	-2.83547
C	8.111893	-0.83218	-2.37313
N	7.129868	-1.38749	-3.06777
H	10.79355	-3.47349	-3.93743
H	9.775758	-3.4929	-5.3834
H	7.116451	-2.79805	-4.68201
H	7.984611	-0.14297	-1.55667
H	10.19643	-1.03432	-2.42724
C	6.771388	-6.42683	-0.29687
H	7.61219	-6.38405	-1.00359
C	5.774938	-5.30815	-0.64645
H	6.274894	-4.33425	-0.62306
H	5.459637	-5.45899	-1.68885
C	4.53469	-5.27046	0.256158
H	4.083617	-6.27104	0.318009
H	4.822575	-4.99208	1.277597
C	3.496586	-4.27001	-0.26867
H	3.935492	-3.27455	-0.36135
H	3.162315	-4.55102	-1.27367
N	2.302794	-4.17774	0.572721
H	1.618353	-4.91985	0.50055
C	2.003096	-3.20028	1.447181
N	2.845273	-2.23008	1.773567
H	3.762123	-2.12316	1.334723
H	2.48847	-1.42965	2.334514
N	0.746722	-3.19586	1.963042
H	0.19351	-4.05845	1.840703
H	0.594762	-2.74795	2.874534
C	7.35524	-6.42527	1.116802
O	7.339973	-7.43531	1.817583
N	7.931677	-5.25856	1.540747
H	8.000822	-4.44633	0.922745

C	8.657575	-5.261	2.792776
H	8.154877	-5.96001	3.467427
C	8.80132	-3.87662	3.419571
O	8.921177	-2.838	2.761496
N	8.882371	-3.87567	4.772594
H	8.736808	-4.74565	5.264034
C	9.192773	-2.66961	5.545397
H	10.02692	-2.1617	5.050825
C	8.014812	-1.69196	5.678277
H	8.37152	-0.79014	6.190271
H	7.718168	-1.3869	4.670583
C	6.815435	-2.26565	6.437887
H	6.505026	-3.22798	6.013092
H	7.071676	-2.44092	7.489668
S	5.356549	-1.15094	6.469364
C	4.790838	-1.32549	4.741095
H	3.948285	-0.64695	4.586764
H	5.579043	-1.05487	4.032413
H	4.457945	-2.34897	4.541105
O	3.434767	-0.99734	-2.22026
O	8.005312	-2.62581	0.149503
Fe	5.142517	-1.08871	-2.76309
C	-2.04491	-1.99215	-4.97949
H	-1.9731	-3.07241	-4.77504
H	-2.51653	-1.54812	-4.09669
C	-3.01248	-1.87665	-6.1674
H	-2.64774	-2.46352	-7.01841
C	-4.46634	-2.3529	-5.82066
O	-4.55199	-3.35569	-5.07877
O	-5.38542	-1.65861	-6.3384
N	-3.15589	-0.45704	-6.64824
H	-3.20941	0.239038	-5.82414
H	-4.07923	-0.43978	-7.1153
H	-2.40078	-0.16341	-7.26976
C	-0.68471	-1.39018	-5.14695
H	-0.08529	-1.65986	-6.01612
H	2.964107	-1.75763	-2.64383
C	-0.05981	-0.60783	-4.02983
H	0.945399	-0.98914	-3.80749
H	-0.66159	-0.74616	-3.12265
C	0.025169	0.907075	-4.33504
H	0.944943	1.176838	-4.85639
H	-0.83058	1.228026	-4.92929
N	-0.03468	1.726045	-3.08378
H	9.681486	-5.65134	2.658777
H	9.538694	-3.00154	6.530485
H	-2.14477	-7.94932	-0.15216

H	6.301131	-7.40765	-0.41568
H	-6.80581	-4.34137	4.515803
H	-3.73523	-1.53	6.183133
H	10.86482	-2.12988	-5.0916
H	8.118773	3.701032	-4.80933
H	1.558671	6.880435	-4.49871
H	4.430824	6.82127	-2.4914
H	-0.92191	5.762614	6.593217
H	-6.06598	2.101117	-4.48069
H	-9.06576	-1.81203	-5.82194
H	-11.2247	2.262179	-0.84874
H	8.411213	-2.50259	1.034316
H	7.072312	-2.3536	0.270403
C	3.810743	-3.34775	-4.12477
O	4.893968	-2.67902	-3.83345
O	2.692662	-3.16894	-3.62455
C	4.030261	-4.42786	-5.17338
H	3.104942	-4.97895	-5.35035
H	4.815707	-5.11651	-4.84363
H	4.370804	-3.96925	-6.10849
H	0.319708	2.684691	-3.3222
H	-1.02674	1.733427	-2.75452
H	0.584426	1.343797	-2.32659
O	1.63219	0.718763	-1.03797
H	2.351298	0.146305	-1.40547
H	1.048025	0.100556	-0.53083
O	-2.52753	-1.12201	-1.1068
H	-2.70986	-0.21326	-1.43263
H	-1.71125	-1.0439	-0.55109
O	-0.20179	-0.87556	0.340092
H	0.035098	-1.74945	0.711594
H	-0.14899	-0.2695	1.144795

[5]**TS2**$_{B,C4}$

C	-4.07911	-1.25391	6.227831
H	-3.5287	-0.90156	5.345525
C	-3.10312	-1.95104	7.18128
H	-3.57314	-2.08518	8.162429
H	-2.89424	-2.95219	6.797746
C	-1.78343	-1.1905	7.356115
H	-1.9599	-0.16934	7.720775
H	-1.17447	-1.68599	8.124895
C	-0.94524	-1.12755	6.074901
O	-1.17871	-1.86903	5.107595
N	0.064233	-0.23878	6.061082
H	0.232792	0.377648	6.842417
H	0.636148	-0.14205	5.212417
C	-5.18054	-2.17159	5.697054
O	-5.13731	-3.40349	5.809764
N	-6.18961	-1.53878	5.045106
H	-6.1087	-0.5371	4.896041
C	-7.14569	-2.26723	4.209082
H	-7.98797	-1.59498	4.016512
C	-6.52	-2.75424	2.886407
H	-5.72534	-3.46702	3.132139
H	-7.27841	-3.31251	2.32129
C	-5.94139	-1.59824	2.055557
H	-5.50872	-0.86018	2.737404
H	-6.73544	-1.06936	1.514219
C	-4.81059	-1.96016	1.09471
H	-4.37952	-1.03168	0.71663
H	-4.02129	-2.50447	1.634434
N	-5.26397	-2.76225	-0.04474
H	-6.01546	-3.44182	0.085575
C	-4.5803	-2.87547	-1.19382
N	-3.54529	-2.08085	-1.48684
H	-3.37889	-1.1691	-1.02042
H	-3.13204	-2.23435	-2.40442
N	-4.92678	-3.83606	-2.07244
H	-5.72197	-4.4273	-1.85779
H	-4.55329	-3.76288	-3.01941
C	-2.87426	-6.51105	0.732286
H	-1.99884	-7.17207	0.709374
H	-2.75845	-5.78512	-0.0822
C	-3.01008	-5.82957	2.058479
N	-2.01644	-5.00707	2.567175
C	-2.46565	-4.58296	3.744127
H	-1.95998	-3.89391	4.409841
N	-3.68895	-5.09015	4.020081

H	-4.2776	-4.76766	4.790781
C	-4.05419	-5.88272	2.950925
H	-4.99516	-6.41118	2.922695
C	7.488069	2.511558	-5.53452
C	7.405939	3.001393	-4.08648
C	6.151931	2.216301	-6.26003
O	8.105449	2.476651	-3.21113
C	5.246601	1.291605	-5.49995
C	5.22754	-0.07099	-5.30401
N	4.229119	1.797529	-4.71755
C	3.64674	0.773805	-4.08585
N	4.213826	-0.38894	-4.41896
H	8.043809	3.259005	-6.11685
H	5.61062	3.148403	-6.45804
H	6.393278	1.786207	-7.23901
H	5.875951	-0.8314	-5.71294
H	2.854767	0.879661	-3.36615
H	3.969165	2.841248	-4.66111
N	6.60605	4.068703	-3.82639
H	5.819643	4.319711	-4.42818
C	6.54795	4.642405	-2.48685
H	7.559489	4.638525	-2.07024
C	5.588934	3.910046	-1.5334
O	4.539637	4.441855	-1.15341
N	5.991945	2.676442	-1.15834
C	5.181687	1.752811	-0.36143
C	4.629856	2.466907	0.897232
C	6.075436	0.576528	0.069032
O	5.379168	2.903285	1.76528
C	5.315351	-0.61034	0.673155
C	4.6272	-1.55584	-0.31507
O	4.090618	-2.60155	0.135872
O	4.675237	-1.25125	-1.55366
H	6.853031	2.332703	-1.58179
H	4.349976	1.382178	-0.9725
H	6.643571	0.227552	-0.80046
H	6.786898	0.965208	0.805348
H	4.554409	-0.27156	1.389401
H	6.002515	-1.23376	1.259298
N	3.27425	2.552572	0.958587
H	2.721014	2.096401	0.236566
C	2.563571	3.155736	2.081848
H	1.529833	3.287828	1.740823
C	2.475475	2.225386	3.300122
H	3.432834	1.735978	3.492626
H	2.238153	2.836867	4.178377
C	1.360367	1.192788	3.112741

O	0.322985	1.580965	2.509886
O	1.535804	0.021447	3.589235
C	3.028398	4.572277	2.461277
O	2.822087	5.005719	3.600282
N	3.50813	5.365411	1.467836
H	3.792933	4.940765	0.584621
C	4.072612	6.671067	1.812663
H	4.680129	6.558519	2.717398
C	4.953985	7.206391	0.679743
H	5.799082	6.534896	0.503254
H	5.331879	8.191901	0.963064
H	4.388737	7.299247	-0.25389
C	3.019685	7.72627	2.201835
O	3.366726	8.757577	2.766514
N	1.72601	7.444007	1.870465
H	1.540214	6.580428	1.37726
C	0.615511	8.284001	2.275974
H	1.077096	9.179604	2.706959
C	-0.26077	8.728562	1.080046
H	0.361798	9.35682	0.43334
H	-1.05865	9.353204	1.490511
C	-0.85404	7.5929	0.270162
C	-0.19772	7.101253	-0.86778
H	0.741998	7.552907	-1.17833
C	-0.74067	6.05547	-1.62
H	-0.20962	5.702423	-2.50128
C	-1.95604	5.479836	-1.24256
H	-2.39312	4.671175	-1.82324
C	-2.61729	5.957943	-0.10729
H	-3.56608	5.51921	0.192086
C	-2.07396	7.002868	0.641127
H	-2.58994	7.372511	1.522423
C	-0.24892	7.675591	3.407035
O	-1.34243	8.179914	3.67638
N	0.286887	6.627295	4.076582
H	1.209845	6.282039	3.822958
C	-0.34572	6.041458	5.252286
H	-1.09525	6.754614	5.602129
C	-0.97084	4.657537	5.003256
H	-0.1878	3.983481	4.630564
H	-1.29039	4.2574	5.978795
C	-2.16407	4.617746	4.029177
H	-1.82208	5.024116	3.067487
C	-2.59158	3.160287	3.783715
H	-3.38587	3.128171	3.024266
H	-1.75614	2.547824	3.427741
H	-2.97773	2.709741	4.711779

C	-3.34987	5.468434	4.508409
H	-4.20502	5.359395	3.829269
H	-3.68201	5.15434	5.508304
H	-3.08966	6.530259	4.544169
C	-5.64264	1.449398	-4.70812
H	-5.03631	1.134381	-5.56093
C	-4.80299	1.274512	-3.43122
H	-5.27473	1.76411	-2.57504
H	-4.72687	0.206633	-3.18001
C	-3.35745	1.782527	-3.57368
O	-2.81236	1.697937	-4.7149
O	-2.78994	2.197858	-2.52037
C	-6.93972	0.658722	-4.67228
O	-8.02659	1.166214	-4.37192
N	-6.82278	-0.67168	-4.9682
H	-5.90428	-1.04446	-5.23233
C	-7.99011	-1.47514	-5.28185
H	-8.6811	-0.90579	-5.91622
C	-8.84092	-1.97555	-4.11448
O	-9.78398	-2.74287	-4.32747
N	-8.52496	-1.51639	-2.87762
H	-7.70864	-0.92607	-2.78749
C	-9.41215	-1.68996	-1.73687
H	-10.3394	-2.10362	-2.1476
C	-8.83554	-2.64782	-0.68399
H	-9.58453	-2.77953	0.104498
H	-7.96425	-2.18916	-0.21024
C	-8.39696	-4.01634	-1.18318
O	-7.4323	-4.5981	-0.65314
N	-9.11757	-4.59897	-2.16568
H	-9.67995	-4.05353	-2.82018
H	-8.77964	-5.49533	-2.49376
C	-9.81254	-0.36054	-1.05156
O	-10.3395	-0.4035	0.057474
N	-9.57551	0.776901	-1.74879
H	-9.17825	0.739269	-2.6853
C	-9.97452	2.085577	-1.24201
H	-10.643	1.905963	-0.39755
C	-8.79169	2.984636	-0.83369
H	-8.12072	3.088001	-1.6955
H	-9.2047	3.987125	-0.64779
C	-7.99623	2.533432	0.378328
C	-6.6051	2.398121	0.311789
H	-6.09287	2.583298	-0.6292
C	-5.84218	2.039809	1.426052
H	-4.76872	1.900537	1.320317
C	-6.47984	1.82488	2.649397

O	-5.78718	1.457019	3.788907
H	-4.85027	1.691431	3.674665
C	-7.86892	1.944588	2.743686
H	-8.35242	1.777971	3.701778
C	-8.61163	2.2914	1.61705
H	-9.69051	2.378698	1.711804
C	2.818527	6.285916	-3.67689
H	3.174168	6.354548	-2.64161
H	1.802391	6.687643	-3.72038
C	2.845615	4.815522	-4.0746
O	3.896983	4.399404	-4.67308
O	1.871467	4.079415	-3.78011
C	8.042188	-5.02102	-5.77623
C	7.220552	-4.26665	-4.78363
C	6.031538	-3.59264	-4.88947
N	7.584349	-4.1147	-3.45409
C	6.637895	-3.37592	-2.82355
N	5.681501	-3.03927	-3.6708
H	8.182642	-6.06875	-5.48112
H	7.545082	-5.01598	-6.75013
H	5.398978	-3.48659	-5.75901
H	6.664145	-3.178	-1.76244
H	8.400146	-4.50923	-3.00762
C	4.293082	-7.72885	1.317123
H	4.909928	-8.28089	0.593267
C	3.573154	-6.58705	0.580597
H	4.298192	-5.88144	0.158342
H	3.054133	-7.03222	-0.28049
C	2.54839	-5.83071	1.437243
H	1.867475	-6.54485	1.920494
H	3.056894	-5.28631	2.24327
C	1.734399	-4.84751	0.58218
H	2.388778	-4.11162	0.109645
H	1.230969	-5.37559	-0.23393
N	0.691211	-4.14328	1.32695
H	-0.18552	-4.63022	1.537655
C	0.818989	-2.95987	1.932494
N	1.954769	-2.27842	2.018761
H	2.794786	-2.53207	1.48996
H	1.916148	-1.36515	2.494906
N	-0.32233	-2.39726	2.441018
H	-1.10726	-3.04292	2.48063
H	-0.23758	-1.84175	3.297631
C	5.210162	-7.38082	2.491127
O	5.179169	-8.0319	3.532603
N	6.112242	-6.36858	2.286368
H	6.145494	-5.85401	1.404693

C	7.180177	-6.16339	3.24111
H	6.90814	-6.72735	4.136952
C	7.470089	-4.69582	3.558456
O	7.446507	-3.80027	2.705505
N	7.857585	-4.4575	4.833652
H	7.800943	-5.21434	5.499659
C	8.347767	-3.15264	5.28806
H	9.058748	-2.78537	4.541056
C	7.242344	-2.10558	5.489674
H	7.714095	-1.15039	5.749267
H	6.743769	-1.95807	4.527527
C	6.221753	-2.47861	6.568878
H	5.826346	-3.48849	6.40681
H	6.685469	-2.46612	7.562432
S	4.80796	-1.31155	6.673545
C	3.897434	-1.78469	5.16126
H	3.131316	-1.02858	4.974858
H	4.561899	-1.80221	4.292454
H	3.422841	-2.76435	5.276662
O	1.986793	-1.37325	-2.754
O	6.391496	-4.29274	0.197789
Fe	3.749457	-2.0294	-3.17277
C	-0.54584	-1.99657	-4.81082
H	0.112565	-2.70337	-5.32314
H	-0.9356	-2.52497	-3.9332
C	-1.78653	-1.7422	-5.73638
H	-1.66684	-2.34951	-6.64009
C	-3.19774	-2.12291	-5.15471
O	-3.21858	-2.94735	-4.21568
O	-4.15035	-1.55686	-5.75497
N	-1.97131	-0.33126	-6.25317
H	-2.20173	0.405345	-5.51324
H	-2.85429	-0.3856	-6.78518
H	-1.20079	0.000182	-6.83769
C	0.245043	-0.79361	-4.40442
H	1.120804	-0.52806	-4.98739
H	1.460892	-2.20073	-2.57964
C	-0.33254	0.218311	-3.47849
H	-0.02632	-0.03009	-2.45613
H	-1.42445	0.16843	-3.46835
C	0.120487	1.641839	-3.81176
H	1.152908	1.687205	-4.14897
H	-0.52114	2.083717	-4.5743
N	0.02969	2.524932	-2.6133
H	8.127557	-6.58698	2.867056
H	8.89889	-3.326	6.218856
H	-3.75811	-7.11909	0.515673

H	3.564869	-8.4407	1.717374
H	-7.5176	-3.11961	4.783618
H	-4.52744	-0.36594	6.692627
H	9.037112	-4.57669	-5.90831
H	8.098142	1.605481	-5.49831
H	3.48277	6.87934	-4.3106
H	6.192016	5.670077	-2.56825
H	0.419359	5.94874	6.032767
H	-5.90736	2.501521	-4.84373
H	-7.66844	-2.35125	-5.85055
H	-10.5493	2.595054	-2.02457
H	6.863242	-3.99587	1.0092
H	5.528923	-3.82767	0.243291
C	1.928694	-4.39612	-3.34078
O	3.02597	-3.80451	-3.70187
O	1.029793	-3.88408	-2.64919
C	1.799104	-5.83379	-3.82075
H	0.802398	-6.22418	-3.60614
H	2.550044	-6.45163	-3.31452
H	2.005595	-5.89432	-4.89421
H	0.539058	3.407671	-2.8397
H	-0.98228	2.659323	-2.40352
H	0.520493	2.052888	-1.82453
O	1.516424	0.768953	-0.86047
H	1.789601	0.005982	-1.42313
H	0.819889	0.402936	-0.25288
O	-3.10121	0.442165	-0.35053
H	-3.05574	1.145838	-1.0383
H	-2.19609	0.375339	0.045884
O	-0.6064	-0.01119	0.697322
H	-0.62348	-0.89938	1.113608
H	-0.36698	0.597161	1.470851

[5]**Pr**$_{B,C4}$

C	-2.20521	-3.888	6.458298
H	-2.01586	-2.8989	6.025617
C	-0.96322	-4.77536	6.284009
H	-0.93112	-5.53065	7.077357
H	-1.05281	-5.32247	5.343061
C	0.350035	-3.98549	6.291903
H	0.442145	-3.37806	7.2026
H	1.20195	-4.67891	6.307508
C	0.511641	-3.06338	5.077635
O	-0.346	-3.03657	4.174853
N	1.609613	-2.29501	5.059982
H	2.344366	-2.40041	5.750742
H	1.772634	-1.62286	4.299961
C	-3.44888	-4.43375	5.756114
O	-3.44954	-5.48502	5.1076
N	-4.56093	-3.65913	5.874773
H	-4.47427	-2.77834	6.362988
C	-5.77389	-3.91226	5.097971
H	-6.61105	-3.43427	5.61824
C	-5.66888	-3.417	3.645701
H	-4.7539	-3.83901	3.213302
H	-6.5042	-3.84003	3.074054
C	-5.65854	-1.88352	3.505261
H	-5.09318	-1.42594	4.327163
H	-6.68241	-1.49395	3.586443
C	-5.00906	-1.37658	2.211893
H	-5.10403	-0.28709	2.144816
H	-3.93901	-1.61676	2.220541
N	-5.60207	-2.02262	1.034245
H	-6.50349	-2.49818	1.12565
C	-4.94093	-2.27385	-0.1021
N	-3.76149	-1.68385	-0.37061
H	-3.57902	-0.71356	-0.05933
H	-3.3522	-1.95325	-1.26356
N	-5.45281	-3.1447	-0.98579
H	-6.21233	-3.74707	-0.69191
H	-4.96173	-3.27234	-1.87539
C	-2.87208	-6.19499	-1.30817
H	-1.98613	-6.71557	-1.6941
H	-2.94856	-5.23813	-1.83808
C	-2.78107	-5.99786	0.173447
N	-1.79056	-5.21705	0.748107
C	-1.99645	-5.27453	2.058932
H	-1.41913	-4.76695	2.821505
N	-3.06429	-6.04755	2.357892

H	-3.42777	-6.1511	3.304906
C	-3.5793	-6.51524	1.166676
H	-4.44003	-7.1657	1.128958
C	6.611946	4.027295	-5.48823
C	6.664514	4.213617	-3.96726
C	5.243943	3.61624	-6.09781
O	7.462527	3.556509	-3.27993
C	4.615737	2.469745	-5.3649
C	4.817444	1.107128	-5.36263
N	3.753364	2.721397	-4.32037
C	3.481475	1.555827	-3.72014
N	4.102454	0.541467	-4.32238
H	6.943738	4.959004	-5.96459
H	4.55065	4.464318	-6.08171
H	5.407975	3.360328	-7.15033
H	5.438021	0.50484	-6.01071
H	2.871129	1.468213	-2.83745
H	3.352556	3.697692	-4.10903
N	5.86826	5.170729	-3.42733
H	5.000671	5.457897	-3.88822
C	5.958194	5.516693	-2.01197
H	7.010984	5.466873	-1.71966
C	5.114901	4.628249	-1.08083
O	4.086853	5.055934	-0.54939
N	5.600892	3.377574	-0.89286
C	4.869071	2.329848	-0.17856
C	4.378881	2.832637	1.199063
C	5.803344	1.129532	0.047684
O	5.168731	3.164421	2.077407
C	5.11377	-0.13727	0.593737
C	4.473872	-1.04841	-0.4487
O	4.504872	-2.29032	-0.28708
O	3.892556	-0.49028	-1.46185
H	6.412872	3.117766	-1.44875
H	4.010992	2.022538	-0.78209
H	6.314861	0.887923	-0.89047
H	6.563128	1.45281	0.767187
H	4.334477	0.119077	1.326471
H	5.838379	-0.74927	1.138657
N	3.028724	2.822692	1.353894
H	2.454003	2.478086	0.587156
C	2.372175	3.130429	2.620135
H	1.299723	3.185221	2.398454
C	2.539511	1.999045	3.651494
H	3.595082	1.748256	3.772825
H	2.152008	2.357438	4.610017
C	1.719224	0.789042	3.196624

O	0.498044	0.994389	2.972645
O	2.317144	-0.33293	3.051018
C	2.728643	4.512039	3.196249
O	2.546957	4.749401	4.394005
N	3.103284	5.474749	2.313089
H	3.353954	5.20995	1.361583
C	3.562821	6.771669	2.810294
H	4.163523	6.597377	3.709779
C	4.420472	7.486993	1.762416
H	5.321556	6.905103	1.546451
H	4.708754	8.466477	2.151691
H	3.869685	7.625169	0.825724
C	2.427625	7.696569	3.29576
O	2.694139	8.713206	3.927574
N	1.161697	7.303604	2.982246
H	1.050716	6.474045	2.413564
C	-0.027	7.975295	3.470846
H	0.319802	8.923506	3.895689
C	-1.02493	8.285523	2.327383
H	-0.56502	9.04009	1.679421
H	-1.90829	8.730501	2.793392
C	-1.41175	7.071788	1.505702
C	-0.73342	6.763733	0.316439
H	0.049808	7.431288	-0.03725
C	-1.05174	5.622729	-0.4262
H	-0.51048	5.409022	-1.3456
C	-2.06139	4.767508	0.020974
H	-2.31447	3.876755	-0.54855
C	-2.75113	5.062187	1.200731
H	-3.53767	4.399758	1.553125
C	-2.43136	6.206309	1.934065
H	-2.96684	6.434262	2.851276
C	-0.73188	7.229663	4.63333
O	-1.84923	7.60063	5.001092
N	-0.03887	6.219136	5.208757
H	0.88051	5.970078	4.854305
C	-0.52376	5.490956	6.375146
H	-1.35258	6.068879	6.78887
C	-0.94462	4.043018	6.070831
H	-0.0816	3.52079	5.635158
H	-1.15287	3.547945	7.032632
C	-2.16095	3.868603	5.140952
H	-1.93319	4.379357	4.195205
C	-2.37648	2.38122	4.818204
H	-3.21731	2.252822	4.124493
H	-1.48493	1.937113	4.360031
H	-2.60854	1.812713	5.731093

C	-3.4374	4.498494	5.720038
H	-4.29497	4.302464	5.064521
H	-3.67232	4.073413	6.706664
H	-3.33933	5.583453	5.823493
C	-5.75156	1.104977	-4.48203
H	-5.20735	0.733583	-5.35584
C	-4.87092	0.898288	-3.23546
H	-5.30381	1.397764	-2.3665
H	-4.81262	-0.17204	-2.99707
C	-3.42521	1.376399	-3.43849
O	-2.88324	1.169475	-4.56676
O	-2.8339	1.9108	-2.4522
C	-7.10342	0.410698	-4.39575
O	-8.16876	1.027824	-4.28406
N	-7.06456	-0.95558	-4.43989
H	-6.1612	-1.42715	-4.54704
C	-8.26268	-1.74161	-4.66973
H	-8.904	-1.23583	-5.40309
C	-9.1628	-2.02227	-3.46474
O	-10.0465	-2.88162	-3.53759
N	-8.96131	-1.25826	-2.36238
H	-8.19287	-0.6011	-2.37491
C	-9.90505	-1.21741	-1.25623
H	-10.7784	-1.78852	-1.58824
C	-9.32699	-1.84292	0.022029
H	-10.0931	-1.78975	0.803658
H	-8.48149	-1.2457	0.378094
C	-8.84802	-3.28134	-0.09889
O	-7.90488	-3.69592	0.60226
N	-9.50647	-4.10702	-0.94029
H	-10.0279	-3.75744	-1.74597
H	-9.14139	-5.04876	-1.01239
C	-10.4303	0.203132	-0.93969
O	-11.0144	0.387337	0.126464
N	-10.235	1.15164	-1.88598
H	-9.7141	0.937326	-2.73269
C	-10.6197	2.541388	-1.66142
H	-11.1425	2.563211	-0.7038
C	-9.42232	3.508223	-1.67897
H	-9.02686	3.556432	-2.70125
H	-9.81741	4.511055	-1.45686
C	-8.28168	3.158497	-0.73981
C	-6.9599	3.331976	-1.16742
H	-6.77175	3.729806	-2.16257
C	-5.87216	3.008932	-0.35589
H	-4.86024	3.164697	-0.71773
C	-6.08933	2.474115	0.920775

O	-5.06482	2.090109	1.738599
H	-4.26956	1.869746	1.191212
C	-7.40472	2.325107	1.378852
H	-7.56964	1.927471	2.376208
C	-8.48081	2.666882	0.560057
H	-9.48795	2.506368	0.934182
C	1.883189	7.00968	-2.9844
H	2.376591	7.062443	-2.00569
H	0.845653	7.332689	-2.87459
C	1.963818	5.566682	-3.46609
O	3.073994	5.210483	-3.99523
O	0.971454	4.819043	-3.29059
C	9.712178	0.633157	-2.9758
C	8.512899	-0.25531	-2.9346
C	7.20209	-0.01643	-3.25234
N	8.532817	-1.58065	-2.52258
C	7.273984	-2.08337	-2.59723
N	6.439986	-1.15481	-3.04059
H	10.44683	0.301522	-3.7212
H	9.393442	1.647263	-3.2334
H	6.770145	0.906359	-3.60605
H	7.015429	-3.08326	-2.27208
H	9.34319	-2.0916	-2.20158
C	3.961739	-7.51335	-1.38852
H	4.736594	-7.74085	-2.13463
C	3.462716	-6.07713	-1.61481
H	4.297159	-5.36996	-1.54839
H	3.090128	-6.01156	-2.64612
C	2.345739	-5.64562	-0.65498
H	1.54059	-6.39355	-0.65382
H	2.728398	-5.5957	0.372725
C	1.772633	-4.28053	-1.05717
H	2.560026	-3.52332	-1.07288
H	1.372995	-4.32317	-2.07534
N	0.693	-3.8305	-0.17635
H	-0.16939	-4.39161	-0.12785
C	0.794292	-2.91553	0.789272
N	1.926559	-2.32591	1.149341
H	2.815044	-2.56807	0.715429
H	1.93108	-1.54861	1.842206
N	-0.36639	-2.51466	1.411712
H	-1.17052	-3.08269	1.15018
H	-0.28832	-2.41713	2.437224
C	4.544412	-7.86017	-0.01862
O	4.255351	-8.90994	0.552166
N	5.439962	-6.9761	0.521625
H	5.750196	-6.14932	0.006924

C	6.153117	-7.38276	1.713552
H	5.446081	-7.889	2.378133
C	6.86303	-6.23601	2.426761
O	7.354533	-5.26286	1.8464
N	6.991267	-6.40869	3.766378
H	6.55817	-7.21904	4.185194
C	7.817013	-5.54286	4.6131
H	8.796711	-5.42869	4.136252
C	7.222126	-4.1483	4.866892
H	7.921716	-3.58906	5.500187
H	7.168929	-3.6274	3.907125
C	5.841962	-4.17581	5.526985
H	5.139557	-4.76361	4.925053
H	5.892449	-4.6366	6.521055
S	5.1201	-2.50975	5.812539
C	5.056916	-1.88011	4.094096
H	4.3395	-1.05718	4.053319
H	6.036983	-1.52424	3.767021
H	4.703123	-2.66279	3.415299
O	1.45485	-1.74432	-3.54826
O	6.408929	-4.39321	-0.6239
Fe	4.259848	-1.29636	-3.29054
C	-0.79824	-2.49066	-4.0608
H	-0.26949	-3.39536	-4.37603
H	-1.09669	-2.64798	-3.01988
C	-2.08846	-2.39396	-4.91146
H	-2.08139	-3.20156	-5.6515
C	-3.46597	-2.52727	-4.16771
O	-3.46106	-2.98725	-3.00116
O	-4.44296	-2.16019	-4.87193
N	-2.26924	-1.13909	-5.74381
H	-2.42373	-0.23898	-5.17837
H	-3.18691	-1.28419	-6.19328
H	-1.53352	-0.99633	-6.43845
C	0.219849	-1.3256	-4.11453
H	0.384884	-1.03532	-5.1707
H	1.757241	-2.57771	-4.0093
C	-0.25913	-0.11202	-3.30562
H	0.185441	-0.2164	-2.31197
H	-1.34174	-0.1313	-3.16998
C	0.1305	1.238711	-3.906
H	1.170954	1.245993	-4.22734
H	-0.51343	1.493116	-4.7491
N	-0.05386	2.319001	-2.88894
H	6.927393	-8.13276	1.475925
H	7.966378	-6.07741	5.557214
H	-3.74792	-6.79936	-1.56581

H	3.151316	-8.23125	-1.54819
H	-5.94633	-4.99143	5.110413
H	-2.43356	-3.72773	7.520665
H	10.22081	0.677028	-2.00432
H	7.355771	3.258006	-5.71061
H	2.418066	7.678369	-3.66546
H	5.59652	6.53831	-1.8862
H	0.279878	5.477585	7.12223
H	-5.94917	2.169407	-4.63248
H	-7.97601	-2.70771	-5.09144
H	-11.337	2.847512	-2.43347
H	6.917921	-4.53254	0.202573
H	5.748237	-3.70403	-0.40166
C	3.590485	-3.85459	-4.86264
O	4.323017	-2.92295	-4.34843
O	2.351256	-3.9321	-4.75736
C	4.355406	-4.91247	-5.63961
H	3.672392	-5.64039	-6.08114
H	5.056158	-5.42242	-4.96926
H	4.951394	-4.43497	-6.42462
H	0.334526	3.252577	-3.19261
H	-1.07881	2.381053	-2.68098
H	0.458283	2.051559	-2.0257
O	1.530758	1.210224	-0.72655
H	2.190679	0.514664	-0.92368
H	0.809469	0.786513	-0.18249
O	-3.11877	1.023789	0.139268
H	-3.08043	1.391166	-0.7805
H	-2.17342	0.906101	0.422739
O	-0.56073	0.295387	0.725309
H	-0.62601	-0.68186	0.812528
H	-0.22419	0.570632	1.642077

^{5}**TS1**$_{B,C5}$

C	-3.98748	0.012334	5.986943
H	-3.33692	0.128002	5.111526
C	-3.17652	-0.56502	7.152138
H	-3.73598	-0.44691	8.087255
H	-3.04881	-1.64049	7.007158
C	-1.79878	0.090473	7.307822
H	-1.89062	1.182618	7.388167
H	-1.33525	-0.2434	8.246326
C	-0.82222	-0.23437	6.171363
O	-1.0973	-1.0677	5.294292
N	0.360853	0.406723	6.203328
H	0.522635	1.148405	6.869567
H	0.995567	0.336312	5.396863
C	-5.16715	-0.86196	5.555114
O	-5.38794	-1.98232	6.036097
N	-5.93421	-0.33001	4.572785
H	-5.65736	0.554812	4.156754
C	-6.95777	-1.08887	3.853094
H	-7.46248	-0.37429	3.196813
C	-6.40505	-2.27261	3.031512
H	-6.17278	-3.09361	3.71819
H	-7.21228	-2.62646	2.375287
C	-5.14582	-1.93587	2.222139
H	-4.40664	-1.49138	2.897804
H	-5.34979	-1.17988	1.455845
C	-4.47585	-3.15532	1.573937
H	-3.41881	-2.94873	1.396104
H	-4.52114	-4.01564	2.251514
N	-5.05581	-3.57094	0.292032
H	-5.86593	-4.19567	0.29321
C	-4.39783	-3.47594	-0.87647
N	-3.34791	-2.66908	-1.03136
H	-3.2756	-1.75154	-0.54727
H	-2.7694	-2.82981	-1.86516
N	-4.80108	-4.23469	-1.91627
H	-5.47303	-4.9745	-1.75805
H	-4.46469	-4.00854	-2.85449
C	-2.46263	-6.94437	3.113917
H	-1.50435	-7.33719	3.475861
H	-2.34194	-6.72157	2.045848
C	-2.87686	-5.72833	3.88331
N	-2.10223	-4.5813	3.913135
C	-2.75178	-3.72655	4.69046
H	-2.41762	-2.72829	4.943133
N	-3.90246	-4.25852	5.164061

H	-4.58724	-3.71655	5.692136
C	-4.00259	-5.53633	4.653017
H	-4.83264	-6.1868	4.884695
C	7.521548	3.031474	-5.2796
C	7.209631	3.668462	-3.92312
C	6.335441	2.390221	-6.04463
O	7.878725	3.363999	-2.92771
C	5.484614	1.487038	-5.20082
C	5.593372	0.178279	-4.78493
N	4.357729	1.984014	-4.58152
C	3.834625	1.011986	-3.83167
N	4.546408	-0.11243	-3.92945
H	7.987228	3.789455	-5.92388
H	5.693603	3.170932	-6.47014
H	6.748089	1.836282	-6.89563
H	6.342868	-0.55858	-5.03038
H	2.970447	1.143148	-3.20812
H	3.951705	2.97382	-4.70616
N	6.22962	4.607924	-3.87057
H	5.500163	4.67278	-4.57961
C	5.907534	5.286484	-2.61988
H	6.845667	5.516455	-2.10541
C	4.989495	4.491773	-1.67321
O	3.861954	4.9053	-1.38082
N	5.52213	3.34812	-1.19175
C	4.806153	2.405416	-0.33236
C	4.153749	3.151881	0.853331
C	5.82835	1.389721	0.20455
O	4.831589	3.776499	1.663634
C	5.238096	0.221955	1.001941
C	4.657269	-0.94896	0.208146
O	4.334891	-1.98742	0.828424
O	4.580102	-0.80807	-1.06701
H	6.468187	3.120147	-1.49454
H	4.035464	1.890981	-0.91785
H	6.407147	0.993663	-0.6374
H	6.515122	1.945107	0.852642
H	4.457526	0.557197	1.698219
H	6.021704	-0.21701	1.629441
N	2.798443	3.054026	0.923756
H	2.31415	2.437017	0.276499
C	2.024451	3.630103	2.01999
H	0.973625	3.517245	1.730904
C	2.188034	2.835063	3.323574
H	3.24333	2.671595	3.551447
H	1.759084	3.423975	4.142108
C	1.437277	1.50225	3.230937

O	0.337688	1.516398	2.619077
O	1.956745	0.484081	3.800867
C	2.225054	5.141429	2.225515
O	1.900099	5.664589	3.298254
N	2.632619	5.879674	1.159686
H	2.993292	5.404999	0.331826
C	3.009239	7.280247	1.358833
H	3.482657	7.362445	2.343718
C	4.000659	7.74617	0.288696
H	4.926822	7.167775	0.355123
H	4.222532	8.803549	0.451786
H	3.588748	7.619017	-0.71769
C	1.816236	8.252076	1.445407
O	1.996694	9.417688	1.782183
N	0.591406	7.7299	1.151124
H	0.550312	6.764128	0.851669
C	-0.64424	8.4578	1.365155
H	-0.34085	9.492422	1.561619
C	-1.55811	8.452637	0.117204
H	-1.05059	9.028496	-0.66476
H	-2.47001	8.988663	0.394046
C	-1.90016	7.072953	-0.40871
C	-1.13214	6.482923	-1.4233
H	-0.29361	7.032023	-1.84584
C	-1.43426	5.207831	-1.90877
H	-0.81654	4.780084	-2.69525
C	-2.51752	4.498264	-1.38596
H	-2.76149	3.507614	-1.76139
C	-3.2896	5.074603	-0.37283
H	-4.1356	4.528861	0.038133
C	-2.98452	6.347767	0.112049
H	-3.58307	6.793455	0.90148
C	-1.41474	8.006162	2.631395
O	-2.58553	8.359843	2.797181
N	-0.70907	7.277132	3.527661
H	0.242215	6.988596	3.313119
C	-1.24957	6.875552	4.818556
H	-2.17944	7.430329	4.96009
C	-1.46994	5.359498	4.939008
H	-0.50621	4.861738	4.766927
H	-1.75372	5.142531	5.981273
C	-2.52267	4.751096	3.993687
H	-2.2337	4.997754	2.962603
C	-2.52454	3.220335	4.122812
H	-3.28464	2.772158	3.472459
H	-1.55569	2.788256	3.848222
H	-2.75248	2.921165	5.157206

C	-3.92709	5.328773	4.230957
H	-4.65374	4.842149	3.566428
H	-4.25382	5.162776	5.268206
H	-3.96478	6.402868	4.024117
C	-5.48867	0.293339	-4.75314
H	-4.88217	0.115557	-5.64867
C	-4.60694	0.057162	-3.51382
H	-5.04815	0.517742	-2.62742
H	-4.5082	-1.01588	-3.30984
C	-3.17638	0.587484	-3.67816
O	-2.6109	0.446495	-4.80935
O	-2.63593	1.104431	-2.66366
C	-6.72526	-0.5883	-4.81164
O	-7.8742	-0.14036	-4.79947
N	-6.48605	-1.93693	-4.88464
H	-5.53036	-2.29075	-4.83977
C	-7.55577	-2.86819	-5.19087
H	-8.1725	-2.47858	-6.00856
C	-8.53512	-3.1964	-4.0607
O	-9.46697	-3.97794	-4.26134
N	-8.31576	-2.58317	-2.87097
H	-7.48409	-2.01494	-2.77796
C	-9.25273	-2.66557	-1.76149
H	-10.1444	-3.15403	-2.16938
C	-8.6912	-3.49177	-0.59502
H	-9.46421	-3.56901	0.177279
H	-7.84666	-2.96574	-0.14065
C	-8.20554	-4.88898	-0.95907
O	-7.20513	-5.37774	-0.40839
N	-8.93182	-5.60128	-1.85262
H	-9.50088	-5.14322	-2.56279
H	-8.55482	-6.5085	-2.09864
C	-9.74046	-1.28608	-1.25398
O	-10.2868	-1.21468	-0.15799
N	-9.56102	-0.24167	-2.10205
H	-9.15106	-0.40081	-3.01867
C	-10.1159	1.082488	-1.83931
H	-10.6595	1.015625	-0.89454
C	-9.05082	2.199113	-1.78615
H	-8.41065	2.108386	-2.6729
H	-9.58171	3.15477	-1.88516
C	-8.20871	2.224634	-0.52781
C	-6.96274	1.587495	-0.46325
H	-6.59205	1.06572	-1.34089
C	-6.16935	1.621757	0.683978
H	-5.18624	1.1569	0.698501
C	-6.64239	2.299105	1.810401

O	-5.85004	2.294483	2.944521
H	-6.13405	3.007818	3.538732
C	-7.88284	2.941005	1.779621
H	-8.24658	3.471698	2.657443
C	-8.64968	2.904459	0.615241
H	-9.60675	3.421188	0.59907
C	1.924818	6.046227	-4.1241
H	2.190471	6.322097	-3.09654
H	0.839283	6.125411	-4.2318
C	2.393212	4.613961	-4.3492
O	3.542849	4.461146	-4.88892
O	1.660339	3.663169	-3.97654
C	8.816509	-4.17662	-4.73902
C	7.797128	-3.52878	-3.86079
C	6.50808	-3.1225	-4.09007
N	8.031828	-3.20299	-2.53328
C	6.919831	-2.62445	-2.01759
N	5.975284	-2.55949	-2.94243
H	9.136621	-5.15103	-4.34815
H	8.39621	-4.34085	-5.73493
H	5.918051	-3.20807	-4.98981
H	6.858327	-2.32198	-0.98022
H	8.884912	-3.36477	-2.01643
C	4.467265	-7.68759	0.963625
H	5.198854	-8.02947	0.220637
C	3.901769	-6.31508	0.533656
H	4.710707	-5.57238	0.510647
H	3.542593	-6.39579	-0.49985
C	2.766073	-5.80553	1.430968
H	1.971205	-6.5643	1.480131
H	3.120981	-5.67301	2.459098
C	2.188587	-4.48334	0.912075
H	2.965967	-3.72204	0.841156
H	1.805656	-4.60366	-0.10802
N	1.100235	-3.99558	1.762353
H	0.271858	-4.5746	1.81669
C	1.025974	-2.83233	2.437228
N	2.023395	-1.96519	2.511793
H	2.880696	-2.06446	1.963854
H	1.889436	-1.06106	3.010633
N	-0.16753	-2.53869	3.017039
H	-0.7866	-3.33909	3.215043
H	-0.19031	-1.83342	3.762183
C	5.105611	-7.6341	2.345853
O	4.478099	-7.84355	3.378414
N	6.439497	-7.30253	2.349084
H	6.851908	-6.9222	1.505082

C	7.104828	-6.90421	3.567563
H	6.356753	-6.88408	4.366969
C	7.777495	-5.54067	3.369974
O	7.981278	-5.10316	2.231008
N	8.162654	-4.9088	4.498866
H	7.895727	-5.31856	5.383735
C	8.886127	-3.63253	4.514286
H	9.491384	-3.60024	3.604828
C	7.979563	-2.39269	4.57633
H	8.622853	-1.50517	4.536246
H	7.36476	-2.36603	3.670921
C	7.114405	-2.32552	5.839104
H	6.482287	-3.2174	5.936603
H	7.748132	-2.27853	6.733574
S	6.034771	-0.84454	5.937967
C	4.714953	-1.31391	4.764376
H	4.012432	-0.47778	4.703018
H	5.109354	-1.50128	3.761743
H	4.176937	-2.19955	5.118324
O	2.322307	-1.16866	-2.62365
O	7.111559	-2.60092	1.167321
Fe	3.956765	-1.7359	-2.68903
C	-0.13649	-2.69419	-5.55133
H	0.314619	-3.1417	-6.44596
H	0.158497	-3.31938	-4.70364
C	-1.66389	-2.8519	-5.67483
H	-1.86776	-3.71207	-6.32345
C	-2.47945	-3.11135	-4.37132
O	-1.84468	-3.33768	-3.32841
O	-3.7433	-3.08975	-4.53961
N	-2.37275	-1.68866	-6.33722
H	-2.43532	-0.80701	-5.69936
H	-3.34561	-1.99093	-6.45408
H	-1.97247	-1.43439	-7.24337
C	0.470179	-1.28952	-5.39282
H	0.090981	-0.6319	-6.19262
H	1.544307	-1.38695	-5.59105
C	0.291042	-0.58532	-4.04333
H	1.207014	-0.92993	-3.34801
H	-0.60957	-0.8954	-3.50652
C	0.360673	0.920219	-4.2294
H	1.3286	1.247737	-4.6076
H	-0.40746	1.225972	-4.94372
N	0.083098	1.69962	-2.98477
H	7.877689	-7.6313	3.859091
H	9.56876	-3.65895	5.371914
H	-3.20826	-7.74028	3.206468

H	3.66206	-8.42776	1.007214
H	-7.69633	-1.46093	4.570952
H	-4.35336	1.01928	6.228302
H	9.713275	-3.55447	-4.85476
H	8.27757	2.271523	-5.06733
H	2.421991	6.738087	-4.809
H	5.388241	6.214778	-2.85883
H	-0.54794	7.196138	5.599527
H	-5.83043	1.33054	-4.79244
H	-7.11583	-3.81062	-5.52839
H	-10.8369	1.318148	-2.63287
H	7.452765	-3.45648	1.493282
H	6.139823	-2.65333	1.245865
C	3.242808	-4.03706	-3.40994
O	3.417706	-3.73985	-2.18438
O	3.472801	-3.1705	-4.30699
C	2.747205	-5.41797	-3.77797
H	2.768583	-5.56232	-4.85992
H	1.720563	-5.54125	-3.41458
H	3.363509	-6.17844	-3.28804
H	0.429204	2.667502	-3.15304
H	-0.94633	1.655438	-2.80228
H	0.576173	1.322185	-2.13706
O	1.386743	0.670164	-0.71968
H	1.844745	-0.07383	-1.1704
H	0.711704	0.258298	-0.12286
O	-3.16676	-0.06263	-0.08609
H	-3.03954	0.427098	-0.92896
H	-2.29203	-0.00222	0.374779
O	-0.63961	-0.24869	0.989905
H	-0.63539	-1.11036	1.448647
H	-0.34567	0.402773	1.703825

^{5}**IM1**$_{B,C5}$

C	-3.36057	-0.75159	6.189462
H	-2.83925	-0.42682	5.280488
C	-2.34397	-1.23841	7.22779
H	-2.80584	-1.26341	8.221027
H	-2.05485	-2.2664	6.998949
C	-1.08374	-0.36459	7.275049
H	-1.33355	0.678671	7.509665
H	-0.42619	-0.71313	8.083461
C	-0.27779	-0.41749	5.973182
O	-0.379	-1.38603	5.202246
N	0.54566	0.614557	5.727572
H	0.591535	1.401483	6.358126
H	1.066181	0.658148	4.838374
C	-4.38089	-1.81244	5.764218
O	-4.40985	-2.95389	6.244966
N	-5.23813	-1.39831	4.802689
H	-5.09835	-0.47344	4.402375
C	-6.21475	-2.27322	4.156715
H	-6.88876	-1.62179	3.591629
C	-5.59799	-3.33525	3.22437
H	-5.15507	-4.12976	3.834688
H	-6.42233	-3.79631	2.661911
C	-4.5265	-2.7875	2.272937
H	-3.70314	-2.3764	2.868444
H	-4.91127	-1.95953	1.666793
C	-3.92945	-3.85153	1.343036
H	-2.98825	-3.49618	0.922957
H	-3.70449	-4.76322	1.912057
N	-4.80431	-4.21307	0.224049
H	-5.65411	-4.7474	0.412889
C	-4.50113	-4.0429	-1.07394
N	-3.48315	-3.27822	-1.47249
H	-3.21848	-2.42674	-0.93587
H	-3.2343	-3.33517	-2.46651
N	-5.23015	-4.69822	-2.00298
H	-5.96366	-5.31986	-1.68476
H	-5.26137	-4.30146	-2.94535
C	-1.21048	-7.33998	2.750432
H	-0.23515	-7.73602	3.060486
H	-1.10798	-6.99312	1.714466
C	-1.66244	-6.23404	3.653178
N	-0.9485	-5.05434	3.78039
C	-1.61649	-4.3209	4.660483
H	-1.33893	-3.32791	4.992668
N	-2.71919	-4.96366	5.11037

H	-3.42341	-4.5204	5.702656
C	-2.76835	-6.18598	4.471757
H	-3.55226	-6.90384	4.661575
C	6.261476	2.793011	-6.24198
C	6.151448	3.61129	-4.95528
C	5.062824	1.88047	-6.59334
O	7.015389	3.52278	-4.07707
C	4.703587	0.8849	-5.52805
C	5.277299	-0.29065	-5.10327
N	3.597709	1.069957	-4.72221
C	3.526392	0.038456	-3.86394
N	4.535104	-0.81333	-4.06273
H	6.425167	3.486569	-7.07747
H	4.176277	2.484224	-6.81641
H	5.314837	1.351232	-7.52025
H	6.167067	-0.78333	-5.46346
H	2.763157	-0.06729	-3.1077
H	2.982831	1.936383	-4.77152
N	5.113169	4.492927	-4.86285
H	4.228496	4.287315	-5.33105
C	5.009948	5.380154	-3.70813
H	6.025762	5.644418	-3.40095
C	4.249825	4.760901	-2.52767
O	3.217555	5.27897	-2.08803
N	4.777727	3.611505	-2.05041
C	4.016401	2.680719	-1.21942
C	3.499446	3.366368	0.06185
C	4.919991	1.493677	-0.85568
O	4.263404	3.775388	0.929651
C	4.183087	0.3809	-0.08682
C	4.985606	-0.91289	-0.03513
O	5.226313	-1.47743	1.051112
O	5.411182	-1.38154	-1.16587
H	5.653535	3.297906	-2.46336
H	3.158544	2.322456	-1.8046
H	5.334633	1.075874	-1.77907
H	5.751655	1.874537	-0.2518
H	3.224789	0.151001	-0.57423
H	3.965966	0.6948	0.937278
N	2.143599	3.420165	0.152401
H	1.59559	3.117643	-0.64192
C	1.423396	3.893756	1.328503
H	0.363131	3.762341	1.086306
C	1.708768	3.035884	2.566662
H	2.775214	3.046342	2.802044
H	1.188713	3.478702	3.423535
C	1.235688	1.590207	2.377571

O	0.380467	1.367332	1.488739
O	1.751551	0.715099	3.157404
C	1.603729	5.395231	1.611083
O	1.256344	5.870921	2.698762
N	2.033974	6.168335	0.581623
H	2.388596	5.725683	-0.26566
C	2.281162	7.596089	0.773423
H	2.731547	7.728275	1.763986
C	3.240561	8.132905	-0.29244
H	4.216275	7.645166	-0.20607
H	3.36077	9.209298	-0.14965
H	2.855875	7.947733	-1.30086
C	0.999095	8.456762	0.833501
O	1.070138	9.649276	1.110171
N	-0.17014	7.799639	0.595121
H	-0.11929	6.822591	0.339453
C	-1.47942	8.392979	0.782208
H	-1.32294	9.477102	0.798322
C	-2.43366	8.046971	-0.38714
H	-2.04186	8.529116	-1.29006
H	-3.39921	8.501547	-0.15101
C	-2.59591	6.558715	-0.62616
C	-1.72031	5.866828	-1.47818
H	-0.9437	6.417544	-2.0055
C	-1.83434	4.486573	-1.67136
H	-1.13155	3.97577	-2.32608
C	-2.84629	3.776164	-1.02039
H	-2.94871	2.706577	-1.18137
C	-3.73138	4.453448	-0.17671
H	-4.52505	3.906488	0.327057
C	-3.60496	5.82995	0.022968
H	-4.28689	6.352661	0.687974
C	-2.13388	8.050229	2.14573
O	-3.3156	8.344111	2.343302
N	-1.32899	7.476178	3.071563
H	-0.38046	7.208322	2.821894
C	-1.76479	7.206882	4.434784
H	-2.79366	7.56439	4.509808
C	-1.64294	5.726998	4.828149
H	-0.58515	5.439484	4.76589
H	-1.93365	5.638558	5.886664
C	-2.4686	4.736608	3.986419
H	-2.13177	4.823863	2.944755
C	-2.19459	3.297232	4.448677
H	-2.69581	2.56733	3.802967
H	-1.12368	3.063961	4.431469
H	-2.54773	3.145623	5.480493

C	-3.97391	5.04344	4.0139
H	-4.52796	4.312055	3.410191
H	-4.36605	5.007498	5.041415
H	-4.19503	6.033735	3.603301
C	-6.39975	0.484182	-3.872
H	-6.08928	0.513051	-4.92339
C	-5.2422	-0.07949	-3.0244
H	-5.3349	0.237639	-1.98327
H	-5.25083	-1.1763	-3.03063
C	-3.84668	0.317381	-3.52818
O	-3.62954	0.337042	-4.78482
O	-2.97247	0.560753	-2.65874
C	-7.70166	-0.29971	-3.7728
O	-8.76038	0.208312	-3.39297
N	-7.63722	-1.61508	-4.15428
H	-6.73628	-2.06582	-4.33322
C	-8.83538	-2.42187	-4.29264
H	-9.62278	-1.83053	-4.77291
C	-9.45923	-2.97404	-3.00719
O	-10.414	-3.75105	-3.06618
N	-8.92141	-2.5383	-1.84048
H	-8.10061	-1.95009	-1.88831
C	-9.5434	-2.77492	-0.54689
H	-10.5134	-3.23399	-0.7668
C	-8.70632	-3.71639	0.327996
H	-9.23519	-3.86873	1.27541
H	-7.75067	-3.24566	0.578256
C	-8.38373	-5.07426	-0.28344
O	-7.32035	-5.65475	-0.00995
N	-9.31156	-5.65271	-1.08068
H	-10.0109	-5.1092	-1.58452
H	-9.04902	-6.54132	-1.48909
C	-9.87743	-1.46911	0.219095
O	-10.1061	-1.5227	1.423033
N	-9.9445	-0.34502	-0.53766
H	-9.74774	-0.39135	-1.53327
C	-10.3725	0.943674	-0.00686
H	-10.637	0.784139	1.040689
C	-9.29574	2.04226	-0.14588
H	-8.93864	2.046632	-1.18333
H	-9.79011	3.007663	0.020371
C	-8.13839	1.895068	0.8171
C	-6.99608	1.151856	0.488822
H	-6.92713	0.701098	-0.49743
C	-5.93609	0.991946	1.382736
H	-5.03699	0.447531	1.103374
C	-6.02573	1.58118	2.646086

O	-4.98282	1.368911	3.531388
H	-4.9813	2.073933	4.199483
C	-7.14698	2.334739	2.999187
H	-7.2076	2.79759	3.981884
C	-8.18745	2.489392	2.084396
H	-9.05332	3.084052	2.366348
C	0.924273	5.215248	-4.5376
H	1.367037	5.808493	-3.72947
H	-0.16393	5.268674	-4.44274
C	1.407567	3.778472	-4.37869
O	2.389447	3.408443	-5.10289
O	0.831905	3.052058	-3.52068
C	10.02871	-3.29742	-5.27598
C	8.933466	-2.97343	-4.31413
C	7.571812	-2.90513	-4.45496
N	9.159535	-2.65305	-2.98293
C	7.971302	-2.40616	-2.37886
N	6.98751	-2.54523	-3.25367
H	10.62002	-4.1624	-4.94911
H	9.603924	-3.53737	-6.25456
H	6.969394	-3.11254	-5.32689
H	7.87047	-2.19443	-1.32024
H	10.05733	-2.62226	-2.52064
C	6.817933	-6.3323	2.049914
H	7.623357	-6.5962	1.349702
C	5.782092	-5.47752	1.301686
H	6.248554	-4.56478	0.918516
H	5.46526	-6.03413	0.410344
C	4.545057	-5.11636	2.135253
H	4.098099	-6.02688	2.560018
H	4.828246	-4.48035	2.983603
C	3.505164	-4.38546	1.276377
H	3.932123	-3.46934	0.866803
H	3.226114	-5.00066	0.413407
N	2.273143	-4.05182	1.996366
H	1.62799	-4.80635	2.194942
C	1.893437	-2.84163	2.444656
N	2.680311	-1.77707	2.43325
H	3.635093	-1.79288	2.071751
H	2.275055	-0.84776	2.672694
N	0.610442	-2.70889	2.879519
H	0.103203	-3.58849	3.049505
H	0.431218	-2.00172	3.604219
C	7.478533	-5.72532	3.288656
O	7.577934	-6.3597	4.337813
N	7.998154	-4.46672	3.155243
H	7.969076	-3.96918	2.262127

C	8.795235	-3.93058	4.238365
H	8.36034	-4.27816	5.180046
C	8.919828	-2.40969	4.201198
O	8.993985	-1.75788	3.155429
N	9.035026	-1.82205	5.418017
H	8.911042	-2.39234	6.241905
C	9.271313	-0.38537	5.579649
H	10.03131	-0.09103	4.849638
C	8.013734	0.474894	5.378984
H	8.303611	1.531467	5.426551
H	7.641562	0.294873	4.366116
C	6.913255	0.202665	6.408385
H	6.681689	-0.86832	6.459845
H	7.235134	0.514911	7.409364
S	5.351922	1.113959	6.09347
C	4.738331	0.201901	4.633711
H	3.798463	0.665063	4.321726
H	5.440495	0.251644	3.796451
H	4.54559	-0.84627	4.885576
O	3.032476	-2.59485	-2.24915
O	7.825743	-2.63964	0.794428
Fe	4.85619	-2.44116	-2.69075
C	-2.32111	-2.6	-6.95233
H	-2.37245	-2.7366	-8.04102
H	-1.80707	-3.47852	-6.5497
C	-3.75297	-2.67326	-6.40731
H	-4.31268	-3.38878	-7.02183
C	-3.95303	-3.14286	-4.93501
O	-2.96703	-3.55991	-4.3079
O	-5.16476	-3.07678	-4.53935
N	-4.50321	-1.36703	-6.51725
H	-4.14675	-0.61223	-5.76904
H	-5.47745	-1.56456	-6.27241
H	-4.4658	-0.96818	-7.45849
C	-1.47649	-1.3407	-6.68373
H	-2.01046	-0.43992	-7.0324
H	-0.60473	-1.41047	-7.36088
C	-1.00845	-1.1148	-5.28348
H	2.626475	-3.23754	-2.85401
H	-1.06547	-1.91086	-4.54832
C	-0.26054	0.141687	-4.99427
H	0.810722	0.053947	-5.23632
H	-0.65158	0.966635	-5.60057
N	-0.34004	0.578793	-3.57133
H	9.822966	-4.33245	4.212659
H	9.691592	-0.23846	6.58095
H	-1.92509	-8.16899	2.757566

H	6.37115	-7.27217	2.389253
H	-6.80664	-2.77122	4.93165
H	-3.90131	0.129321	6.562309
H	10.71906	-2.4548	-5.41085
H	7.16578	2.189826	-6.12854
H	1.239466	5.634699	-5.49677
H	4.473279	6.285133	-3.99847
H	-1.15759	7.806712	5.126839
H	-6.62462	1.513054	-3.58129
H	-8.61563	-3.27341	-4.94186
H	-11.2764	1.258156	-0.54462
H	8.317111	-2.14174	1.48183
H	6.898409	-2.32355	0.883784
C	4.968568	-4.88629	-3.30888
O	5.232179	-4.52884	-2.12281
O	4.622394	-4.00662	-4.16851
C	5.099644	-6.33247	-3.72489
H	6.08764	-6.4801	-4.17777
H	4.3456	-6.58494	-4.47498
H	5.01525	-6.99229	-2.85866
H	0.134745	1.513351	-3.48635
H	-1.34152	0.660546	-3.26907
H	0.152756	-0.07613	-2.91184
O	1.107939	-0.77812	-1.6759
H	1.745718	-1.5245	-1.79647
H	0.627494	-0.91585	-0.82058
O	-3.02802	-0.86009	-0.18817
H	-3.01523	-0.25933	-0.96562
H	-2.10684	-0.83144	0.178283
O	-0.39858	-0.95858	0.623436
H	-0.19911	-1.63064	1.306136
H	-0.10998	-0.07777	1.036614

^{5}**TS2**$_{B,C5}$

C	-3.70152	1.192674	5.992086
H	-3.08469	1.115176	5.088052
C	-2.855	0.837134	7.218715
H	-3.3942	1.112274	8.132462
H	-2.71425	-0.24554	7.261135
C	-1.48348	1.522869	7.221267
H	-1.58927	2.612889	7.132743
H	-0.98634	1.344567	8.184773
C	-0.53818	1.02793	6.120308
O	-0.80194	0.025024	5.438269
N	0.604658	1.719351	5.962874
H	0.763679	2.57508	6.474848
H	1.215707	1.506222	5.162656
C	-4.91413	0.28193	5.789518
O	-5.11827	-0.73145	6.472822
N	-5.73467	0.648192	4.774729
H	-5.46983	1.433024	4.186386
C	-6.82211	-0.19488	4.27634
H	-7.33421	0.392704	3.508963
C	-6.36165	-1.54817	3.695695
H	-6.12688	-2.22046	4.527618
H	-7.21491	-1.9883	3.160979
C	-5.13393	-1.44788	2.782018
H	-4.34251	-0.91402	3.319202
H	-5.34649	-0.85288	1.886698
C	-4.55275	-2.80797	2.371244
H	-3.4941	-2.70462	2.125735
H	-4.61259	-3.51368	3.207698
N	-5.20491	-3.43653	1.216842
H	-6.04635	-4.00124	1.359282
C	-4.60621	-3.57342	0.022905
N	-3.54714	-2.84357	-0.32244
H	-3.39662	-1.87396	0.018415
H	-3.00355	-3.1609	-1.13403
N	-5.08961	-4.48509	-0.84878
H	-5.75241	-5.17655	-0.52281
H	-4.74979	-4.49001	-1.81122
C	-2.20205	-6.14555	4.439287
H	-1.2275	-6.45527	4.836904
H	-2.12187	-6.14271	3.344644
C	-2.59984	-4.79995	4.962035
N	-1.84163	-3.66713	4.720691
C	-2.46884	-2.67453	5.336008
H	-2.13211	-1.64621	5.373464
N	-3.59003	-3.10429	5.960916

H	-4.25561	-2.47266	6.408459
C	-3.69298	-4.46064	5.727951
H	-4.50076	-5.05489	6.128234
C	7.099514	1.799568	-6.10429
C	6.938403	2.712712	-4.88632
C	5.831845	1.061568	-6.60759
O	7.653382	2.555845	-3.89052
C	5.093102	0.307215	-5.54169
C	5.195315	-0.96207	-5.01024
N	4.099944	0.93091	-4.81942
C	3.646699	0.057707	-3.90541
N	4.278689	-1.11005	-3.98949
H	7.507866	2.39362	-6.93293
H	5.140023	1.775151	-7.07141
H	6.143924	0.373721	-7.40173
H	5.862882	-1.76675	-5.28326
H	2.902047	0.300625	-3.16601
H	3.69784	1.887767	-5.05414
N	6.039132	3.732742	-4.9717
H	5.238128	3.676836	-5.59903
C	5.877836	4.675337	-3.86877
H	6.868846	4.878309	-3.45185
C	4.942032	4.188159	-2.75112
O	3.909861	4.804661	-2.45829
N	5.327857	3.049819	-2.13552
C	4.457754	2.337519	-1.20802
C	4.076394	3.242639	-0.01817
C	5.150077	1.047968	-0.73734
O	4.91544	3.795826	0.681455
C	4.265779	0.218582	0.213277
C	4.569667	-1.27904	0.203684
O	4.740293	-1.86415	1.311305
O	4.608738	-1.8739	-0.92205
H	6.216382	2.638874	-2.41625
H	3.538298	2.069427	-1.74391
H	5.392533	0.448343	-1.62118
H	6.091023	1.316453	-0.24244
H	3.215244	0.312886	-0.0915
H	4.343622	0.589809	1.238475
N	2.730088	3.374636	0.164472
H	2.112042	2.798003	-0.39042
C	2.125746	4.125337	1.25811
H	1.045685	4.062076	1.085803
C	2.36856	3.469843	2.622699
H	3.432786	3.28579	2.784109
H	2.032669	4.157639	3.407508
C	1.578419	2.158711	2.736481

O	0.520403	2.071479	2.059297
O	2.033968	1.265151	3.525064
C	2.43496	5.629187	1.239362
O	2.162268	6.325454	2.225174
N	2.865514	6.167067	0.069101
H	3.159368	5.557321	-0.69365
C	3.220759	7.583472	0.00044
H	3.702032	7.85461	0.946652
C	4.189832	7.854454	-1.15396
H	5.12955	7.317694	-0.99272
H	4.391004	8.927363	-1.20174
H	3.770703	7.525117	-2.11014
C	2.004842	8.53132	-0.07785
O	2.159297	9.74284	0.028815
N	0.789851	7.933549	-0.2457
H	0.771983	6.92872	-0.36048
C	-0.46614	8.652529	-0.16013
H	-0.19853	9.714772	-0.18893
C	-1.3956	8.355122	-1.36121
H	-0.90804	8.74915	-2.26004
H	-2.31398	8.924665	-1.19656
C	-1.71986	6.887279	-1.55826
C	-0.91254	6.076602	-2.37037
H	-0.05657	6.514412	-2.87894
C	-1.19718	4.71994	-2.55012
H	-0.54729	4.12208	-3.1853
C	-2.30682	4.151951	-1.92045
H	-2.5456	3.10037	-2.06317
C	-3.12013	4.947251	-1.10805
H	-3.98648	4.510742	-0.61697
C	-2.82792	6.300424	-0.92578
H	-3.45514	6.916696	-0.28812
C	-1.2085	8.445016	1.184456
O	-2.38323	8.806092	1.297474
N	-0.47931	7.909808	2.191452
H	0.476181	7.606046	2.022767
C	-0.99692	7.747024	3.542772
H	-1.92581	8.318302	3.599479
C	-1.2109	6.277295	3.938502
H	-0.24975	5.755619	3.83653
H	-1.46949	6.251779	5.009195
C	-2.28496	5.511161	3.143509
H	-2.02174	5.567279	2.07825
C	-2.28256	4.028145	3.544851
H	-3.06337	3.475016	3.009838
H	-1.32378	3.547824	3.319748
H	-2.47593	3.918933	4.622623

C	-3.68414	6.126094	3.306708
H	-4.42653	5.527659	2.761344
H	-3.98467	6.153906	4.364496
H	-3.72852	7.143794	2.90673
C	-5.47865	-0.8402	-4.71771
H	-4.89871	-1.3139	-5.51801
C	-4.65435	-0.83059	-3.41769
H	-5.0989	-0.14676	-2.6915
H	-4.64245	-1.83005	-2.96807
C	-3.18784	-0.43679	-3.59422
O	-2.5255	-0.93413	-4.57678
O	-2.68737	0.324342	-2.73355
C	-6.80626	-1.57242	-4.56762
O	-7.89687	-1.00486	-4.63592
N	-6.70755	-2.92101	-4.34034
H	-5.79118	-3.34726	-4.20542
C	-7.87835	-3.77802	-4.38896
H	-8.51007	-3.49313	-5.23743
C	-8.80397	-3.76931	-3.16836
O	-9.79866	-4.49603	-3.14919
N	-8.47323	-2.92524	-2.15954
H	-7.59427	-2.42921	-2.22404
C	-9.35122	-2.67389	-1.02643
H	-10.2977	-3.16486	-1.27691
C	-8.79069	-3.25378	0.279976
H	-9.51995	-3.06898	1.076462
H	-7.87902	-2.71974	0.563702
C	-8.44938	-4.73856	0.253936
O	-7.48476	-5.17874	0.901263
N	-9.26071	-5.56714	-0.44331
H	-9.82138	-5.23861	-1.22758
H	-8.98405	-6.54107	-0.46257
C	-9.70842	-1.17856	-0.83594
O	-10.2057	-0.81721	0.224918
N	-9.47961	-0.36882	-1.90154
H	-9.1261	-0.76518	-2.76743
C	-9.92615	1.02043	-1.93661
H	-10.4897	1.195506	-1.01765
C	-8.77529	2.040501	-2.07719
H	-8.12797	1.720783	-2.90385
H	-9.22626	2.991385	-2.38942
C	-7.95821	2.264827	-0.82176
C	-6.74854	1.594914	-0.5972
H	-6.38385	0.89648	-1.34587
C	-5.98052	1.812193	0.547478
H	-5.02482	1.310935	0.680834
C	-6.44074	2.716461	1.507474

O	-5.67186	2.898094	2.642773
H	-5.93689	3.722488	3.081842
C	-7.64528	3.397686	1.314837
H	-8.0002	4.105265	2.061666
C	-8.38736	3.172343	0.155956
H	-9.31581	3.720296	0.011357
C	1.677988	5.064065	-5.21619
H	2.081405	5.592923	-4.34385
H	0.588535	5.151662	-5.20767
C	2.122918	3.611352	-5.11098
O	3.262228	3.32354	-5.60038
O	1.378785	2.779071	-4.52278
C	8.080085	-5.96572	-4.7202
C	7.206173	-5.13925	-3.83492
C	5.995009	-4.52897	-4.04061
N	7.52833	-4.83153	-2.52147
C	6.535319	-4.06909	-1.99342
N	5.590867	-3.86703	-2.89541
H	8.244621	-6.97259	-4.31437
H	7.612558	-6.07801	-5.70252
H	5.372855	-4.5389	-4.92335
H	6.536786	-3.7291	-0.9653
H	8.353459	-5.12972	-2.02103
C	4.98377	-7.16849	2.679486
H	5.64359	-7.72039	1.994713
C	4.205561	-6.11482	1.875488
H	4.902912	-5.42309	1.392188
H	3.678084	-6.62777	1.059827
C	3.186287	-5.32586	2.709009
H	2.435168	-6.01339	3.12603
H	3.681681	-4.84712	3.562975
C	2.49466	-4.24928	1.863694
H	3.22446	-3.50819	1.532915
H	2.070047	-4.68667	0.951741
N	1.414698	-3.57155	2.590868
H	0.605235	-4.13191	2.826478
C	1.348326	-2.28544	2.979674
N	2.344897	-1.42389	2.849402
H	3.235315	-1.65806	2.39686
H	2.180253	-0.42781	3.091449
N	0.153818	-1.85542	3.477426
H	-0.48516	-2.58932	3.816046
H	0.148925	-1.026	4.081722
C	5.854342	-6.67979	3.839638
O	5.927048	-7.31252	4.892752
N	6.582255	-5.54169	3.642668
H	6.577739	-5.03961	2.750629

C	7.504907	-5.13838	4.68326
H	6.981885	-5.12155	5.646726
C	8.163479	-3.79198	4.394982
O	8.408118	-3.38286	3.257204
N	8.534078	-3.09524	5.498964
H	8.257735	-3.44213	6.406094
C	9.224427	-1.807	5.410785
H	9.959642	-1.88526	4.605143
C	8.282615	-0.6244	5.13142
H	8.889357	0.272827	4.959658
H	7.750289	-0.82931	4.197129
C	7.289894	-0.3549	6.266156
H	6.735232	-1.26504	6.526839
H	7.82029	-0.02856	7.169194
S	6.07586	0.974322	5.911604
C	4.972368	0.107978	4.740679
H	4.172656	0.801473	4.465757
H	5.493809	-0.19835	3.828914
H	4.521679	-0.77066	5.214498
O	1.740259	-1.92891	-2.38761
O	6.656697	-3.88324	1.161167
Fe	3.746098	-2.67896	-2.59097
C	-0.42966	-3.9505	-4.80351
H	0.046058	-4.5719	-5.57047
H	-0.13417	-4.37092	-3.8397
C	-1.94836	-4.14914	-4.88575
H	-2.14539	-5.16215	-5.25347
C	-2.74599	-4.00024	-3.55455
O	-2.0882	-3.83371	-2.51301
O	-4.00747	-4.07985	-3.69711
N	-2.66083	-3.21879	-5.84227
H	-2.67228	-2.21445	-5.41469
H	-3.6383	-3.52895	-5.85958
H	-2.27345	-3.22282	-6.789
C	0.19738	-2.55436	-4.94681
H	0.007249	-2.12089	-5.94085
H	1.285285	-2.72749	-4.90985
C	-0.07569	-1.51031	-3.92461
H	1.380739	-2.73568	-1.975
H	-0.66681	-1.73039	-3.04657
C	0.274676	-0.114	-4.28929
H	1.335729	-0.04259	-4.5398
H	-0.2776	0.141487	-5.19955
N	-0.04513	0.903626	-3.26708
H	8.309564	-5.88286	4.797489
H	9.767905	-1.6623	6.351461
H	-2.93526	-6.9084	4.71944

H	4.30435	-7.90642	3.118602
H	-7.5354	-0.37534	5.087768
H	-4.03823	2.23684	6.041023
H	9.065483	-5.50608	-4.87273
H	7.85823	1.068809	-5.81395
H	2.088247	5.532678	-6.11498
H	5.453601	5.601026	-4.25998
H	-0.28207	8.202462	4.239843
H	-5.70627	0.179217	-5.03831
H	-7.55553	-4.81016	-4.54939
H	-10.6106	1.138184	-2.78624
H	7.396653	-3.53278	1.696821
H	5.941968	-3.2035	1.232114
C	2.530477	-4.9498	-3.00225
O	2.607847	-4.5468	-1.80558
O	2.858314	-4.20528	-3.98472
C	2.086224	-6.37926	-3.27244
H	2.957192	-7.03688	-3.16239
H	1.703023	-6.49387	-4.28981
H	1.337271	-6.69622	-2.54135
H	0.321595	1.816318	-3.62657
H	-1.07677	0.90063	-3.10112
H	0.416923	0.690258	-2.32594
O	1.146619	0.230474	-0.95929
H	1.431195	-0.65892	-1.32146
H	0.544045	0.058236	-0.19057
O	-3.14939	-0.14105	0.150048
H	-3.03296	0.152614	-0.7762
H	-2.26183	-0.00919	0.576961
O	-0.61518	-0.07713	1.15887
H	-0.4935	-0.78673	1.822499
H	-0.24791	0.751483	1.614401

[5]**Pr**$_{B,C5}$

C	-3.57265	0.846268	6.057841
H	-2.99065	0.907598	5.129963
C	-2.64325	0.509198	7.228785
H	-3.14276	0.732706	8.178236
H	-2.44276	-0.56441	7.23399
C	-1.31122	1.266965	7.168312
H	-1.47499	2.352735	7.140945
H	-0.73417	1.068147	8.082122
C	-0.43679	0.858941	5.977381
O	-0.63948	-0.20195	5.365085
N	0.569355	1.690917	5.657347
H	0.680753	2.574218	6.133373
H	1.139153	1.502849	4.819465
C	-4.68006	-0.1851	5.822144
O	-4.79943	-1.21424	6.501717
N	-5.50261	0.10604	4.787387
H	-5.30475	0.928132	4.222518
C	-6.50955	-0.82051	4.270627
H	-7.0934	-0.25969	3.534817
C	-5.92675	-2.09538	3.627541
H	-5.59892	-2.7721	4.423864
H	-6.74764	-2.60318	3.102054
C	-4.74784	-1.83768	2.680438
H	-3.95862	-1.31655	3.234465
H	-5.03005	-1.1748	1.854642
C	-4.12462	-3.11777	2.108569
H	-3.11822	-2.9116	1.740259
H	-4.02808	-3.87367	2.897643
N	-4.87911	-3.71536	1.002343
H	-5.70745	-4.27663	1.211717
C	-4.41541	-3.80763	-0.25674
N	-3.38836	-3.07564	-0.68697
H	-3.22317	-2.11138	-0.33423
H	-2.97787	-3.34744	-1.58936
N	-4.99171	-4.68828	-1.10105
H	-5.69021	-5.3267	-0.74232
H	-4.86042	-4.55764	-2.10632
C	-1.82616	-6.46616	4.221988
H	-0.87036	-6.81691	4.631661
H	-1.70456	-6.38004	3.134703
C	-2.2341	-5.16033	4.830754
N	-1.46888	-4.01478	4.688773
C	-2.10946	-3.06702	5.359954
H	-1.78648	-2.03799	5.460876
N	-3.24359	-3.53849	5.927627

H	-3.92871	-2.93566	6.385538
C	-3.34306	-4.87371	5.594725
H	-4.16092	-5.49256	5.932206
C	6.646772	1.862324	-6.37656
C	6.632004	2.755416	-5.13573
C	5.344476	1.086407	-6.68789
O	7.458038	2.593565	-4.23209
C	4.851642	0.228929	-5.55863
C	5.254747	-0.98024	-5.04288
N	3.798217	0.637735	-4.76352
C	3.595404	-0.29864	-3.82392
N	4.462418	-1.30387	-3.95651
H	6.898366	2.482114	-7.24723
H	4.545293	1.780332	-6.97099
H	5.53614	0.462384	-7.56904
H	6.060515	-1.62513	-5.36284
H	2.834012	-0.24171	-3.06083
H	3.301677	1.57165	-4.88548
N	5.727265	3.777486	-5.11459
H	4.833399	3.675546	-5.59768
C	5.729026	4.731169	-4.00974
H	6.766591	4.869454	-3.69292
C	4.870133	4.289836	-2.81724
O	3.895994	4.957703	-2.45311
N	5.245307	3.126	-2.24064
C	4.368966	2.36932	-1.34745
C	3.89937	3.238049	-0.16144
C	5.12221	1.128526	-0.83808
O	4.680969	3.649984	0.688168
C	4.247739	0.196162	0.023126
C	4.823736	-1.21011	0.170305
O	4.943202	-1.72826	1.308681
O	5.164551	-1.82651	-0.90405
H	6.078534	2.673867	-2.61137
H	3.490081	2.048549	-1.92288
H	5.493362	0.571187	-1.70493
H	5.98747	1.471408	-0.25941
H	3.260062	0.070449	-0.44222
H	4.095281	0.619852	1.018906
N	2.553776	3.441842	-0.12458
H	2.005089	3.152908	-0.92341
C	1.85485	4.10585	0.9693
H	0.791444	4.0265	0.71847
C	2.047975	3.380182	2.30492
H	3.108603	3.315868	2.556563
H	1.567376	3.970139	3.093412
C	1.435475	1.976635	2.283046

O	0.562034	1.732711	1.416711
O	1.858883	1.158906	3.17269
C	2.137972	5.613117	1.085113
O	1.798698	6.232347	2.100967
N	2.640934	6.2335	-0.01203
H	3.005516	5.673019	-0.7824
C	2.968033	7.657937	0.027335
H	3.429619	7.872893	0.997949
C	3.949519	8.024432	-1.08965
H	4.893759	7.487366	-0.95912
H	4.137889	9.099981	-1.05512
H	3.545225	7.765123	-2.07377
C	1.737361	8.592013	-0.00916
O	1.878362	9.796424	0.1721
N	0.532256	7.993671	-0.22856
H	0.521969	6.996651	-0.39731
C	-0.73627	8.690328	-0.12361
H	-0.49734	9.758163	-0.17452
C	-1.68406	8.341781	-1.29702
H	-1.21876	8.708793	-2.21908
H	-2.60571	8.906414	-1.13465
C	-1.99202	6.862883	-1.42832
C	-1.15353	6.016671	-2.17046
H	-0.29343	6.437384	-2.68744
C	-1.40898	4.64567	-2.27018
H	-0.72518	4.01915	-2.83808
C	-2.52827	4.099479	-1.63728
H	-2.74264	3.037091	-1.72435
C	-3.37662	4.931361	-0.90107
H	-4.25188	4.512875	-0.40965
C	-3.10833	6.297811	-0.79153
H	-3.76141	6.939775	-0.2071
C	-1.45193	8.488934	1.236961
O	-2.61427	8.878055	1.377166
N	-0.71636	7.922123	2.221783
H	0.221396	7.582149	2.024266
C	-1.21246	7.750723	3.579426
H	-2.17272	8.267505	3.632729
C	-1.33219	6.275815	3.994951
H	-0.33687	5.81955	3.910133
H	-1.60269	6.247139	5.062507
C	-2.33824	5.42887	3.193526
H	-2.06347	5.49401	2.132195
C	-2.24153	3.953454	3.60959
H	-2.94559	3.334029	3.043048
H	-1.23767	3.549199	3.436759
H	-2.47224	3.833579	4.679084

C	-3.77918	5.945977	3.329178
H	-4.46933	5.305681	2.762885
H	-4.10168	5.949685	4.381047
H	-3.88555	6.961394	2.934659
C	-6.04704	-0.29948	-4.27111
H	-5.60094	-0.54423	-5.2423
C	-4.98261	-0.50173	-3.17652
H	-5.24272	0.053057	-2.27243
H	-4.90881	-1.56022	-2.89848
C	-3.56832	-0.08891	-3.61122
O	-3.19951	-0.36188	-4.79916
O	-2.84112	0.461031	-2.74189
C	-7.30607	-1.13306	-4.08803
O	-8.42685	-0.63434	-3.95161
N	-7.12548	-2.49174	-4.09592
H	-6.18509	-2.89096	-4.11631
C	-8.25436	-3.40113	-4.15978
H	-8.99235	-3.02766	-4.87863
C	-9.03189	-3.64091	-2.86238
O	-9.96367	-4.44745	-2.84031
N	-8.64912	-2.91095	-1.78535
H	-7.8339	-2.3203	-1.8805
C	-9.41994	-2.86176	-0.55313
H	-10.3509	-3.39731	-0.76843
C	-8.68519	-3.53932	0.610561
H	-9.32957	-3.49887	1.495623
H	-7.77957	-2.97766	0.858372
C	-8.26194	-4.9827	0.367218
O	-7.21184	-5.42775	0.858822
N	-9.09208	-5.77867	-0.34713
H	-9.74182	-5.40025	-1.03479
H	-8.75832	-6.71981	-0.51583
C	-9.85931	-1.42951	-0.15669
O	-10.2408	-1.21819	0.989925
N	-9.83529	-0.50601	-1.15047
H	-9.53292	-0.77123	-2.08401
C	-10.3578	0.845361	-0.98231
H	-10.7451	0.911465	0.036669
C	-9.30562	1.943336	-1.24973
H	-8.82073	1.729144	-2.21046
H	-9.84866	2.88843	-1.3783
C	-8.2702	2.105197	-0.15763
C	-7.05002	1.416674	-0.19184
H	-6.83821	0.764118	-1.03424
C	-6.08941	1.562066	0.809988
H	-5.1303	1.052708	0.751697
C	-6.36073	2.410619	1.885795

O	-5.40568	2.511926	2.882337
H	-5.54723	3.33275	3.381618
C	-7.5678	3.110293	1.948915
H	-7.77367	3.77212	2.787797
C	-8.50625	2.957054	0.929059
H	-9.43825	3.515247	0.9823
C	1.600295	5.042146	-4.94019
H	2.101508	5.644376	-4.17367
H	0.5239	5.21974	-4.86617
C	1.927606	3.579975	-4.66353
O	2.880143	3.060798	-5.33208
O	1.268144	2.982527	-3.7669
C	9.094524	-5.7351	-4.60111
C	8.109631	-5.02039	-3.73576
C	6.897025	-4.43961	-4.00194
N	8.30102	-4.81095	-2.3779
C	7.238766	-4.13339	-1.87732
N	6.365859	-3.89258	-2.84629
H	9.267136	-6.76447	-4.26205
H	8.721317	-5.78264	-5.62769
H	6.375077	-4.38161	-4.94654
H	7.143127	-3.8528	-0.83019
H	9.103109	-5.10442	-1.83784
C	5.67145	-6.86014	3.252457
H	6.366351	-7.4424	2.630443
C	4.784204	-6.00345	2.335137
H	5.404431	-5.32466	1.73942
H	4.295274	-6.67804	1.617661
C	3.70559	-5.2036	3.078435
H	3.053053	-5.88958	3.637548
H	4.166062	-4.5355	3.816576
C	2.861408	-4.37125	2.104265
H	3.479666	-3.61006	1.623089
H	2.473357	-5.01122	1.300985
N	1.716418	-3.72164	2.741414
H	0.961854	-4.31956	3.056244
C	1.53096	-2.40593	2.962898
N	2.457719	-1.48554	2.760732
H	3.381153	-1.68494	2.368068
H	2.191775	-0.48336	2.871705
N	0.289101	-2.02281	3.367864
H	-0.30976	-2.78146	3.719656
H	0.210688	-1.16484	3.92737
C	6.51481	-6.14022	4.307216
O	6.666934	-6.61363	5.432007
N	7.130772	-4.98015	3.933856
H	7.060238	-4.60913	2.984597

C	8.035079	-4.3555	4.876747
H	7.540699	-4.27631	5.851587
C	8.52965	-2.99355	4.397145
O	8.693016	-2.71167	3.206445
N	8.851561	-2.12776	5.389053
H	8.639357	-2.38583	6.341995
C	9.377779	-0.7876	5.121405
H	10.06897	-0.87169	4.278529
C	8.284829	0.242189	4.7908
H	8.766743	1.181807	4.495401
H	7.733832	-0.11836	3.916034
C	7.325778	0.508076	5.955728
H	6.918701	-0.43102	6.351629
H	7.852261	1.005819	6.779164
S	5.918962	1.610212	5.53983
C	4.902564	0.468783	4.538256
H	4.01121	1.014376	4.216106
H	5.433636	0.12042	3.647538
H	4.5873	-0.39356	5.135642
O	0.794334	-2.12	-3.72655
O	6.99914	-3.65265	1.226836
Fe	4.561961	-2.79827	-2.49233
C	-1.2072	-3.66327	-5.81762
H	-1.02026	-4.10522	-6.80576
H	-0.73083	-4.32736	-5.08968
C	-2.7201	-3.74812	-5.55965
H	-3.10023	-4.64833	-6.05685
C	-3.22235	-3.83882	-4.08684
O	-2.37651	-3.96602	-3.18959
O	-4.49403	-3.79409	-3.9755
N	-3.51668	-2.60284	-6.15534
H	-3.37866	-1.66829	-5.60612
H	-4.50224	-2.85039	-6.01963
H	-3.32803	-2.45512	-7.14981
C	-0.52463	-2.28157	-5.80307
H	-1.07409	-1.59381	-6.46068
H	0.468469	-2.39925	-6.25404
C	-0.32649	-1.60588	-4.43372
H	0.907135	-3.10535	-3.81416
H	-1.234	-1.72495	-3.83189
C	-0.06581	-0.11279	-4.64748
H	0.922018	0.032284	-5.09134
H	-0.83094	0.314725	-5.29527
N	-0.11782	0.65845	-3.37026
H	8.920951	-4.98967	5.044168
H	9.954057	-0.48075	6.001659
H	-2.57573	-7.24011	4.414506

H	5.069495	-7.58602	3.808409
H	-7.18486	-1.10247	5.085423
H	-4.02888	1.835947	6.196385
H	10.06571	-5.22461	-4.62382
H	7.467744	1.158251	-6.21939
H	1.965513	5.350325	-5.9234
H	5.327481	5.683753	-4.35979
H	-0.5248	8.258058	4.269155
H	-6.35972	0.746579	-4.3161
H	-7.90426	-4.37295	-4.51775
H	-11.1974	0.981495	-1.67649
H	7.716404	-3.19979	1.717875
H	6.244991	-3.01381	1.246809
C	2.484494	-4.94255	-3.2911
O	1.451376	-4.7137	-3.93767
O	3.112983	-4.05873	-2.57934
C	3.072014	-6.34684	-3.26725
H	3.089016	-6.71883	-2.23676
H	4.110427	-6.32344	-3.61649
H	2.484747	-7.02306	-3.89124
H	0.332568	1.595148	-3.51265
H	-1.12201	0.742757	-3.08743
H	0.40385	0.177997	-2.59505
O	1.269666	-0.78584	-1.41032
H	1.086383	-1.55949	-1.98933
H	0.688523	-0.84712	-0.60869
O	-3.06305	-0.39866	-0.02127
H	-3.02274	-0.00055	-0.91935
H	-2.13496	-0.35034	0.322782
O	-0.42318	-0.59072	0.764806
H	-0.34106	-1.17639	1.543934
H	-0.0582	0.299988	1.085635

Model 3 structures:

^{1}**Re**$_{C}$

C	-0.65538	-6.41822	-2.89492
H	-0.38917	-5.98349	-1.91942
C	-0.62783	-5.37169	-4.00559
H	-0.86387	-5.87026	-4.9582
H	-1.42247	-4.63488	-3.83961
C	0.69163	-4.62009	-4.14219
H	0.971848	-4.15042	-3.18521
H	1.522863	-5.29104	-4.40547
C	0.583267	-3.48951	-5.15815
O	-0.45461	-2.82401	-5.26241
N	1.671563	-3.24796	-5.90761
H	2.600378	-3.64356	-5.71106
H	1.618525	-2.48145	-6.57012
C	-1.99873	-7.1343	-2.80281
O	-2.64409	-7.42196	-3.81704
N	-2.40282	-7.47237	-1.56277
H	-1.95314	-7.07555	-0.73189
C	-3.55655	-8.32146	-1.30325
H	-3.43969	-8.69006	-0.27318
C	-4.92237	-7.64349	-1.4546
H	-5.09509	-7.44329	-2.52449
H	-5.68928	-8.37829	-1.15345
C	-5.08046	-6.36031	-0.64055
H	-4.3668	-5.59685	-0.98939
H	-4.82796	-6.53904	0.417405
C	-6.494	-5.78891	-0.73973
H	-6.78837	-5.6961	-1.80364
H	-7.21038	-6.48978	-0.27212
N	-6.56033	-4.4915	-0.08706
H	-5.66348	-4.00251	0.086414
C	-7.63443	-3.70853	-0.04718
N	-8.83126	-4.13477	-0.54676
H	-8.99553	-5.13258	-0.6073
H	-9.63018	-3.54903	-0.30119
N	-7.5369	-2.47758	0.438544
H	-6.68022	-2.26594	0.987444
H	-8.3789	-1.89579	0.474534
C	-7.46048	-2.89753	-4.48128
H	-7.82315	-3.93691	-4.46266
C	-6.16496	-2.78374	-3.64697
H	-5.88171	-1.72616	-3.56652
H	-6.31999	-3.14207	-2.61898
C	-5.06052	-3.55862	-4.28587
N	-4.37166	-3.08382	-5.38828

C	-3.56777	-4.06812	-5.77412
H	-2.84944	-4.02325	-6.59043
N	-3.71556	-5.15374	-4.97918
H	-3.18662	-6.0362	-4.95768
C	-4.65161	-4.84651	-4.02279
H	-4.93395	-5.55507	-3.25565
C	-8.52795	-1.93293	-4.01614
O	-8.50557	-0.73955	-4.32297
N	-9.48867	-2.45677	-3.21256
H	-9.34963	-3.39009	-2.83811
C	-10.525	-1.63275	-2.5948
H	-10.8037	-0.87534	-3.34194
C	-11.7444	-2.46247	-2.22824
H	-12.5178	-1.8282	-1.7733
H	-11.4971	-3.24752	-1.49848
H	-12.1618	-2.938	-3.1272
C	-9.90201	-0.91083	-1.38203
O	-9.97349	-1.38646	-0.24143
N	-9.23791	0.204589	-1.71767
H	-9.15951	0.368475	-2.7229
C	-8.43293	1.061244	-0.85979
H	-8.28351	0.575837	0.114973
C	-7.08531	1.380992	-1.53264
H	-7.30304	1.526273	-2.60744
C	-6.48148	2.681835	-1.00201
H	-7.15642	3.539037	-1.16317
H	-6.26597	2.579793	0.072422
H	-5.52922	2.890448	-1.50998
C	-6.10003	0.217153	-1.39473
H	-6.55747	-0.74058	-1.67367
H	-5.75318	0.135116	-0.35429
H	-5.22052	0.370743	-2.0399
C	-2.45346	1.472741	5.955864
H	-1.51947	2.052225	6.017303
C	-2.10791	-0.0166	5.944292
H	-1.35791	-0.1848	5.148941
C	-1.49342	-0.45259	7.2703
H	-1.27186	-1.532	7.262293
H	-2.19781	-0.27025	8.096208
H	-0.55793	0.092576	7.469508
O	-3.29041	-0.74202	5.652715
H	-3.03905	-1.64636	5.335959
C	-3.21744	1.913126	4.735394
O	-4.18179	2.698211	4.831403
N	-2.85966	1.415593	3.539663
H	-2.10784	0.727795	3.436421
C	-3.73207	1.535192	2.394087

H	-3.9147	2.583091	2.122218
H	-3.23844	1.054446	1.539568
C	-5.08593	0.831753	2.580729
O	-6.04886	1.155739	1.878167
N	-5.10136	-0.12877	3.514154
H	-4.28354	-0.27754	4.117377
C	-6.21844	-1.01263	3.770647
H	-7.05975	-0.68809	3.142187
C	-5.82385	-2.45445	3.450915
H	-5.00581	-2.78683	4.110385
H	-6.6886	-3.1149	3.628994
O	-5.44853	-2.60318	2.100088
H	-4.59157	-2.16539	1.87292
C	-1.28631	8.229368	4.67007
H	-0.60172	7.884627	5.45906
C	-2.11957	7.065681	4.130133
H	-2.94718	7.466749	3.524325
H	-2.58671	6.550915	4.988562
C	-1.35016	6.036364	3.285556
H	-1.08337	6.526909	2.332389
C	-0.04335	5.573122	3.937462
H	0.682868	6.393301	4.026282
H	0.416964	4.788524	3.323319
H	-0.22697	5.145579	4.938526
C	-2.26414	4.850659	2.979318
H	-1.77553	4.128996	2.312142
H	-3.20447	5.180724	2.51314
H	-2.53341	4.32624	3.906785
C	-0.51314	8.952967	3.58514
O	-1.02462	9.305642	2.521252
N	0.782289	9.221758	3.869689
H	1.184306	8.847892	4.71922
C	1.631477	9.922316	2.947368
H	1.151456	10.86543	2.640534
C	1.967681	9.119808	1.683484
O	1.74817	7.915651	1.580859
N	2.520749	9.871931	0.713099
H	2.764147	10.83189	0.924393
C	2.993148	9.29387	-0.52394
H	2.158282	8.852382	-1.08161
C	4.069707	8.233019	-0.2777
O	4.892448	8.336246	0.616808
N	4.026244	7.200829	-1.15531
C	4.997321	6.136146	-1.09268
C	5.023173	5.434055	-2.4454
C	4.717744	5.128881	0.071618
O	4.029681	5.398494	-3.1767

C	3.336505	4.567041	0.058915
C	2.828152	3.346755	-0.34139
N	2.231312	5.323894	0.415325
C	1.123133	4.597034	0.214506
N	1.444742	3.389908	-0.24762
H	3.217783	7.077743	-1.76857
H	5.984289	6.574635	-0.88358
H	5.466225	4.326022	0.042009
H	4.885795	5.694686	1.000278
H	3.365306	2.465172	-0.67945
H	0.112759	4.939672	0.396171
H	2.215037	6.298538	0.750655
N	6.186179	4.829904	-2.76273
H	6.900752	4.690078	-2.03212
C	6.201238	3.874552	-3.84779
H	5.703503	4.298693	-4.72963
C	5.521911	2.587888	-3.38895
O	5.774101	2.102867	-2.27864
N	4.61124	2.024121	-4.19835
C	3.943125	0.808743	-3.75881
C	4.962053	-0.34835	-3.74788
C	2.74275	0.468749	-4.64092
O	5.638189	-0.58476	-4.73685
C	1.719241	-0.41344	-3.90524
C	0.813682	0.39001	-2.99521
O	-0.40735	0.467182	-3.26727
O	1.319366	0.994045	-1.98765
H	4.507109	2.347838	-5.15304
H	3.604625	0.997976	-2.73532
H	2.25303	1.398261	-4.97692
H	3.099257	-0.05632	-5.54091
H	2.243523	-1.18752	-3.32222
H	1.0765	-0.93364	-4.62257
N	5.014131	-1.02226	-2.57829
H	4.457029	-0.68066	-1.78046
C	5.92515	-2.11513	-2.313
H	5.603605	-2.55725	-1.35832
C	5.914047	-3.21449	-3.40157
H	6.564193	-2.94618	-4.24013
H	6.302849	-4.14528	-2.95882
C	4.550504	-3.49787	-4.0054
O	3.487943	-3.51991	-3.19689
O	4.39623	-3.7034	-5.19101
C	7.372623	-1.65015	-2.08077
O	8.259648	-2.47051	-1.84289
N	7.602547	-0.32454	-2.16631
H	6.82179	0.325996	-2.22603

C	8.955707	0.212767	-2.23777
H	9.580931	-0.60691	-2.62456
C	9.029819	1.40436	-3.17991
H	10.07648	1.699467	-3.33566
H	8.506829	2.269629	-2.75565
H	8.58355	1.137537	-4.14916
C	9.490642	0.543223	-0.82842
O	9.728252	1.691763	-0.4586
N	9.67891	-0.55546	-0.06968
H	9.299415	-1.42717	-0.44725
C	10.18187	-0.53176	1.298865
H	11.17634	-0.04952	1.28178
C	9.312009	0.281108	2.277237
H	9.468747	1.342402	2.049101
H	9.712862	0.080245	3.280602
C	7.83236	-0.02298	2.242748
C	6.943192	0.882171	1.641169
H	7.334548	1.783842	1.164925
C	5.563213	0.664229	1.685516
H	4.883425	1.404944	1.256929
C	5.05622	-0.47872	2.310351
H	3.97976	-0.62041	2.393936
C	5.931396	-1.41426	2.870526
H	5.53367	-2.3138	3.346279
C	7.308396	-1.18042	2.844119
H	7.992213	-1.88491	3.317522
C	10.40171	-1.97515	1.802622
O	10.26832	-2.25555	2.983173
N	10.75341	-2.88462	0.856246
H	10.86208	-2.57526	-0.10187
C	10.70923	-4.32052	1.093805
H	10.77164	-4.46548	2.179992
C	9.440288	-4.95355	0.509163
H	9.431841	-4.77049	-0.57948
H	9.503779	-6.04854	0.643332
C	8.118849	-4.43908	1.102394
H	8.126078	-3.33722	1.034646
C	6.932679	-4.91662	0.262451
H	7.065262	-4.6295	-0.79175
H	6.819744	-6.01362	0.311296
H	6.000122	-4.46456	0.627589
C	7.940984	-4.81859	2.575969
H	8.720059	-4.36683	3.207271
H	6.962908	-4.48274	2.95537
H	7.985664	-5.9144	2.703115
C	3.113285	-5.94548	5.901788
H	3.35628	-6.12387	6.961604

C	2.844602	-4.44112	5.676476
H	1.846302	-4.1593	6.045039
H	3.587763	-3.87235	6.25551
C	3.032211	-4.02896	4.209083
O	4.156861	-4.15702	3.715739
O	2.003691	-3.57606	3.584659
C	1.929186	-6.81237	5.517195
O	1.834656	-7.40359	4.441823
N	0.914503	-6.84667	6.430032
H	1.066289	-6.40489	7.328827
C	-0.24833	-7.70032	6.271942
H	0.050475	-8.7437	6.083508
C	-1.19268	-7.33519	5.12231
O	-2.06997	-8.09873	4.767606
N	-0.94719	-6.1444	4.509947
H	-0.21137	-5.54741	4.86847
C	-1.62722	-5.76536	3.294253
H	-2.71444	-5.87285	3.428742
C	-1.27632	-4.31881	2.929148
H	-1.90385	-4.01336	2.082395
H	-0.22085	-4.2932	2.637077
C	-1.50279	-3.34326	4.069338
O	-2.64392	-2.97793	4.380362
N	-0.38677	-2.95246	4.702803
H	0.566038	-3.14646	4.31364
H	-0.46245	-2.23844	5.418237
C	-1.27282	-6.65705	2.086653
O	-1.93179	-6.5429	1.049937
N	-0.22348	-7.48643	2.228726
H	0.29156	-7.49111	3.112079
C	0.104208	-8.51925	1.264688
H	-0.34175	-8.24197	0.300109
C	1.623792	-8.72365	1.121471
H	2.042369	-9.01641	2.095986
H	1.782375	-9.55978	0.421764
C	2.305752	-7.48669	0.621941
C	2.269779	-7.15363	-0.76224
H	1.819383	-7.86015	-1.46383
C	2.778082	-5.96374	-1.22179
H	2.741529	-5.69304	-2.27675
C	3.345089	-5.01127	-0.28634
O	3.711277	-3.84999	-0.65203
H	3.686232	-3.52669	-2.21388
C	3.443484	-5.39537	1.101468
H	3.910267	-4.7165	1.822655
C	2.922269	-6.59339	1.530188
H	2.955287	-6.85439	2.589578

C	0.219346	7.644421	-4.19536
C	0.184699	7.592455	-2.67422
C	-0.3876	6.364323	-4.81797
O	1.175994	7.26875	-2.01604
C	0.075296	5.135574	-4.11007
C	-0.61244	4.246509	-3.31555
N	1.405359	4.773324	-3.98892
C	1.488327	3.72789	-3.14601
N	0.282938	3.382933	-2.71421
H	-0.31306	8.530372	-4.57471
H	-0.13927	6.33319	-5.8918
H	-1.48548	6.3941	-4.75215
H	-1.68225	4.185989	-3.14335
H	2.432032	3.291411	-2.84457
H	2.239241	5.301598	-4.24362
N	-0.99534	7.881634	-2.09652
H	-1.84218	7.999602	-2.65026
C	-1.22306	7.803267	-0.67148
H	-0.6469	8.55755	-0.11339
H	-0.90546	6.821184	-0.28068
C	-2.72458	7.96419	-0.43919
O	-3.52037	7.72224	-1.34571
N	-3.09412	8.358602	0.79421
H	-2.37972	8.671987	1.464296
C	-4.49879	8.568365	1.100987
H	-4.98476	9.018755	0.220981
C	-5.23497	7.277854	1.474206
H	-5.05048	6.529298	0.688949
H	-4.79937	6.875389	2.403613
C	-6.74086	7.478473	1.637821
H	-7.19341	7.742552	0.667501
H	-6.94541	8.334459	2.30752
C	-7.46427	6.244312	2.183237
H	-8.55512	6.388862	2.159844
H	-7.24487	5.36869	1.556757
N	-7.0667	5.943932	3.559567
H	-7.26349	6.666684	4.243642
C	-6.22181	4.973113	3.946717
N	-5.96662	3.909785	3.19044
H	-6.58626	3.579707	2.460132
H	-5.29157	3.240057	3.597413
N	-5.60289	5.06103	5.132753
H	-5.58696	5.927816	5.652273
H	-5.01315	4.274121	5.42687
C	-5.62035	0.683707	-5.1848
H	-6.5476	0.100528	-5.25532
H	-5.57441	1.400703	-6.02159

N	-4.48165	-0.2157	-5.18349
H	-4.57794	-1.20191	-5.49622
C	-3.24545	0.136635	-4.83029
N	-2.98806	1.348733	-4.29424
H	-3.72612	1.872103	-3.83989
H	-2.05626	1.503834	-3.91573
N	-2.23107	-0.70359	-5.02438
H	-2.3688	-1.64017	-5.38806
H	-1.32752	-0.48994	-4.60177
O	-2.94427	2.451045	-1.56408
C	-2.51395	3.366164	-0.83666
O	-1.3	3.451103	-0.4282
C	-3.40566	4.512794	-0.41946
H	-4.35607	4.125912	-0.03017
H	-2.91945	5.136004	0.335824
O	-0.27392	1.034762	0.239155
O	-1.23085	-0.51168	2.22485
Fe	0.055782	2.219973	-1.00924
C	-2.80562	-1.52406	-1.54309
N	-2.1953	-0.19498	-1.31165
H	-1.56733	-0.06834	-0.48427
H	-1.57602	0.067427	-2.10653
H	-2.8663	0.588653	-1.24204
C	-3.58493	-2.02932	-0.31599
O	-3.35367	-1.47924	0.804559
O	-4.35487	-2.97187	-0.55351
C	-1.78978	-2.57885	-2.00424
C	-0.85022	-3.12299	-0.92531
C	0.405536	-2.30857	-0.56988
C	0.897229	-2.81448	0.778525
N	2.197644	-2.30688	1.258803
H	2.17739	-1.27059	1.345839
H	2.333206	-2.75587	2.227313
H	2.969717	-2.56701	0.624443
H	-8.98654	1.998475	-0.67363
H	2.574909	10.17462	3.454893
H	3.435622	10.09467	-1.1341
H	7.243551	3.639957	-4.10701
H	11.60281	-4.78439	0.64672
H	-1.95015	8.977284	5.136595
H	-4.55866	9.295311	1.925881
H	1.276548	7.743649	-4.47875
H	-3.63177	5.142886	-1.29344
H	3.98147	-6.24743	5.301143
H	-0.35375	-9.47584	1.572225
H	-0.82734	-7.69147	7.206109
H	-3.087	1.711195	6.82175

H	-6.52213	-0.93308	4.828415
H	0.097735	-7.20088	-3.09717
H	-3.51471	-9.187	-1.98198
H	-7.23366	-2.64844	-5.528
H	-0.87292	0.078353	1.509101
H	-1.97996	-0.98519	1.792102
H	0.162213	-2.52913	1.539181
H	0.96338	-3.91205	0.774109
H	0.206529	-1.22853	-0.49652
H	1.180195	-2.43852	-1.34343
H	-1.43681	-3.30375	-0.01062
H	-0.51588	-4.12289	-1.23636
H	-1.23232	-2.21614	-2.88038
H	-2.41219	-3.40757	-2.36873
H	-3.53426	-1.39161	-2.3546
H	-5.68625	1.250216	-4.24229
O	1.965908	0.512822	1.572617
H	1.126081	0.662588	1.037924
H	1.675328	0.26498	2.491054
O	0.260503	2.6388	2.406157
H	-0.208	2.17317	1.668661
H	1.183932	2.44022	2.173665
O	3.280517	-0.2418	-0.62653
H	2.519014	0.170317	-1.08437
H	3.21567	0.118294	0.278187
O	0.98225	-0.36907	3.897943
H	0.130016	-0.58288	3.467118
H	0.807968	0.435516	4.436712
O	0.401347	2.086059	4.993221
H	0.232802	2.366155	4.054498
H	1.180792	2.587302	5.269133
O	7.63701	3.548223	-0.7654
H	7.036241	2.859872	-1.1096
H	8.484562	3.078468	-0.61152

^{3}**Re**$_{C}$

C	-0.90281	-6.39026	-2.92536
H	-0.61872	-5.97504	-1.94633
C	-0.83179	-5.33791	-4.02891
H	-1.09158	-5.81971	-4.98398
H	-1.59416	-4.56927	-3.85605
C	0.517807	-4.64127	-4.1662
H	0.822347	-4.19195	-3.20683
H	1.318661	-5.34435	-4.43939
C	0.450807	-3.49999	-5.17418
O	-0.55785	-2.78885	-5.26511
N	1.540464	-3.30174	-5.93431
H	2.456768	-3.72878	-5.74626
H	1.51309	-2.52815	-6.59021
C	-2.27596	-7.04874	-2.83987
O	-2.92877	-7.3058	-3.85775
N	-2.70071	-7.37198	-1.60281
H	-2.23819	-6.99798	-0.76796
C	-3.88631	-8.17946	-1.35265
H	-3.7843	-8.56361	-0.32659
C	-5.22626	-7.44988	-1.49664
H	-5.38944	-7.22703	-2.56366
H	-6.02013	-8.16091	-1.20861
C	-5.33922	-6.17489	-0.66262
H	-4.59107	-5.43671	-0.99296
H	-5.10265	-6.38189	0.393887
C	-6.72691	-5.54232	-0.76122
H	-7.00833	-5.41462	-1.82484
H	-7.47657	-6.22079	-0.31332
N	-6.74123	-4.25633	-0.08262
H	-5.82402	-3.81394	0.103682
C	-7.77204	-3.41697	-0.04901
N	-8.9859	-3.77623	-0.55967
H	-9.20396	-4.76385	-0.6191
H	-9.75292	-3.14762	-0.31728
N	-7.61179	-2.1943	0.442099
H	-6.74734	-2.03119	0.993686
H	-8.42219	-1.56994	0.482126
C	-7.54845	-2.57284	-4.47392
H	-7.95316	-3.59652	-4.45358
C	-6.24437	-2.51283	-3.64775
H	-5.91591	-1.46806	-3.57175
H	-6.40772	-2.86176	-2.61776
C	-5.17769	-3.33439	-4.29253
N	-4.47232	-2.88802	-5.39656
C	-3.716	-3.9073	-5.7883

H	-2.99957	-3.89325	-6.60739
N	-3.91022	-4.98744	-4.996
H	-3.42167	-5.8933	-4.97947
C	-4.82708	-4.64043	-4.03464
H	-5.13854	-5.33716	-3.26785
C	-8.57269	-1.56534	-4.00258
O	-8.49386	-0.37074	-4.29532
N	-9.56093	-2.05324	-3.21024
H	-9.46828	-2.99694	-2.84759
C	-10.5618	-1.1888	-2.58926
H	-10.8057	-0.41499	-3.33177
C	-11.8173	-1.96679	-2.23077
H	-12.5652	-1.30122	-1.77788
H	-11.6076	-2.76365	-1.50198
H	-12.2503	-2.42141	-3.13315
C	-9.91137	-0.50289	-1.36992
O	-9.99932	-0.98736	-0.23427
N	-9.20741	0.591529	-1.69342
H	-9.12102	0.762887	-2.69656
C	-8.37433	1.408683	-0.82392
H	-8.23705	0.90282	0.142284
C	-7.01875	1.696914	-1.49455
H	-7.23516	1.889773	-2.56234
C	-6.35817	2.951094	-0.92116
H	-6.99466	3.842508	-1.05069
H	-6.14701	2.801109	0.148405
H	-5.398	3.135494	-1.42341
C	-6.08331	0.488273	-1.40443
H	-6.58587	-0.43702	-1.71374
H	-5.73425	0.35568	-0.37012
H	-5.203	0.625785	-2.05217
C	-2.37659	1.543296	5.924617
H	-1.42118	2.09032	5.960814
C	-2.08239	0.042142	5.935342
H	-1.34951	-0.16542	5.134014
C	-1.46425	-0.3909	7.260216
H	-1.27805	-1.47705	7.268697
H	-2.15041	-0.17086	8.092281
H	-0.50875	0.126782	7.435702
O	-3.29204	-0.64946	5.670867
H	-3.0741	-1.56155	5.35292
C	-3.14911	1.984666	4.70981
O	-4.09254	2.793982	4.805234
N	-2.82128	1.45515	3.519187
H	-2.09695	0.737333	3.428177
C	-3.70087	1.586149	2.381239
H	-3.85345	2.63657	2.100159

H	-3.23349	1.078892	1.52721
C	-5.07306	0.924865	2.587172
O	-6.02803	1.259381	1.878723
N	-5.11143	-0.01715	3.538708
H	-4.29542	-0.177	4.141547
C	-6.24544	-0.8779	3.800221
H	-7.08739	-0.52656	3.187205
C	-5.88856	-2.32477	3.457234
H	-5.07296	-2.68538	4.104417
H	-6.76749	-2.96692	3.632347
O	-5.5278	-2.46238	2.100704
H	-4.6614	-2.0455	1.874172
C	-0.91698	8.329948	4.623827
H	-0.19348	7.951672	5.361169
C	-1.83968	7.207977	4.148855
H	-2.67657	7.643776	3.579751
H	-2.28604	6.737499	5.042915
C	-1.17637	6.116193	3.291496
H	-0.97132	6.555503	2.298955
C	0.16221	5.633769	3.86041
H	0.92692	6.423693	3.83865
H	0.536159	4.793841	3.261927
H	0.050017	5.270851	4.896868
C	-2.14994	4.951763	3.112375
H	-1.72796	4.1751	2.461723
H	-3.10738	5.29009	2.688214
H	-2.37258	4.489496	4.085024
C	-0.19087	9.035616	3.495518
O	-0.7429	9.357276	2.442275
N	1.104304	9.344595	3.738563
H	1.544266	8.984898	4.575589
C	1.902963	10.06464	2.787072
H	1.342433	10.94247	2.428169
C	2.3104	9.231667	1.563661
O	2.076146	8.030259	1.47251
N	2.922652	9.958004	0.608718
H	3.172749	10.91752	0.813928
C	3.40798	9.362408	-0.61611
H	2.572123	8.953185	-1.19633
C	4.443466	8.267023	-0.34631
O	5.288678	8.373134	0.526354
N	4.342996	7.205933	-1.18439
C	5.278386	6.111375	-1.10298
C	5.252692	5.357557	-2.42827
C	4.997277	5.164213	0.105614
O	4.268795	5.385092	-3.17441
C	3.616285	4.601988	0.134472

C	3.118879	3.334627	-0.10484
N	2.503613	5.377261	0.420155
C	1.408641	4.601731	0.335752
N	1.738065	3.349519	0.021215
H	3.511457	7.086727	-1.76692
H	6.284319	6.525539	-0.93695
H	5.740035	4.354941	0.112018
H	5.175404	5.773254	1.005105
H	3.669334	2.429905	-0.35362
H	0.393734	4.952304	0.489314
H	2.480775	6.380817	0.650106
N	6.359491	4.642917	-2.71016
H	7.057793	4.466597	-1.97165
C	6.325598	3.683869	-3.79101
H	5.844438	4.130917	-4.6706
C	5.595803	2.424489	-3.33459
O	5.838439	1.916089	-2.23332
N	4.658055	1.906338	-4.14527
C	3.959695	0.70061	-3.72684
C	4.944115	-0.48703	-3.75018
C	2.747431	0.40989	-4.6099
O	5.608974	-0.71851	-4.74799
C	1.718455	-0.48511	-3.896
C	0.823223	0.310239	-2.9701
O	-0.38446	0.454532	-3.26764
O	1.332462	0.846466	-1.92483
H	4.567365	2.242848	-5.09703
H	3.632999	0.876467	-2.69655
H	2.268346	1.357289	-4.90936
H	3.089667	-0.08797	-5.53069
H	2.237422	-1.27573	-3.33134
H	1.070505	-0.98065	-4.62573
N	4.975793	-1.19235	-2.59911
H	4.44008	-0.84647	-1.78773
C	5.850878	-2.31935	-2.3578
H	5.511945	-2.77138	-1.41353
C	5.806649	-3.39322	-3.46921
H	6.45687	-3.12123	-4.30676
H	6.177802	-4.34204	-3.05004
C	4.433225	-3.63394	-4.07059
O	3.372466	-3.64394	-3.25946
O	4.269346	-3.8159	-5.25882
C	7.310788	-1.90377	-2.1077
O	8.170769	-2.75342	-1.87455
N	7.578813	-0.58365	-2.16665
H	6.817043	0.088566	-2.22303
C	8.944845	-0.07773	-2.20391

H	9.558841	-0.90546	-2.59167
C	9.064614	1.127395	-3.12412
H	10.12066	1.399405	-3.25657
H	8.55516	1.997251	-2.6931
H	8.629087	0.888037	-4.10537
C	9.45986	0.218608	-0.77979
O	9.734616	1.353046	-0.39267
N	9.591743	-0.89279	-0.02803
H	9.191944	-1.74875	-0.41898
C	10.09281	-0.89914	1.341112
H	11.09692	-0.43704	1.329528
C	9.243404	-0.07774	2.329798
H	9.418343	0.981829	2.106279
H	9.650316	-0.29008	3.328716
C	7.756958	-0.35133	2.315257
C	6.871033	0.595095	1.775021
H	7.269344	1.504258	1.317283
C	5.487553	0.407247	1.858823
H	4.810572	1.181813	1.489747
C	4.972425	-0.7479	2.454428
H	3.895907	-0.87173	2.566947
C	5.843514	-1.72317	2.94983
H	5.439837	-2.63091	3.403948
C	7.223513	-1.51817	2.8902
H	7.90391	-2.25274	3.320426
C	10.2861	-2.35311	1.823593
O	10.15225	-2.64843	3.000383
N	10.61762	-3.25433	0.861982
H	10.72855	-2.93181	-0.09147
C	10.54548	-4.69281	1.075397
H	10.59627	-4.85671	2.159485
C	9.269852	-5.29131	0.469497
H	9.275016	-5.0909	-0.61614
H	9.310404	-6.38932	0.586377
C	7.953359	-4.76095	1.059802
H	7.983824	-3.6583	1.013945
C	6.76643	-5.19742	0.198838
H	6.912493	-4.88458	-0.84623
H	6.636186	-6.29346	0.217588
H	5.838115	-4.7409	0.569344
C	7.753175	-5.16504	2.523903
H	8.536556	-4.74412	3.171062
H	6.779702	-4.81398	2.901338
H	7.771789	-6.26382	2.629647
C	2.868979	-6.13129	5.867453
H	3.091939	-6.32139	6.929578
C	2.656396	-4.61839	5.642779

H	1.668424	-4.3007	6.00954
H	3.417829	-4.07769	6.225202
C	2.863794	-4.21119	4.176023
O	3.976069	-4.40833	3.677783
O	1.86346	-3.68949	3.559782
C	1.659853	-6.95224	5.461936
O	1.54725	-7.50841	4.369561
N	0.644133	-6.98147	6.373729
H	0.809164	-6.57217	7.285465
C	-0.54622	-7.79085	6.189209
H	-0.28261	-8.83889	5.975829
C	-1.46997	-7.36482	5.043839
O	-2.36276	-8.09381	4.65632
N	-1.18871	-6.16174	4.472484
H	-0.44198	-5.59522	4.856766
C	-1.84399	-5.72917	3.261063
H	-2.93612	-5.78623	3.386093
C	-1.42063	-4.29548	2.923106
H	-2.02344	-3.95033	2.073741
H	-0.36153	-4.31598	2.643179
C	-1.61197	-3.32416	4.073948
O	-2.73706	-2.90991	4.382704
N	-0.48305	-2.99303	4.718345
H	0.460746	-3.21722	4.3205
H	-0.53097	-2.28645	5.443728
C	-1.52453	-6.6152	2.039104
O	-2.1893	-6.46928	1.010032
N	-0.49489	-7.47237	2.159397
H	0.024887	-7.50598	3.03964
C	-0.2152	-8.51096	1.185991
H	-0.6444	-8.2048	0.222514
C	1.293085	-8.7868	1.043331
H	1.696918	-9.09993	2.017799
H	1.412544	-9.62843	0.342673
C	2.031222	-7.58159	0.546317
C	2.029362	-7.25552	-0.84004
H	1.565877	-7.95004	-1.54521
C	2.585044	-6.08597	-1.29743
H	2.573612	-5.82005	-2.3543
C	3.167387	-5.14741	-0.35702
O	3.569709	-3.99714	-0.71861
H	3.567716	-3.66859	-2.27564
C	3.236669	-5.53062	1.03259
H	3.719269	-4.86897	1.75929
C	2.667417	-6.70691	1.459457
H	2.68021	-6.96424	2.520273
C	0.5926	7.588208	-4.13587

C	0.513077	7.575993	-2.61646
C	-0.03347	6.309368	-4.74008
O	1.489776	7.283248	-1.92317
C	0.357581	5.08373	-3.98385
C	-0.40068	4.217432	-3.22995
N	1.66797	4.679098	-3.79515
C	1.674007	3.625648	-2.96107
N	0.434846	3.317715	-2.59582
H	0.094869	8.47702	-4.55367
H	0.253787	6.235221	-5.80206
H	-1.13145	6.376669	-4.71929
H	-1.47908	4.186801	-3.11052
H	2.582576	3.150078	-2.61177
H	2.538106	5.177255	-4.00196
N	-0.68708	7.85658	-2.07867
H	-1.5207	7.957102	-2.65566
C	-0.95166	7.79436	-0.65917
H	-0.36165	8.529022	-0.09061
H	-0.68042	6.80232	-0.25722
C	-2.45184	8.003835	-0.46401
O	-3.23405	7.769076	-1.38441
N	-2.83527	8.42519	0.75618
H	-2.12284	8.732441	1.431172
C	-4.23883	8.673027	1.037017
H	-4.70445	9.101852	0.135643
C	-5.00296	7.409265	1.444029
H	-4.8257	6.634351	0.683498
H	-4.58256	7.026229	2.388639
C	-6.50618	7.640907	1.588235
H	-6.94245	7.903089	0.609967
H	-6.70196	8.507777	2.246439
C	-7.25973	6.425896	2.136367
H	-8.34697	6.594757	2.106384
H	-7.0561	5.542529	1.515411
N	-6.87497	6.122955	3.515913
H	-7.04632	6.857899	4.193881
C	-6.06649	5.125278	3.912452
N	-5.85162	4.045992	3.16567
H	-6.48491	3.73417	2.439203
H	-5.19758	3.357306	3.575641
N	-5.44539	5.200053	5.097904
H	-5.38765	6.073098	5.603957
H	-4.87846	4.397186	5.39396
C	-5.57638	0.939369	-5.16805
H	-6.5291	0.398059	-5.23453
H	-5.50349	1.657368	-6.00203
N	-4.47881	-0.01014	-5.17809

H	-4.61875	-0.98663	-5.50214
C	-3.22586	0.282316	-4.82826
N	-2.91272	1.473994	-4.27629
H	-3.6261	2.031194	-3.82298
H	-1.97334	1.584556	-3.90123
N	-2.25208	-0.59984	-5.04186
H	-2.44084	-1.52732	-5.40641
H	-1.33704	-0.43696	-4.6213
O	-2.81976	2.586887	-1.52127
C	-2.35932	3.454639	-0.75329
O	-1.15178	3.46746	-0.32013
C	-3.21203	4.617926	-0.30064
H	-4.14567	4.241435	0.137663
H	-2.68368	5.234368	0.431784
O	-0.31528	0.982292	0.266779
O	-1.25909	-0.58377	2.26754
Fe	0.123018	2.121834	-0.93365
C	-2.90271	-1.42668	-1.57026
N	-2.24502	-0.12095	-1.33527
H	-1.63608	-0.04122	-0.49153
H	-1.60019	0.118172	-2.1174
H	-2.88337	0.688977	-1.26591
C	-3.68248	-1.91086	-0.3348
O	-3.41787	-1.3711	0.783827
O	-4.48143	-2.83017	-0.56379
C	-1.9242	-2.5138	-2.03786
C	-1.00419	-3.10144	-0.96352
C	0.284526	-2.34293	-0.60183
C	0.766947	-2.89157	0.734134
N	2.084305	-2.43367	1.217136
H	2.094718	-1.39485	1.329351
H	2.212125	-2.89818	2.176956
H	2.847474	-2.69992	0.575077
H	-8.89765	2.359916	-0.62106
H	2.814142	10.42806	3.286129
H	3.897015	10.14777	-1.21043
H	7.35509	3.402637	-4.05551
H	11.43383	-5.16639	0.627952
H	-1.51107	9.103194	5.14145
H	-4.29565	9.429007	1.835728
H	1.659568	7.650798	-4.39085
H	-3.47601	5.244905	-1.16546
H	3.73327	-6.46059	5.275569
H	-0.71944	-9.44703	1.484418
H	-1.12966	-7.78462	7.120667
H	-2.9845	1.82009	6.797423
H	-6.53215	-0.80342	4.86283

H	-0.18374	-7.20286	-3.13312
H	-3.8762	-9.03831	-2.04091
H	-7.3177	-2.33319	-5.52209
H	-0.88219	-0.02161	1.546535
H	-2.03324	-1.01489	1.836342
H	0.045849	-2.59534	1.50345
H	0.792946	-3.99059	0.709285
H	0.127098	-1.25824	-0.50611
H	1.048163	-2.4865	-1.38399
H	-1.59498	-3.26777	-0.04876
H	-0.71084	-4.11095	-1.28398
H	-1.35449	-2.16608	-2.91198
H	-2.57662	-3.31671	-2.40794
H	-3.63241	-1.26826	-2.37567
H	-5.61154	1.503698	-4.22252
O	1.997018	0.338504	1.599381
H	1.181453	0.595316	1.102926
H	1.708866	0.175318	2.536326
O	0.162584	2.574556	2.44815
H	-0.29194	2.06346	1.737922
H	1.01985	2.741813	2.022382
O	3.317216	-0.40541	-0.60301
H	2.556926	0.029942	-1.04132
H	3.293557	-0.04669	0.302947
O	1.000033	-0.45406	3.941967
H	0.142419	-0.66581	3.525134
H	0.843066	0.357725	4.476346
O	0.543257	2.020752	5.017169
H	0.352606	2.287829	4.07949
H	1.378358	2.454222	5.240214
O	7.721423	3.292968	-0.68669
H	7.095197	2.630711	-1.03694
H	8.547577	2.787525	-0.52976

^{5}**Re**$_{C}$

C	-0.37229	-6.40968	-2.9972
H	-0.09912	-5.98208	-2.02067
C	-0.38611	-5.34859	-4.09488
H	-0.61318	-5.84228	-5.05231
H	-1.20276	-4.63922	-3.91604
C	0.908967	-4.55293	-4.23047
H	1.179022	-4.08439	-3.26968
H	1.758392	-5.1954	-4.50704
C	0.754914	-3.41789	-5.23659
O	-0.30238	-2.78338	-5.32075
N	1.821875	-3.14686	-6.00878
H	2.772629	-3.48097	-5.81047
H	1.735476	-2.3729	-6.65902
C	-1.69509	-7.16103	-2.8917
O	-2.34064	-7.46805	-3.89994
N	-2.08012	-7.50958	-1.64797
H	-1.6357	-7.10051	-0.82008
C	-3.19493	-8.40761	-1.38118
H	-3.04696	-8.78455	-0.35802
C	-4.59047	-7.78711	-1.50333
H	-4.78158	-7.56658	-2.56604
H	-5.32113	-8.56357	-1.21701
C	-4.80067	-6.53581	-0.6522
H	-4.1156	-5.73485	-0.97385
H	-4.54513	-6.73665	0.401021
C	-6.23499	-6.01591	-0.73965
H	-6.52777	-5.89626	-1.80133
H	-6.9252	-6.76064	-0.3017
N	-6.35324	-4.7478	-0.03931
H	-5.47894	-4.22063	0.128797
C	-7.46025	-4.01354	0.035329
N	-8.64292	-4.47712	-0.46156
H	-8.76633	-5.47848	-0.55009
H	-9.4631	-3.92542	-0.20532
N	-7.41037	-2.79507	0.558242
H	-6.54737	-2.54946	1.07653
H	-8.27285	-2.24738	0.614976
C	-7.33525	-3.0886	-4.38335
H	-7.65691	-4.14161	-4.38456
C	-6.02486	-2.94806	-3.57782
H	-5.77146	-1.88357	-3.48594
H	-6.14079	-3.3256	-2.55206
C	-4.91804	-3.68382	-4.25893
N	-4.32403	-3.20914	-5.41505
C	-3.49661	-4.16463	-5.82407

H	-2.83923	-4.11347	-6.69036
N	-3.53657	-5.23083	-4.99117
H	-2.97444	-6.09304	-4.99059
C	-4.42672	-4.94085	-3.98647
H	-4.6238	-5.63624	-3.18219
C	-8.43279	-2.18072	-3.87525
O	-8.45649	-0.97619	-4.13369
N	-9.37461	-2.7729	-3.09666
H	-9.20155	-3.71431	-2.75918
C	-10.4521	-2.01808	-2.46265
H	-10.7621	-1.25226	-3.18863
C	-11.6324	-2.91653	-2.13086
H	-12.4395	-2.3343	-1.66494
H	-11.3515	-3.70903	-1.42156
H	-12.0193	-3.38562	-3.0467
C	-9.87547	-1.30437	-1.22331
O	-9.91468	-1.82666	-0.10138
N	-9.28807	-0.1343	-1.51174
H	-9.20819	0.071029	-2.50896
C	-8.54218	0.729344	-0.60954
H	-8.36849	0.205592	0.34118
C	-7.21115	1.161982	-1.24724
H	-7.43469	1.421686	-2.29972
C	-6.65151	2.413894	-0.56847
H	-7.34957	3.26469	-0.64721
H	-6.44341	2.197263	0.489176
H	-5.6995	2.695932	-1.03626
C	-6.19144	0.019477	-1.23889
H	-6.61684	-0.90406	-1.65149
H	-5.86163	-0.18642	-0.2109
H	-5.30567	0.284613	-1.83537
C	-2.32051	1.318806	5.92673
H	-1.41834	1.949667	5.935091
C	-1.88734	-0.1481	5.950968
H	-1.18075	-0.30989	5.116155
C	-1.16507	-0.48759	7.250314
H	-0.88863	-1.55417	7.277133
H	-1.82143	-0.30095	8.113988
H	-0.24894	0.112792	7.356954
O	-3.04066	-0.95192	5.766639
H	-2.75907	-1.85144	5.465824
C	-3.16579	1.674424	4.731302
O	-4.15766	2.422294	4.845876
N	-2.84603	1.139262	3.540833
H	-2.06446	0.484562	3.432707
C	-3.78457	1.153196	2.441099
H	-4.05352	2.173572	2.139045

H	-3.30938	0.66806	1.578131
C	-5.07039	0.360585	2.723176
O	-6.08752	0.580843	2.05856
N	-4.97042	-0.56591	3.686184
H	-4.12085	-0.63214	4.258892
C	-5.98611	-1.55923	3.964901
H	-6.90463	-1.26295	3.439093
C	-5.50891	-2.93343	3.491373
H	-4.62242	-3.25033	4.063682
H	-6.30717	-3.67469	3.662019
O	-5.2212	-2.93299	2.109482
H	-4.39613	-2.44573	1.871566
C	-1.9962	7.911288	4.634958
H	-1.30996	7.578138	5.427335
C	-2.67809	6.716788	3.964442
H	-3.50296	7.085639	3.336516
H	-3.14063	6.092882	4.749424
C	-1.76028	5.835706	3.101218
H	-1.44823	6.440024	2.230631
C	-0.48661	5.410097	3.838083
H	0.163492	6.270136	4.053446
H	0.085167	4.70211	3.226033
H	-0.72645	4.899143	4.787063
C	-2.54776	4.627393	2.590517
H	-1.95007	4.005597	1.910327
H	-3.45802	4.946057	2.059677
H	-2.86952	3.995906	3.430542
C	-1.26662	8.79185	3.639481
O	-1.78563	9.18322	2.592947
N	-0.00777	9.152216	3.98098
H	0.413572	8.740316	4.803228
C	0.795745	9.983326	3.128536
H	0.222619	10.87767	2.837113
C	1.269696	9.277199	1.850722
O	1.152766	8.066127	1.681693
N	1.813036	10.11674	0.948997
H	1.968151	11.08069	1.218092
C	2.396918	9.641188	-0.28542
H	1.629617	9.170341	-0.9116
C	3.538451	8.652671	-0.02689
O	4.320697	8.793502	0.898422
N	3.599314	7.642654	-0.92872
C	4.632281	6.639305	-0.84815
C	4.712504	5.927478	-2.19613
C	4.393913	5.629525	0.319192
O	3.757885	5.919164	-2.97882
C	3.078161	4.932528	0.241578

C	2.739557	3.646654	-0.12693
N	1.87221	5.573298	0.4852
C	0.87061	4.714101	0.25055
N	1.357864	3.529693	-0.11941
H	2.814383	7.468908	-1.56079
H	5.591074	7.135522	-0.63382
H	5.212212	4.896541	0.339165
H	4.460865	6.221326	1.244407
H	3.396161	2.82459	-0.40321
H	-0.18497	4.950533	0.338444
H	1.730342	6.551266	0.783355
N	5.871423	5.292212	-2.45352
H	6.559779	5.14289	-1.69931
C	5.945446	4.375424	-3.56786
H	5.445667	4.813125	-4.44166
C	5.319718	3.035774	-3.18637
O	5.527878	2.528526	-2.07705
N	4.514499	2.442042	-4.08311
C	3.916411	1.154052	-3.76124
C	5.011836	0.069094	-3.78332
C	2.784691	0.805336	-4.72702
O	5.745355	-0.05236	-4.75206
C	1.82041	-0.24483	-4.15149
C	0.831225	0.332957	-3.16076
O	-0.39224	0.264511	-3.40948
O	1.272527	0.884334	-2.09205
H	4.466866	2.795324	-5.03227
H	3.519655	1.243564	-2.74478
H	2.222895	1.719058	-4.98191
H	3.225303	0.420398	-5.66036
H	2.388509	-1.05318	-3.66142
H	1.237038	-0.709	-4.95291
N	5.059984	-0.6767	-2.6581
H	4.45395	-0.42749	-1.86073
C	6.034411	-1.71777	-2.41275
H	5.723946	-2.2122	-1.47975
C	6.105802	-2.77397	-3.53826
H	6.721641	-2.4197	-4.37108
H	6.575575	-3.68466	-3.13412
C	4.761128	-3.14511	-4.14012
O	3.715463	-3.30393	-3.32384
O	4.607029	-3.29986	-5.3331
C	7.441954	-1.16381	-2.13174
O	8.390572	-1.92534	-1.94559
N	7.565788	0.179872	-2.10914
H	6.73583	0.767226	-2.13419
C	8.870653	0.827173	-2.14384

H	9.558984	0.084736	-2.57644
C	8.846916	2.074944	-3.01231
H	9.866294	2.459701	-3.15256
H	8.262549	2.868586	-2.53208
H	8.414583	1.833383	-3.99444
C	9.378302	1.116881	-0.71656
O	9.521313	2.257785	-0.27962
N	9.657609	-0.00476	-0.02309
H	9.348746	-0.88104	-0.45016
C	10.16973	-0.01937	1.342238
H	11.12272	0.540641	1.347745
C	9.247192	0.666595	2.368688
H	9.311587	1.747419	2.193822
H	9.676431	0.450029	3.356906
C	7.798621	0.238875	2.331154
C	6.82647	1.08532	1.773905
H	7.131192	2.038534	1.335887
C	5.471436	0.74291	1.817357
H	4.721647	1.437094	1.430166
C	5.074509	-0.46581	2.396336
H	4.017118	-0.71022	2.484122
C	6.035323	-1.34026	2.912731
H	5.722702	-2.28989	3.352057
C	7.385159	-0.98369	2.887775
H	8.133738	-1.6434	3.326311
C	10.50729	-1.46704	1.76146
O	10.4125	-1.82176	2.925527
N	10.91319	-2.29295	0.761575
H	10.99016	-1.92265	-0.17778
C	10.97512	-3.73973	0.91514
H	11.055	-3.94248	1.990856
C	9.750982	-4.42709	0.297088
H	9.721057	-4.17991	-0.77843
H	9.894522	-5.52062	0.364282
C	8.400306	-4.04691	0.925098
H	8.327551	-2.9452	0.926771
C	7.244941	-4.55624	0.061081
H	7.347636	-4.19427	-0.97325
H	7.213712	-5.65949	0.04004
H	6.284165	-4.19839	0.456558
C	8.26247	-4.52845	2.37285
H	9.012522	-4.06191	3.02819
H	7.266282	-4.28719	2.775985
H	8.386177	-5.62392	2.431507
C	3.485435	-5.99961	5.751522
H	3.737238	-6.18619	6.807694
C	3.15062	-4.50532	5.551801

H	2.145036	-4.27279	5.934249
H	3.873506	-3.91341	6.133667
C	3.309549	-4.06373	4.089301
O	4.4289	-4.1663	3.579871
O	2.264846	-3.61273	3.48948
C	2.336995	-6.90422	5.34807
O	2.24714	-7.44344	4.245108
N	1.343006	-7.02933	6.275127
H	1.491849	-6.62656	7.192532
C	0.2102	-7.91676	6.088824
H	0.544165	-8.93696	5.841831
C	-0.76392	-7.52767	4.972332
O	-1.6155	-8.30525	4.586649
N	-0.57455	-6.29664	4.422426
H	0.144079	-5.69139	4.801153
C	-1.28532	-5.88368	3.235687
H	-2.36597	-6.03612	3.379272
C	-0.98967	-4.40994	2.934765
H	-1.64817	-4.08419	2.119416
H	0.056234	-4.33632	2.618507
C	-1.21171	-3.4961	4.126149
O	-2.35185	-3.18585	4.494456
N	-0.0883	-3.09296	4.738672
H	0.853451	-3.23372	4.302812
H	-0.16621	-2.42168	5.494509
C	-0.92064	-6.70745	1.9828
O	-1.60666	-6.57735	0.965702
N	0.164297	-7.49863	2.067361
H	0.696673	-7.52146	2.940222
C	0.495793	-8.49347	1.064616
H	0.032648	-8.19389	0.115103
C	2.015897	-8.67028	0.892974
H	2.454059	-8.97947	1.853805
H	2.175247	-9.48739	0.171482
C	2.671627	-7.41192	0.412236
C	2.627177	-7.05614	-0.96623
H	2.194948	-7.76203	-1.6797
C	3.103541	-5.84524	-1.4054
H	3.058839	-5.5558	-2.45517
C	3.642345	-4.89395	-0.45241
O	3.962683	-3.71178	-0.79214
H	3.919973	-3.33796	-2.34281
C	3.759839	-5.30348	0.926417
H	4.211355	-4.62795	1.659999
C	3.27084	-6.5216	1.335101
H	3.31641	-6.80093	2.389427
C	-0.01377	7.746412	-4.02624

C	-0.16849	7.702506	-2.51353
C	-0.52047	6.4553	-4.70573
O	0.782081	7.427021	-1.7769
C	-0.02573	5.215687	-4.03763
C	-0.71164	4.173549	-3.45449
N	1.310081	4.939801	-3.806
C	1.387548	3.788144	-3.11075
N	0.179196	3.294645	-2.87141
H	-0.54413	8.614054	-4.44859
H	-0.22538	6.478556	-5.76844
H	-1.6204	6.424858	-4.69085
H	-1.78513	4.01395	-3.43571
H	2.329121	3.378291	-2.76394
H	2.123705	5.555105	-3.88994
N	-1.39757	7.956827	-2.02739
H	-2.20543	8.052842	-2.64085
C	-1.71923	7.924069	-0.61944
H	-1.27507	8.768109	-0.06738
H	-1.3152	7.011605	-0.14953
C	-3.24313	7.92692	-0.48893
O	-3.94878	7.671328	-1.46212
N	-3.7264	8.198713	0.739189
H	-3.08313	8.524023	1.472107
C	-5.1591	8.217512	0.979201
H	-5.65775	8.580202	0.067296
C	-5.72348	6.842558	1.354376
H	-5.43022	6.116287	0.580172
H	-5.24977	6.509555	2.292096
C	-7.2437	6.835539	1.509889
H	-7.7277	6.990973	0.531495
H	-7.56896	7.682315	2.142684
C	-7.78431	5.535498	2.112692
H	-8.88489	5.522222	2.087508
H	-7.43815	4.674114	1.525241
N	-7.34215	5.359842	3.497348
H	-7.58736	6.105317	4.140114
C	-6.4367	4.468063	3.933812
N	-6.13119	3.372936	3.249065
H	-6.72781	2.965613	2.538653
H	-5.38752	2.787183	3.665988
N	-5.80793	4.671666	5.102444
H	-5.81852	5.578197	5.549225
H	-5.14682	3.955793	5.421396
C	-5.62981	0.600433	-4.98538
H	-6.56755	0.03124	-5.02916
H	-5.62926	1.369034	-5.77623
N	-4.5098	-0.3129	-5.12478

H	-4.64713	-1.2799	-5.46415
C	-3.23763	0.014883	-4.89234
N	-2.91087	1.217166	-4.37353
H	-3.57075	1.698642	-3.76759
H	-1.93762	1.320917	-4.09109
N	-2.26688	-0.84101	-5.20704
H	-2.49443	-1.77011	-5.54304
H	-1.32929	-0.70143	-4.83262
O	-3.34614	2.196642	-1.70369
C	-2.80267	3.193192	-1.18497
O	-1.61525	3.194692	-0.70488
C	-3.52024	4.521201	-1.11096
H	-4.5484	4.46017	-1.4836
H	-3.52002	4.879819	-0.07317
O	-0.28755	0.897026	0.332257
O	-1.10133	-0.73918	2.291434
Fe	0.052834	2.115864	-0.92504
C	-2.72427	-1.62607	-1.58698
N	-2.12864	-0.2879	-1.35371
H	-1.56346	-0.17932	-0.4851
H	-1.47305	-0.05503	-2.13402
H	-2.80747	0.505053	-1.34446
C	-3.47573	-2.16292	-0.35374
O	-3.20132	-1.66063	0.780311
O	-4.25815	-3.09281	-0.59901
C	-1.68832	-2.65911	-2.05912
C	-0.72406	-3.18618	-0.99068
C	0.522169	-2.35083	-0.64617
C	1.055161	-2.87337	0.681233
N	2.341084	-2.32829	1.165587
H	2.290023	-1.30079	1.280803
H	2.508461	-2.79282	2.125325
H	3.118797	-2.5321	0.516699
H	-9.15365	1.621132	-0.38328
H	1.682057	10.31846	3.688521
H	2.81058	10.50431	-0.82669
H	7.001117	4.196865	-3.81852
H	11.8969	-4.11083	0.439665
H	-2.75443	8.547479	5.122677
H	-5.36101	8.942736	1.783403
H	1.058712	7.884916	-4.22319
H	-2.97453	5.271968	-1.69931
H	4.364353	-6.2516	5.143444
H	0.057163	-9.46674	1.34732
H	-0.35417	-7.978	7.029953
H	-2.92595	1.551353	6.814223
H	-6.19282	-1.58357	5.047641

H	0.39497	-7.17206	-3.22209
H	-3.1276	-9.26155	-2.07232
H	-7.14266	-2.8058	-5.42859
H	-0.7819	-0.13281	1.567116
H	-1.8351	-1.23012	1.855958
H	0.319925	-2.63858	1.457915
H	1.16615	-3.96703	0.64632
H	0.297014	-1.27896	-0.54493
H	1.282145	-2.44354	-1.43966
H	-1.29445	-3.38229	-0.06892
H	-0.37429	-4.17771	-1.30997
H	-1.14713	-2.28016	-2.93883
H	-2.29453	-3.50179	-2.41916
H	-3.46245	-1.50341	-2.39119
H	-5.61534	1.104582	-4.00598
O	2.062497	0.54741	1.576475
H	1.196105	0.614946	1.091435
H	1.827971	0.225883	2.49227
O	0.213438	2.530768	2.501081
H	-0.2664	2.008526	1.815758
H	1.131528	2.41685	2.199245
O	3.280607	-0.22555	-0.67176
H	2.50436	0.123866	-1.15579
H	3.205895	0.175116	0.216768
O	1.204867	-0.49716	3.851397
H	0.339248	-0.75716	3.476287
H	1.020469	0.272834	4.436554
O	0.592787	1.882148	5.057403
H	0.358629	2.196479	4.145366
H	1.361702	2.406259	5.320365
O	7.306593	3.984026	-0.46446
H	6.738533	3.285945	-0.84289
H	8.180011	3.55302	-0.34194

^{5}**TS1**$_{C,C3}$

C	3.768861	-6.51274	-2.05125
H	3.973604	-6.15072	-1.03283
C	2.907821	-5.53384	-2.84307
H	2.620085	-6.01309	-3.78214
H	1.967554	-5.34118	-2.30085
C	3.571696	-4.21867	-3.21145
H	3.770626	-3.57857	-2.33801
H	4.554829	-4.40125	-3.68335
C	2.732317	-3.45001	-4.22605
O	1.781845	-3.97999	-4.8068
N	3.107812	-2.17764	-4.448
H	3.931606	-1.7698	-4.00886
H	2.719254	-1.67433	-5.2363
C	3.090824	-7.87895	-2.00435
O	2.645029	-8.41468	-3.02875
N	3.007242	-8.44856	-0.79323
H	3.275822	-7.88988	0.021465
C	2.310254	-9.70188	-0.55531
H	2.526883	-9.99018	0.484424
C	0.794321	-9.62218	-0.77781
H	0.605205	-9.55166	-1.86073
H	0.352442	-10.5761	-0.44084
C	0.116867	-8.44724	-0.07267
H	0.514533	-7.49258	-0.45209
H	0.340005	-8.45069	1.007942
C	-1.3956	-8.46438	-0.2858
H	-1.61677	-8.63532	-1.35409
H	-1.8406	-9.31168	0.271618
N	-1.98856	-7.20207	0.119134
H	-1.44824	-6.55411	0.713085
C	-3.17336	-6.75821	-0.28315
N	-4.03867	-7.58423	-0.91778
H	-3.95166	-8.57784	-0.74578
H	-4.98249	-7.20624	-1.08618
N	-3.50464	-5.47693	-0.11633
H	-2.82302	-4.79846	0.255384
H	-4.48518	-5.20089	-0.12876
C	-3.65532	-6.34795	-4.23819
H	-3.53109	-7.38198	-3.88381
C	-2.52347	-5.47999	-3.66975
H	-2.68935	-4.43939	-3.98734
H	-2.55018	-5.48873	-2.57218
C	-1.21075	-6.00383	-4.15251
N	-0.83441	-5.91996	-5.48191
C	0.277519	-6.63311	-5.59578

H	0.865442	-6.75425	-6.5035
N	0.620295	-7.19147	-4.41327
H	1.467383	-7.73037	-4.16377
C	-0.3012	-6.78645	-3.47807
H	-0.22326	-7.04268	-2.42877
C	-5.04046	-5.83619	-3.91418
O	-5.3228	-4.64191	-3.89379
N	-5.97391	-6.79923	-3.67848
H	-5.67036	-7.76266	-3.61146
C	-7.30667	-6.46297	-3.23213
H	-7.74784	-5.75793	-3.95562
C	-8.17212	-7.7193	-3.13087
H	-9.19246	-7.46071	-2.81517
H	-7.75574	-8.41547	-2.38571
H	-8.22914	-8.22917	-4.10405
C	-7.21836	-5.76104	-1.86538
O	-6.37323	-6.09752	-1.03434
N	-8.14485	-4.81709	-1.64043
H	-8.74182	-4.55478	-2.4175
C	-8.15766	-3.95901	-0.4676
H	-7.38543	-4.33027	0.220783
C	-7.8745	-2.49302	-0.8312
H	-8.64263	-2.17222	-1.56212
C	-8.03391	-1.6344	0.427424
H	-9.03362	-1.76865	0.876077
H	-7.27732	-1.94655	1.164716
H	-7.90253	-0.56508	0.199366
C	-6.48969	-2.33433	-1.46655
H	-6.36058	-2.9989	-2.33344
H	-5.7098	-2.58803	-0.73285
H	-6.31256	-1.30518	-1.80891
C	-2.84453	-0.8841	5.511635
H	-2.67998	0.199151	5.6253
C	-1.48654	-1.55318	5.306346
H	-0.98342	-1.04201	4.474565
C	-0.59841	-1.42095	6.536919
H	0.37661	-1.89775	6.351737
H	-1.06167	-1.91986	7.402418
H	-0.42538	-0.36178	6.781016
O	-1.68837	-2.91533	4.95621
H	-0.89622	-3.20091	4.442179
C	-3.81743	-1.11434	4.382406
O	-5.03247	-1.28614	4.619204
N	-3.34779	-1.16467	3.129373
H	-2.35571	-1.01738	2.901906
C	-4.12725	-1.72596	2.050749
H	-5.0964	-1.22538	1.938829

H	-3.569	-1.58353	1.115596
C	-4.38921	-3.23483	2.167394
O	-5.26197	-3.74227	1.459648
N	-3.5904	-3.89913	3.016149
H	-2.93172	-3.37749	3.604557
C	-3.61074	-5.33425	3.221892
H	-4.10828	-5.79541	2.359696
C	-2.19726	-5.89643	3.414124
H	-1.76085	-5.47296	4.335667
H	-2.28931	-6.98492	3.574545
O	-1.34315	-5.67194	2.315362
H	-0.76405	-4.89979	2.481548
C	-5.8946	5.549971	5.196884
H	-5.03072	5.733232	5.852902
C	-5.9419	4.084093	4.761525
H	-6.91876	3.879574	4.29615
H	-5.89924	3.462396	5.67308
C	-4.83125	3.635083	3.795911
H	-5.0562	4.070467	2.806684
C	-3.44054	4.136236	4.197594
H	-3.38223	5.233439	4.167675
H	-2.69295	3.754108	3.489942
H	-3.17083	3.792707	5.211338
C	-4.86401	2.110516	3.662077
H	-4.08295	1.749044	2.982183
H	-5.83857	1.757708	3.290635
H	-4.69564	1.631221	4.640172
C	-5.891	6.529949	4.040292
O	-6.65928	6.438072	3.081466
N	-5.01555	7.556461	4.140448
H	-4.35511	7.567606	4.906421
C	-4.95455	8.596632	3.14999
H	-5.94411	9.068465	3.035274
C	-4.47999	8.117489	1.773498
O	-3.88719	7.056467	1.603283
N	-4.76682	8.987068	0.782269
H	-5.12203	9.900761	1.037097
C	-4.20452	8.817503	-0.53849
H	-4.53729	7.868658	-0.97365
C	-2.6737	8.861891	-0.48805
O	-2.0827	9.576165	0.306409
N	-2.05717	8.061111	-1.39065
C	-0.61572	7.984758	-1.41914
C	-0.16534	7.439112	-2.76891
C	-0.04217	7.122868	-0.2496
O	-0.88458	6.721869	-3.46879
C	-0.56751	5.729162	-0.23449

C	-0.04214	4.523244	-0.65835
N	-1.85539	5.435495	0.17957
C	-2.07218	4.12166	-0.01011
N	-0.99519	3.528084	-0.51396
H	-2.59121	7.353901	-1.89774
H	-0.21705	9.000465	-1.28072
H	1.054102	7.126293	-0.30255
H	-0.32142	7.649136	0.675463
H	0.952001	4.323385	-1.05003
H	-3.00746	3.626464	0.215099
H	-2.55135	6.091578	0.553048
N	1.099991	7.772436	-3.10898
H	1.722116	8.173406	-2.39412
C	1.759144	7.092419	-4.19815
H	1.090587	7.046548	-5.06829
C	2.184898	5.693225	-3.76121
O	2.698956	5.497718	-2.65183
N	1.950948	4.681465	-4.60675
C	2.244743	3.315851	-4.20084
C	3.766159	3.139725	-4.0286
C	1.673709	2.328727	-5.22019
O	4.543849	3.485142	-4.90054
C	1.469155	0.916073	-4.65855
C	0.301475	0.792554	-3.69514
O	-0.59462	-0.05815	-3.91251
O	0.287028	1.576367	-2.69039
H	1.599673	4.869872	-5.53813
H	1.757671	3.162814	-3.23272
H	0.706114	2.714248	-5.58559
H	2.357247	2.276674	-6.08186
H	2.383724	0.588642	-4.14043
H	1.297622	0.216923	-5.4876
N	4.110556	2.587391	-2.83681
H	3.403209	2.512705	-2.09281
C	5.473806	2.350518	-2.41987
H	5.414834	1.828063	-1.45159
C	6.255524	1.456532	-3.41547
H	6.506839	2.042374	-4.30623
H	7.176063	1.106336	-2.92878
C	5.387664	0.30313	-3.87799
O	5.282305	-0.76657	-3.06862
O	4.750398	0.313892	-4.90523
C	6.272025	3.640333	-2.1501
O	7.46064	3.582829	-1.83337
N	5.602669	4.800455	-2.29034
H	4.59662	4.795049	-2.44737
C	6.30022	6.075979	-2.40149

H	7.323902	5.829294	-2.72443
C	5.639907	6.979894	-3.43202
H	6.264659	7.865347	-3.61281
H	4.665135	7.334226	-3.07578
H	5.511304	6.429348	-4.37517
C	6.430394	6.74736	-1.02062
O	5.868277	7.801816	-0.73228
N	7.233648	6.067242	-0.17588
H	7.517494	5.131085	-0.47545
C	7.518366	6.503582	1.184744
H	7.886374	7.543646	1.126505
C	6.285295	6.523955	2.108173
H	5.667726	7.377842	1.802965
H	6.661931	6.715167	3.12269
C	5.438516	5.272782	2.100051
C	4.211735	5.260606	1.414438
H	3.904291	6.145158	0.850552
C	3.363432	4.153099	1.482851
H	2.391128	4.181357	0.989019
C	3.743585	3.027737	2.222843
H	3.044215	2.193955	2.322801
C	4.985449	3.004933	2.86804
H	5.303857	2.12463	3.431189
C	5.822814	4.122855	2.809421
H	6.774931	4.122833	3.340855
C	8.665242	5.65883	1.776858
O	8.713816	5.407848	2.970791
N	9.597139	5.236298	0.883502
H	9.479209	5.490799	-0.0897
C	10.55602	4.183485	1.185811
H	10.71105	4.1902	2.272488
C	10.0627	2.816764	0.693947
H	9.895836	2.880209	-0.39575
H	10.86872	2.076364	0.846377
C	8.774345	2.314512	1.364428
H	8.017318	3.114295	1.286261
C	8.202308	1.113152	0.611956
H	8.003515	1.367541	-0.44038
H	8.896786	0.254884	0.632922
H	7.262432	0.788292	1.079059
C	8.968476	1.997014	2.850737
H	9.225213	2.89845	3.425997
H	8.050881	1.564359	3.277348
H	9.776704	1.256459	2.985426
C	7.082511	-2.39986	5.564852
H	7.600206	-2.32368	6.536225
C	6.084953	-1.23236	5.430788

H	5.210763	-1.38773	6.083727
H	6.579632	-0.30358	5.742257
C	5.613776	-1.04536	3.989144
O	6.097464	-0.11123	3.330119
O	4.769671	-1.89098	3.510884
C	6.454102	-3.7787	5.484775
O	6.773286	-4.62314	4.647519
N	5.494689	-4.02935	6.415156
H	5.293909	-3.31461	7.103454
C	4.895375	-5.33542	6.586001
H	5.672175	-6.1125	6.666726
C	3.967227	-5.8068	5.468007
O	3.512421	-6.93503	5.491371
N	3.719924	-4.92683	4.454862
H	4.166788	-4.01195	4.432435
C	3.037025	-5.40216	3.276384
H	2.236807	-6.08767	3.591129
C	2.444746	-4.25941	2.445107
H	1.89913	-4.70256	1.603376
H	3.271713	-3.64992	2.054855
C	1.490878	-3.3725	3.217681
O	0.273337	-3.63981	3.27375
N	2.032027	-2.30724	3.807649
H	3.041486	-2.11538	3.727671
H	1.441019	-1.52598	4.101105
C	3.931871	-6.21827	2.332496
O	3.403493	-6.79159	1.379561
N	5.257449	-6.20115	2.560479
H	5.625127	-5.7215	3.386423
C	6.19352	-6.9066	1.70614
H	5.670282	-7.14457	0.769791
C	7.47145	-6.08916	1.434615
H	8.020241	-5.96134	2.37957
H	8.106242	-6.68589	0.759546
C	7.20386	-4.74026	0.832415
C	6.9014	-4.5942	-0.55244
H	6.893042	-5.48116	-1.19112
C	6.620317	-3.36121	-1.09162
H	6.381528	-3.23612	-2.14897
C	6.595227	-2.18502	-0.2447
O	6.22921	-1.05085	-0.68387
H	5.771775	-0.754	-2.19558
C	6.942992	-2.34306	1.149315
H	6.957675	-1.45084	1.777915
C	7.222521	-3.5884	1.658135
H	7.438232	-3.71734	2.719781
C	-5.49105	5.602088	-3.82221

C	-5.20592	5.514312	-2.32972
C	-5.07804	4.30554	-4.55848
O	-4.18755	6.009649	-1.84397
C	-3.79748	3.742977	-4.03959
C	-3.55369	2.569226	-3.36223
N	-2.59075	4.424942	-4.02906
C	-1.68628	3.680728	-3.36798
N	-2.2379	2.548431	-2.94818
H	-6.55386	5.816266	-4.01405
H	-5.02275	4.51018	-5.64074
H	-5.85126	3.533792	-4.42738
H	-4.2303	1.746208	-3.14904
H	-0.67435	4.017373	-3.17384
H	-2.399	5.400234	-4.25852
N	-6.10415	4.844336	-1.5837
H	-6.88369	4.346434	-2.00821
C	-5.98487	4.673854	-0.15025
H	-5.81169	5.634097	0.357001
H	-5.12687	4.025331	0.103255
C	-7.25944	3.980372	0.326218
O	-7.82648	3.171143	-0.40943
N	-7.69299	4.303111	1.557037
H	-7.25234	5.069824	2.082241
C	-8.94489	3.772334	2.066421
H	-9.71381	3.845932	1.27937
C	-8.85455	2.319097	2.538235
H	-8.52801	1.700135	1.686316
H	-8.07538	2.245263	3.313418
C	-10.1892	1.813818	3.084499
H	-10.9568	1.838968	2.292564
H	-10.5491	2.495835	3.876137
C	-10.1494	0.392055	3.648128
H	-11.1534	0.088075	3.980794
H	-9.86107	-0.33282	2.872207
N	-9.24597	0.236126	4.786979
H	-9.56332	0.609368	5.674172
C	-7.98179	-0.21862	4.739215
N	-7.42998	-0.63629	3.603822
H	-7.89782	-0.54723	2.714203
H	-6.46656	-1.01213	3.666547
N	-7.24136	-0.26512	5.854149
H	-7.65746	-0.11384	6.762801
H	-6.29219	-0.65955	5.76995
C	-3.51141	-3.80296	-6.86966
H	-3.59064	-4.75594	-7.40844
H	-3.7785	-3.00298	-7.57987
N	-2.14449	-3.65935	-6.40499

H	-1.57988	-4.53933	-6.26096
C	-1.69818	-2.56404	-5.79601
N	-2.49162	-1.48121	-5.63941
H	-3.47092	-1.51148	-5.88083
H	-2.10831	-0.65632	-5.18274
N	-0.45601	-2.49574	-5.31772
H	0.243645	-3.25202	-5.35357
H	-0.20401	-1.63528	-4.82837
O	-3.68728	-0.22808	-1.57862
C	-3.85015	0.631329	-0.70829
O	-2.91647	1.446909	-0.32833
C	-5.1923	0.855321	-0.04716
H	-5.75433	-0.08152	-0.01365
H	-5.07802	1.273025	0.960567
O	-0.49324	0.23983	-0.53024
O	-0.94822	-1.1283	1.707675
Fe	-1.27959	1.488329	-1.4199
C	-0.40884	-2.78996	-1.77738
N	-1.60114	-1.9805	-2.18805
H	-2.06955	-1.5894	-1.35647
H	-1.33437	-1.17524	-2.79287
H	-2.30867	-2.54143	-2.67147
C	-0.85086	-3.73203	-0.63258
O	-1.72105	-3.24507	0.164864
O	-0.32519	-4.84954	-0.58046
C	0.727459	-1.83864	-1.35215
C	1.702829	-2.55668	-0.41925
C	3.047752	-1.87088	-0.19746
C	2.947409	-0.60251	0.636941
N	4.26319	-0.27278	1.249816
H	4.296879	0.669046	1.659509
H	4.502504	-0.93929	2.036847
H	5.039444	-0.32747	0.562597
H	-9.13723	-4.04503	0.033447
H	-4.25033	9.369661	3.493268
H	-4.55899	9.63896	-1.17811
H	2.662852	7.654446	-4.47787
H	11.51823	4.434237	0.712372
H	-6.79286	5.78232	5.794677
H	-9.25615	4.421835	2.898886
H	-4.90725	6.455763	-4.19384
H	-5.7755	1.574379	-0.64546
H	7.854677	-2.33326	4.786503
H	6.487138	-7.86661	2.16478
H	4.324192	-5.35028	7.524443
H	-3.33048	-1.26503	6.42189
H	-4.20606	-5.58879	4.118875

H	4.738912	-6.65479	-2.5613
H	2.727159	-10.4811	-1.21222
H	-3.56018	-6.38264	-5.33643
H	-0.92165	-0.54714	0.897161
H	-1.10407	-2.02694	1.325633
H	2.629639	0.252192	0.031727
H	2.238147	-0.72984	1.464653
H	3.527683	-1.63717	-1.16203
H	3.717415	-2.58283	0.313555
H	1.214975	-2.69974	0.557285
H	1.897207	-3.57387	-0.79394
H	0.216603	-0.83274	-0.90421
H	1.268634	-1.52173	-2.26147
H	-0.12364	-3.40691	-2.64123
H	-4.23911	-3.81618	-6.03777
O	0.765792	1.799699	1.285444
H	0.3515	1.16124	0.642487
H	0.967541	1.254171	2.08151
O	-1.80487	2.021118	2.154571
H	-2.32225	1.720534	1.381209
H	-0.90068	2.178476	1.794293
O	2.262554	2.148227	-0.90086
H	1.526099	1.823223	-1.4538
H	1.876101	2.241248	-0.00187
O	0.755368	0.139699	3.416327
H	0.145456	-0.39082	2.843969
H	0.157223	0.689021	3.979879
O	-1.15853	1.620627	4.662207
H	-1.56574	1.754585	3.764358
H	-0.91309	2.508971	4.953673
O	3.091684	7.811461	-1.13735
H	3.096775	6.904204	-1.49912
H	4.034279	8.016024	-0.96275

[5]**IM1**$_{C,C3}$

C	3.545973	-5.13504	-3.14943
H	3.680854	-4.57731	-2.21102
C	2.659221	-4.3816	-4.13969
H	2.547319	-5.00665	-5.03549
H	1.648367	-4.26748	-3.71905
C	3.179655	-3.01353	-4.56167
H	3.287941	-2.32203	-3.70987
H	4.191341	-3.09355	-4.9971
C	2.272289	-2.36078	-5.60128
O	1.269786	-2.93327	-6.04038
N	2.625466	-1.1283	-6.00815
H	3.434528	-0.60736	-5.64495
H	2.086044	-0.71916	-6.76337
C	3.007699	-6.53803	-2.87944
O	2.705812	-7.29167	-3.80921
N	2.912642	-6.90693	-1.58613
H	3.013258	-6.22606	-0.82905
C	2.574855	-8.25946	-1.16558
H	2.870525	-8.3305	-0.10805
C	1.104027	-8.66179	-1.31205
H	0.882548	-8.78668	-2.38236
H	0.999169	-9.66101	-0.8536
C	0.109452	-7.68954	-0.67839
H	0.059864	-6.75574	-1.2605
H	0.441265	-7.40272	0.333791
C	-1.29657	-8.28366	-0.59656
H	-1.61615	-8.61815	-1.60105
H	-1.28392	-9.18125	0.050292
N	-2.23008	-7.30186	-0.07395
H	-1.88716	-6.33477	0.060134
C	-3.55051	-7.44518	-0.00702
N	-4.16153	-8.57148	-0.46612
H	-3.61898	-9.41761	-0.57837
H	-5.1519	-8.66191	-0.24013
N	-4.29762	-6.45916	0.475319
H	-3.80115	-5.71854	0.999696
H	-5.31366	-6.58114	0.501781
C	-4.3642	-6.87495	-4.64052
H	-4.1732	-7.87171	-5.06952
C	-3.30046	-6.56264	-3.56702
H	-3.56946	-5.63668	-3.03432
H	-3.28872	-7.35746	-2.80795
C	-1.93494	-6.41137	-4.14504
N	-1.58117	-5.3075	-4.89662
C	-0.31966	-5.49282	-5.26376

H	0.292313	-4.79011	-5.82732
N	0.152293	-6.671	-4.79109
H	1.133189	-6.97996	-4.77677
C	-0.86111	-7.2702	-4.07973
H	-0.73567	-8.2366	-3.60523
C	-5.75819	-6.79874	-4.05722
O	-6.51036	-5.8457	-4.25438
N	-6.09785	-7.83187	-3.23905
H	-5.39295	-8.52189	-3.00525
C	-7.34575	-7.85766	-2.48248
H	-8.12612	-7.46147	-3.1492
C	-7.69852	-9.27543	-2.06346
H	-8.64421	-9.2887	-1.50437
H	-6.92574	-9.70232	-1.40644
H	-7.80407	-9.917	-2.94997
C	-7.21395	-6.90292	-1.27672
O	-6.84976	-7.31379	-0.16657
N	-7.47981	-5.62338	-1.5763
H	-7.60278	-5.43034	-2.57232
C	-7.40414	-4.47333	-0.68906
H	-6.97309	-4.78656	0.272411
C	-6.58447	-3.32934	-1.31141
H	-6.88274	-3.26847	-2.37541
C	-6.92853	-1.98773	-0.65836
H	-8.0053	-1.76315	-0.74173
H	-6.6319	-1.99652	0.400574
H	-6.36664	-1.18165	-1.1471
C	-5.08198	-3.61674	-1.24503
H	-4.8369	-4.58605	-1.70047
H	-4.74386	-3.63119	-0.19945
H	-4.5138	-2.83528	-1.76864
C	-2.59555	0.048077	5.900006
H	-2.18874	1.068908	5.822815
C	-1.44187	-0.95966	5.974051
H	-0.73596	-0.73433	5.154256
C	-0.69351	-0.84938	7.297347
H	0.102868	-1.60999	7.352928
H	-1.3763	-1.03399	8.140622
H	-0.23145	0.142019	7.399055
O	-1.98404	-2.25901	5.796087
H	-1.24868	-2.86087	5.52369
C	-3.53264	-0.2034	4.740727
O	-4.76662	-0.17475	4.895556
N	-2.98337	-0.47075	3.538248
H	-1.96755	-0.61134	3.437894
C	-3.7636	-1.02868	2.450542
H	-4.59772	-0.37603	2.168928

H	-3.11264	-1.11677	1.570221
C	-4.31066	-2.43715	2.719245
O	-5.24727	-2.86818	2.036326
N	-3.69775	-3.12407	3.690495
H	-2.99001	-2.67068	4.280769
C	-3.93513	-4.52889	3.9544
H	-4.86495	-4.81757	3.444703
C	-2.75817	-5.36391	3.445986
H	-1.8454	-5.11831	4.011626
H	-2.97969	-6.43263	3.603301
O	-2.54374	-5.17554	2.064314
H	-2.15969	-4.29523	1.83077
C	-6.95219	5.096319	4.338319
H	-6.1733	5.473564	5.017463
C	-6.57099	3.73126	3.767321
H	-7.42987	3.336602	3.201842
H	-6.40431	3.03462	4.607223
C	-5.33149	3.712022	2.856577
H	-5.57718	4.291907	1.94986
C	-4.11127	4.365566	3.513224
H	-4.2303	5.456603	3.591205
H	-3.19694	4.174657	2.934869
H	-3.93501	3.956197	4.522316
C	-5.03834	2.270155	2.443627
H	-4.21721	2.197655	1.716285
H	-5.92446	1.797147	1.991624
H	-4.77418	1.673049	3.327006
C	-7.24682	6.114372	3.253756
O	-7.89864	5.838196	2.24674
N	-6.78809	7.368254	3.481416
H	-6.14982	7.517086	4.253084
C	-6.91927	8.400386	2.491992
H	-7.90536	8.304563	2.012267
C	-5.85157	8.29735	1.394135
O	-5.11923	7.315742	1.297994
N	-5.82822	9.338293	0.541169
H	-6.36716	10.16501	0.768385
C	-4.86212	9.411974	-0.53624
H	-5.04387	8.613883	-1.26671
C	-3.43247	9.324701	0.00705
O	-3.09021	9.924471	1.012833
N	-2.59946	8.549735	-0.73054
C	-1.2225	8.386365	-0.33138
C	-0.41255	7.906019	-1.53344
C	-1.06452	7.432375	0.891932
O	-0.93845	7.353936	-2.50195
C	-1.55504	6.047507	0.643594

C	-0.87927	4.854802	0.48371
N	-2.89225	5.735736	0.471369
C	-2.99314	4.419977	0.21019
N	-1.79072	3.848938	0.213141
H	-2.97315	7.938744	-1.45766
H	-0.84401	9.367497	-0.00618
H	-0.00712	7.394286	1.189268
H	-1.62453	7.904504	1.713343
H	0.188138	4.663585	0.552473
H	-3.92468	3.902349	0.005975
H	-3.68513	6.387588	0.555394
N	0.917561	8.113446	-1.44291
H	1.335165	8.369633	-0.54134
C	1.819769	7.533599	-2.40862
H	1.414774	7.673712	-3.41974
C	2.063921	6.050946	-2.12555
O	2.265314	5.632279	-0.97978
N	2.027113	5.229006	-3.18828
C	2.321448	3.815153	-3.04722
C	3.841079	3.605509	-3.02936
C	1.698099	3.024513	-4.20595
O	4.562649	4.199695	-3.8139
C	1.571009	1.519532	-3.93574
C	0.410821	1.177968	-3.02643
O	-0.46185	0.36614	-3.41012
O	0.370857	1.734988	-1.87693
H	2.015084	5.62902	-4.12027
H	1.889598	3.498829	-2.09352
H	0.702342	3.44412	-4.42144
H	2.319995	3.175039	-5.10263
H	2.489801	1.135711	-3.46771
H	1.450456	0.969974	-4.87518
N	4.295931	2.699912	-2.12566
H	3.675303	2.354104	-1.38072
C	5.702046	2.33135	-2.11012
H	5.848831	1.69981	-1.22077
C	6.131968	1.561697	-3.37465
H	6.326892	2.263234	-4.19146
H	7.069465	1.023828	-3.16469
C	5.086715	0.582282	-3.8635
O	4.630277	-0.3348	-3.00885
O	4.6448	0.616209	-4.99508
C	6.60475	3.569289	-1.92716
O	7.67034	3.678285	-2.50988
N	6.1447	4.480796	-1.0244
H	5.187487	4.354157	-0.71172
C	6.655864	5.849001	-0.99742

H	6.694814	6.233139	-2.03035
C	5.734204	6.734745	-0.15981
H	6.127569	7.759781	-0.16207
H	5.700278	6.378161	0.883327
H	4.709357	6.74151	-0.5606
C	8.100611	5.992741	-0.48933
O	8.687669	7.044432	-0.66807
N	8.624871	4.921578	0.159697
H	8.046487	4.098852	0.286696
C	9.997741	4.878443	0.621641
H	10.39641	5.893602	0.470142
C	10.09283	4.523764	2.118418
H	9.583287	5.315181	2.688454
H	11.15708	4.536836	2.385614
C	9.491393	3.176495	2.441452
C	8.121079	3.048792	2.726226
H	7.49832	3.947512	2.764796
C	7.549763	1.799319	2.983221
H	6.48775	1.691808	3.20669
C	8.353692	0.655894	2.969182
H	7.910179	-0.31608	3.190448
C	9.718202	0.766254	2.689939
H	10.35165	-0.12394	2.682392
C	10.2813	2.015069	2.420735
H	11.34129	2.104071	2.179925
C	10.88708	3.92778	-0.2113
O	11.9599	3.542501	0.237242
N	10.40252	3.58654	-1.42555
H	9.485113	3.918603	-1.71466
C	11.06946	2.64666	-2.30622
H	12.07477	2.478363	-1.89866
C	10.29215	1.332279	-2.44536
H	9.311833	1.563973	-2.89568
H	10.82595	0.687316	-3.16665
C	10.07672	0.559701	-1.13447
H	9.568433	1.236405	-0.42413
C	9.154681	-0.64186	-1.36337
H	8.187103	-0.33242	-1.79017
H	9.6102	-1.36838	-2.05795
H	8.946947	-1.16807	-0.41746
C	11.3996	0.123165	-0.49738
H	12.01029	0.988629	-0.2034
H	11.21966	-0.47821	0.407161
H	11.98655	-0.49513	-1.19838
C	6.848617	-2.51266	5.260167
H	7.48659	-2.45955	6.158667
C	5.829549	-1.35827	5.30245

H	5.123023	-1.50114	6.138395
H	6.361695	-0.41508	5.482485
C	5.024589	-1.20813	4.008101
O	5.127235	-0.165	3.352059
O	4.285932	-2.2062	3.654643
C	6.242027	-3.904	5.196618
O	6.482687	-4.70319	4.291011
N	5.398297	-4.21828	6.213268
H	5.24469	-3.53609	6.944915
C	4.768575	-5.51578	6.329943
H	5.517214	-6.31676	6.225061
C	3.680952	-5.83029	5.30019
O	3.135578	-6.91858	5.305318
N	3.415796	-4.85426	4.386202
H	3.913511	-3.95868	4.381531
C	2.552244	-5.12739	3.267534
H	1.947223	-6.00466	3.548856
C	1.62041	-3.94757	2.930975
H	0.823475	-4.29329	2.262819
H	2.202067	-3.17716	2.416119
C	0.970564	-3.32845	4.155608
O	-0.13452	-3.70902	4.557489
N	1.675119	-2.34986	4.745322
H	2.612291	-2.10534	4.403098
H	1.306246	-1.90028	5.575095
C	3.327942	-5.56249	2.009714
O	2.730903	-5.66802	0.932757
N	4.634769	-5.83985	2.174816
H	5.072375	-5.63883	3.07658
C	5.456803	-6.46473	1.156462
H	4.89131	-6.4646	0.216152
C	6.820275	-5.7685	0.9624
H	7.371692	-5.78595	1.914057
H	7.3889	-6.36131	0.228329
C	6.668211	-4.35694	0.486807
C	6.423935	-4.07022	-0.88627
H	6.444011	-4.88857	-1.61039
C	6.140271	-2.79306	-1.3042
H	5.920315	-2.56569	-2.34843
C	6.061646	-1.71748	-0.33529
O	5.613591	-0.56983	-0.63944
H	5.071273	-0.33048	-2.10523
C	6.418926	-2.0052	1.03347
H	6.402954	-1.17063	1.735134
C	6.685956	-3.29369	1.424899
H	6.880332	-3.53816	2.469877
C	-5.08431	6.166689	-4.2383

C	-5.32497	6.087781	-2.73729
C	-4.45315	4.877759	-4.80889
O	-4.56673	6.642085	-1.93719
C	-3.32578	4.355579	-3.98015
C	-3.13132	3.115508	-3.40948
N	-2.24944	5.114617	-3.55615
C	-1.47351	4.347229	-2.76426
N	-1.98056	3.127823	-2.64924
H	-6.02327	6.37903	-4.7729
H	-4.12543	5.077759	-5.84336
H	-5.20911	4.080788	-4.87546
H	-3.75273	2.22967	-3.508
H	-0.59275	4.731375	-2.2603
H	-2.09506	6.120975	-3.62539
N	-6.38614	5.362349	-2.33784
H	-6.94867	4.821488	-2.99466
C	-6.75125	5.160754	-0.95634
H	-7.13817	6.078912	-0.48506
H	-5.87642	4.868453	-0.35469
C	-7.80335	4.048119	-0.90888
O	-8.07303	3.409786	-1.92243
N	-8.36267	3.834387	0.299039
H	-8.1676	4.493057	1.062822
C	-9.40038	2.83738	0.487275
H	-9.8311	2.610802	-0.49888
C	-8.89017	1.548187	1.139763
H	-8.09076	1.117763	0.515006
H	-8.42706	1.801444	2.107946
C	-9.99555	0.513946	1.349143
H	-10.3823	0.168684	0.375679
H	-10.8557	0.977775	1.866279
C	-9.5443	-0.71515	2.141788
H	-10.3482	-1.46584	2.185708
H	-8.69127	-1.19173	1.641487
N	-9.15299	-0.37441	3.510913
H	-9.87695	0.019888	4.101631
C	-7.89546	-0.32146	3.978265
N	-6.88113	-0.90186	3.356988
H	-6.96276	-1.64239	2.666119
H	-5.95305	-0.75203	3.790206
N	-7.63108	0.338508	5.120347
H	-8.29475	0.987204	5.520356
H	-6.67325	0.320068	5.474538
C	-4.58364	-2.94698	-4.83517
H	-4.95173	-3.97757	-4.9044
H	-5.01252	-2.36393	-5.66634
N	-3.13092	-2.94028	-4.88282

H	-2.59777	-3.83963	-4.99711
C	-2.41505	-1.82694	-4.76519
N	-3.00469	-0.66183	-4.40825
H	-3.79366	-0.68409	-3.76025
H	-2.36306	0.109018	-4.23631
N	-1.10047	-1.81832	-5.00345
H	-0.58407	-2.55879	-5.47502
H	-0.57523	-0.99706	-4.70045
O	-4.15356	-0.29086	-1.83623
C	-4.27806	0.789748	-1.21316
O	-3.35476	1.311138	-0.50464
C	-5.58201	1.556561	-1.28165
H	-5.95304	1.744729	-0.26486
H	-5.40125	2.539701	-1.73967
O	-0.61266	0.601853	0.415061
O	-0.50892	-1.05294	2.417758
Fe	-1.48297	2.028863	-0.82622
C	-1.01301	-2.59164	-1.50844
N	-1.59957	-1.22505	-1.39628
H	-1.30363	-0.73435	-0.51682
H	-1.2331	-0.62117	-2.16661
H	-2.64343	-1.14347	-1.46339
C	-1.43667	-3.50701	-0.33882
O	-1.58648	-2.96901	0.802752
O	-1.53649	-4.70583	-0.63079
C	0.480063	-2.47915	-1.60669
C	1.433101	-3.31026	-0.81988
C	2.69342	-2.55581	-0.36717
C	2.314895	-1.34595	0.481865
N	3.393416	-0.8373	1.361231
H	3.015806	-0.11814	2.011966
H	3.782268	-1.54288	2.042118
H	4.175975	-0.42317	0.835595
H	-8.43187	-4.12277	-0.48545
H	-6.87064	9.388115	2.974996
H	-4.9771	10.38469	-1.03528
H	2.788888	8.052546	-2.34824
H	11.18885	3.104844	-3.30196
H	-7.87298	5.003	4.940069
H	-10.1981	3.277605	1.108683
H	-4.41427	7.023254	-4.39715
H	-6.35086	1.037664	-1.86436
H	7.517887	-2.40433	4.396125
H	5.643959	-7.51814	1.427144
H	4.324746	-5.61749	7.329633
H	-3.20976	-0.00488	6.809505
H	-4.06464	-4.68067	5.038495

H	4.552265	-5.27124	-3.58424
H	3.192339	-8.96846	-1.73865
H	-4.30238	-6.14705	-5.45954
H	-0.61059	-0.37394	1.679201
H	-0.78632	-1.88164	1.960637
H	2.020133	-0.50403	-0.1538
H	1.465881	-1.58505	1.133459
H	3.285231	-2.23044	-1.23674
H	3.322752	-3.25221	0.207389
H	0.942805	-3.74187	0.062405
H	1.741842	-4.1906	-1.40992
H	0.285132	0.466588	0.080898
H	0.869023	-1.85739	-2.42122
H	-1.41848	-3.04141	-2.42998
H	-4.96589	-2.53545	-3.88823
O	0.695337	2.479734	1.870764
H	0.094072	1.901111	1.335609
H	1.166093	1.791098	2.403305
O	-1.28669	2.385251	3.750881
H	-1.92675	1.798628	3.320573
H	-0.67025	2.660867	3.030293
O	2.453326	1.79943	-0.18722
H	1.722024	1.795426	-0.84706
H	2.061501	2.301099	0.560128
O	1.703213	0.334005	3.182624
H	0.967288	-0.30972	3.047427
H	1.561074	0.684473	4.096061
O	0.67117	1.441295	5.377733
H	-0.10346	1.788504	4.865791
H	1.030212	2.206073	5.847648
O	2.32269	7.653921	0.86254
H	2.417845	6.804204	0.37782
H	3.125163	7.77116	1.386157

[5]**TS2**$_{C,C3}$

C	2.046169	-6.68768	-1.53975
H	1.868015	-6.31552	-0.52047
C	1.728633	-5.60979	-2.564
H	2.003102	-5.95619	-3.5719
H	0.648683	-5.41574	-2.57563
C	2.431293	-4.29694	-2.25543
H	2.089779	-3.96373	-1.26252
H	3.521476	-4.41546	-2.18563
C	2.072111	-3.2176	-3.26052
O	0.901398	-3.1189	-3.68105
N	3.048542	-2.3908	-3.62354
H	3.983841	-2.33656	-3.18409
H	2.88207	-1.62214	-4.26698
C	1.231451	-7.95275	-1.7506
O	0.83166	-8.3106	-2.87129
N	0.974907	-8.65012	-0.63428
H	1.241004	-8.22769	0.264819
C	0.16324	-9.85384	-0.61372
H	0.193969	-10.2349	0.417875
C	-1.29136	-9.63105	-1.05372
H	-1.30581	-9.49794	-2.14674
H	-1.85929	-10.5518	-0.83465
C	-1.96206	-8.42614	-0.39485
H	-1.39947	-7.50817	-0.63267
H	-1.93458	-8.51376	0.704961
C	-3.40533	-8.25861	-0.86466
H	-3.44178	-8.3384	-1.96638
H	-4.03029	-9.07936	-0.46456
N	-3.93487	-6.96525	-0.46066
H	-3.41866	-6.37821	0.220536
C	-5.03976	-6.40738	-0.95122
N	-5.80067	-7.05	-1.86403
H	-5.69913	-8.04877	-1.97962
H	-6.68553	-6.59786	-2.11586
N	-5.38923	-5.17233	-0.58468
H	-4.86489	-4.69392	0.150187
H	-6.29886	-4.81711	-0.87354
C	-4.82791	-5.45184	-5.13488
H	-4.89726	-6.54449	-5.02491
C	-3.7918	-4.88811	-4.14314
H	-3.80492	-3.79171	-4.22031
H	-4.10259	-5.12938	-3.11704
C	-2.42206	-5.42493	-4.40301
N	-1.63992	-4.98452	-5.45546
C	-0.54445	-5.73511	-5.44146

H	0.302911	-5.6329	-6.11665
N	-0.59292	-6.64888	-4.44898
H	0.139359	-7.3011	-4.10143
C	-1.76917	-6.45985	-3.76861
H	-2.02357	-7.05215	-2.89822
C	-6.17111	-4.78644	-4.93888
O	-6.29456	-3.56414	-4.8915
N	-7.24741	-5.60127	-4.77524
H	-7.11798	-6.60533	-4.76385
C	-8.52419	-5.04796	-4.36421
H	-8.80042	-4.25346	-5.07534
C	-9.60238	-6.1285	-4.33906
H	-10.5715	-5.69622	-4.05243
H	-9.35162	-6.91069	-3.60593
H	-9.71027	-6.58729	-5.33298
C	-8.35124	-4.43006	-2.96602
O	-7.82605	-5.08947	-2.06217
N	-8.82564	-3.18751	-2.7978
H	-9.12646	-2.69159	-3.62992
C	-8.69229	-2.42524	-1.56729
H	-8.35772	-3.12392	-0.78766
C	-7.70789	-1.24805	-1.67831
H	-8.08213	-0.57608	-2.47454
C	-7.72137	-0.47646	-0.35444
H	-8.74513	-0.14669	-0.1026
H	-7.32958	-1.12814	0.44354
H	-7.08723	0.419161	-0.41109
C	-6.29346	-1.70687	-2.04707
H	-6.2915	-2.29128	-2.97815
H	-5.86877	-2.31617	-1.2348
H	-5.62657	-0.84549	-2.2025
C	-3.64627	-0.20743	5.661771
H	-3.344	0.839097	5.822326
C	-2.38707	-1.0781	5.639262
H	-1.68667	-0.64246	4.907028
C	-1.6901	-1.09081	6.994485
H	-0.78685	-1.71888	6.949692
H	-2.35468	-1.50859	7.766472
H	-1.38735	-0.07464	7.291095
O	-2.76111	-2.38295	5.233691
H	-1.94676	-2.89284	4.981322
C	-4.47833	-0.28044	4.403257
O	-5.72494	-0.19943	4.455166
N	-3.84285	-0.44944	3.236758
H	-2.81816	-0.54529	3.198624
C	-4.51653	-0.89637	2.040665
H	-5.39424	-0.28028	1.805417

H	-3.81964	-0.79904	1.196865
C	-4.94975	-2.37217	2.069953
O	-5.68591	-2.79945	1.175718
N	-4.45786	-3.09836	3.082364
H	-3.82906	-2.67397	3.779421
C	-4.64529	-4.53055	3.216327
H	-5.54302	-4.81713	2.649778
C	-3.42752	-5.29532	2.698184
H	-2.52353	-5.01831	3.266139
H	-3.59564	-6.37679	2.837698
O	-3.23284	-5.05641	1.315712
H	-2.69783	-4.22952	1.154785
C	-6.34119	5.830365	4.631459
H	-5.57684	5.861625	5.422177
C	-6.36989	4.464754	3.944132
H	-7.27686	4.403099	3.325377
H	-6.46731	3.68047	4.714831
C	-5.15539	4.155575	3.054934
H	-5.15977	4.881415	2.225055
C	-3.82443	4.331681	3.790763
H	-3.65701	5.38021	4.075792
H	-2.99122	4.035259	3.139045
H	-3.78306	3.702832	4.69543
C	-5.30157	2.754882	2.459775
H	-4.4379	2.495075	1.835665
H	-6.21015	2.682114	1.844462
H	-5.37585	1.995254	3.251399
C	-6.1227	6.958575	3.64227
O	-6.724	7.03002	2.569338
N	-5.23179	7.905377	4.014114
H	-4.70273	7.777534	4.866891
C	-4.92286	9.017644	3.160264
H	-5.85549	9.491761	2.815907
C	-4.09111	8.634887	1.929143
O	-3.56612	7.532001	1.794568
N	-3.99487	9.632258	1.027207
H	-4.35753	10.54339	1.281407
C	-3.09086	9.546296	-0.09796
H	-3.4105	8.759213	-0.79105
C	-1.66035	9.280405	0.378118
O	-1.21368	9.787633	1.393616
N	-0.94818	8.452852	-0.42591
C	0.418516	8.129492	-0.1005
C	1.129708	7.659093	-1.36606
C	0.520034	7.083721	1.052789
O	0.507822	7.220302	-2.33529
C	-0.17631	5.793037	0.77941

C	0.307171	4.526037	0.513591
N	-1.55419	5.670173	0.72462
C	-1.85946	4.394968	0.42675
N	-0.75478	3.657177	0.302124
H	-1.412	7.921848	-1.1634
H	0.905372	9.041986	0.275287
H	1.577696	6.89206	1.281471
H	0.076619	7.572173	1.93416
H	1.342578	4.214672	0.40923
H	-2.87275	4.028419	0.293739
H	-2.24534	6.418413	0.885554
N	2.475065	7.731985	-1.33205
H	2.954092	7.898522	-0.43891
C	3.260292	7.068807	-2.3458
H	2.859444	7.302758	-3.34084
C	3.276493	5.557267	-2.11442
O	3.386801	5.102577	-0.95919
N	3.146882	4.786677	-3.19312
C	3.1807	3.328077	-3.13674
C	4.646436	2.883081	-3.21777
C	2.349926	2.749045	-4.28954
O	5.350131	3.247473	-4.14358
C	1.898178	1.300153	-4.06675
C	0.881906	1.156516	-2.96824
O	-0.15152	0.442943	-3.12487
O	1.067511	1.775311	-1.86857
H	3.189073	5.221879	-4.10968
H	2.736143	3.037388	-2.18155
H	1.46753	3.393052	-4.43874
H	2.955348	2.794106	-5.20815
H	2.784031	0.678201	-3.83377
H	1.47009	0.891365	-4.992
N	5.075653	2.128717	-2.17794
H	4.468203	1.999454	-1.3709
C	6.429852	1.588826	-2.16139
H	6.561254	1.102179	-1.18049
C	6.67402	0.557503	-3.27541
H	6.794387	1.084533	-4.22785
H	7.598549	0.013737	-3.04527
C	5.482219	-0.39415	-3.37922
O	5.440386	-1.38852	-2.56526
O	4.588271	-0.12549	-4.19926
C	7.459944	2.736508	-2.22244
O	8.510395	2.643026	-2.83088
N	7.118269	3.831503	-1.48002
H	6.185349	3.836606	-1.08469
C	7.798896	5.112392	-1.60753

H	7.839747	5.405352	-2.66958
C	7.049848	6.172416	-0.7997
H	7.55043	7.142371	-0.91878
H	7.054674	5.906066	0.270578
H	6.002397	6.265666	-1.12573
C	9.268386	5.090451	-1.1446
O	10.02723	5.982569	-1.47617
N	9.57988	4.059068	-0.32715
H	8.836569	3.41558	-0.08049
C	10.8744	3.828543	0.269902
H	11.50032	4.698321	0.016066
C	10.7493	3.703747	1.803862
H	10.59062	4.706448	2.227031
H	11.70587	3.313667	2.175975
C	9.602138	2.798064	2.183781
C	8.352429	3.339393	2.5255
H	8.245074	4.426486	2.586902
C	7.260716	2.511629	2.804122
H	6.301994	2.95274	3.092817
C	7.399479	1.121833	2.74078
H	6.582384	0.458792	3.030622
C	8.634536	0.57319	2.375491
H	8.749442	-0.51213	2.32119
C	9.725986	1.400308	2.101847
H	10.68908	0.967453	1.827067
C	11.59443	2.591201	-0.31607
O	12.52035	2.07841	0.301471
N	11.14952	2.16715	-1.51832
H	10.34616	2.622226	-1.94662
C	11.75042	1.055072	-2.23467
H	12.65073	0.768168	-1.67625
C	10.80378	-0.13509	-2.41628
H	9.961258	0.180281	-3.05553
H	11.35016	-0.90984	-2.98428
C	10.24552	-0.75836	-1.12652
H	9.609705	0.002336	-0.63557
C	9.364484	-1.96665	-1.46033
H	8.538299	-1.70527	-2.13994
H	9.960193	-2.75546	-1.95051
H	8.91776	-2.39753	-0.55277
C	11.3454	-1.15679	-0.13625
H	11.95005	-0.29718	0.186279
H	10.90707	-1.62018	0.762759
H	12.02585	-1.8991	-0.58848
C	5.383873	-3.61999	5.461015
H	5.505055	-3.03998	6.388681
C	4.246243	-3.05525	4.601784

H	4.004515	-3.78807	3.816144
H	3.336213	-2.92837	5.209144
C	4.485974	-1.74652	3.852511
O	5.582197	-1.19344	3.885313
O	3.466285	-1.35246	3.162408
C	4.990319	-5.06257	5.714258
O	5.253491	-5.96627	4.924506
N	4.123489	-5.27211	6.746871
H	3.95806	-4.52984	7.415492
C	3.387093	-6.51626	6.852356
H	4.066599	-7.38031	6.88163
C	2.428848	-6.78443	5.678336
O	1.98237	-7.89496	5.470299
N	2.140704	-5.70998	4.885412
H	2.536298	-4.80912	5.115446
C	1.398346	-5.86154	3.653476
H	0.431998	-6.34522	3.855361
C	1.162027	-4.50411	2.973284
H	0.557183	-4.69124	2.073071
H	2.12312	-4.07735	2.651783
C	0.409499	-3.51285	3.838121
O	-0.73951	-3.77061	4.232181
N	1.059086	-2.38202	4.141526
H	1.959961	-2.08572	3.71085
H	0.574178	-1.65001	4.650776
C	2.095719	-6.75437	2.611844
O	1.397822	-7.26334	1.731207
N	3.434384	-6.83116	2.654722
H	3.93852	-6.49039	3.476056
C	4.196762	-7.59354	1.675
H	3.686553	-7.49993	0.706786
C	5.651034	-7.11876	1.567338
H	6.133183	-7.22912	2.5512
H	6.165023	-7.81821	0.887908
C	5.83716	-5.69886	1.076436
C	5.726751	-5.36159	-0.2816
H	5.511739	-6.14433	-1.01576
C	5.910501	-4.05054	-0.72953
H	5.842778	-3.80297	-1.79028
C	6.206257	-3.03103	0.190575
O	6.343651	-1.73167	-0.1813
H	6.126827	-1.60541	-1.16316
C	6.345435	-3.35503	1.546642
H	6.597236	-2.57041	2.261513
C	6.159607	-4.67032	1.973697
H	6.27289	-4.91905	3.029549
C	-3.91623	6.758855	-3.83491

C	-4.08952	6.495183	-2.34603
C	-3.40968	5.518758	-4.61035
O	-3.19981	6.776727	-1.53796
C	-2.36055	4.763544	-3.86562
C	-2.39149	3.489518	-3.34201
N	-1.169	5.304399	-3.42227
C	-0.54593	4.387083	-2.65467
N	-1.26262	3.2755	-2.57784
H	-4.85626	7.11515	-4.28378
H	-3.05052	5.845927	-5.60023
H	-4.24266	4.824355	-4.79661
H	-3.14502	2.721711	-3.48228
H	0.398537	4.578373	-2.1578
H	-0.82748	6.263488	-3.47583
N	-5.25143	5.935472	-1.95516
H	-5.93464	5.594696	-2.62865
C	-5.58448	5.692593	-0.56858
H	-5.58098	6.622517	0.020155
H	-4.84105	5.029302	-0.09273
C	-6.94843	5.000152	-0.52452
O	-7.33294	4.330621	-1.48013
N	-7.65397	5.175467	0.609808
H	-7.31353	5.828309	1.326721
C	-8.99266	4.631637	0.758066
H	-9.49766	4.670654	-0.21967
C	-9.02472	3.194525	1.290778
H	-8.52345	2.532313	0.565704
H	-8.43646	3.151945	2.22004
C	-10.4469	2.709285	1.568645
H	-11.024	2.645376	0.630714
H	-10.974	3.451216	2.195513
C	-10.5263	1.346142	2.262737
H	-11.5776	1.05572	2.408198
H	-10.0847	0.559576	1.634575
N	-9.8758	1.319874	3.572603
H	-10.3554	1.805172	4.322338
C	-8.64287	0.858354	3.844336
N	-7.90377	0.233853	2.93343
H	-8.22585	0.067083	1.991185
H	-6.97521	-0.10262	3.24832
N	-8.11868	1.023166	5.067441
H	-8.66505	1.406122	5.826549
H	-7.19511	0.607855	5.248748
C	-3.85992	-2.52094	-7.04919
H	-3.82189	-3.38951	-7.71901
H	-3.90256	-1.6211	-7.68473
N	-2.65866	-2.52973	-6.23401

H	-2.13473	-3.44045	-6.13252
C	-2.37861	-1.58688	-5.3434
N	-3.15748	-0.49233	-5.2117
H	-4.04989	-0.48362	-5.68777
H	-3.12706	0.01846	-4.32546
N	-1.30997	-1.70453	-4.53652
H	-0.63995	-2.47445	-4.63019
H	-0.87201	-0.83952	-4.19426
O	-3.44166	0.528441	-2.48237
C	-3.63889	1.203123	-1.46469
O	-2.75144	1.333042	-0.52299
C	-4.92356	1.977705	-1.27931
H	-5.25363	1.960364	-0.2338
H	-4.73048	3.029128	-1.54434
O	-0.17161	0.235504	0.222766
O	-1.25474	-0.98649	2.422903
Fe	-0.80177	1.828187	-1.00114
C	-0.39754	-2.12811	-1.01922
N	-1.50895	-1.31653	-1.56479
H	-2.08014	-0.93539	-0.80094
H	-1.13218	-0.52594	-2.14132
H	-2.11202	-1.86615	-2.18381
C	-0.93944	-3.18149	-0.02351
O	-1.99122	-2.83226	0.61427
O	-0.31032	-4.23237	0.065441
C	0.70363	-1.22359	-0.50374
C	1.66857	-1.78534	0.507791
C	3.118009	-1.70946	0.022744
C	3.684448	-0.29448	-0.02163
N	4.551253	-0.02809	1.153506
H	4.76707	0.972163	1.238188
H	4.100058	-0.41595	2.061744
H	5.438381	-0.54726	0.998523
H	-9.68866	-2.05262	-1.27379
H	-4.35608	9.764883	3.73624
H	-3.10398	10.50906	-0.62897
H	4.296585	7.43645	-2.29506
H	12.07916	1.406145	-3.22767
H	-7.30962	6.019378	5.125114
H	-9.5417	5.294067	1.445716
H	-3.18401	7.575002	-3.91289
H	-5.71743	1.613462	-1.94062
H	6.333354	-3.58549	4.913914
H	4.180238	-8.66539	1.942045
H	2.807121	-6.51651	7.786287
H	-4.30807	-0.51091	6.486298
H	-4.80896	-4.76761	4.279808

H	3.114429	-6.96313	-1.59194
H	0.614488	-10.6207	-1.26376
H	-4.48952	-5.25333	-6.16385
H	-1.03308	-0.44267	1.6335
H	-1.45957	-1.86433	2.029493
H	4.292695	-0.17327	-0.92273
H	2.893426	0.46264	-0.04356
H	3.19857	-2.13456	-0.98178
H	3.735268	-2.34722	0.667703
H	1.559547	-1.26384	1.468846
H	1.395746	-2.82755	0.712858
H	0.54555	0.658921	0.762984
H	1.172254	-0.66021	-1.31116
H	0.044309	-2.67473	-1.87299
H	-4.77767	-2.59277	-6.43948
O	1.492234	1.689625	1.8308
H	0.989818	2.514191	1.908487
H	1.311241	1.225793	2.70894
O	-2.02361	1.834554	2.135654
H	-2.57133	1.489095	1.405269
H	-1.39841	2.410054	1.662423
O	3.795486	2.694302	0.40743
H	3.603922	3.553596	-0.01502
H	3.013902	2.441089	0.934378
O	0.499623	0.481448	3.942296
H	-0.08906	-0.13264	3.449686
H	-0.12777	1.0881	4.39668
O	-1.59154	2.041307	4.774524
H	-1.92625	2.001341	3.848224
H	-1.51948	2.981019	4.985093
O	3.898197	7.058031	0.919715
H	3.88979	6.226869	0.400668
H	4.77323	7.127032	1.323541

^{5}Pr$_{C,C3}$

C	-3.91006	6.135064	-2.38673
H	-4.68014	5.934647	-1.62847
C	-2.8217	5.054157	-2.32969
H	-1.95973	5.360183	-2.93424
H	-2.46277	4.958363	-1.29112
C	-3.31421	3.710617	-2.86034
H	-3.90156	3.154362	-2.11474
H	-4.0102	3.876809	-3.70253
C	-2.21182	2.827443	-3.42407
O	-1.08724	3.292765	-3.69234
N	-2.54483	1.55247	-3.6276
H	-3.46951	1.141162	-3.38151
H	-1.90522	0.938634	-4.12043
C	-3.34015	7.534434	-2.23082
O	-2.89188	8.157217	-3.20742
N	-3.33107	8.028133	-0.98456
H	-3.52688	7.384995	-0.20486
C	-2.7857	9.330188	-0.64258
H	-3.04939	9.510327	0.410352
C	-1.26858	9.457907	-0.82948
H	-1.05309	9.525395	-1.90799
H	-0.95497	10.41768	-0.38302
C	-0.46722	8.307686	-0.22183
H	-0.68614	7.373791	-0.76412
H	-0.76734	8.119271	0.822528
C	1.034645	8.574291	-0.29036
H	1.314726	8.894688	-1.30941
H	1.291825	9.412469	0.387305
N	1.787718	7.375678	0.041501
H	1.377363	6.696973	0.722505
C	3.090181	7.220204	-0.18222
N	3.853539	8.258931	-0.61163
H	3.557602	9.191827	-0.34755
H	4.864894	8.072608	-0.62383
N	3.651312	6.023993	-0.02388
H	3.077923	5.172944	0.086687
H	4.666316	5.950015	-0.01118
C	3.470924	7.00323	-4.67963
H	3.165589	8.056712	-4.58901
C	2.538909	6.124578	-3.81593
H	2.872044	5.078339	-3.87538
H	2.621445	6.418448	-2.75936
C	1.118021	6.238614	-4.26502
N	0.638596	5.56367	-5.37134
C	-0.61665	5.962157	-5.53348

H	-1.30203	5.604672	-6.29953
N	-0.96103	6.876175	-4.59929
H	-1.87445	7.325638	-4.39356
C	0.124452	7.059607	-3.77848
H	0.090529	7.742104	-2.93749
C	4.922858	6.822476	-4.29885
O	5.586769	5.845836	-4.64627
N	5.441235	7.790642	-3.49657
H	4.81708	8.492636	-3.11439
C	6.783376	7.685448	-2.93977
H	7.434033	7.311572	-3.74387
C	7.283044	9.039394	-2.45685
H	8.30663	8.954885	-2.0654
H	6.651566	9.430861	-1.64504
H	7.284559	9.760471	-3.28674
C	6.750028	6.646064	-1.80612
O	6.327866	6.941116	-0.68007
N	7.156077	5.416044	-2.15148
H	7.304772	5.261144	-3.14584
C	7.100507	4.251839	-1.28529
H	6.570769	4.531979	-0.3633
C	6.412632	3.056059	-1.95656
H	6.950515	2.85527	-2.90329
C	6.553654	1.823273	-1.06546
H	7.61146	1.574046	-0.88602
H	6.056012	1.996689	-0.0981
H	6.087573	0.954442	-1.54312
C	4.945357	3.338973	-2.28802
H	4.849654	4.214354	-2.94426
H	4.360207	3.515233	-1.37346
H	4.508173	2.477143	-2.81649
C	2.893205	0.947101	5.774021
H	2.792018	-0.13728	5.936468
C	1.498722	1.577789	5.714697
H	0.885772	0.982826	5.017159
C	0.813182	1.55223	7.075677
H	-0.18835	2.00356	7.003212
H	1.393817	2.133013	7.808943
H	0.698611	0.521397	7.445838
O	1.625525	2.902318	5.225061
H	0.717227	3.236668	4.999733
C	3.719085	1.161905	4.529542
O	4.949897	1.357817	4.604277
N	3.091349	1.16074	3.342405
H	2.071415	1.043938	3.279754
C	3.710721	1.699771	2.153399
H	4.643644	1.181435	1.899678

H	3.021199	1.556966	1.310166
C	4.002959	3.211699	2.221414
O	4.899662	3.689761	1.516193
N	3.207982	3.890433	3.057189
H	2.587868	3.370316	3.693893
C	3.227614	5.326567	3.26784
H	3.830537	5.781409	2.472601
C	1.808788	5.905417	3.292534
H	1.231072	5.451219	4.11483
H	1.883843	6.984245	3.506924
O	1.105914	5.761192	2.078655
H	0.864499	4.816683	1.892859
C	7.390948	-3.98465	4.529715
H	6.631267	-4.32336	5.250183
C	6.946533	-2.70415	3.82324
H	7.795354	-2.30948	3.243627
H	6.715072	-1.94562	4.590674
C	5.7379	-2.84417	2.881936
H	6.060731	-3.45871	2.023694
C	4.55353	-3.55366	3.544922
H	4.777384	-4.61052	3.751501
H	3.674425	-3.53643	2.884326
H	4.258668	-3.05383	4.481247
C	5.343455	-1.46258	2.359489
H	4.504927	-1.52118	1.65297
H	6.185329	-0.97152	1.849191
H	5.031908	-0.80932	3.186489
C	7.736287	-5.10329	3.564848
O	8.330024	-4.90561	2.504377
N	7.392743	-6.35092	3.964797
H	6.816636	-6.45608	4.79081
C	7.60187	-7.49601	3.123852
H	8.545012	-7.35739	2.572955
C	6.482767	-7.6811	2.090883
O	5.668383	-6.7874	1.861443
N	6.506978	-8.85914	1.444542
H	7.129401	-9.58451	1.779995
C	5.523715	-9.2	0.436194
H	5.630978	-8.54409	-0.43754
C	4.103638	-9.10699	1.003147
O	3.8283	-9.49484	2.125173
N	3.195687	-8.57119	0.147704
C	1.814533	-8.42028	0.5398
C	0.981103	-8.14097	-0.71067
C	1.626404	-7.29237	1.602133
O	1.502063	-7.74011	-1.75473
C	2.046828	-5.95189	1.105181

C	1.31409	-4.84324	0.736657
N	3.367444	-5.62505	0.841996
C	3.413719	-4.38959	0.333083
N	2.180962	-3.87049	0.25178
H	3.497043	-8.12112	-0.71491
H	1.477487	-9.35486	1.014209
H	0.574912	-7.25325	1.918691
H	2.228752	-7.59384	2.471849
H	0.241388	-4.68369	0.794136
H	4.32874	-3.89126	0.029859
H	4.200592	-6.20787	1.045694
N	-0.34697	-8.31405	-0.57044
H	-0.75198	-8.45339	0.363831
C	-1.26564	-7.84313	-1.58208
H	-0.89119	-8.11376	-2.57793
C	-1.47966	-6.33105	-1.47259
O	-1.62844	-5.79808	-0.35833
N	-1.4637	-5.64321	-2.6168
C	-1.72042	-4.207	-2.71684
C	-3.23294	-4.00251	-2.89557
C	-0.97373	-3.6663	-3.94334
O	-3.81931	-4.62562	-3.76503
C	-0.96803	-2.1486	-4.10062
C	-0.1631	-1.35228	-3.11695
O	0.32144	-0.26003	-3.44622
O	-0.02611	-1.81968	-1.89624
H	-1.49103	-6.16552	-3.48801
H	-1.36157	-3.73777	-1.79517
H	0.055669	-4.04956	-3.92733
H	-1.46799	-4.0905	-4.83206
H	-2.00605	-1.74596	-4.07505
H	-0.60551	-1.87751	-5.10248
N	-3.80805	-3.11829	-2.05032
H	-3.30428	-2.88169	-1.19166
C	-5.24089	-2.84223	-2.13696
H	-5.49784	-2.23119	-1.25883
C	-5.60768	-2.07145	-3.41105
H	-5.54918	-2.74519	-4.27227
H	-6.63938	-1.70713	-3.31355
C	-4.63331	-0.91107	-3.60603
O	-4.7955	0.124394	-2.85741
O	-3.70446	-1.0296	-4.41649
C	-6.03695	-4.15801	-2.01116
O	-6.95763	-4.44686	-2.75149
N	-5.63466	-4.9555	-0.97249
H	-4.82338	-4.64998	-0.44593
C	-5.94244	-6.38054	-0.93656

H	-5.72102	-6.81814	-1.92456
C	-5.09798	-7.07263	0.133063
H	-5.33909	-8.14399	0.139085
H	-5.32677	-6.66069	1.129961
H	-4.02043	-6.94671	-0.05544
C	-7.42447	-6.72525	-0.70198
O	-7.80878	-7.85871	-0.93221
N	-8.19652	-5.72712	-0.21423
H	-7.76351	-4.83149	-0.01657
C	-9.62105	-5.85905	0.014717
H	-9.85913	-6.92057	-0.15396
C	-9.99607	-5.47948	1.460144
H	-9.53075	-6.21183	2.137267
H	-11.0861	-5.57595	1.544603
C	-9.56047	-4.08006	1.827171
C	-8.29361	-3.84307	2.38899
H	-7.64201	-4.69431	2.610361
C	-7.87553	-2.54562	2.700217
H	-6.90504	-2.35244	3.162013
C	-8.72451	-1.46269	2.454944
H	-8.39884	-0.46306	2.740797
C	-9.98163	-1.68435	1.8883
H	-10.65	-0.84208	1.694832
C	-10.3959	-2.98019	1.572988
H	-11.3729	-3.15404	1.120161
C	-10.4722	-5.05239	-0.99341
O	-11.6526	-4.83156	-0.75238
N	-9.83156	-4.65054	-2.11236
H	-8.84096	-4.85164	-2.2261
C	-10.4538	-3.82646	-3.13276
H	-11.5338	-3.82578	-2.93481
C	-9.88698	-2.40182	-3.16004
H	-8.81265	-2.4654	-3.40423
H	-10.3644	-1.85739	-3.99519
C	-10.0619	-1.59839	-1.86234
H	-9.58618	-2.17124	-1.04645
C	-9.33746	-0.2526	-1.95897
H	-8.26666	-0.38147	-2.17825
H	-9.77445	0.377058	-2.75323
H	-9.40632	0.306435	-1.01245
C	-11.5372	-1.40755	-1.49631
H	-12.0352	-2.3679	-1.29999
H	-11.638	-0.78839	-0.59121
H	-12.0789	-0.89339	-2.30924
C	-6.69695	2.548767	5.217712
H	-6.78158	2.062938	6.201607
C	-5.40638	2.134278	4.499354

H	-5.22123	2.84773	3.681957
H	-4.54257	2.200671	5.180528
C	-5.35252	0.768776	3.814777
O	-6.31413	0.011117	3.816607
O	-4.22972	0.569789	3.201932
C	-6.60352	4.059565	5.306989
O	-6.97969	4.795589	4.397393
N	-5.85922	4.549707	6.340931
H	-5.5929	3.92823	7.0949
C	-5.36787	5.9135	6.329135
H	-6.19276	6.629802	6.202936
C	-4.37403	6.226938	5.195299
O	-4.09115	7.37275	4.906761
N	-3.87169	5.145924	4.528041
H	-4.15158	4.219855	4.82007
C	-3.10854	5.293404	3.309126
H	-2.27448	5.990128	3.476152
C	-2.55462	3.945802	2.829385
H	-1.91494	4.15591	1.962382
H	-3.37618	3.29444	2.49858
C	-1.68989	3.229715	3.849478
O	-0.67622	3.781459	4.305473
N	-2.08531	1.996507	4.186555
H	-2.89381	1.499589	3.748242
H	-1.50365	1.436376	4.802654
C	-3.91612	5.8789	2.135366
O	-3.28742	6.298264	1.155393
N	-5.25062	5.822329	2.212486
H	-5.69968	5.4961	3.071229
C	-6.1387	6.322587	1.174761
H	-5.56244	6.385746	0.242859
C	-7.37533	5.433448	0.989125
H	-7.92506	5.388883	1.941575
H	-8.03431	5.942441	0.266556
C	-7.07235	4.030397	0.513643
C	-6.72536	3.766579	-0.82017
H	-6.70143	4.586719	-1.54397
C	-6.43066	2.47337	-1.25836
H	-6.17703	2.275955	-2.30101
C	-6.47691	1.402687	-0.35187
O	-6.13813	0.13785	-0.71806
H	-5.69154	0.131854	-1.64183
C	-6.85287	1.641597	0.974949
H	-6.92761	0.804403	1.66803
C	-7.13952	2.941325	1.394892
H	-7.43366	3.123698	2.429696
C	5.802801	-6.58733	-3.72856

C	5.940409	-6.19963	-2.26069
C	5.052949	-5.52401	-4.56367
O	5.192248	-6.67056	-1.39924
C	3.84596	-4.99579	-3.86162
C	3.491845	-3.70886	-3.5241
N	2.879732	-5.79803	-3.27851
C	2.014294	-5.0189	-2.61256
N	2.354547	-3.73928	-2.73525
H	6.790816	-6.77208	-4.1773
H	4.788	-5.96374	-5.53977
H	5.710855	-4.66886	-4.778
H	3.978312	-2.77655	-3.79759
H	1.188735	-5.4249	-2.03953
H	2.840835	-6.81283	-3.20494
N	6.90357	-5.30238	-1.97403
H	7.400653	-4.81226	-2.71605
C	7.200384	-4.81884	-0.64608
H	7.770975	-5.55196	-0.05319
H	6.276177	-4.64648	-0.0737
C	7.996385	-3.5146	-0.78289
O	8.073231	-2.95231	-1.87202
N	8.58732	-3.07495	0.347209
H	8.530954	-3.66067	1.189226
C	9.51174	-1.95391	0.331346
H	9.806138	-1.78539	-0.71474
C	8.944564	-0.65831	0.918983
H	8.043609	-0.36814	0.35452
H	8.6185	-0.84304	1.95582
C	9.9694	0.477221	0.894265
H	10.24406	0.716378	-0.14715
H	10.90536	0.145981	1.380098
C	9.495873	1.771591	1.561986
H	10.26181	2.556029	1.462678
H	8.594115	2.144175	1.061092
N	9.190736	1.591223	2.980294
H	9.958912	1.304653	3.577249
C	7.957042	1.546212	3.512042
N	6.890584	2.005595	2.872866
H	6.91412	2.673783	2.110156
H	5.992096	1.881821	3.377459
N	7.770295	1.010979	4.729733
H	8.49867	0.488888	5.196492
H	6.829134	1.045449	5.130855
C	3.647226	3.975856	-6.70236
H	3.393481	4.841439	-7.3281
H	4.022488	3.191094	-7.37954
N	2.435985	3.549898	-6.02361

H	1.654344	4.255283	-5.92641
C	2.31629	2.407853	-5.36194
N	3.32112	1.499787	-5.33934
H	4.215203	1.756763	-5.73505
H	3.350969	0.819707	-4.57941
N	1.189243	2.11467	-4.70262
H	0.388807	2.758703	-4.59641
H	0.96996	1.137449	-4.5107
O	3.755864	-0.05174	-2.76615
C	3.909902	-0.74191	-1.76348
O	2.95678	-0.91988	-0.86895
C	5.20411	-1.4774	-1.48083
H	5.034196	-2.55966	-1.58401
H	5.999894	-1.20402	-2.1815
O	-0.124	0.203828	-0.00719
O	0.458803	1.070778	2.491836
Fe	1.708285	-2.4434	-1.239
C	0.407916	2.314687	-1.03305
N	1.498809	1.543761	-1.69581
H	1.89736	0.822232	-1.07161
H	1.143453	0.986879	-2.50099
H	2.255962	2.178024	-1.9703
C	1.073325	3.179942	0.065925
O	0.443076	3.393107	1.133333
O	2.197311	3.62364	-0.25124
C	-0.7117	1.327749	-0.68749
C	-1.86415	1.92726	0.110821
C	-3.16205	1.125717	0.07879
C	-2.99504	-0.32763	0.513249
N	-4.25968	-0.87114	1.067523
H	-4.19353	-1.8904	1.165969
H	-4.38001	-0.39172	2.049957
H	-5.07044	-0.61715	0.453744
H	8.129743	3.967185	-0.99811
H	7.701173	-8.40066	3.742755
H	5.697643	-10.2397	0.12436
H	-2.24085	-8.3323	-1.43385
H	-10.2987	-4.29881	-4.11708
H	8.303721	-3.78331	5.117076
H	10.41753	-2.24554	0.88931
H	5.2539	-7.53941	-3.74428
H	5.530597	-1.29422	-0.44879
H	-7.57472	2.268217	4.622892
H	-6.45857	7.348971	1.427394
H	-4.88309	6.133914	7.290762
H	3.476506	1.372	6.603722
H	3.710322	5.557136	4.234949

H	-4.4	6.113792	-3.37243
H	-3.28436	10.10336	-1.24791
H	3.360883	6.72263	-5.73583
H	0.314737	0.587908	1.648785
H	0.331371	2.012236	2.227258
H	-2.67861	-0.95477	-0.32991
H	-2.23656	-0.43518	1.297409
H	-3.61185	1.143283	-0.92258
H	-3.87568	1.616546	0.756145
H	-1.53394	2.047655	1.145549
H	-2.05916	2.94306	-0.26324
H	-0.22212	-0.58522	-0.57707
H	-1.10887	0.968288	-1.64749
H	0.01839	3.016305	-1.78851
H	4.443379	4.289753	-6.00554
O	-0.73116	-2.43337	2.099933
H	-0.00282	-1.90786	1.728375
H	-0.9495	-1.92481	2.92353
O	1.896282	-1.43385	1.680554
H	2.392841	-0.87161	1.058723
H	2.110588	-2.33705	1.376982
O	-2.71047	-3.36729	0.52996
H	-2.38947	-4.25441	0.281079
H	-2.00375	-3.00187	1.119642
O	-0.76054	-0.7094	4.188435
H	-0.47639	0.027864	3.60497
H	0.09435	-1.05322	4.520669
O	1.83161	-1.74324	4.445945
H	1.974332	-1.53923	3.496757
H	1.66343	-2.69563	4.459796
O	-1.70911	-7.62835	1.705287
H	-1.85554	-6.84144	1.140426
H	-2.52058	-7.76723	2.211176

[5]**TS1**$_{C,C4}$

C	1.000584	-6.02854	-3.12233
H	1.105058	-5.52874	-2.14617
C	0.492985	-5.07881	-4.20086
H	0.294785	-5.66917	-5.10616
H	-0.47261	-4.64456	-3.90033
C	1.469107	-3.96424	-4.55044
H	1.597621	-3.26105	-3.71044
H	2.477814	-4.36357	-4.73768
C	1.015588	-3.17292	-5.77038
O	-0.16172	-3.15264	-6.13958
N	1.989796	-2.49826	-6.41086
H	2.933963	-2.41163	-6.02042
H	1.732211	-1.95047	-7.22423
C	0.130554	-7.27485	-2.98454
O	-0.46084	-7.77117	-3.94987
N	0.097412	-7.82107	-1.75484
H	0.423567	-7.27219	-0.95053
C	-0.68732	-9.00446	-1.44362
H	-0.33574	-9.36937	-0.4664
C	-2.20055	-8.75845	-1.38755
H	-2.57462	-8.66187	-2.41921
H	-2.67567	-9.66214	-0.9678
C	-2.58823	-7.52441	-0.57489
H	-2.19125	-6.61271	-1.04672
H	-2.13078	-7.55809	0.427713
C	-4.0997	-7.3778	-0.43889
H	-4.57135	-7.45512	-1.4369
H	-4.50563	-8.2091	0.168607
N	-4.43746	-6.09798	0.161572
H	-3.66269	-5.44829	0.39447
C	-5.66748	-5.59742	0.205329
N	-6.72374	-6.30031	-0.28671
H	-6.63882	-7.30496	-0.37074
H	-7.64275	-5.91198	-0.05728
N	-5.87301	-4.37256	0.684468
H	-5.12705	-4.00487	1.307148
H	-6.83953	-4.04885	0.759655
C	-6.76309	-4.95511	-4.51178
H	-6.91417	-6.02986	-4.69952
C	-5.50606	-4.75081	-3.6379
H	-5.43184	-3.6859	-3.36943
H	-5.61302	-5.30498	-2.69357
C	-4.25497	-5.17624	-4.3299
N	-3.74002	-4.46988	-5.40121
C	-2.66008	-5.13176	-5.79799

H	-1.97147	-4.81838	-6.58013
N	-2.46335	-6.23496	-5.03885
H	-1.64017	-6.85435	-5.00028
C	-3.46183	-6.2771	-4.09629
H	-3.51361	-7.06448	-3.35404
C	-7.96946	-4.33974	-3.83841
O	-8.33598	-3.18681	-4.0609
N	-8.57079	-5.12392	-2.90264
H	-8.14807	-6.01669	-2.67509
C	-9.63025	-4.63044	-2.02911
H	-10.2829	-3.99989	-2.65092
C	-10.4341	-5.77903	-1.44127
H	-11.2365	-5.39642	-0.79549
H	-9.80422	-6.43583	-0.82216
H	-10.885	-6.37677	-2.24629
C	-8.99178	-3.74066	-0.94102
O	-8.63083	-4.21328	0.145636
N	-8.83034	-2.46538	-1.31767
H	-9.02904	-2.28065	-2.3018
C	-8.19373	-1.39569	-0.56538
H	-7.83245	-1.78394	0.396519
C	-7.04828	-0.74815	-1.36436
H	-7.38885	-0.69604	-2.41542
C	-6.78345	0.677304	-0.87904
H	-7.6661	1.323261	-1.02093
H	-6.52322	0.655004	0.190565
H	-5.93615	1.12268	-1.41955
C	-5.77474	-1.59725	-1.31513
H	-5.97658	-2.65017	-1.55374
H	-5.32912	-1.56613	-0.3093
H	-5.03332	-1.23028	-2.04328
C	-2.64746	0.812417	5.976717
H	-2.03165	1.721431	6.064736
C	-1.70851	-0.38503	5.80049
H	-1.0102	-0.14879	4.984478
C	-0.88453	-0.64465	7.055038
H	-0.2154	-1.50543	6.897728
H	-1.54061	-0.88045	7.907228
H	-0.2668	0.230835	7.307486
O	-2.47683	-1.52574	5.441449
H	-1.89533	-2.1407	4.929061
C	-3.62184	0.987007	4.837887
O	-4.80492	1.324328	5.051089
N	-3.19055	0.721497	3.59645
H	-2.22931	0.403383	3.413022
C	-4.10408	0.500556	2.500428
H	-4.68847	1.397186	2.254169

H	-3.50829	0.236449	1.615739
C	-5.07768	-0.66882	2.72702
O	-6.11682	-0.73589	2.06095
N	-4.68793	-1.55739	3.648458
H	-3.82587	-1.3956	4.186195
C	-5.37368	-2.7918	3.964171
H	-6.23041	-2.8925	3.283037
C	-4.41023	-3.97114	3.818766
H	-3.58104	-3.87073	4.539925
H	-4.94371	-4.90485	4.060705
O	-3.91438	-4.09136	2.50697
H	-3.28728	-3.37133	2.259167
C	-3.54711	7.859721	4.706815
H	-2.72828	7.768958	5.435574
C	-4.10904	6.481765	4.356427
H	-5.05555	6.603244	3.80521
H	-4.36739	5.976937	5.304191
C	-3.17468	5.568352	3.543854
H	-3.15403	5.953851	2.508609
C	-1.7306	5.568346	4.057232
H	-1.2583	6.555233	3.946038
H	-1.13261	4.851732	3.478438
H	-1.68463	5.270845	5.119702
C	-3.75117	4.1526	3.517676
H	-3.12507	3.47237	2.928148
H	-4.76894	4.134827	3.098957
H	-3.81077	3.740567	4.536758
C	-3.08774	8.659236	3.503451
O	-3.7459	8.74445	2.465411
N	-1.92269	9.330385	3.658506
H	-1.37818	9.177759	4.497252
C	-1.41596	10.22239	2.652787
H	-2.20994	10.9218	2.344876
C	-0.88386	9.522033	1.395078
O	-0.6917	8.31159	1.335676
N	-0.65766	10.37864	0.379033
H	-0.74211	11.37155	0.55873
C	-0.07441	9.953374	-0.87341
H	-0.73942	9.245632	-1.38116
C	1.304183	9.320249	-0.66972
O	2.09897	9.752363	0.148592
N	1.555591	8.272438	-1.49327
C	2.823152	7.586562	-1.4305
C	3.02562	6.804144	-2.72538
C	2.936044	6.660238	-0.1802
O	2.081814	6.458685	-3.44084
C	1.861715	5.630855	-0.11685

C	1.85604	4.286653	-0.42094
N	0.552378	5.937155	0.223907
C	-0.1908	4.82797	0.112767
N	0.565835	3.798606	-0.27326
H	0.794754	7.836714	-2.01783
H	3.618895	8.339964	-1.33012
H	3.92495	6.181715	-0.16666
H	2.882417	7.326124	0.694022
H	2.68467	3.653763	-0.7296
H	-1.25659	4.776606	0.3031
H	0.178967	6.85776	0.498417
N	4.310884	6.501189	-3.00915
H	5.027754	6.589119	-2.27566
C	4.611735	5.577208	-4.07764
H	4.021123	5.832982	-4.96746
C	4.338327	4.145358	-3.62867
O	4.69544	3.749521	-2.51133
N	3.673262	3.33867	-4.46803
C	3.333348	1.993158	-4.03398
C	4.610368	1.146174	-3.90961
C	2.330005	1.343147	-4.98676
O	5.425678	1.09529	-4.81268
C	1.597116	0.145137	-4.36663
C	0.580215	0.535425	-3.31343
O	-0.60407	0.139388	-3.41484
O	0.97664	1.270443	-2.35039
H	3.467016	3.640622	-5.413
H	2.87978	2.091597	-3.04425
H	1.58953	2.100019	-5.29724
H	2.863128	1.009685	-5.89121
H	2.326442	-0.53938	-3.90387
H	1.084332	-0.42265	-5.14952
N	4.705026	0.481778	-2.7249
H	4.125111	0.796309	-1.93768
C	5.848861	-0.32778	-2.36069
H	5.60007	-0.80257	-1.39884
C	6.144265	-1.43884	-3.39768
H	6.660421	-1.01921	-4.26646
H	6.783836	-2.19735	-2.92301
C	4.855368	-2.06369	-3.90526
O	4.117877	-2.74946	-3.03264
O	4.463888	-1.92397	-5.0456
C	7.123824	0.49586	-2.09587
O	8.181959	-0.06714	-1.81568
N	7.007634	1.835411	-2.19611
H	6.094238	2.267721	-2.32003
C	8.180771	2.695919	-2.28769

H	8.998783	2.04664	-2.63654
C	7.965396	3.828086	-3.28106
H	8.910099	4.361187	-3.45547
H	7.245044	4.556998	-2.89196
H	7.602388	3.417834	-4.23472
C	8.596799	3.208928	-0.89476
O	8.521457	4.391465	-0.56744
N	9.074173	2.234161	-0.09293
H	8.947245	1.273594	-0.42009
C	9.608692	2.477626	1.241201
H	10.38033	3.26294	1.145015
C	8.578201	3.006526	2.255103
H	8.362739	4.046524	1.97996
H	9.083282	3.008055	3.23144
C	7.276506	2.245661	2.3581
C	6.102378	2.792097	1.812649
H	6.156818	3.731243	1.254646
C	4.864468	2.177262	2.01494
H	3.955138	2.650328	1.641861
C	4.783352	0.986407	2.746077
H	3.806924	0.546558	2.965513
C	5.953069	0.401412	3.246245
H	5.909489	-0.53575	3.806491
C	7.187849	1.029326	3.056442
H	8.093935	0.597006	3.481823
C	10.32937	1.213764	1.754087
O	10.33748	0.927435	2.940663
N	10.94848	0.471025	0.799621
H	10.89457	0.783012	-0.16231
C	11.39236	-0.89575	1.033025
H	11.59433	-0.99075	2.107851
C	10.34559	-1.91334	0.562265
H	10.17032	-1.75658	-0.51672
H	10.76993	-2.92807	0.667785
C	8.998255	-1.84414	1.298576
H	8.649867	-0.79649	1.269931
C	7.937692	-2.66564	0.565639
H	7.817649	-2.31649	-0.47113
H	8.203449	-3.73718	0.540502
H	6.970594	-2.57619	1.079036
C	9.105949	-2.25975	2.769084
H	9.755112	-1.57617	3.335326
H	8.113155	-2.26542	3.243989
H	9.518586	-3.28066	2.853902
C	5.444712	-5.8163	5.277657
H	5.966845	-6.16032	6.187058
C	5.260009	-4.29005	5.351593

H	4.523544	-4.02032	6.126899
H	6.214995	-3.82748	5.631253
C	4.823782	-3.67738	4.019624
O	5.63608	-2.96659	3.407907
O	3.641055	-3.95264	3.584439
C	4.16073	-6.61714	5.157653
O	3.969458	-7.45559	4.276772
N	3.22408	-6.34802	6.104776
H	3.43614	-5.65383	6.809884
C	1.975449	-7.06975	6.202142
H	2.155197	-8.15651	6.186224
C	0.958634	-6.81085	5.090144
O	-0.08517	-7.4373	5.067238
N	1.318511	-5.90761	4.133968
H	2.206715	-5.40367	4.177995
C	0.527809	-5.78108	2.934976
H	-0.48433	-6.13755	3.186468
C	0.442226	-4.33952	2.420566
H	-0.32071	-4.31106	1.633501
H	1.409808	-4.06013	1.984623
C	0.066687	-3.34177	3.501611
O	-1.1181	-3.06431	3.733816
N	1.098517	-2.77843	4.149213
H	2.064093	-3.08481	3.982235
H	0.948659	-1.96781	4.742402
C	1.011586	-6.69934	1.799841
O	0.564501	-6.53819	0.661058
N	1.883847	-7.66821	2.136922
H	2.324714	-7.6437	3.058937
C	2.336057	-8.68435	1.210155
H	1.791415	-8.54217	0.269078
C	3.856782	-8.63413	0.953841
H	4.390575	-8.87472	1.884282
H	4.09626	-9.4134	0.211215
C	4.283309	-7.28825	0.455871
C	3.952394	-6.86146	-0.8621
H	3.436651	-7.55568	-1.53036
C	4.243953	-5.58991	-1.28852
H	3.974954	-5.23877	-2.28584
C	4.871987	-4.6481	-0.38405
O	5.033904	-3.42869	-0.70552
H	4.556843	-2.91864	-2.14879
C	5.250965	-5.111	0.93288
H	5.751354	-4.40976	1.602643
C	4.944694	-6.39006	1.329446
H	5.165697	-6.72778	2.342608
C	-2.33951	7.35893	-4.13203

C	-2.28522	7.312562	-2.61258
C	-2.56209	5.949481	-4.72605
O	-1.21223	7.309731	-2.00493
C	-1.7093	4.920086	-4.06381
C	-2.05024	3.818448	-3.30969
N	-0.32785	4.986769	-4.00238
C	0.111017	3.965572	-3.24231
N	-0.90653	3.235413	-2.80142
H	-3.13118	8.039729	-4.4813
H	-2.37925	5.985027	-5.81313
H	-3.61122	5.643981	-4.59694
H	-3.03614	3.410482	-3.10635
H	1.159374	3.822644	-3.00514
H	0.292067	5.755626	-4.25957
N	-3.46997	7.237596	-1.97981
H	-4.33076	7.072563	-2.49823
C	-3.60491	7.118502	-0.54543
H	-3.29566	8.033538	-0.01747
H	-2.96187	6.308571	-0.15882
C	-5.06403	6.773523	-0.253
O	-5.75811	6.237468	-1.11664
N	-5.51197	7.076949	0.979844
H	-4.92367	7.64727	1.601044
C	-6.9024	6.834042	1.323848
H	-7.52742	7.094096	0.454436
C	-7.18812	5.382782	1.723581
H	-6.77122	4.71833	0.951003
H	-6.65363	5.15608	2.660866
C	-8.68247	5.103717	1.881577
H	-9.1873	5.203763	0.906313
H	-9.14817	5.861433	2.539053
C	-8.99411	3.712458	2.43878
H	-10.0765	3.514029	2.406974
H	-8.50916	2.942306	1.823218
N	-8.53137	3.562719	3.818841
H	-8.91865	4.218015	4.4894
C	-7.45852	2.860843	4.223184
N	-6.92787	1.887005	3.492466
H	-7.41917	1.402803	2.751106
H	-6.07685	1.453368	3.896545
N	-6.88891	3.136083	5.406078
H	-7.08935	3.998996	5.892782
H	-6.08632	2.564593	5.692642
C	-5.76716	-1.17152	-5.12305
H	-6.51267	-1.97127	-5.21412
H	-5.89058	-0.46616	-5.96203
N	-4.43628	-1.74767	-5.09146

H	-4.27576	-2.73592	-5.39715
C	-3.34569	-1.084	-4.71195
N	-3.43221	0.157359	-4.18635
H	-4.29973	0.504749	-3.80048
H	-2.58182	0.61528	-3.86712
N	-2.13729	-1.63463	-4.8338
H	-1.94459	-2.46748	-5.38594
H	-1.33417	-1.092	-4.50423
O	-3.50506	1.560105	-1.49935
C	-3.31113	2.422097	-0.62674
O	-2.14789	2.681246	-0.13455
C	-4.44643	3.281882	-0.1115
H	-5.31212	2.658453	0.144411
H	-4.14124	3.871114	0.760039
O	-0.28144	0.562732	0.054376
O	-0.99641	-0.43421	2.412082
Fe	-0.38278	2.063601	-0.99481
C	-1.77747	-2.33976	-1.25263
N	-2.11919	-0.8938	-1.28744
H	-1.62824	-0.36773	-0.52329
H	-1.74264	-0.44681	-2.14381
H	-3.11894	-0.68912	-1.2112
C	-2.33764	-2.99098	0.029698
O	-2.65555	-2.2118	0.976402
O	-2.38538	-4.23278	0.010509
C	-0.25367	-2.51134	-1.33392
C	0.482523	-1.82626	-0.18666
C	1.96222	-2.17196	-0.11317
C	2.618304	-1.46358	1.067954
N	3.883222	-2.10837	1.502994
H	4.486355	-1.48412	2.055143
H	3.724831	-2.92957	2.152658
H	4.458608	-2.43785	0.703964
H	-8.95749	-0.63126	-0.3393
H	-0.59608	10.81486	3.086612
H	0.05096	10.83889	-1.51325
H	5.679953	5.656704	-4.32824
H	12.34405	-1.05502	0.502254
H	-4.33132	8.463724	5.195499
H	-7.17121	7.517387	2.144293
H	-1.37594	7.768221	-4.46679
H	-4.76825	3.974152	-0.90567
H	6.086825	-6.08309	4.427336
H	2.078507	-9.68296	1.599935
H	1.494031	-6.83638	7.161649
H	-3.25286	0.705359	6.888286
H	-5.76301	-2.75782	4.997075

H	2.009093	-6.38706	-3.39844
H	-0.47308	-9.78366	-2.19019
H	-6.62662	-4.4641	-5.48433
H	-0.80303	0.024423	1.549643
H	-1.46111	-1.25656	2.145597
H	2.84729	-0.43243	0.795718
H	1.955767	-1.44981	1.942436
H	2.486728	-1.90204	-1.04599
H	2.080603	-3.26897	-0.00704
H	0.227173	-0.59113	-0.14929
H	0.022577	-2.1516	0.758768
H	0.09747	-2.14146	-2.31146
H	-0.07004	-3.60007	-1.32576
H	-2.25464	-2.81621	-2.12056
H	-6.00429	-0.64403	-4.18543
O	1.609538	1.633941	1.65808
H	0.949324	1.129746	1.117919
H	1.674471	1.11984	2.501353
O	-0.75646	2.844001	2.40477
H	-1.37592	2.695009	1.667176
H	0.135465	2.617319	2.057047
O	3.079184	1.253438	-0.62296
H	2.260368	1.117732	-1.13521
H	2.784579	1.503945	0.278669
O	1.225277	0.17834	3.916518
H	0.437881	-0.16262	3.432752
H	0.864213	0.939127	4.42902
O	-0.11593	2.359721	4.936361
H	-0.51746	2.56122	4.053894
H	0.399003	3.147511	5.157122
O	6.028973	5.649791	-0.95444
H	5.631621	4.836359	-1.32124
H	6.961351	5.407891	-0.77186

^{5}**IM1**$_{C,C4}$

C	6.442227	0.45979	-2.66276
H	5.994359	0.464053	-1.65491
C	5.380309	0.349048	-3.7526
H	5.888453	0.270127	-4.72569
H	4.824076	-0.58858	-3.63165
C	4.382983	1.501555	-3.80753
H	3.977731	1.736987	-2.8088
H	4.861171	2.432001	-4.1429
C	3.188981	1.195146	-4.71061
O	2.790928	0.030017	-4.89321
N	2.613258	2.26353	-5.26693
H	2.822676	3.214006	-4.90226
H	1.767005	2.119857	-5.80642
C	7.479717	-0.65424	-2.74412
O	7.725716	-1.25006	-3.80679
N	8.138719	-0.92664	-1.61062
H	7.832546	-0.54528	-0.70785
C	9.168184	-1.94861	-1.5095
H	9.708127	-1.74501	-0.57386
C	8.615015	-3.3801	-1.4902
H	8.297411	-3.64787	-2.51278
H	9.44226	-4.06361	-1.23066
C	7.440772	-3.55749	-0.52826
H	6.607842	-2.90804	-0.83262
H	7.705522	-3.21257	0.483985
C	6.942696	-5.00124	-0.48926
H	6.899842	-5.39941	-1.51647
H	7.644399	-5.64286	0.074336
N	5.6	-5.08343	0.076952
H	5.37551	-4.5247	0.921989
C	4.631765	-5.86596	-0.38441
N	4.893537	-6.88169	-1.23601
H	5.825162	-7.27715	-1.22999
H	4.101548	-7.50116	-1.46529
N	3.35194	-5.60443	-0.07344
H	3.145513	-4.78511	0.515194
H	2.663775	-6.3581	-0.08387
C	3.413516	-6.53065	-5.06072
H	4.443343	-6.90566	-5.16028
C	3.375973	-5.32525	-4.09901
H	2.318476	-5.04402	-3.97791
H	3.742714	-5.65322	-3.11571
C	4.178096	-4.15619	-4.56532
N	3.750239	-3.33183	-5.58915
C	4.710102	-2.43467	-5.77043

H	4.67164	-1.60095	-6.46759
N	5.742241	-2.64889	-4.92683
H	6.564721	-2.03472	-4.73727
C	5.421634	-3.73072	-4.14284
H	6.089981	-4.09483	-3.36863
C	2.46603	-7.58336	-4.52952
O	1.256101	-7.38603	-4.45428
N	3.029016	-8.7201	-4.04155
H	4.034722	-8.83185	-4.07447
C	2.244194	-9.6483	-3.2535
H	1.377122	-9.96788	-3.85381
C	3.083234	-10.8629	-2.85886
H	2.478014	-11.5754	-2.2813
H	3.935627	-10.5576	-2.23176
H	3.462553	-11.3779	-3.75387
C	1.728309	-8.92654	-1.99454
O	2.465305	-8.16565	-1.36053
N	0.477799	-9.22912	-1.62059
H	-0.08167	-9.76888	-2.27216
C	-0.20445	-8.62334	-0.48685
H	0.555395	-8.17814	0.169419
C	-1.21884	-7.54729	-0.90948
H	-1.93692	-8.01783	-1.60895
C	-1.98947	-7.07835	0.328738
H	-2.54765	-7.91657	0.783644
H	-1.27134	-6.67987	1.061609
H	-2.70922	-6.28758	0.072229
C	-0.54299	-6.37493	-1.62638
H	0.034589	-6.71784	-2.49734
H	0.126111	-5.84698	-0.93018
H	-1.29669	-5.65948	-1.98522
C	-0.02206	-2.71041	5.780035
H	-0.75637	-1.88892	5.829364
C	1.390618	-2.11443	5.74026
H	1.405004	-1.32643	4.96811
C	1.748587	-1.51365	7.098452
H	2.769618	-1.09988	7.096616
H	1.721029	-2.29223	7.876053
H	1.052102	-0.70774	7.379508
O	2.315722	-3.13036	5.375889
H	3.147975	-2.70371	5.058895
C	-0.38853	-3.59878	4.613596
O	-1.12225	-4.59693	4.796342
N	0.101781	-3.29136	3.413881
H	0.711545	-2.4582	3.281684
C	0.069319	-4.2101	2.303261
H	-0.91703	-4.67757	2.192196

H	0.256006	-3.66305	1.370552
C	1.13067	-5.31272	2.358335
O	1.069113	-6.24598	1.549504
N	2.105191	-5.14781	3.265257
H	2.060518	-4.35309	3.913888
C	3.269904	-6.00571	3.37147
H	3.343852	-6.58973	2.444315
C	4.550028	-5.20459	3.62214
H	4.481808	-4.6984	4.599421
H	5.388797	-5.92163	3.692407
O	4.805999	-4.25626	2.608286
H	4.77003	-3.35407	2.993842
C	-6.13388	-2.62144	5.306084
H	-5.85524	-1.66685	5.776814
C	-4.99523	-3.16064	4.436457
H	-5.24787	-4.18498	4.121849
H	-4.0872	-3.24608	5.057158
C	-4.66812	-2.32756	3.185793
H	-5.5359	-2.405	2.505586
C	-4.46766	-0.8454	3.521872
H	-5.4156	-0.35918	3.794619
H	-4.06605	-0.30021	2.657201
H	-3.7504	-0.72282	4.348832
C	-3.45032	-2.92098	2.471956
H	-3.27044	-2.43992	1.501007
H	-3.58897	-3.99967	2.29261
H	-2.54293	-2.78863	3.076897
C	-7.43694	-2.4767	4.540725
O	-7.84871	-3.35233	3.781394
N	-8.14296	-1.34312	4.781374
H	-7.71102	-0.61142	5.332262
C	-9.36395	-1.04459	4.083999
H	-9.87919	-1.99462	3.873015
C	-9.119	-0.34702	2.741016
O	-8.01451	-0.38438	2.199614
N	-10.1977	0.229916	2.182669
H	-11.0223	0.37619	2.752633
C	-10.1058	0.909816	0.905028
H	-9.73436	0.219117	0.137304
C	-9.19644	2.14353	0.99346
O	-9.16303	2.838965	1.995908
N	-8.46259	2.379116	-0.12166
C	-7.54897	3.498837	-0.18833
C	-7.109	3.679081	-1.63921
C	-6.31518	3.317022	0.752474
O	-7.13318	2.735382	-2.43526
C	-5.52354	2.077969	0.498252

C	-4.24271	1.893659	0.02382
N	-6.02888	0.799967	0.693908
C	-5.09998	-0.09357	0.337969
N	-3.99394	0.532963	-0.06852
H	-8.38981	1.668413	-0.85367
H	-8.06662	4.406023	0.15924
H	-5.66448	4.196769	0.665264
H	-6.72122	3.308808	1.77513
H	-3.50892	2.640311	-0.26065
H	-5.23067	-1.16999	0.358365
H	-6.94054	0.528375	1.091515
N	-6.65188	4.900005	-1.97267
H	-6.46588	5.598558	-1.23561
C	-5.87061	5.055162	-3.17844
H	-6.35153	4.528231	-4.01318
C	-4.44766	4.53913	-2.9401
O	-3.93509	4.634695	-1.8219
N	-3.79441	3.975429	-3.97957
C	-2.41358	3.526967	-3.79765
C	-1.45994	4.730779	-3.92765
C	-2.00222	2.392574	-4.73347
O	-1.10378	5.150717	-5.01686
C	-0.81442	1.585072	-4.15511
C	-1.24311	0.7464	-2.98046
O	-1.08233	-0.5113	-2.9356
O	-1.86458	1.2909	-2.01033
H	-4.19224	4.019399	-4.91213
H	-2.37245	3.162039	-2.76976
H	-2.85756	1.720594	-4.90712
H	-1.70459	2.822225	-5.70228
H	-0.01838	2.275908	-3.82051
H	-0.38568	0.919658	-4.91632
N	-1.13875	5.242559	-2.7191
H	-1.42254	4.704027	-1.90322
C	-0.09723	6.208728	-2.42074
H	0.225054	5.961278	-1.39632
C	1.130012	6.109189	-3.32419
H	0.871039	6.336083	-4.36758
H	1.872027	6.855677	-3.00949
C	1.801884	4.730093	-3.31582
O	2.918979	4.657131	-3.90999
O	1.248589	3.751525	-2.74436
C	-0.68028	7.616859	-2.25949
O	0.023986	8.595546	-2.01271
N	-2.03133	7.66187	-2.29354
H	-2.51103	6.772819	-2.3909
C	-2.82224	8.85769	-2.0541

H	-2.24956	9.703907	-2.46604
C	-4.17315	8.7475	-2.74428
H	-4.75394	9.668726	-2.60486
H	-4.75946	7.927254	-2.30508
H	-4.03371	8.571445	-3.82102
C	-2.97553	9.114792	-0.53423
O	-4.06518	9.052225	0.035065
N	-1.81378	9.426823	0.070884
H	-0.96918	9.267074	-0.48397
C	-1.68045	9.87194	1.45342
H	-2.31694	10.76775	1.574916
C	-2.1595	8.857975	2.513166
H	-3.24989	8.783153	2.416995
H	-1.93129	9.315815	3.48576
C	-1.55141	7.476091	2.44315
C	-2.28938	6.403585	1.914282
H	-3.29566	6.585809	1.529245
C	-1.76898	5.106865	1.90982
H	-2.35844	4.275486	1.519349
C	-0.48983	4.866429	2.419248
H	-0.08034	3.855179	2.430934
C	0.265399	5.926129	2.925906
H	1.270959	5.737701	3.306749
C	-0.26515	7.218607	2.945862
H	0.311421	8.042317	3.366237
C	-0.23105	10.3328	1.732173
O	0.213673	10.31261	2.869147
N	0.486583	10.75965	0.660202
H	0.05344	10.77817	-0.25414
C	1.932018	10.93923	0.699602
H	2.206474	11.10849	1.74878
C	2.67092	9.732505	0.106038
H	2.331365	9.595942	-0.93493
H	3.748586	9.97197	0.060376
C	2.470786	8.412527	0.867074
H	1.384445	8.247837	0.973404
C	3.016114	7.231632	0.063622
H	2.55464	7.176478	-0.93315
H	4.107272	7.301258	-0.07732
H	2.813896	6.290008	0.593389
C	3.082162	8.445128	2.271257
H	2.594575	9.195327	2.910605
H	2.981197	7.464294	2.76107
H	4.160053	8.678003	2.220251
C	5.851338	4.474475	4.447569
H	5.4761	5.004929	5.336664
C	4.946887	3.290688	4.083614

H	5.500897	2.674752	3.355532
H	4.785613	2.661138	4.969908
C	3.590921	3.452488	3.399994
O	3.347508	4.43384	2.676359
O	2.850354	2.426055	3.549442
C	7.208906	3.822171	4.680014
O	8.058492	3.738778	3.797253
N	7.341361	3.117256	5.840563
H	6.655488	3.238072	6.576001
C	8.394669	2.130461	5.998607
H	9.387033	2.579767	5.849484
C	8.326276	0.9521	5.004547
O	9.297873	0.255599	4.79251
N	7.129191	0.781785	4.367358
H	6.34612	1.354442	4.648672
C	6.95095	-0.13618	3.260872
H	7.315696	-1.13513	3.547811
C	5.468611	-0.24549	2.852673
H	5.400486	-0.92074	1.987945
H	5.094475	0.741408	2.539843
C	4.589318	-0.80278	3.951575
O	4.58543	-2.0176	4.222445
N	3.864498	0.090951	4.644564
H	3.581716	1.002505	4.232428
H	3.227525	-0.2832	5.338104
C	7.771483	0.219413	2.001803
O	7.823338	-0.62385	1.102094
N	8.360557	1.422245	1.934579
H	8.29757	2.066644	2.723511
C	9.291972	1.777844	0.870534
H	9.060926	1.153589	-0.00182
C	9.233876	3.263104	0.488048
H	9.454239	3.871188	1.379971
H	10.04821	3.445387	-0.23069
C	7.911364	3.683633	-0.10383
C	7.683394	3.708106	-1.4855
H	8.502378	3.471938	-2.17152
C	6.429709	4.038453	-2.01442
H	6.274394	4.067413	-3.0953
C	5.370983	4.345311	-1.15148
O	4.112358	4.616407	-1.58225
H	3.955247	4.603025	-2.56506
C	5.595974	4.371641	0.233312
H	4.800187	4.671052	0.914813
C	6.848597	4.041315	0.739228
H	7.027338	4.082137	1.813412
C	-8.51056	-1.20825	-3.51409

C	-8.27785	-1.31873	-2.01403
C	-7.24186	-1.50399	-4.3443
O	-8.20852	-0.3194	-1.29544
C	-6.02475	-0.82742	-3.80421
C	-4.79928	-1.33481	-3.43582
N	-5.97244	0.515786	-3.47458
C	-4.7734	0.773135	-2.92474
N	-4.03349	-0.32898	-2.87535
H	-9.31839	-1.88723	-3.82983
H	-7.43248	-1.20645	-5.38947
H	-7.03951	-2.58536	-4.36485
H	-4.42701	-2.34775	-3.55133
H	-4.50087	1.742681	-2.52576
H	-6.72584	1.20784	-3.41516
N	-8.11817	-2.55983	-1.51592
H	-8.15718	-3.39785	-2.09544
C	-7.90028	-2.81799	-0.11421
H	-8.75753	-2.49441	0.498905
H	-7.03805	-2.25068	0.268694
C	-7.66336	-4.31794	0.064868
O	-7.59612	-5.05628	-0.91459
N	-7.53536	-4.72536	1.342153
H	-7.66069	-4.06216	2.113657
C	-7.44066	-6.12606	1.703216
H	-7.68237	-6.7172	0.808263
C	-6.06879	-6.53054	2.250429
H	-5.30514	-6.39748	1.464275
H	-5.80117	-5.84532	3.071077
C	-6.07449	-7.97362	2.75317
H	-6.31865	-8.66177	1.925977
H	-6.88334	-8.09977	3.495124
C	-4.7614	-8.44703	3.377709
H	-4.84369	-9.50536	3.669307
H	-3.94273	-8.4034	2.645379
N	-4.36806	-7.69027	4.564477
H	-4.91046	-7.85382	5.404887
C	-3.42076	-6.7388	4.630109
N	-2.68624	-6.39796	3.576135
H	-2.79189	-6.84203	2.676044
H	-1.93898	-5.69724	3.744704
N	-3.19151	-6.09938	5.784779
H	-3.65129	-6.37826	6.640239
H	-2.41522	-5.42289	5.802472
C	0.573223	-4.78997	-6.7008
H	1.284201	-5.00276	-7.50939
H	-0.40946	-4.60636	-7.16442
N	1.039476	-3.61259	-5.98855

H	2.074023	-3.39532	-6.0144
C	0.339978	-3.0282	-5.02543
N	-0.8733	-3.47808	-4.67559
H	-1.17896	-4.38609	-5.00021
H	-1.38086	-3.11025	-3.86808
N	0.870669	-1.98139	-4.34538
H	1.609408	-1.40802	-4.78188
H	0.195176	-1.42049	-3.80168
O	-2.48286	-3.63439	-2.37659
C	-3.33853	-3.37814	-1.51402
O	-3.3848	-2.2557	-0.88424
C	-4.41427	-4.36842	-1.15195
H	-4.18939	-5.36506	-1.54876
H	-4.54877	-4.40464	-0.06192
O	-1.13929	-0.69652	0.033845
O	-0.25825	-3.13044	-0.74
Fe	-2.59457	-0.48601	-1.21263
C	3.288039	-2.16777	-1.4629
N	2.165086	-3.06217	-1.86399
H	1.289517	-2.9693	-1.26541
H	1.872097	-2.86005	-2.83754
H	2.446571	-4.04594	-1.77298
C	3.656175	-2.39775	0.011959
O	2.753678	-2.95222	0.716728
O	4.751044	-1.95967	0.36855
C	2.939761	-0.69316	-1.65176
C	1.952609	-0.11474	-0.69712
C	1.69375	1.352706	-0.70486
C	2.69142	2.143417	0.164238
N	2.314781	3.5828	0.275845
H	1.284338	3.733463	0.140702
H	2.597715	3.974619	1.214509
H	2.808635	4.120684	-0.4663
H	-0.71297	-9.4233	0.077068
H	-10.0237	-0.43538	4.720547
H	-11.1134	1.243612	0.618039
H	-5.80738	6.12488	-3.43007
H	2.189277	11.85217	0.139616
H	-6.33229	-3.32828	6.1303
H	-8.20903	-6.34024	2.465403
H	-8.85644	-0.18096	-3.69437
H	-5.3727	-4.03711	-1.57903
H	5.929195	5.190547	3.620381
H	10.31747	1.523118	1.19266
H	8.362434	1.731808	7.022677
H	-0.15227	-3.32542	6.682685
H	3.149734	-6.7291	4.199258

H	6.982765	1.416222	-2.74391
H	9.871239	-1.83818	-2.34823
H	3.076212	-6.21799	-6.05909
H	-0.63897	-2.32605	-0.31358
H	-0.92429	-3.38521	-1.40851
H	2.719245	1.726404	1.174187
H	3.707784	2.096468	-0.25117
H	0.695712	1.544761	-0.29452
H	1.716169	1.764821	-1.72607
H	-0.27488	-0.42139	-0.32507
H	1.69549	-0.67986	0.202182
H	2.65726	-0.4939	-2.69892
H	3.899928	-0.16472	-1.51641
H	4.138701	-2.4217	-2.10889
H	0.516425	-5.67832	-6.04617
O	-1.33615	1.899477	1.07872
H	-1.40775	1.007541	0.689536
H	-0.50295	1.823953	1.601486
O	-1.27834	-0.40606	3.044526
H	-1.15056	-0.75466	2.140561
H	-1.90454	0.319518	2.896424
O	-0.31384	3.796919	-0.5503
H	0.09032	3.549398	-1.41319
H	-0.79708	3.042508	-0.14097
O	1.899539	-1.4114	2.695826
H	2.341252	-1.94472	1.986526
H	1.7064	-0.50862	2.376307
O	0.866516	1.179231	2.469217
H	0.267938	0.733122	3.098253
H	1.518904	1.763575	2.956463
O	-5.3153	6.540025	-0.1902
H	-4.54357	6.060629	-0.53901
H	-5.01896	7.464699	-0.0405

[5]**Pr**$_{C,C4}$

C	5.468013	3.971367	-2.76835
H	4.993729	3.774045	-1.7913
C	4.638203	3.404493	-3.91569
H	5.153058	3.603414	-4.86747
H	4.608116	2.315219	-3.81773
C	3.200858	3.911647	-3.98118
H	2.771275	4.011818	-2.96974
H	3.148678	4.923763	-4.40921
C	2.270357	2.992449	-4.7728
O	2.610123	1.848704	-5.11576
N	1.054368	3.486297	-5.03312
H	0.676286	4.370325	-4.64748
H	0.393801	2.860224	-5.47956
C	6.889036	3.41539	-2.75258
O	7.396202	2.883785	-3.75465
N	7.565494	3.571621	-1.60666
H	7.085972	3.840227	-0.739
C	8.956427	3.184273	-1.43267
H	9.28905	3.665721	-0.50197
C	9.190073	1.671436	-1.3465
H	9.072126	1.240348	-2.35452
H	10.243	1.509583	-1.05554
C	8.248429	0.956934	-0.37856
H	7.209895	1.054684	-0.72567
H	8.260438	1.441449	0.611058
C	8.591958	-0.52733	-0.2533
H	8.820619	-0.93378	-1.25214
H	9.497937	-0.66397	0.366362
N	7.470851	-1.28935	0.285346
H	6.932459	-0.89994	1.081385
C	7.175604	-2.53948	-0.05873
N	8.062518	-3.32037	-0.70309
H	9.046014	-3.09524	-0.62865
H	7.782588	-4.29439	-0.89753
N	5.940429	-3.01584	0.17257
H	5.250958	-2.39242	0.620932
H	5.788268	-4.02335	0.218126
C	6.727335	-4.13597	-4.91393
H	7.699637	-4.0088	-5.41502
C	6.56768	-3.10538	-3.77321
H	5.71463	-3.43283	-3.15571
H	7.444288	-3.14838	-3.1138
C	6.343562	-1.69219	-4.19426
N	5.130269	-1.26491	-4.7084

C	5.262608	0.044926	-4.92208
H	4.479857	0.7247	-5.26638
N	6.495544	0.473388	-4.57219
H	6.816856	1.464011	-4.49021
C	7.196086	-0.61077	-4.10141
H	8.219873	-0.52457	-3.7492
C	6.569636	-5.50691	-4.28908
O	5.489588	-6.08608	-4.2376
N	7.660711	-5.99285	-3.63304
H	8.539075	-5.49019	-3.67469
C	7.536436	-7.11384	-2.71597
H	7.121872	-7.97147	-3.26951
C	8.896561	-7.48513	-2.13424
H	8.802642	-8.35074	-1.46349
H	9.31028	-6.6485	-1.55029
H	9.601018	-7.74748	-2.93746
C	6.556327	-6.72727	-1.58964
O	6.780294	-5.74693	-0.86943
N	5.491798	-7.52772	-1.44842
H	5.324405	-8.17763	-2.20933
C	4.372756	-7.27081	-0.55181
H	4.73575	-6.66246	0.288842
C	3.20733	-6.55541	-1.25773
H	3.021717	-7.08886	-2.20851
C	1.932716	-6.6291	-0.41411
H	1.627192	-7.67304	-0.23352
H	2.088016	-6.12717	0.553438
H	1.105178	-6.11916	-0.92694
C	3.567162	-5.10562	-1.58126
H	4.480105	-5.05265	-2.18694
H	3.705896	-4.54225	-0.64954
H	2.760872	-4.62922	-2.14757
C	1.44208	-2.09541	5.787534
H	0.374795	-1.82596	5.86379
C	2.284277	-0.81814	5.71207
H	1.848066	-0.17051	4.929805
C	2.270172	-0.08789	7.053087
H	2.894371	0.819082	7.02121
H	2.684426	-0.73645	7.839763
H	1.248648	0.208426	7.339095
O	3.605286	-1.17519	5.334825
H	4.093833	-0.36388	5.046216
C	1.620559	-3.04104	4.622933
O	1.624721	-4.27244	4.810622
N	1.771257	-2.5198	3.39151
H	1.933557	-1.49471	3.24595
C	2.255528	-3.34139	2.298534

H	1.62528	-4.22646	2.149
H	2.203828	-2.7658	1.362107
C	3.709666	-3.79713	2.462794
O	4.12807	-4.757	1.800797
N	4.453129	-3.06439	3.302519
H	4.005153	-2.32788	3.858852
C	5.878429	-3.22851	3.513288
H	6.302949	-3.71949	2.628013
C	6.551589	-1.87847	3.782175
H	6.199648	-1.48565	4.750369
H	7.638469	-2.05446	3.884311
O	6.296991	-0.93715	2.761529
H	5.833696	-0.15753	3.142195
C	-3.72735	-5.81064	5.206952
H	-3.90385	-4.87696	5.761078
C	-2.50321	-5.68739	4.300213
H	-2.28646	-6.6755	3.865313
H	-1.63413	-5.42777	4.930049
C	-2.60738	-4.65586	3.161378
H	-3.31704	-5.05825	2.416104
C	-3.14745	-3.30509	3.642051
H	-4.21801	-3.3653	3.888267
H	-3.01699	-2.52796	2.877741
H	-2.59954	-2.95291	4.532805
C	-1.23938	-4.51137	2.488873
H	-1.2727	-3.82212	1.632719
H	-0.86058	-5.4828	2.13349
H	-0.50521	-4.12172	3.208824
C	-4.98521	-6.23043	4.469718
O	-4.98262	-7.08022	3.578745
N	-6.13607	-5.65859	4.894759
H	-6.08626	-4.88188	5.54181
C	-7.39175	-5.95011	4.259406
H	-7.49354	-7.03925	4.133197
C	-7.51994	-5.29455	2.878229
O	-6.63081	-4.58145	2.419514
N	-8.6543	-5.59306	2.219533
H	-9.3948	-6.06895	2.721048
C	-8.96213	-4.98391	0.941462
H	-8.20037	-5.25535	0.200241
C	-9.05768	-3.45822	1.064889
O	-9.53147	-2.92524	2.05521
N	-8.58392	-2.77997	-0.00881
C	-8.58586	-1.33487	-0.02424
C	-8.34342	-0.86067	-1.45559
C	-7.52491	-0.73326	0.951419
O	-7.82249	-1.59403	-2.30046

C	-6.13221	-1.17227	0.650229
C	-5.07873	-0.51976	0.042706
N	-5.67343	-2.45433	0.891918
C	-4.41277	-2.5529	0.432892
N	-4.01532	-1.39557	-0.08852
H	-8.04136	-3.26406	-0.72622
H	-9.56698	-0.97808	0.325239
H	-7.58871	0.363461	0.926671
H	-7.82486	-1.0562	1.959204
H	-5.04611	0.506047	-0.31571
H	-3.81685	-3.45956	0.475745
H	-6.18429	-3.22531	1.348294
N	-8.68952	0.416323	-1.71371
H	-8.94692	1.05263	-0.94473
C	-8.23612	1.055155	-2.9287
H	-8.35141	0.367578	-3.77685
C	-6.78412	1.510546	-2.77577
O	-6.39227	2.026207	-1.72117
N	-5.94679	1.308892	-3.80721
C	-4.54578	1.704539	-3.70003
C	-4.43871	3.241389	-3.65658
C	-3.72447	1.156411	-4.8639
O	-4.79764	3.921842	-4.60518
C	-2.21888	1.418884	-4.72326
C	-1.49784	0.589305	-3.68301
O	-0.30985	0.243453	-3.88833
O	-2.11961	0.294112	-2.60697
H	-6.30504	0.960945	-4.68962
H	-4.16691	1.287388	-2.76103
H	-3.90585	0.074085	-4.96207
H	-4.0769	1.649168	-5.784
H	-2.02697	2.468409	-4.43594
H	-1.71161	1.257246	-5.68445
N	-3.9348	3.704399	-2.49604
H	-3.64526	3.036879	-1.78502
C	-3.62265	5.08885	-2.20194
H	-3.10352	5.074928	-1.22993
C	-2.69116	5.735677	-3.23444
H	-3.152	5.688368	-4.23275
H	-2.56243	6.801015	-3.00186
C	-1.30561	5.086931	-3.32302
O	-0.42117	5.695098	-3.98359
O	-1.09174	3.979672	-2.74558
C	-4.89645	5.901063	-1.92662
O	-4.8543	7.113768	-1.72019
N	-6.03708	5.18062	-1.85786
H	-5.97603	4.166625	-1.88864

C	-7.34697	5.784436	-1.66748
H	-7.28003	6.800854	-2.08578
C	-8.42283	4.988846	-2.3906
H	-9.38948	5.506882	-2.32871
H	-8.55252	4.002891	-1.92521
H	-8.14597	4.867277	-3.448
C	-7.6414	5.934828	-0.15853
O	-8.52548	5.299148	0.41359
N	-6.82006	6.818071	0.445274
H	-6.02775	7.13374	-0.12026
C	-6.83348	7.128342	1.869391
H	-7.83553	7.519302	2.122009
C	-6.58857	5.903807	2.778365
H	-7.46941	5.25503	2.692733
H	-6.53749	6.288191	3.805654
C	-5.34114	5.120752	2.448764
C	-5.40588	4.004778	1.597914
H	-6.37361	3.693934	1.200811
C	-4.25721	3.272249	1.290633
H	-4.32042	2.404121	0.633636
C	-3.02291	3.639585	1.83549
H	-2.13543	3.045462	1.617495
C	-2.94374	4.753342	2.67449
H	-1.97721	5.054362	3.080827
C	-4.09295	5.487828	2.976778
H	-4.03263	6.361909	3.625838
C	-5.82228	8.25405	2.182288
O	-5.33477	8.354361	3.297592
N	-5.52508	9.097051	1.159677
H	-5.99091	8.976917	0.26918
C	-4.39299	10.01331	1.193328
H	-4.15696	10.19143	2.250292
C	-3.18402	9.457004	0.429346
H	-3.48927	9.265474	-0.61366
H	-2.4063	10.24116	0.392943
C	-2.58536	8.167175	1.011889
H	-3.40218	7.430278	1.102431
C	-1.55264	7.565622	0.057352
H	-1.97698	7.411381	-0.94501
H	-0.67049	8.217747	-0.05141
H	-1.19902	6.597728	0.440896
C	-1.98279	8.376473	2.405068
H	-2.74847	8.663134	3.140688
H	-1.49595	7.454693	2.757639
H	-1.21124	9.166002	2.378052
C	2.23214	6.853531	4.212699
H	1.586291	7.099846	5.069722

C	2.1821	5.354624	3.88785
H	3.067754	5.130168	3.271465
H	2.289663	4.761554	4.806489
C	1.066767	4.73071	3.052907
O	0.345257	5.433855	2.32259
O	1.068352	3.456471	3.07893
C	3.707427	7.1159	4.492065
O	4.477044	7.530308	3.62954
N	4.190158	6.640021	5.675957
H	3.542978	6.358409	6.402389
C	5.618888	6.47801	5.881933
H	6.152854	7.43072	5.754891
C	6.295382	5.494477	4.904378
O	7.493214	5.524184	4.709855
N	5.452985	4.630995	4.261943
H	4.482262	4.610077	4.542123
C	5.878967	3.760707	3.185228
H	6.738855	3.154949	3.512433
C	4.737841	2.820785	2.752722
H	5.093837	2.192535	1.925913
H	3.89116	3.414849	2.376914
C	4.267082	1.910918	3.863649
O	4.946711	0.933018	4.233146
N	3.11815	2.254359	4.465508
H	2.378372	2.770476	3.941277
H	2.766486	1.607727	5.160993
C	6.388494	4.496986	1.92759
O	6.969044	3.822702	1.071858
N	6.148035	5.810865	1.804618
H	5.694624	6.319161	2.565078
C	6.740089	6.606839	0.734874
H	6.901424	5.946399	-0.12706
C	5.882534	7.812257	0.327153
H	5.728536	8.454962	1.209069
H	6.473774	8.40014	-0.39235
C	4.547598	7.443791	-0.2729
C	4.343833	7.353158	-1.65608
H	5.159159	7.607423	-2.34
C	3.110322	6.959133	-2.19009
H	2.966086	6.91077	-3.27124
C	2.053517	6.638601	-1.32995
O	0.849882	6.170627	-1.75068
H	0.659123	6.165756	-2.72426
C	2.227325	6.772533	0.05504
H	1.390191	6.588066	0.727303
C	3.458566	7.165469	0.566756
H	3.582376	7.291556	1.642004

C	-6.46705	-5.81104	-3.31166
C	-6.17659	-5.70314	-1.82034
C	-5.31899	-5.27214	-4.19189
O	-6.64334	-4.78961	-1.13564
C	-4.81765	-3.93721	-3.74778
C	-3.53991	-3.48902	-3.49004
N	-5.64058	-2.87495	-3.41986
C	-4.87273	-1.86506	-2.97182
N	-3.58913	-2.19984	-2.99411
H	-6.67606	-6.85569	-3.58966
H	-5.67119	-5.23651	-5.23719
H	-4.4676	-5.96925	-4.17705
H	-2.60159	-4.01819	-3.63499
H	-5.29682	-0.94074	-2.5984
H	-6.65521	-2.84512	-3.3091
N	-5.36915	-6.64987	-1.30571
H	-4.90692	-7.34074	-1.89661
C	-4.9792	-6.72211	0.081876
H	-5.81293	-7.03668	0.732168
H	-4.66618	-5.73646	0.461079
C	-3.82844	-7.73047	0.180429
O	-3.34027	-8.21344	-0.83788
N	-3.41914	-8.03531	1.428695
H	-3.94017	-7.66711	2.234687
C	-2.4313	-9.07496	1.650739
H	-2.45025	-9.74864	0.7811
C	-1.00534	-8.55123	1.853644
H	-0.7268	-7.91612	0.996819
H	-0.98533	-7.90231	2.744732
C	0.005781	-9.68864	2.012285
H	0.134083	-10.2183	1.053689
H	-0.38381	-10.4449	2.719025
C	1.385397	-9.2344	2.495769
H	2.097904	-10.0737	2.480271
H	1.780774	-8.46043	1.826295
N	1.330783	-8.68569	3.851084
H	0.951748	-9.2975	4.566063
C	1.476222	-7.39269	4.184088
N	2.08481	-6.51993	3.389726
H	2.70626	-6.78182	2.634875
H	2.07581	-5.53839	3.714721
N	1.00191	-6.94906	5.358974
H	0.33021	-7.49072	5.88569
H	1.146141	-5.96188	5.59112
C	2.481877	-4.50655	-4.83638
H	2.068909	-4.77794	-5.82253
H	1.783837	-4.84758	-4.06008

N	2.713358	-3.06891	-4.74127
H	3.627357	-2.6827	-5.007
C	1.729292	-2.15447	-4.68254
N	0.508665	-2.48068	-4.24388
H	0.379439	-3.25606	-3.57687
H	-0.13352	-1.69565	-4.14702
N	1.986168	-0.90378	-5.06989
H	2.86007	-0.67621	-5.52274
H	1.331505	-0.13844	-4.90648
O	-0.01468	-4.34506	-2.17701
C	-1.04108	-4.15822	-1.47581
O	-1.3892	-3.01198	-1.04594
C	-1.89175	-5.34381	-1.07774
H	-1.59968	-6.25679	-1.60776
H	-1.78836	-5.51324	0.003755
O	1.104562	-0.22095	-1.68038
O	1.726637	-2.67947	-0.85478
Fe	-2.38796	-1.30766	-1.40443
C	4.279843	-0.1651	-1.5688
N	3.92372	-1.56343	-1.96221
H	3.017661	-1.94484	-1.54995
H	3.92829	-1.64926	-2.99081
H	4.641977	-2.2023	-1.59482
C	4.48406	-0.07196	-0.04609
O	3.992222	-1.02422	0.638406
O	5.04043	0.95248	0.351068
C	3.250891	0.893585	-1.97821
C	1.925363	0.87311	-1.22517
C	1.128562	2.172972	-1.32648
C	1.428889	3.211544	-0.24515
N	0.234608	4.079148	-0.03788
H	-0.62701	3.508341	-0.09299
H	0.231811	4.58089	0.896982
H	0.199847	4.80496	-0.78526
H	4.035642	-8.24248	-0.1531
H	-8.21464	-5.60443	4.903057
H	-9.93584	-5.36556	0.602133
H	-8.85007	1.949489	-3.11329
H	-4.70442	10.9757	0.757214
H	-3.54363	-6.58976	5.967765
H	-2.73969	-9.65862	2.533967
H	-7.3868	-5.23589	-3.4893
H	-2.95052	-5.13059	-1.27771
H	1.914465	7.459435	3.355418
H	7.734617	6.960872	1.060402
H	5.79539	6.132635	6.910748
H	1.698153	-2.67189	6.688018

H	6.077331	-3.8925	4.374701
H	5.539105	5.069776	-2.83565
H	9.5508	3.594744	-2.26319
H	5.936747	-4.01038	-5.66536
H	1.223735	-1.85333	-0.97956
H	1.188675	-3.35012	-1.33436
H	1.646355	2.721114	0.709606
H	2.278304	3.865579	-0.48887
H	0.080583	1.895638	-1.19615
H	1.17971	2.611334	-2.3319
H	0.651636	-0.00845	-2.54419
H	2.101086	0.652632	-0.16289
H	3.055482	0.877725	-3.06008
H	3.746063	1.847973	-1.75808
H	5.230646	0.058374	-2.07082
H	3.437131	-5.02854	-4.69205
O	-0.97668	-0.34188	-0.02402
H	-0.20136	-0.29804	-0.64106
H	-0.94425	0.513167	0.462145
O	-0.85088	-1.14276	2.647429
H	-0.13452	-1.75318	2.881863
H	-0.88516	-1.1745	1.67066
O	-2.02756	2.466821	-0.88841
H	-1.74	3.056824	-1.65298
H	-2.03446	1.583328	-1.29201
O	2.395755	-0.04768	2.551927
H	3.051993	-0.3309	1.863533
H	1.671541	0.459838	2.129008
O	0.03557	1.335185	1.862314
H	-0.37001	0.697051	2.485231
H	0.22989	2.17941	2.353607
O	-8.44903	2.485194	0.12312
H	-7.60998	2.50244	-0.37475
H	-8.66179	3.426215	0.301607

[5]**TS1**$_{C,C5}$

C	-0.45633	-6.39178	-2.78063
H	-0.28928	-5.86793	-1.82626
C	-0.40166	-5.43812	-3.96934
H	-0.55886	-6.02149	-4.88971
H	-1.23552	-4.72941	-3.91086
C	0.8844	-4.62855	-4.0983
H	1.099344	-4.08559	-3.16323
H	1.758994	-5.26769	-4.28984
C	0.746433	-3.57413	-5.19157
O	-0.31632	-2.96152	-5.34294
N	1.826871	-3.3563	-5.96035
H	2.77063	-3.68282	-5.71826
H	1.752872	-2.63488	-6.66966
C	-1.76943	-7.16809	-2.7248
O	-2.38178	-7.4739	-3.75394
N	-2.18373	-7.53397	-1.49628
H	-1.74818	-7.14122	-0.65515
C	-3.31309	-8.42561	-1.27297
H	-3.21789	-8.79013	-0.23904
C	-4.69558	-7.79426	-1.47304
H	-4.83062	-7.58501	-2.54652
H	-5.44939	-8.55693	-1.2108
C	-4.92307	-6.52692	-0.65211
H	-4.20115	-5.7491	-0.94798
H	-4.7303	-6.71851	0.416581
C	-6.33518	-5.96663	-0.81923
H	-6.57457	-5.85944	-1.8953
H	-7.07041	-6.67727	-0.39832
N	-6.43683	-4.67959	-0.15115
H	-5.54834	-4.1867	0.045298
C	-7.51544	-3.90303	-0.12582
N	-8.70301	-4.33286	-0.64154
H	-8.86111	-5.3312	-0.7083
H	-9.50751	-3.74827	-0.40956
N	-7.42933	-2.6722	0.365594
H	-6.57574	-2.45925	0.912242
H	-8.2738	-2.09539	0.404289
C	-7.27164	-3.04958	-4.52363
H	-7.61346	-4.09556	-4.48953
C	-5.97528	-2.89831	-3.69779
H	-5.71085	-1.83428	-3.64226
H	-6.1162	-3.23736	-2.66135
C	-4.86352	-3.66867	-4.32897
N	-4.22779	-3.22814	-5.47684
C	-3.4058	-4.20567	-5.84108

H	-2.71744	-4.18185	-6.68379
N	-3.49075	-5.25386	-4.98814
H	-2.94083	-6.12189	-4.94789
C	-4.40284	-4.92739	-4.01487
H	-4.63145	-5.60085	-3.19974
C	-8.35645	-2.09951	-4.06982
O	-8.33545	-0.90078	-4.35533
N	-9.33489	-2.64461	-3.30277
H	-9.19862	-3.58384	-2.94258
C	-10.3941	-1.83994	-2.6994
H	-10.6634	-1.07488	-3.44228
C	-11.6127	-2.68809	-2.37351
H	-12.4043	-2.06878	-1.92947
H	-11.374	-3.4802	-1.64836
H	-12.0025	-3.15605	-3.28869
C	-9.80857	-1.12969	-1.46179
O	-9.89196	-1.62863	-0.3319
N	-9.16043	0.005538	-1.76005
H	-9.06005	0.192094	-2.75911
C	-8.39703	0.856093	-0.85944
H	-8.23286	0.335485	0.094524
C	-7.0578	1.258284	-1.5022
H	-7.27617	1.494045	-2.56129
C	-6.48322	2.519739	-0.85523
H	-7.17622	3.373649	-0.9436
H	-6.27607	2.324192	0.20786
H	-5.53315	2.79254	-1.33529
C	-6.0529	0.103387	-1.46366
H	-6.50205	-0.83153	-1.82146
H	-5.70311	-0.06159	-0.43479
H	-5.17946	0.321442	-2.09778
C	-2.62146	1.390972	5.894772
H	-1.73843	2.048913	5.914706
C	-2.15227	-0.06508	5.924
H	-1.40608	-0.19818	5.120527
C	-1.48043	-0.4037	7.250288
H	-1.16766	-1.46015	7.270791
H	-2.18317	-0.25487	8.084341
H	-0.59129	0.224704	7.41306
O	-3.27308	-0.89786	5.677321
H	-2.94553	-1.77728	5.363219
C	-3.45469	1.71257	4.682036
O	-4.51202	2.365873	4.784876
N	-3.05433	1.243974	3.489133
H	-2.2159	0.665372	3.377586
C	-3.94416	1.246612	2.351843
H	-4.23329	2.264208	2.056923

H	-3.41557	0.790218	1.504295
C	-5.21948	0.415687	2.564098
O	-6.20876	0.619539	1.853497
N	-5.13474	-0.51716	3.521622
H	-4.30508	-0.56639	4.124231
C	-6.13906	-1.52689	3.778172
H	-7.04426	-1.26139	3.214198
C	-5.61242	-2.8962	3.346819
H	-4.73245	-3.17656	3.94821
H	-6.39293	-3.65718	3.512684
O	-5.28897	-2.91267	1.973602
H	-4.46714	-2.41356	1.746985
C	-1.58187	8.204385	4.728734
H	-0.82166	7.862899	5.446904
C	-2.4722	7.039956	4.296413
H	-3.35315	7.432452	3.762641
H	-2.8574	6.561576	5.21405
C	-1.80212	5.966355	3.419229
H	-1.7152	6.382335	2.399812
C	-0.38923	5.592568	3.880123
H	0.308929	6.436613	3.786273
H	0.000958	4.775232	3.258104
H	-0.39062	5.240992	4.926168
C	-2.70835	4.736885	3.366704
H	-2.28118	3.939958	2.746172
H	-3.70498	4.982177	2.969833
H	-2.85094	4.325564	4.376612
C	-0.91782	8.93416	3.578662
O	-1.50963	9.227288	2.538411
N	0.368183	9.299348	3.787619
H	0.841702	8.969692	4.61863
C	1.107612	10.06369	2.822991
H	0.510143	10.93291	2.504696
C	1.503581	9.271823	1.569213
O	1.376765	8.054413	1.481364
N	1.99252	10.05767	0.589236
H	2.164019	11.03413	0.795546
C	2.511074	9.513594	-0.64544
H	1.710881	9.02159	-1.20984
C	3.654703	8.528226	-0.39129
O	4.497802	8.723931	0.468047
N	3.649124	7.457293	-1.22248
C	4.688962	6.461573	-1.13933
C	4.750537	5.711471	-2.46592
C	4.495525	5.491019	0.066591
O	3.770325	5.614637	-3.21015
C	3.184847	4.780954	0.070365

C	2.837133	3.476823	-0.21944
N	1.988538	5.418861	0.359431
C	0.988911	4.529172	0.230752
N	1.462909	3.33344	-0.11445
H	2.822783	7.248123	-1.78597
H	5.645248	6.976968	-0.96571
H	5.321343	4.766944	0.084034
H	4.592451	6.114577	0.968405
H	3.490496	2.653896	-0.49946
H	-0.06013	4.764238	0.375838
H	1.857868	6.408854	0.609481
N	5.941831	5.14645	-2.74915
H	6.64562	5.04394	-2.00324
C	6.038542	4.203789	-3.8385
H	5.537424	4.608146	-4.72801
C	5.429275	2.866868	-3.42541
O	5.631859	2.396059	-2.29864
N	4.645921	2.236453	-4.31354
C	3.994823	0.991032	-3.936
C	5.03729	-0.13745	-3.86019
C	2.87164	0.63862	-4.90925
O	5.7495	-0.39945	-4.81644
C	1.8707	-0.36951	-4.32492
C	0.983859	0.234237	-3.25348
O	-0.26542	0.187531	-3.39591
O	1.531579	0.789035	-2.2503
H	4.572672	2.580939	-5.26388
H	3.572388	1.153799	-2.94102
H	2.336207	1.561348	-5.19095
H	3.316735	0.221431	-5.82673
H	2.407032	-1.22861	-3.88879
H	1.224289	-0.7676	-5.11472
N	5.06922	-0.77022	-2.66422
H	4.517344	-0.40562	-1.87601
C	6.008013	-1.8212	-2.33558
H	5.680348	-2.23088	-1.36797
C	6.048404	-2.96665	-3.37358
H	6.706557	-2.7186	-4.21216
H	6.453268	-3.86638	-2.8836
C	4.703863	-3.3155	-3.98647
O	3.626329	-3.32576	-3.1945
O	4.575152	-3.58411	-5.16178
C	7.432074	-1.29245	-2.09245
O	8.355582	-2.0674	-1.84294
N	7.594197	0.044395	-2.16715
H	6.780944	0.651286	-2.24419
C	8.915358	0.656997	-2.21151

H	9.593169	-0.12127	-2.59552
C	8.935463	1.861417	-3.14008
H	9.965579	2.217386	-3.27736
H	8.358202	2.689909	-2.71302
H	8.518641	1.580805	-4.11861
C	9.408055	1.0046	-0.79096
O	9.586419	2.161047	-0.4127
N	9.636911	-0.0869	-0.03341
H	9.308023	-0.97582	-0.4179
C	10.15378	-0.0439	1.329316
H	11.12094	0.490877	1.301048
C	9.260947	0.722927	2.323446
H	9.353022	1.789723	2.085609
H	9.70001	0.553766	3.317071
C	7.798232	0.342482	2.342656
C	6.835547	1.221136	1.819114
H	7.15839	2.149756	1.341638
C	5.470764	0.942257	1.944891
H	4.731945	1.666303	1.591368
C	5.05272	-0.23758	2.567087
H	3.990874	-0.42577	2.719354
C	6.000784	-1.14981	3.040487
H	5.674876	-2.08056	3.510618
C	7.361713	-0.8538	2.938721
H	8.102144	-1.5373	3.35361
C	10.46017	-1.47504	1.820862
O	10.3605	-1.76951	3.001299
N	10.8491	-2.35741	0.863405
H	10.92827	-2.03619	-0.09364
C	10.88414	-3.79551	1.089396
H	10.96561	-3.946	2.173606
C	9.644706	-4.48925	0.510403
H	9.61664	-4.29618	-0.57616
H	9.766805	-5.58059	0.632821
C	8.303597	-4.05162	1.121275
H	8.255571	-2.94963	1.074305
C	7.137302	-4.57156	0.278782
H	7.24647	-4.25102	-0.76831
H	7.083881	-5.67392	0.300404
H	6.184816	-4.18001	0.662082
C	8.15507	-4.46629	2.588508
H	8.915858	-3.98841	3.222865
H	7.164375	-4.18676	2.980343
H	8.25491	-5.56064	2.694777
C	3.402568	-5.82417	5.95251
H	3.638204	-5.99764	7.014647
C	3.07401	-4.33243	5.728386

H	2.058063	-4.09253	6.077455
H	3.778174	-3.73461	6.327525
C	3.278058	-3.89601	4.269649
O	4.394165	-4.05725	3.768976
O	2.269095	-3.37706	3.662835
C	2.260771	-6.73584	5.545103
O	2.192006	-7.29679	4.451488
N	1.249367	-6.84371	6.455926
H	1.382426	-6.42426	7.368315
C	0.122937	-7.73998	6.270366
H	0.464564	-8.76528	6.056866
C	-0.82951	-7.38493	5.124054
O	-1.66791	-8.1769	4.739327
N	-0.63213	-6.16802	4.546525
H	0.069058	-5.54812	4.933777
C	-1.30658	-5.78915	3.327624
H	-2.39473	-5.90325	3.448026
C	-0.96248	-4.33822	2.976518
H	-1.56222	-4.04743	2.104708
H	0.103908	-4.29192	2.725355
C	-1.25834	-3.36913	4.106918
O	-2.42385	-3.0697	4.398122
N	-0.17674	-2.91319	4.754755
H	0.790514	-3.04806	4.377852
H	-0.30275	-2.19094	5.455058
C	-0.93456	-6.66858	2.116567
O	-1.61191	-6.57775	1.089953
N	0.150524	-7.45276	2.243604
H	0.673746	-7.43977	3.122302
C	0.512166	-8.46675	1.271993
H	0.046269	-8.20363	0.312619
C	2.038746	-8.60299	1.117026
H	2.476608	-8.88202	2.08715
H	2.231015	-9.42629	0.410837
C	2.656647	-7.33118	0.622431
C	2.609856	-6.99854	-0.76102
H	2.208278	-7.73026	-1.46648
C	3.046366	-5.77885	-1.21585
H	2.998381	-5.51006	-2.27052
C	3.549549	-4.79461	-0.27655
O	3.848196	-3.61318	-0.63917
H	3.811796	-3.29762	-2.2101
C	3.666084	-5.17696	1.110587
H	4.099699	-4.48311	1.83765
C	3.216278	-6.40488	1.535114
H	3.267565	-6.66599	2.593886
C	-0.19235	7.576547	-4.04867

C	-0.22261	7.543594	-2.5288
C	-0.73976	6.262543	-4.64853
O	0.778123	7.251215	-1.87057
C	-0.18895	5.052974	-3.97071
C	-0.81994	4.053818	-3.26278
N	1.162152	4.775322	-3.86223
C	1.309068	3.661282	-3.1215
N	0.126807	3.193643	-2.73876
H	-0.76737	8.430001	-4.4398
H	-0.52362	6.246037	-5.73
H	-1.83513	6.228078	-4.54966
H	-1.88356	3.905907	-3.09859
H	2.277837	3.258575	-2.84852
H	1.964399	5.373365	-4.06834
N	-1.40798	7.815336	-1.95351
H	-2.25597	7.886731	-2.51279
C	-1.6361	7.763615	-0.52746
H	-1.16213	8.602783	0.005143
H	-1.19827	6.846788	-0.09681
C	-3.14844	7.758806	-0.30638
O	-3.90743	7.423419	-1.21524
N	-3.57131	8.130953	0.916702
H	-2.89304	8.517765	1.5858
C	-4.99492	8.234461	1.187294
H	-5.4868	8.671376	0.303292
C	-5.65877	6.891343	1.508678
H	-5.37409	6.164708	0.732405
H	-5.25814	6.511531	2.463494
C	-7.18254	6.997475	1.57067
H	-7.58018	7.246551	0.572706
H	-7.48327	7.833152	2.229873
C	-7.87323	5.719683	2.05332
H	-8.96618	5.80812	1.958201
H	-7.56606	4.865649	1.434099
N	-7.55035	5.42264	3.45002
H	-7.80759	6.135933	4.124025
C	-6.68188	4.488699	3.874423
N	-6.35386	3.438023	3.128275
H	-6.93353	3.089229	2.37466
H	-5.65646	2.802059	3.552863
N	-6.11627	4.600778	5.084105
H	-6.14974	5.470911	5.597361
H	-5.48624	3.849662	5.389102
C	-5.48232	0.576878	-5.22659
H	-6.41627	0.00377	-5.29422
H	-5.43987	1.309778	-6.04963
N	-4.35438	-0.3362	-5.26042

H	-4.46834	-1.31325	-5.58857
C	-3.10532	-0.00737	-4.92664
N	-2.82123	1.188039	-4.36578
H	-3.53668	1.716955	-3.88177
H	-1.86662	1.334213	-4.04389
N	-2.10665	-0.85345	-5.16373
H	-2.27198	-1.77441	-5.55335
H	-1.20066	-0.68278	-4.7205
O	-3.06144	2.317346	-1.53752
C	-2.57811	3.026045	-0.63391
O	-1.44068	2.803353	-0.07472
C	-3.33001	4.252301	-0.15834
H	-4.31305	3.954331	0.232189
H	-2.78157	4.790758	0.621257
O	-0.06383	0.307348	0.02602
O	-1.27812	-0.59824	2.273671
Fe	0.156223	1.881525	-1.02906
C	-2.6914	-1.70268	-1.66945
N	-2.19583	-0.3234	-1.48377
H	-1.60312	-0.20315	-0.6301
H	-1.5365	-0.07202	-2.25142
H	-2.90327	0.430766	-1.44714
C	-3.4892	-2.20136	-0.44933
O	-3.28986	-1.62276	0.664132
O	-4.22266	-3.17429	-0.67845
C	-1.53774	-2.66918	-1.97484
C	-0.61895	-3.01696	-0.79672
C	0.567402	-2.12557	-0.42921
C	1.109552	-2.61503	0.898924
N	2.420911	-2.11164	1.347518
H	2.409515	-1.0805	1.460356
H	2.579383	-2.57864	2.307514
H	3.172633	-2.35719	0.682306
H	-8.98865	1.762506	-0.63831
H	2.026122	10.44185	3.297061
H	2.908025	10.34337	-1.24792
H	7.09946	4.038529	-4.07634
H	11.7963	-4.20746	0.629241
H	-2.19268	8.955233	5.259613
H	-5.12879	8.9372	2.024292
H	0.858093	7.721547	-4.338
H	-3.51366	4.931039	-1.00469
H	4.290348	-6.08411	5.360945
H	0.098456	-9.44347	1.578792
H	-0.45971	-7.77821	7.201516
H	-3.25072	1.605627	6.769865
H	-6.38554	-1.53719	4.852863

H	0.348305	-7.14431	-2.8591
H	-3.21862	-9.29008	-1.94803
H	-7.05236	-2.81191	-5.57487
H	-0.84835	-0.13331	1.509483
H	-1.98438	-1.1239	1.836699
H	0.388953	-2.35973	1.685779
H	1.186769	-3.71378	0.880429
H	0.265254	-0.93399	-0.28079
H	1.333523	-2.07899	-1.21969
H	-1.23736	-3.17242	0.100799
H	-0.18587	-4.01083	-1.00621
H	-0.9679	-2.31333	-2.84555
H	-2.03431	-3.59427	-2.29556
H	-3.36938	-1.69806	-2.53335
H	-5.52839	1.124588	-4.27161
O	1.999451	0.69138	1.725995
H	1.216941	0.556599	1.133225
H	1.687516	0.414015	2.630224
O	-0.16616	2.521018	2.366677
H	-0.75434	2.679631	1.600264
H	0.697066	2.289348	1.980119
O	3.416048	-0.10716	-0.56271
H	2.661136	0.212479	-1.09958
H	3.364684	0.376488	0.279344
O	0.908023	-0.20753	3.993721
H	0.082428	-0.47282	3.540992
H	0.684392	0.618227	4.48087
O	0.215645	2.277092	4.968848
H	-0.03596	2.502163	4.03843
H	0.985426	2.829296	5.162083
O	7.397577	3.908934	-0.71639
H	6.828717	3.200648	-1.07411
H	8.262919	3.474594	-0.55842

[5]**IM1**$_{C,C5}$

C	6.529424	1.788793	-2.80434
H	6.063876	1.85112	-1.8071
C	5.514245	1.395938	-3.87376
H	6.015638	1.392886	-4.85364
H	5.196277	0.36161	-3.70289
C	4.272262	2.27659	-3.94062
H	3.857156	2.460148	-2.93404
H	4.501829	3.274803	-4.3409
C	3.147672	1.652047	-4.76452
O	3.114995	0.426987	-4.99147
N	2.209689	2.499827	-5.18661
H	2.134855	3.467167	-4.80097
H	1.404005	2.107979	-5.66103
C	7.71561	0.832447	-2.76147
O	8.109513	0.234893	-3.77768
N	8.328521	0.702307	-1.57784
H	7.914314	1.071843	-0.7135
C	9.511446	-0.11904	-1.37541
H	9.952394	0.212123	-0.42438
C	9.227303	-1.62534	-1.31842
H	9.011526	-1.98203	-2.34023
H	10.15573	-2.13211	-1.00134
C	8.069575	-1.99466	-0.3915
H	7.132912	-1.55593	-0.76375
H	8.206748	-1.54874	0.6065
C	7.894286	-3.509	-0.28034
H	7.967629	-3.95813	-1.28442
H	8.705977	-3.94968	0.326703
N	6.590275	-3.8604	0.271517
H	6.228278	-3.32977	1.085652
C	5.814699	-4.83881	-0.18075
N	6.298039	-5.80163	-0.99719
H	7.289971	-5.99917	-0.96446
H	5.651703	-6.57326	-1.21828
N	4.503519	-4.84499	0.10332
H	4.108665	-4.08042	0.66523
H	3.980272	-5.71984	0.065916
C	4.888397	-5.85465	-4.88476
H	5.970085	-6.04137	-4.96319
C	4.619931	-4.65557	-3.95074
H	3.527081	-4.5675	-3.84907
H	5.024579	-4.89134	-2.956
C	5.197155	-3.36736	-4.4347
N	4.610011	-2.63954	-5.45237
C	5.388374	-1.58492	-5.65296

H	5.182518	-0.77336	-6.34606
N	6.455813	-1.60306	-4.82591
H	7.146656	-0.83995	-4.65452
C	6.349544	-2.72159	-4.03361
H	7.08437	-2.95492	-3.26947
C	4.134555	-7.04512	-4.33622
O	2.907099	-7.07199	-4.2983
N	4.877403	-8.03868	-3.78005
H	5.887747	-7.97433	-3.78875
C	4.241987	-9.05984	-2.97129
H	3.47412	-9.56098	-3.58243
C	5.270473	-10.0792	-2.48482
H	4.781019	-10.8648	-1.89228
H	6.024777	-9.594	-1.84575
H	5.775673	-10.5557	-3.33806
C	3.552277	-8.38642	-1.76994
O	4.136488	-7.50891	-1.12611
N	2.336581	-8.85219	-1.45554
H	1.89896	-9.48171	-2.1197
C	1.506165	-8.31719	-0.38667
H	2.153608	-7.75632	0.30041
C	0.382946	-7.40171	-0.90235
H	-0.20885	-7.97779	-1.6397
C	-0.53355	-7.03084	0.268062
H	-1.01232	-7.93073	0.694816
H	0.069066	-6.53183	1.042029
H	-1.33076	-6.34545	-0.05282
C	0.934358	-6.15312	-1.59721
H	1.615701	-6.42421	-2.41656
H	1.467236	-5.5257	-0.8667
H	0.110891	-5.56241	-2.02227
C	0.443962	-2.37223	5.75425
H	-0.41887	-1.68611	5.745946
C	1.733423	-1.54333	5.742119
H	1.633875	-0.77556	4.956295
C	1.940448	-0.87151	7.098289
H	2.873815	-0.28671	7.118347
H	2.023911	-1.6331	7.888611
H	1.106157	-0.19567	7.344354
O	2.83141	-2.38891	5.421471
H	3.585988	-1.83104	5.113558
C	0.277609	-3.33241	4.601348
O	-0.27241	-4.44169	4.785886
N	0.729076	-2.96331	3.400788
H	1.224409	-2.05556	3.273102
C	0.895331	-3.90423	2.317929
H	0.009915	-4.54075	2.200523

H	1.010526	-3.35846	1.37206
C	2.128162	-4.80396	2.442926
O	2.260232	-5.75021	1.658036
N	3.017919	-4.46382	3.3872
H	2.820668	-3.66913	4.006556
C	4.308501	-5.10335	3.555933
H	4.506234	-5.70471	2.658668
C	5.423462	-4.08086	3.79256
H	5.244905	-3.55656	4.745969
H	6.37333	-4.63546	3.906551
O	5.529314	-3.14451	2.742021
H	5.336671	-2.24574	3.085997
C	-5.38808	-3.83395	5.22542
H	-5.34439	-2.85563	5.726928
C	-4.16657	-4.0465	4.327819
H	-4.16604	-5.08905	3.974346
H	-3.25649	-3.93086	4.940085
C	-4.0672	-3.11251	3.110285
H	-4.90071	-3.3664	2.430397
C	-4.21948	-1.63829	3.503733
H	-5.25126	-1.40643	3.805755
H	-3.98075	-0.97714	2.658468
H	-3.53717	-1.37618	4.32714
C	-2.75207	-3.37376	2.373142
H	-2.66696	-2.78232	1.452034
H	-2.65941	-4.43659	2.095061
H	-1.8966	-3.12499	3.015053
C	-6.69919	-3.99226	4.477683
O	-6.89592	-4.91446	3.687705
N	-7.6628	-3.08237	4.765645
H	-7.41943	-2.27974	5.333102
C	-8.92081	-3.07467	4.070647
H	-9.21622	-4.11706	3.875175
C	-8.83928	-2.35602	2.718326
O	-7.75376	-2.09806	2.199716
N	-10.0206	-2.0985	2.129582
H	-10.8678	-2.18266	2.678749
C	-10.0837	-1.41106	0.85487
H	-9.53076	-1.9762	0.093555
C	-9.52483	0.014297	0.966403
O	-9.68642	0.686008	1.972181
N	-8.85863	0.439857	-0.13425
C	-8.26466	1.757396	-0.18304
C	-7.89938	2.063674	-1.63441
C	-7.02145	1.882395	0.754634
O	-7.72097	1.15366	-2.45003
C	-5.94338	0.887085	0.481636

C	-4.66112	1.032832	-0.00271
N	-6.10904	-0.47795	0.670086
C	-4.98811	-1.10646	0.299214
N	-4.07803	-0.22021	-0.10863
H	-8.60051	-0.21865	-0.87243
H	-8.99688	2.495465	0.179049
H	-6.61637	2.900786	0.686835
H	-7.40812	1.752521	1.776693
H	-4.14234	1.944266	-0.28051
H	-4.84639	-2.18132	0.308984
H	-6.92058	-0.97212	1.071433
N	-7.74317	3.363405	-1.94571
H	-7.71356	4.072002	-1.19589
C	-7.04926	3.720413	-3.16346
H	-7.41494	3.109781	-3.99945
C	-5.54239	3.538632	-2.95944
O	-5.03459	3.77645	-1.85774
N	-4.80476	3.085466	-3.99227
C	-3.36881	2.885594	-3.80126
C	-2.65281	4.247558	-3.81826
C	-2.75855	1.921508	-4.81703
O	-2.53637	4.888726	-4.85116
C	-1.37932	1.379078	-4.37033
C	-1.47066	0.48875	-3.15956
O	-1.05148	-0.7018	-3.13346
O	-2.06317	0.929759	-2.11099
H	-5.21119	3.016036	-4.91913
H	-3.26796	2.452558	-2.80468
H	-3.44868	1.079658	-4.98599
H	-2.62992	2.452208	-5.77349
H	-0.71077	2.219027	-4.10662
H	-0.9196	0.805838	-5.18714
N	-2.24329	4.631764	-2.59217
H	-2.31497	3.966078	-1.82946
C	-1.41725	5.784314	-2.29184
H	-1.01702	5.593658	-1.28317
C	-0.23588	5.967246	-3.24986
H	-0.59713	6.205701	-4.26053
H	0.373637	6.819269	-2.9209
C	0.673612	4.740532	-3.38048
O	1.788313	4.930326	-3.95527
O	0.296954	3.619569	-2.9465
C	-2.28073	7.041848	-2.12033
O	-1.79234	8.155433	-1.93397
N	-3.6139	6.810124	-2.12018
H	-3.92057	5.842372	-2.14908
C	-4.61267	7.860058	-2.01252

H	-4.17003	8.75777	-2.47166
C	-5.88623	7.466852	-2.7451
H	-6.61545	8.287678	-2.71851
H	-6.35514	6.600581	-2.25852
H	-5.65527	7.225373	-3.79308
C	-4.87895	8.197318	-0.52799
O	-5.94575	7.937246	0.028163
N	-3.8317	8.800673	0.067683
H	-2.95114	8.76741	-0.45353
C	-3.81907	9.278406	1.445089
H	-4.64998	9.998287	1.556216
C	-4.04139	8.176692	2.503057
H	-5.08758	7.85496	2.42426
H	-3.89837	8.659447	3.479102
C	-3.13001	6.980226	2.373706
C	-3.57722	5.814645	1.729628
H	-4.59362	5.781836	1.330944
C	-2.75506	4.689768	1.632039
H	-3.11957	3.776561	1.160914
C	-1.4634	4.721197	2.166628
H	-0.82283	3.840894	2.112929
C	-0.99778	5.879933	2.790527
H	0.018454	5.905552	3.188194
C	-1.82819	6.99822	2.899859
H	-1.47398	7.900787	3.398123
C	-2.51505	10.05976	1.725804
O	-2.0697	10.13523	2.860203
N	-1.92173	10.64211	0.651579
H	-2.35952	10.55733	-0.257
C	-0.55099	11.1356	0.678878
H	-0.3124	11.36331	1.725815
C	0.430536	10.11856	0.080923
H	0.12036	9.905487	-0.95658
H	1.428417	10.58924	0.022349
C	0.533762	8.788838	0.845448
H	-0.48921	8.396962	0.976054
C	1.296878	7.749206	0.024676
H	0.823859	7.59988	-0.95637
H	2.342274	8.052982	-0.14945
H	1.317396	6.784057	0.55246
C	1.153735	8.956481	2.235934
H	0.531656	9.588709	2.886178
H	1.271426	7.978387	2.727502
H	2.157156	9.411016	2.162613
C	4.698804	5.695406	4.325436
H	4.223975	6.167389	5.199581
C	4.076359	4.327824	4.018751

H	4.783171	3.793556	3.362526
H	4.001596	3.732029	4.939059
C	2.764495	4.160412	3.258324
O	2.307822	5.061951	2.537207
O	2.306203	2.971691	3.34772
C	6.168443	5.366619	4.556195
O	7.009039	5.451871	3.665036
N	6.463048	4.735454	5.729021
H	5.776867	4.723611	6.473812
C	7.714853	4.018261	5.89348
H	8.579558	4.67998	5.740993
C	7.914829	2.849903	4.906271
O	9.02326	2.41803	4.663121
N	6.779981	2.373216	4.311596
H	5.883858	2.712821	4.632945
C	6.809202	1.406445	3.232147
H	7.394628	0.524983	3.538273
C	5.388997	0.949871	2.842591
H	5.474389	0.237897	2.010505
H	4.802938	1.814624	2.494846
C	4.654826	0.260762	3.972248
O	4.899583	-0.91823	4.284
N	3.776934	1.022241	4.650421
H	3.285631	1.805448	4.172099
H	3.231865	0.550939	5.362795
C	7.516587	1.906185	1.954123
O	7.781869	1.068682	1.087138
N	7.776488	3.216056	1.829903
H	7.570561	3.852342	2.601177
C	8.603757	3.742245	0.750358
H	8.503241	3.07092	-0.11203
C	8.233176	5.176087	0.35231
H	8.331638	5.828838	1.234194
H	8.985204	5.513643	-0.37823
C	6.846794	5.309049	-0.22871
C	6.603491	5.209856	-1.60489
H	7.445094	5.08725	-2.29315
C	5.306497	5.281001	-2.12521
H	5.135673	5.217054	-3.2019
C	4.220228	5.456955	-1.25921
O	2.92855	5.487276	-1.68153
H	2.781827	5.359368	-2.65695
C	4.449882	5.597253	0.117124
H	3.616516	5.782356	0.794263
C	5.746379	5.516106	0.615936
H	5.92616	5.638645	1.684013
C	-8.04751	-3.06884	-3.51858

C	-7.76163	-3.11502	-2.0242
C	-6.76299	-2.99503	-4.37753
O	-7.89768	-2.11853	-1.31081
C	-5.75833	-2.02868	-3.84136
C	-4.4431	-2.20905	-3.47257
N	-6.0521	-0.72235	-3.49369
C	-4.96136	-0.17641	-2.92892
N	-3.96383	-1.05095	-2.89028
H	-8.63995	-3.94393	-3.82825
H	-7.05237	-2.73963	-5.41103
H	-6.27965	-3.98221	-4.42746
H	-3.82194	-3.08964	-3.604
H	-4.95459	0.823612	-2.51346
H	-6.95383	-0.23978	-3.44182
N	-7.32057	-4.28719	-1.52718
H	-7.18986	-5.11866	-2.10325
C	-7.01657	-4.48229	-0.13149
H	-7.90329	-4.33648	0.506854
H	-6.27632	-3.74823	0.223394
C	-6.46416	-5.89851	0.040284
O	-6.27308	-6.61151	-0.94059
N	-6.20446	-6.25642	1.313199
H	-6.47307	-5.64022	2.087834
C	-5.78806	-7.59857	1.669272
H	-5.84666	-8.21585	0.761536
C	-4.37931	-7.65285	2.265594
H	-3.64884	-7.32224	1.506692
H	-4.32307	-6.92904	3.094801
C	-4.03403	-9.05249	2.771562
H	-4.06705	-9.77565	1.93891
H	-4.80772	-9.38388	3.487093
C	-2.66454	-9.17758	3.442441
H	-2.49413	-10.2182	3.757789
H	-1.85518	-8.94646	2.734173
N	-2.5046	-8.33024	4.622243
H	-2.98333	-8.62828	5.464408
C	-1.87052	-7.1459	4.67133
N	-1.28147	-6.61701	3.604655
H	-1.29363	-7.06483	2.700013
H	-0.75907	-5.73323	3.759294
N	-1.81989	-6.4592	5.821529
H	-2.12892	-6.87243	6.690604
H	-1.26609	-5.59141	5.826926
C	1.77205	-4.61369	-6.52414
H	2.546283	-4.71273	-7.29598
H	0.794488	-4.63809	-7.03194
N	1.973749	-3.34382	-5.84759

H	2.955893	-2.94892	-5.8443
C	1.10944	-2.84376	-4.97409
N	-0.02653	-3.49161	-4.67293
H	-0.12348	-4.46552	-4.92944
H	-0.6311	-3.18257	-3.91011
N	1.38309	-1.67784	-4.34131
H	2.031462	-0.99303	-4.7735
H	0.580454	-1.24977	-3.86109
O	-1.62098	-3.99154	-2.40171
C	-2.53887	-3.87756	-1.57721
O	-2.79351	-2.77673	-0.95506
C	-3.46294	-5.02315	-1.25183
H	-3.0931	-5.96983	-1.66158
H	-3.607	-5.10211	-0.16519
O	-1.02865	-0.78221	0.016728
O	0.451796	-2.86962	-0.81683
Fe	-2.46722	-0.88001	-1.25744
C	3.878158	-1.58961	-1.40839
N	2.892888	-2.62377	-1.84549
H	1.984694	-2.61743	-1.2827
H	2.618312	-2.46775	-2.82914
H	3.286624	-3.56389	-1.72632
C	4.126172	-1.69604	0.103952
O	3.257761	-2.35462	0.758529
O	5.104342	-1.08065	0.532529
C	3.394795	-0.17868	-1.72623
C	2.076403	0.241792	-1.03223
C	1.868333	1.720166	-1.06546
C	2.399157	2.59745	0.016643
N	1.602045	3.853398	0.161788
H	0.576721	3.650121	0.042354
H	1.76471	4.319401	1.094517
H	1.888779	4.530297	-0.57601
H	1.075339	-9.16578	0.170984
H	-9.69643	-2.61445	4.701549
H	-11.1373	-1.34653	0.547163
H	-7.24242	4.779447	-3.39041
H	-0.5056	12.08181	0.116872
H	-5.38996	-4.59409	6.025707
H	-6.50552	-8.00982	2.400381
H	-8.66786	-2.17681	-3.68253
H	-4.4506	-4.81665	-1.6911
H	4.610404	6.377256	3.471044
H	9.662865	3.711891	1.06316
H	7.771154	3.628691	6.920139
H	0.385063	-2.97966	6.669272
H	4.291624	-5.79963	4.414849

H	6.9312	2.796151	-3.00283
H	10.23111	0.085063	-2.18204
H	4.514598	-5.62843	-5.89321
H	-0.06805	-2.12769	-0.41855
H	-0.16569	-3.28523	-1.45095
H	2.388045	2.075659	0.980325
H	3.447106	2.908385	-0.16159
H	1.501345	2.206483	-1.97557
H	-0.40662	-0.04753	-0.10595
H	2.075303	-0.12111	0.007259
H	3.302111	-0.04887	-2.81011
H	4.207958	0.478156	-1.38774
H	4.809505	-1.78182	-1.95866
H	1.858647	-5.47599	-5.83875
O	-1.94714	1.486379	1.330569
H	-1.89592	0.577207	0.983925
H	-1.03348	1.639489	1.6707
O	-1.15991	-0.41497	3.496519
H	-0.99066	-0.98741	2.730229
H	-1.86303	0.176119	3.176474
O	-0.93883	3.219	-0.55835
H	-0.63094	3.017942	-1.46725
H	-1.39072	2.453005	-0.14871
O	2.236196	-0.83778	2.67566
H	2.70821	-1.35746	1.97298
H	1.791633	-0.06508	2.276114
O	0.580239	1.366054	2.304963
H	0.161237	0.882039	3.044141
H	1.144977	2.088877	2.721201
O	-6.75122	5.228527	-0.14065
H	-5.92987	4.90739	-0.55411
H	-6.62846	6.194809	-0.01476
H	1.236063	-0.26706	-1.53888

[5]**TS2**$_{C,C5}$

C	30.04273	41.48735	60.14723
H	30.28208	40.44714	60.4155
C	31.24888	42.41311	60.28731
H	30.94348	43.42031	59.96978
H	31.51342	42.50676	61.34684
C	32.50411	42.01124	59.52099
H	32.84195	40.99427	59.7831
H	32.33471	41.98835	58.43175
C	33.65288	42.97131	59.83142
O	33.57972	43.76275	60.77657
N	34.73788	42.90557	59.04151
H	34.90295	42.20044	58.31169
H	35.48333	43.56613	59.23381
C	28.85519	41.98762	60.96755
O	28.55317	43.18387	60.98576
N	28.14113	41.04639	61.62172
H	28.51639	40.10483	61.76879
C	26.86	41.32554	62.25679
H	26.34331	40.35795	62.35019
C	26.92663	42.00467	63.62853
H	27.30375	43.03061	63.48924
H	25.89007	42.10816	63.99397
C	27.77093	41.25546	64.6569
H	28.8177	41.19298	64.32007
H	27.42677	40.2122	64.7489
C	27.74185	41.91836	66.03393
H	28.02416	42.98549	65.94779
H	26.71269	41.89295	66.43824
N	28.65519	41.22885	66.93041
H	29.3459	40.59529	66.49576
C	28.96527	41.61301	68.16545
N	28.31805	42.65521	68.76005
H	27.39021	42.89307	68.43091
H	28.50111	42.75891	69.75974
N	29.9481	41.00421	68.8181
H	30.308	40.13186	68.39774
H	30.16042	41.29247	69.77661
C	31.10371	45.40978	66.85314
H	30.06551	45.73714	66.68833
C	31.33808	44.06547	66.12898
H	32.34405	43.69605	66.36986
H	30.63586	43.29396	66.47543
C	31.19577	44.25464	64.65579
N	32.16669	44.89952	63.90966
C	31.66611	45.04141	62.68967

H	32.18749	45.45317	61.83001
N	30.41642	44.52474	62.61774
H	29.82023	44.38608	61.7937
C	30.10391	44.01075	63.85473
H	29.15517	43.53008	64.05011
C	31.42764	45.34307	68.32803
O	32.58158	45.42091	68.75051
N	30.36155	45.16047	69.15122
H	29.46811	44.9185	68.73569
C	30.50645	44.98164	70.5925
H	31.30003	45.67216	70.91413
C	29.21338	45.31293	71.31985
H	29.33938	45.18978	72.40447
H	28.39357	44.64791	71.00982
H	28.92022	46.35242	71.11484
C	30.98471	43.5394	70.85242
O	30.18041	42.61956	71.05479
N	32.31458	43.3969	70.76535
H	32.81218	44.2208	70.42328
C	33.07591	42.16028	70.83991
H	32.38672	41.30415	70.83511
C	34.07807	42.05865	69.67741
H	34.54614	43.05614	69.57164
C	35.1887	41.0561	69.9967
H	35.74218	41.34108	70.90749
H	34.75822	40.05248	70.12483
H	35.9009	41.00567	69.16367
C	33.37223	41.71514	68.3631
H	32.55363	42.41513	68.15274
H	32.95242	40.7014	68.41355
H	34.08032	41.74756	67.52193
C	33.72895	33.52396	68.73231
H	34.63212	33.05217	68.31504
C	32.59384	33.41819	67.71338
H	32.95746	33.83237	66.75574
C	32.19149	31.96752	67.4725
H	31.35898	31.90894	66.75247
H	31.84716	31.50399	68.40962
H	33.0363	31.3859	67.07263
O	31.50391	34.19156	68.18903
H	30.92863	34.43227	67.42229
C	34.04098	34.9492	69.10906
O	34.23589	35.26781	70.30008
N	34.06445	35.87685	68.1385
H	33.89935	35.6475	67.15381
C	34.04577	37.28867	68.4447
H	34.91778	37.5973	69.03611

H	34.07888	37.84336	67.49698
C	32.7717	37.74424	69.17244
O	32.77303	38.80421	69.80825
N	31.71094	36.93895	69.0296
H	31.81151	36.02686	68.56942
C	30.37348	37.27839	69.46734
H	30.445	38.14638	70.13751
C	29.49384	37.5983	68.25664
H	29.3644	36.70185	67.63138
H	28.49874	37.91986	68.60628
O	30.03265	38.65443	67.48955
H	30.80619	38.40002	66.93426
C	40.18657	34.06067	71.03256
H	39.9971	33.08712	70.5566
C	38.99332	34.99851	70.84676
H	39.12492	35.87564	71.49985
H	38.08823	34.47736	71.20571
C	38.74882	35.48967	69.40972
H	39.56518	36.18977	69.15883
C	38.77903	34.36067	68.37422
H	39.78221	33.9194	68.28375
H	38.49697	34.745	67.38465
H	38.05806	33.56462	68.62815
C	37.42761	36.25732	69.3593
H	37.24735	36.68769	68.36674
H	37.41383	37.07686	70.09316
H	36.58358	35.59573	69.59857
C	41.48745	34.64962	70.52332
O	41.82676	35.81277	70.74587
N	42.28744	33.80172	69.83583
H	41.9353	32.88545	69.59095
C	43.54936	34.22894	69.29945
H	44.12974	34.74223	70.08218
C	43.40995	35.17577	68.10009
O	42.335	35.37553	67.5411
N	44.56921	35.75822	67.73702
H	45.42551	35.43914	68.17395
C	44.66546	36.59104	66.55785
H	44.04037	37.48347	66.67199
C	44.25255	35.82395	65.29793
O	44.53887	34.64833	65.1393
N	43.57022	36.56753	64.39177
C	43.10571	35.96024	63.16851
C	42.80771	37.05193	62.14386
C	41.86705	35.03865	63.40043
O	42.5661	38.21645	62.46864
C	40.70256	35.74625	64.00348

C	39.53738	36.23771	63.4519
N	40.65284	36.10765	65.33994
C	39.51588	36.79185	65.5551
N	38.80918	36.89085	64.43106
H	43.18831	37.47739	64.65822
H	43.90605	35.30958	62.78477
H	41.57362	34.57586	62.44831
H	42.21483	34.23362	64.06494
H	39.1991	36.16585	62.4208
H	39.22115	37.21191	66.51104
H	41.36606	35.91358	66.05796
N	42.80199	36.63254	60.86044
H	42.78967	35.6269	60.65871
C	42.29439	37.48344	59.81145
H	42.7163	38.49236	59.91421
C	40.76626	37.53308	59.84058
O	40.089	36.52058	60.04896
N	40.19981	38.73814	59.65805
C	38.75694	38.87816	59.66597
C	38.1667	38.45403	58.31863
C	38.36142	40.32919	59.97494
O	38.68858	38.7803	57.26725
C	36.90668	40.48665	60.43174
C	36.66222	39.98206	61.83717
O	36.10088	40.73451	62.67185
O	37.03457	38.79734	62.12172
H	40.76995	39.5162	59.34536
H	38.38311	38.2181	60.45279
H	39.03633	40.71649	60.75595
H	38.52035	40.9376	59.0704
H	36.23548	39.93031	59.76178
H	36.59586	41.53544	60.37064
N	36.99415	37.76208	58.39208
H	36.73347	37.29552	59.26447
C	36.27145	37.43463	57.17177
H	35.42519	36.79743	57.47121
C	35.75179	38.68098	56.42475
H	36.54595	39.09604	55.79774
H	34.92998	38.37598	55.75835
C	35.27128	39.79891	57.32341
O	34.34665	39.5288	58.24571
O	35.70293	40.93132	57.234
C	37.14119	36.60279	56.20326
O	37.06963	36.75461	54.99518
N	37.93841	35.66969	56.79257
H	38.02519	35.7176	57.80261
C	39.06288	35.07755	56.06965

H	39.57413	35.87808	55.50922
C	40.04479	34.44397	57.05155
H	40.87221	33.99745	56.48476
H	39.54982	33.64737	57.63262
H	40.44695	35.19219	57.74921
C	38.68046	34.05692	54.98343
O	39.54555	33.65176	54.22759
N	37.38082	33.67081	54.94102
H	36.74119	34.0338	55.63803
C	36.84704	32.81549	53.89954
H	37.7155	32.48263	53.31049
C	36.13381	31.57969	54.48102
H	36.86932	31.01218	55.07144
H	35.82114	30.95668	53.63359
C	34.93566	31.93508	55.32991
C	35.07893	32.27123	56.68766
H	36.06989	32.22167	57.14917
C	33.97004	32.63169	57.45935
H	34.07643	32.87133	58.51951
C	32.69513	32.65246	56.88609
H	31.83844	32.90093	57.51171
C	32.54151	32.31753	55.53884
H	31.54778	32.31862	55.08475
C	33.65051	31.97046	54.76548
H	33.53412	31.73136	53.70767
C	35.90804	33.56615	52.9278
O	35.15867	32.9382	52.19117
N	35.98807	34.91546	52.96311
H	36.6167	35.3652	53.62395
C	35.10699	35.78254	52.20519
H	34.56836	35.15065	51.48695
C	34.14153	36.55338	53.11427
H	34.74588	37.1799	53.79208
H	33.5498	37.24603	52.48842
C	33.19077	35.67553	53.9435
H	33.80418	34.95058	54.50761
C	32.42558	36.51958	54.96791
H	33.11301	37.06544	55.63463
H	31.78008	37.26388	54.47023
H	31.78465	35.88635	55.60278
C	32.22907	34.87901	53.05661
H	32.76935	34.16406	52.41955
H	31.51756	34.30389	53.66766
H	31.64526	35.55371	52.40684
C	29.50216	31.87727	60.71078
H	29.1625	30.84065	60.86344
C	30.77796	32.13364	61.53989

H	30.54052	32.27082	62.60501
H	31.41904	31.24006	61.46861
C	31.62928	33.29567	61.00512
O	31.86637	33.32913	59.79586
O	32.07606	34.14563	61.8608
C	28.3805	32.82856	61.08068
O	28.15639	33.88364	60.48639
N	27.66805	32.4864	62.19352
H	27.83485	31.57872	62.61109
C	26.5434	33.27089	62.66886
H	25.80252	33.42567	61.86876
C	26.89051	34.67402	63.18023
O	26.02372	35.50204	63.38364
N	28.21933	34.93015	63.32686
H	28.88054	34.17978	63.16747
C	28.72372	36.25242	63.6111
H	28.20475	36.66737	64.4894
C	30.22987	36.17474	63.88127
H	30.57568	37.15147	64.24442
H	30.73763	35.92549	62.94381
C	30.58088	35.12632	64.92177
O	30.26612	35.27713	66.10878
N	31.20243	34.04089	64.43933
H	31.61114	34.03339	63.47782
H	31.55432	33.35235	65.0955
C	28.46181	37.26839	62.47812
O	28.69132	38.46312	62.68523
N	28.00471	36.77978	61.3092
H	27.89142	35.76889	61.20819
C	27.4053	37.61776	60.28659
H	27.78535	38.63974	60.41063
C	27.68639	37.11396	58.85787
H	27.27842	36.09827	58.7439
H	27.14686	37.77524	58.16171
C	29.15277	37.1134	58.55974
C	29.81694	38.31532	58.17624
H	29.21984	39.21071	57.98681
C	31.18524	38.36984	58.06903
H	31.70642	39.29182	57.80617
C	31.97181	37.19204	58.36959
O	33.24235	37.23152	58.45383
H	33.76064	38.17858	58.30364
C	31.28309	35.96098	58.66161
H	31.85931	35.05197	58.85604
C	29.91367	35.93782	58.76871
H	29.40802	35.01434	59.05909
C	42.85184	40.98124	66.04978

C	42.41505	39.69295	66.7298
C	41.65367	41.80094	65.52524
O	42.37449	38.62591	66.1118
C	40.65564	40.97684	64.78239
C	39.3133	40.77935	65.01692
N	40.95447	40.16685	63.70126
C	39.83128	39.52397	63.32947
N	38.81099	39.86689	64.10874
H	43.44985	41.60466	66.732
H	42.03731	42.62079	64.89466
H	41.12595	42.27679	66.36544
H	38.69224	41.2442	65.77617
H	39.81387	38.78987	62.5311
H	41.86568	39.87995	63.33814
N	42.06264	39.78681	68.02497
H	41.94536	40.69378	68.47417
C	41.60191	38.67013	68.81817
H	42.42949	38.0164	69.13976
H	40.92679	38.02836	68.22929
C	40.86614	39.24062	70.03422
O	40.54745	40.42701	70.06451
N	40.60979	38.37054	71.0304
H	40.99259	37.41795	70.98145
C	39.98248	38.82997	72.25737
H	40.32368	39.85823	72.45343
C	38.45138	38.80915	72.20762
H	38.1158	39.36341	71.3162
H	38.11464	37.76774	72.07803
C	37.81086	39.41233	73.45756
H	38.00803	40.49639	73.49945
H	38.27502	38.98854	74.36767
C	36.29677	39.19725	73.5312
H	35.86664	39.74463	74.38415
H	35.81839	39.58723	72.62295
N	35.95833	37.77919	73.6598
H	36.35998	37.29473	74.4553
C	35.40582	37.01082	72.70562
N	34.73382	37.52142	71.68282
H	34.34853	38.45803	71.6627
H	34.40077	36.83065	70.98582
N	35.52465	35.67477	72.76987
H	36.1976	35.23793	73.38434
H	35.06265	35.11841	72.04384
C	35.01569	44.617	66.47226
H	34.24304	45.26339	66.90572
H	35.99059	45.12704	66.53985
N	34.67544	44.30734	65.09428

H	33.82501	44.72735	64.65068
C	35.36584	43.449	64.34804
N	36.35273	42.69703	64.889
H	36.31369	42.42468	65.86815
H	36.76539	41.99508	64.27908
N	35.13031	43.32611	63.04143
H	34.42888	43.86202	62.52966
H	35.50923	42.49708	62.57695
O	36.28636	40.44538	66.71515
C	37.0566	39.49586	66.98777
O	37.06902	38.38221	66.35658
C	38.08404	39.65689	68.08871
H	37.97092	38.858	68.8329
H	39.08678	39.55016	67.65116
O	35.41036	37.56218	64.02808
O	33.45339	36.31341	65.37015
Fe	37.26667	38.33512	64.23343
C	33.09824	40.64956	64.62857
N	34.41479	39.98965	64.75547
H	34.37553	38.95587	64.67644
H	35.05819	40.28575	63.98851
H	34.94129	40.17985	65.64566
C	32.00775	39.93868	65.46021
O	32.23218	38.74963	65.84651
O	30.98113	40.61111	65.62744
C	32.65083	40.77006	63.16478
C	32.2182	39.46948	62.47056
C	33.24829	38.52621	61.9261
C	32.82185	37.09794	61.8881
N	33.60794	36.14405	61.07741
H	34.62926	36.16301	61.27476
H	33.14511	35.18293	61.27901
H	33.54062	36.36023	60.06612
H	33.62021	42.13784	71.80136
H	44.12141	33.34445	68.98003
H	45.71231	36.90634	66.4413
H	42.60572	37.07361	58.83845
H	35.71173	36.49943	51.62531
H	40.33391	33.85802	72.10748
H	40.34232	38.19402	73.08175
H	43.50361	40.68355	65.21582
H	38.02424	40.63129	68.58485
H	29.73733	32.00626	59.64629
H	26.31188	37.65499	60.43644
H	26.04073	32.72261	63.47797
H	33.45332	33.01009	69.66395
H	29.94528	36.43376	70.0317

H	29.70212	41.46342	59.0965
H	26.27133	41.96051	61.57771
H	31.76008	46.17176	66.40898
H	34.24928	36.73065	64.94791
H	32.86532	37.08337	65.5303
H	32.79273	36.69569	62.91471
H	31.78339	37.02966	61.5141
H	35.0634	37.8277	63.16175
H	34.02101	38.89799	61.24405
H	31.51571	38.92447	63.12106
H	31.59326	39.76809	61.60745
H	33.42496	41.2882	62.58099
H	31.78245	41.44185	63.19478
H	33.20153	41.66702	65.02916
H	35.04963	43.71192	67.10014
O	36.22194	35.10415	63.28549
H	35.85125	35.97947	63.56364
H	35.51276	34.46126	63.54602
O	36.57775	35.55154	66.08582
H	36.68539	36.49553	66.29952
H	36.76169	35.45856	65.1304
O	36.39714	36.45312	60.9686
H	36.66534	37.29172	61.40827
H	36.56711	35.75586	61.64866
O	34.21237	33.7176	64.48459
H	33.79589	34.54035	64.80728
H	34.74334	33.38501	65.24244
O	35.91757	33.05825	66.59312
H	36.22479	33.9988	66.59209
H	36.68032	32.54642	66.29179
O	41.63554	34.25245	60.15281
H	40.89165	34.88441	60.05161
H	41.47852	33.52699	59.53567

[5]**Pr**$_{C,C5}$

C	7.331513	1.274535	-2.8222
H	6.811787	1.597536	-1.90707
C	6.342839	0.769653	-3.8662
H	6.889781	0.47797	-4.77312
H	5.861128	-0.14465	-3.49389
C	5.267046	1.786022	-4.22207
H	4.820832	2.220165	-3.31321
H	5.699015	2.64062	-4.77191
C	4.146567	1.199019	-5.06855
O	4.256301	0.099607	-5.62799
N	3.032557	1.943244	-5.13382
H	2.902072	2.823814	-4.60983
H	2.268086	1.625904	-5.71813
C	8.400793	0.245365	-2.48019
O	8.949855	-0.44164	-3.34997
N	8.722307	0.174256	-1.17649
H	8.099374	0.605585	-0.48106
C	9.78069	-0.67384	-0.65745
H	10.00656	-0.30386	0.354376
C	9.432809	-2.16663	-0.59354
H	9.502098	-2.59079	-1.60802
H	10.21156	-2.66603	0.008905
C	8.0486	-2.45167	-0.01166
H	7.272131	-2.14686	-0.73154
H	7.871077	-1.8488	0.894847
C	7.853911	-3.93075	0.309656
H	8.132197	-4.55114	-0.56086
H	8.529979	-4.22253	1.136732
N	6.465587	-4.19226	0.647236
H	5.848588	-3.4079	0.930507
C	5.885705	-5.38961	0.656798
N	6.61768	-6.5281	0.593044
H	7.566603	-6.51511	0.947207
H	6.079763	-7.39895	0.674856
N	4.557899	-5.46284	0.69873
H	4.034372	-4.65488	0.324346
H	4.121714	-6.37744	0.7972
C	5.533077	-6.47698	-3.86437
H	6.596255	-6.69613	-4.05163
C	5.418191	-5.22094	-2.9731
H	4.371631	-5.04395	-2.68554
H	5.965828	-5.38943	-2.03503
C	5.964875	-4.00426	-3.64082
N	5.278715	-3.32385	-4.63109
C	6.077243	-2.34335	-5.03375

H	5.812418	-1.57062	-5.75162
N	7.248832	-2.36485	-4.35761
H	7.972752	-1.62985	-4.32743
C	7.194384	-3.40888	-3.46412
H	8.017794	-3.6348	-2.79537
C	4.844503	-7.66283	-3.22443
O	3.691938	-7.99391	-3.48852
N	5.57744	-8.31022	-2.27313
H	6.485307	-7.94338	-2.01097
C	5.021552	-9.40603	-1.49292
H	4.466057	-10.0498	-2.19144
C	6.124884	-10.211	-0.82134
H	5.699752	-11.0485	-0.25052
H	6.699279	-9.5889	-0.11867
H	6.81089	-10.6172	-1.57854
C	4.027434	-8.8309	-0.46782
O	4.419393	-8.3287	0.594872
N	2.740249	-8.89375	-0.83939
H	2.582094	-9.12201	-1.81924
C	1.623162	-8.34591	-0.08925
H	2.018326	-7.94081	0.854023
C	0.867876	-7.25745	-0.86601
H	0.596313	-7.68435	-1.85029
C	-0.42448	-6.8937	-0.13327
H	-1.08534	-7.76646	-0.00888
H	-0.18905	-6.47117	0.855736
H	-0.97593	-6.12559	-0.68981
C	1.732961	-6.01865	-1.08983
H	2.664839	-6.26619	-1.61497
H	1.972921	-5.55104	-0.12612
H	1.188161	-5.27284	-1.68274
C	-0.72571	-1.35796	5.403336
H	-1.61454	-0.77535	5.113134
C	0.511801	-0.45575	5.34819
H	0.481557	0.102462	4.39217
C	0.510963	0.553658	6.492321
H	1.410162	1.188823	6.45286
H	0.521827	0.030319	7.460609
H	-0.37437	1.207507	6.450272
O	1.657121	-1.28679	5.384909
H	2.440674	-0.73747	5.130433
C	-0.61691	-2.54945	4.486661
O	-0.96453	-3.68853	4.856212
N	-0.13136	-2.32776	3.254942
H	0.229818	-1.40209	3.000555
C	0.209456	-3.39691	2.3485
H	-0.6129	-4.11473	2.252147

H	0.390061	-2.9685	1.353003
C	1.494209	-4.1561	2.698478
O	1.670241	-5.28963	2.237255
N	2.372754	-3.48546	3.459174
H	2.071267	-2.62263	3.926261
C	3.730302	-3.93496	3.718137
H	3.888229	-4.85323	3.138487
C	4.745723	-2.85769	3.334733
H	4.588293	-1.9546	3.946425
H	5.756818	-3.23807	3.555296
O	4.722644	-2.51786	1.959611
H	3.807427	-2.36691	1.62913
C	-5.67472	-4.13651	4.849702
H	-5.72603	-3.21826	5.453675
C	-4.47878	-4.09971	3.8938
H	-4.35103	-5.09766	3.450264
H	-3.56371	-3.90171	4.476177
C	-4.5784	-3.07439	2.753524
H	-5.42009	-3.37777	2.108458
C	-4.87698	-1.66238	3.264994
H	-5.89961	-1.58023	3.659007
H	-4.79288	-0.92857	2.451887
H	-4.16465	-1.36986	4.053371
C	-3.29907	-3.10874	1.915418
H	-3.39489	-2.51666	0.996562
H	-3.04022	-4.14141	1.63052
H	-2.4556	-2.68912	2.479492
C	-6.99032	-4.36919	4.127502
O	-7.12754	-5.24837	3.276071
N	-8.01632	-3.56675	4.502839
H	-7.8154	-2.7966	5.129107
C	-9.28025	-3.56455	3.816193
H	-9.44957	-4.5756	3.41503
C	-9.28586	-2.58741	2.633417
O	-8.23435	-2.25006	2.094149
N	-10.5011	-2.18733	2.218933
H	-11.3084	-2.40077	2.791592
C	-10.6659	-1.30221	1.081269
H	-10.3309	-1.80473	0.163741
C	-9.90324	0.014731	1.288101
O	-9.88719	0.588356	2.363387
N	-9.27146	0.463776	0.175049
C	-8.48197	1.677615	0.155381
C	-8.12129	1.949736	-1.31667
C	-7.23633	1.568449	1.08867
O	-8.27379	1.066329	-2.16668
C	-6.2641	0.531078	0.63839

C	-4.97079	0.66483	0.186393
N	-6.59192	-0.79495	0.405559
C	-5.55429	-1.39637	-0.19366
N	-4.54742	-0.53247	-0.3584
H	-9.22886	-0.10658	-0.6672
H	-9.08577	2.515898	0.538913
H	-6.72881	2.542469	1.131857
H	-7.63579	1.353758	2.090919
H	-4.35769	1.556697	0.176188
H	-5.56121	-2.43161	-0.52091
H	-7.43722	-1.28592	0.734143
N	-7.6558	3.180409	-1.61237
H	-7.31071	3.7976	-0.8566
C	-7.10615	3.440358	-2.92253
H	-7.68621	2.888515	-3.6775
C	-5.65212	2.969549	-2.99043
O	-5.20635	2.297601	-2.0506
N	-4.90008	3.232321	-4.07201
C	-3.49352	2.831438	-4.06427
C	-2.64182	4.091402	-3.89341
C	-3.0806	1.969927	-5.25812
O	-2.77206	5.057174	-4.63115
C	-1.6508	1.426381	-5.12539
C	-1.35183	0.48926	-3.96712
O	-0.35456	-0.26131	-4.03993
O	-2.10573	0.531921	-2.92652
H	-5.20876	3.899464	-4.77427
H	-3.3846	2.214804	-3.17575
H	-3.79846	1.137861	-5.3448
H	-3.14445	2.561551	-6.18544
H	-0.92512	2.248768	-4.99265
H	-1.35315	0.905606	-6.04745
N	-1.83124	4.04613	-2.81155
H	-1.65351	3.143692	-2.38949
C	-0.95306	5.139056	-2.42978
H	-0.5613	4.87512	-1.43375
C	0.220557	5.346375	-3.39555
H	-0.18472	5.594433	-4.39006
H	0.818274	6.208838	-3.07638
C	1.108084	4.110188	-3.53273
O	2.35056	4.275546	-3.68881
O	0.563929	2.967392	-3.48905
C	-1.78275	6.427292	-2.2179
O	-1.32584	7.542875	-2.42078
N	-3.04395	6.199876	-1.7781
H	-3.29346	5.268316	-1.44555
C	-4.09125	7.197198	-1.88766

H	-3.66495	8.011417	-2.49183
C	-5.31941	6.628728	-2.58313
H	-6.09777	7.397865	-2.6884
H	-5.74044	5.809073	-1.98658
H	-5.02971	6.263692	-3.57832
C	-4.42102	7.783464	-0.50518
O	-5.45882	7.523574	0.102785
N	-3.45753	8.612408	-0.0548
H	-2.56824	8.566433	-0.54973
C	-3.50231	9.346453	1.204768
H	-4.41563	9.967826	1.195025
C	-3.57907	8.442225	2.452506
H	-4.59428	8.027559	2.494879
H	-3.43342	9.099305	3.321004
C	-2.56972	7.319861	2.463056
C	-2.95247	6.016876	2.10912
H	-3.99218	5.818635	1.838878
C	-2.02398	4.971625	2.129238
H	-2.35202	3.957119	1.893807
C	-0.69217	5.218708	2.473044
H	0.023019	4.399936	2.525416
C	-0.29435	6.518017	2.803252
H	0.748295	6.718933	3.054884
C	-1.22901	7.554341	2.812518
H	-0.92508	8.562636	3.094032
C	-2.29749	10.31415	1.277475
O	-1.80553	10.62299	2.349348
N	-1.84033	10.77148	0.081126
H	-2.33722	10.50704	-0.76017
C	-0.52754	11.38047	-0.09006
H	-0.23539	11.79513	0.883374
C	0.50135	10.36287	-0.60194
H	0.137991	9.94885	-1.55956
H	1.439822	10.89952	-0.8293
C	0.795613	9.203338	0.363948
H	-0.17076	8.753566	0.652209
C	1.594783	8.097314	-0.32777
H	1.066037	7.725687	-1.21862
H	2.587476	8.453459	-0.6498
H	1.757542	7.258117	0.365225
C	1.494917	9.672521	1.644078
H	0.853858	10.34117	2.237548
H	1.775696	8.8147	2.272571
H	2.425497	10.21357	1.399372
C	5.386649	5.930213	4.531054
H	4.969463	6.355178	5.45662
C	4.421003	4.921761	3.89345

H	4.996046	4.288218	3.201024
H	3.995083	4.249616	4.65596
C	3.267697	5.439118	3.030678
O	3.104928	6.631248	2.820656
O	2.571395	4.462041	2.53328
C	6.654889	5.130275	4.761753
O	7.523443	5.014586	3.899844
N	6.667741	4.344988	5.878608
H	5.964961	4.492378	6.593004
C	7.618166	3.259273	6.026818
H	8.651354	3.619635	5.915217
C	7.464464	2.123908	4.996194
O	8.334354	1.290327	4.842976
N	6.316031	2.150171	4.256183
H	5.634116	2.869635	4.451857
C	6.121017	1.306467	3.099318
H	6.358741	0.26526	3.361882
C	4.675964	1.396567	2.592411
H	4.571551	0.645382	1.796311
H	4.499015	2.387386	2.154581
C	3.620309	1.098718	3.64087
O	3.655572	0.042473	4.285997
N	2.671911	2.032577	3.793428
H	2.649907	2.941985	3.276609
H	1.918713	1.852103	4.447609
C	7.033525	1.656209	1.90376
O	7.043129	0.880676	0.937981
N	7.715057	2.804357	1.96474
H	7.636306	3.400758	2.791437
C	8.632244	3.26806	0.935892
H	8.521929	2.613623	0.062787
C	8.381249	4.732424	0.551851
H	8.508046	5.361201	1.445631
H	9.169805	5.024844	-0.16101
C	7.01814	4.975813	-0.05126
C	6.713854	4.556666	-1.35403
H	7.487842	4.078969	-1.95886
C	5.446601	4.753648	-1.90873
H	5.229323	4.437714	-2.93025
C	4.454102	5.395609	-1.15613
O	3.186348	5.580837	-1.62421
H	3.042527	5.215269	-2.54108
C	4.744356	5.847658	0.13608
H	3.988751	6.391986	0.702273
C	6.010729	5.62524	0.677433
H	6.235358	5.980084	1.68461
C	-8.81623	-3.53284	-3.16806

C	-8.33994	-3.50666	-1.71816
C	-7.67254	-3.46303	-4.20098
O	-8.61712	-2.57114	-0.96718
C	-6.67855	-2.39796	-3.87494
C	-5.30508	-2.42971	-3.77827
N	-7.03443	-1.11684	-3.48739
C	-5.92398	-0.43693	-3.1672
N	-4.84627	-1.20078	-3.32944
H	-9.41388	-4.44042	-3.34854
H	-8.111	-3.30219	-5.20048
H	-7.13315	-4.42058	-4.25764
H	-4.63632	-3.2563	-4.00177
H	-5.93466	0.560885	-2.74363
H	-7.95796	-0.72181	-3.31438
N	-7.59915	-4.56442	-1.32906
H	-7.27382	-5.25035	-2.00717
C	-7.12553	-4.79699	0.016283
H	-7.92998	-5.15199	0.68161
H	-6.76495	-3.86918	0.484202
C	-6.01246	-5.85113	-0.05418
O	-5.54614	-6.1814	-1.14221
N	-5.64006	-6.38418	1.130002
H	-6.13057	-6.06682	1.975382
C	-4.83844	-7.59478	1.200067
H	-4.59327	-7.87753	0.166771
C	-3.55817	-7.46743	2.028215
H	-2.87454	-6.74597	1.551494
H	-3.80792	-7.05098	3.018144
C	-2.87195	-8.82485	2.206314
H	-2.66465	-9.27653	1.221111
H	-3.5654	-9.52018	2.712697
C	-1.55093	-8.78066	2.980286
H	-1.14477	-9.79538	3.104902
H	-0.80204	-8.21284	2.415327
N	-1.68654	-8.17714	4.30682
H	-2.22986	-8.69441	4.989349
C	-1.44325	-6.88294	4.57903
N	-0.66228	-6.13523	3.815991
H	0.030651	-6.49147	3.163321
H	-0.61694	-5.13022	4.07764
N	-2.00113	-6.3047	5.656953
H	-2.76231	-6.74213	6.157744
H	-1.76161	-5.33241	5.857461
C	2.154821	-5.07477	-4.79616
H	3.025499	-5.71613	-4.96325
H	1.367944	-5.37161	-5.50706
N	2.535844	-3.68682	-4.99117

H	3.572988	-3.46963	-4.97643
C	1.639449	-2.70795	-4.98755
N	0.340422	-2.9833	-4.70569
H	0.140273	-3.76586	-4.09077
H	-0.2598	-2.17095	-4.57623
N	1.962124	-1.44274	-5.27093
H	2.902339	-1.11358	-5.53707
H	1.250132	-0.73539	-5.09148
O	-0.56203	-3.63501	-1.87327
C	-1.74919	-3.35337	-1.66164
O	-2.11121	-2.15677	-1.31842
C	-2.83446	-4.4012	-1.72941
H	-2.99719	-4.79179	-0.71301
H	-3.79387	-3.98371	-2.05774
O	1.164354	1.529934	-1.48481
O	1.087789	-0.22627	1.808268
Fe	-3.30106	-0.71993	-2.01191
C	2.408489	-1.61489	-1.88102
N	0.95896	-1.28977	-1.75675
H	0.786029	-0.65599	-0.96981
H	0.568898	-0.80503	-2.59759
H	0.393761	-2.16875	-1.65119
C	2.877333	-2.41656	-0.64935
O	2.439528	-2.04889	0.487204
O	3.669701	-3.34308	-0.8629
C	3.276773	-0.37709	-2.14355
C	3.393565	0.654473	-1.00599
C	2.509759	1.899003	-1.16563
C	2.563946	2.703205	0.127462
N	1.802117	3.974056	0.162608
H	0.793366	3.832412	0.059472
H	2.00433	4.391197	1.170551
H	2.151081	4.654945	-0.55253
H	0.931298	-9.16937	0.164268
H	-10.0948	-3.33699	4.520715
H	-11.7347	-1.06301	0.987031
H	-7.1729	4.511989	-3.15692
H	-0.61676	12.22222	-0.79474
H	-5.55067	-4.97131	5.561076
H	-5.45913	-8.40629	1.621757
H	-9.49319	-2.67498	-3.28286
H	-2.54668	-5.24305	-2.37039
H	5.591046	6.75584	3.838461
H	9.668272	3.155984	1.29987
H	7.525652	2.831212	7.034958
H	-0.87215	-1.75223	6.418442
H	3.853982	-4.17224	4.78939

H	7.864177	2.161527	-3.20856
H	10.68236	-0.53867	-1.27373
H	5.061445	-6.29559	-4.83879
H	0.274763	-0.30131	1.259071
H	1.727105	-0.82912	1.352002
H	2.200382	2.079946	0.954165
H	3.606735	2.970038	0.336068
H	0.949293	1.966888	-2.37184
H	2.889105	2.51948	-1.99343
H	3.1612	0.173765	-0.04536
H	4.439265	0.987942	-0.91991
H	2.917055	0.103134	-3.06466
H	4.27069	-0.78167	-2.37773
H	2.506862	-2.28632	-2.73862
H	1.804864	-5.2748	-3.77057
O	-1.05458	2.273138	-0.14633
H	-0.23711	2.046228	-0.65999
H	-0.73939	2.303214	0.805275
O	-1.24593	-0.36174	0.471582
H	-1.5091	-1.07936	-0.14994
H	-1.34471	0.522191	0.037718
O	-3.60324	3.622398	-0.35202
H	-4.02889	3.054816	-1.02635
H	-2.69156	3.285188	-0.24964
O	-0.16488	2.089988	2.361788
H	0.528696	1.399296	2.318434
H	-0.9336	1.626485	2.763521
O	-2.23426	0.365927	2.924196
H	-2.07779	-0.05827	2.051377
H	-3.15783	0.651445	2.906959
O	-6.15482	4.779484	0.183231
H	-5.22936	4.469599	0.190633
H	-6.08638	5.75957	0.225685

$^{5}\mathbf{Re}_{C2}$

C	-0.10808	-6.5304	-2.8828
H	0.137805	-5.97433	-1.96546
C	-0.23678	-5.59933	-4.08164
H	-0.43524	-6.20251	-4.98065
H	-1.11629	-4.96575	-3.9366
C	0.957839	-4.682	-4.31666
H	1.207985	-4.12307	-3.39877
H	1.866681	-5.24392	-4.58065
C	0.650864	-3.64252	-5.38995
O	-0.48865	-3.18862	-5.53784
N	1.690876	-3.24849	-6.14867
H	2.669574	-3.41825	-5.88847
H	1.507046	-2.54042	-6.85131
C	-1.36779	-7.36484	-2.6754
O	-2.02525	-7.78282	-3.63498
N	-1.68435	-7.65021	-1.39809
H	-1.26281	-7.13733	-0.61698
C	-2.72661	-8.5983	-1.0331
H	-2.55402	-8.8461	0.02523
C	-4.16635	-8.11106	-1.21321
H	-4.39571	-8.07463	-2.28934
H	-4.82183	-8.88789	-0.78095
C	-4.46928	-6.7578	-0.5692
H	-4.0656	-5.93833	-1.18543
H	-3.97656	-6.67335	0.414085
C	-5.9728	-6.53839	-0.40606
H	-6.48603	-6.70927	-1.36769
H	-6.37878	-7.2809	0.308141
N	-6.24152	-5.18331	0.032013
H	-5.52339	-4.67604	0.587236
C	-7.33106	-4.47751	-0.24364
N	-8.46355	-5.07163	-0.69773
H	-8.63925	-6.0329	-0.43199
H	-9.2734	-4.45096	-0.80209
N	-7.30784	-3.15511	-0.10104
H	-6.40408	-2.66932	-0.10278
H	-8.19099	-2.64962	-0.14156
C	-7.5458	-3.12174	-4.71621
H	-7.91428	-3.91743	-5.38381
C	-6.72631	-3.74287	-3.56408
H	-6.48816	-2.97019	-2.81547
H	-7.33135	-4.49775	-3.04218
C	-5.44888	-4.36764	-4.01784
N	-4.43602	-3.61602	-4.58056
C	-3.4385	-4.45457	-4.83135

H	-2.46796	-4.18661	-5.25119
N	-3.7647	-5.71756	-4.46364
H	-3.13463	-6.53305	-4.43661
C	-5.04054	-5.68183	-3.94635
H	-5.53707	-6.57258	-3.5756
C	-8.69466	-2.29776	-4.17108
O	-8.66047	-1.07308	-4.09236
N	-9.75607	-3.00819	-3.69558
H	-9.72596	-4.0209	-3.71237
C	-10.8404	-2.35747	-2.97192
H	-11.1456	-1.47592	-3.55593
C	-12.0203	-3.3037	-2.80036
H	-12.8481	-2.79608	-2.28516
H	-11.7338	-4.17503	-2.19197
H	-12.3803	-3.64922	-3.78051
C	-10.2947	-1.89107	-1.61114
O	-10.0481	-2.70922	-0.71314
N	-10.0704	-0.57955	-1.48763
H	-10.1125	-0.03693	-2.34435
C	-9.43757	0.033092	-0.32237
H	-8.51635	-0.51117	-0.05902
C	-9.07915	1.496158	-0.59826
H	-10.0033	2.012385	-0.92253
C	-8.58131	2.166012	0.685992
H	-9.34895	2.152339	1.476474
H	-7.69353	1.635383	1.065511
H	-8.29971	3.210591	0.488802
C	-8.0309	1.611012	-1.71142
H	-8.33827	1.111026	-2.64257
H	-7.09135	1.145696	-1.37638
H	-7.83299	2.667593	-1.94499
C	-3.01578	1.510569	4.932677
H	-2.10243	2.121229	4.870892
C	-2.61765	0.072017	5.275278
H	-1.83312	-0.24576	4.564747
C	-2.05128	-0.01653	6.688551
H	-1.80974	-1.06217	6.938456
H	-2.79816	0.325461	7.421887
H	-1.13957	0.590231	6.77755
O	-3.76074	-0.75407	5.126556
H	-3.43905	-1.68776	5.103455
C	-3.8054	1.648366	3.650617
O	-4.7884	2.41345	3.57889
N	-3.42359	0.909141	2.601082
H	-2.6074	0.283054	2.66233
C	-4.26771	0.735788	1.442142
H	-4.76178	1.677007	1.178527

H	-3.64771	0.441849	0.591093
C	-5.3613	-0.33187	1.574008
O	-6.14999	-0.47883	0.637394
N	-5.36962	-1.04063	2.715338
H	-4.68611	-0.83439	3.452535
C	-6.26531	-2.1552	2.967615
H	-7.0082	-2.17812	2.162289
C	-5.51775	-3.48935	3.057144
H	-4.84412	-3.48993	3.927454
H	-6.25936	-4.29226	3.206279
O	-4.78606	-3.80755	1.890014
H	-4.20873	-3.06048	1.59357
C	-1.89052	7.930853	4.964299
H	-1.42859	7.51723	5.873061
C	-2.55363	6.833419	4.124327
H	-3.02329	7.311938	3.252736
H	-3.37207	6.368743	4.70169
C	-1.59869	5.739027	3.626167
H	-0.74099	6.227414	3.12799
C	-1.04993	4.887989	4.774109
H	-0.44388	5.479249	5.479197
H	-0.41774	4.077286	4.390022
H	-1.87052	4.427946	5.349905
C	-2.31101	4.881343	2.580199
H	-1.65776	4.08307	2.20493
H	-2.62535	5.499904	1.724765
H	-3.21583	4.411517	2.998376
C	-0.87553	8.682161	4.129831
O	-1.19659	9.318628	3.122328
N	0.415273	8.609049	4.519413
H	0.662476	8.035172	5.314716
C	1.454295	9.272578	3.772829
H	1.302743	10.36397	3.794009
C	1.549454	8.796499	2.321127
O	1.296216	7.639935	1.991798
N	1.973034	9.741611	1.457937
H	2.292236	10.62634	1.83346
C	2.356164	9.386715	0.109739
H	1.509123	8.930665	-0.41409
C	3.539812	8.4139	0.119625
O	4.419857	8.489818	0.962107
N	3.514669	7.490235	-0.8711
C	4.552732	6.489834	-0.94795
C	4.560655	5.886925	-2.34713
C	4.384972	5.381874	0.140033
O	3.538346	5.819071	-3.03577
C	3.073486	4.676069	0.077664

C	2.725701	3.422603	-0.37699
N	1.877782	5.276021	0.445754
C	0.871377	4.424709	0.200137
N	1.34897	3.282924	-0.29555
H	2.66307	7.344041	-1.419
H	5.518219	6.975733	-0.74547
H	5.210236	4.662928	0.053688
H	4.501679	5.892736	1.107663
H	3.3726	2.632674	-0.74998
H	-0.17895	4.638177	0.371166
H	1.751076	6.215855	0.846782
N	5.750692	5.394855	-2.74566
H	6.510522	5.288414	-2.05667
C	5.811225	4.505117	-3.88181
H	5.229359	4.920231	-4.71542
C	5.294334	3.125524	-3.4795
O	5.60726	2.61972	-2.39411
N	4.464386	2.498512	-4.32503
C	3.913709	1.202429	-3.96153
C	5.041154	0.1546	-3.90512
C	2.839549	0.767677	-4.95465
O	5.809987	0.012898	-4.84169
C	1.948779	-0.35128	-4.40193
C	0.87915	0.130244	-3.44415
O	-0.31452	-0.12676	-3.69504
O	1.24363	0.780247	-2.39799
H	4.294304	2.880749	-5.24811
H	3.472883	1.308607	-2.96389
H	2.215532	1.636102	-5.22631
H	3.337962	0.4184	-5.8727
H	2.558693	-1.10694	-3.88036
H	1.439977	-0.87107	-5.21828
N	5.063812	-0.55896	-2.75464
H	4.463074	-0.27064	-1.96943
C	6.039634	-1.5889	-2.47019
H	5.749777	-2.03219	-1.50569
C	6.070881	-2.70286	-3.54459
H	6.66155	-2.38853	-4.41061
H	6.54065	-3.59759	-3.1076
C	4.693711	-3.06443	-4.07581
O	3.736072	-3.36501	-3.1958
O	4.436788	-3.07701	-5.26147
C	7.460745	-1.03579	-2.26023
O	8.40645	-1.80016	-2.06644
N	7.601254	0.303899	-2.31304
H	6.779241	0.901657	-2.35917
C	8.909962	0.929265	-2.45893

H	9.566506	0.151232	-2.87862
C	8.852738	2.117436	-3.40737
H	9.86735	2.482826	-3.61741
H	8.29264	2.948204	-2.96169
H	8.376044	1.812621	-4.35036
C	9.493782	1.296849	-1.07982
O	9.668259	2.458218	-0.71559
N	9.794859	0.212387	-0.33575
H	9.44951	-0.68106	-0.69503
C	10.33437	0.271101	1.01775
H	11.28599	0.831446	0.975252
C	9.42856	1.00358	2.027334
H	9.500834	2.076706	1.811716
H	9.862475	0.819605	3.019693
C	7.976056	0.589236	2.008455
C	7.027758	1.397355	1.361317
H	7.355766	2.307054	0.855362
C	5.668946	1.074877	1.397004
H	4.940945	1.736983	0.923276
C	5.242247	-0.07235	2.070622
H	4.179628	-0.29155	2.15169
C	6.178488	-0.90864	2.685745
H	5.829632	-1.80363	3.205725
C	7.535652	-0.57719	2.65738
H	8.268805	-1.20789	3.160959
C	10.68013	-1.15317	1.501737
O	10.58526	-1.45904	2.679893
N	11.09632	-2.01686	0.538807
H	11.1572	-1.6864	-0.41663
C	11.17865	-3.45342	0.759492
H	11.30096	-3.60358	1.839954
C	9.941012	-4.18448	0.223269
H	9.878079	-4.00935	-0.86486
H	10.09235	-5.27077	0.358268
C	8.606852	-3.76613	0.861509
H	8.529566	-2.66739	0.789109
C	7.429858	-4.33662	0.069048
H	7.49509	-4.03666	-0.98751
H	7.408454	-5.43953	0.116033
H	6.476532	-3.96181	0.467801
C	8.510596	-4.14402	2.342443
H	9.293776	-3.65526	2.939309
H	7.537794	-3.833	2.756783
H	8.598679	-5.23637	2.475217
C	3.940391	-5.85521	5.440824
H	4.493364	-6.1358	6.353412
C	3.739207	-4.32897	5.422562

H	3.127535	-4.01065	6.284222
H	4.715564	-3.83551	5.515411
C	3.050049	-3.84171	4.144904
O	3.733657	-3.12611	3.365094
O	1.861772	-4.20766	3.938276
C	2.661908	-6.67787	5.392836
O	2.437912	-7.50969	4.512395
N	1.775574	-6.44971	6.394949
H	1.996807	-5.74515	7.086731
C	0.54935	-7.20663	6.536748
H	0.759992	-8.28669	6.498346
C	-0.53142	-6.96427	5.478147
O	-1.53983	-7.64755	5.479489
N	-0.26612	-5.99982	4.556458
H	0.606515	-5.46112	4.574767
C	-1.13876	-5.81244	3.424127
H	-2.11675	-6.23613	3.705107
C	-1.30955	-4.33232	3.041528
H	-2.1428	-4.24516	2.335334
H	-0.38685	-3.99182	2.562721
C	-1.59718	-3.43064	4.228484
O	-2.75142	-3.1589	4.578747
N	-0.51401	-2.94701	4.855927
H	0.433746	-3.17164	4.535744
H	-0.64061	-2.3265	5.646183
C	-0.69431	-6.62495	2.19164
O	-1.25309	-6.45065	1.103395
N	0.281901	-7.53023	2.39614
H	0.751176	-7.55251	3.303642
C	0.662843	-8.53514	1.426204
H	0.197872	-8.27887	0.465685
C	2.191084	-8.6671	1.268526
H	2.632551	-8.90881	2.246223
H	2.379618	-9.51395	0.588955
C	2.81621	-7.42187	0.720303
C	2.735692	-7.12219	-0.66852
H	2.262985	-7.84507	-1.33755
C	3.227821	-5.94337	-1.17289
H	3.152141	-5.69352	-2.23222
C	3.835049	-4.97168	-0.28444
O	4.203202	-3.82939	-0.69788
H	4.032003	-3.45625	-2.2431
C	3.959548	-5.31496	1.115166
H	4.424493	-4.58929	1.783555
C	3.442463	-6.49542	1.591544
H	3.477347	-6.73949	2.654019
C	-0.21325	7.805485	-3.75851

C	-0.20781	7.908918	-2.24417
C	-0.93818	6.541202	-4.26213
O	0.650093	7.341173	-1.56265
C	-0.39369	5.25119	-3.74027
C	-1.04205	4.135651	-3.25875
N	0.952271	4.925393	-3.71309
C	1.071801	3.672383	-3.23358
N	-0.11816	3.157399	-2.9401
H	-0.68547	8.691131	-4.2099
H	-0.89757	6.545896	-5.3654
H	-2.00462	6.587719	-3.99235
H	-2.11194	3.98895	-3.13952
H	2.033539	3.202242	-3.06022
H	1.76623	5.543175	-3.77662
N	-1.20107	8.637046	-1.69775
H	-1.95995	9.002578	-2.26999
C	-1.41974	8.767408	-0.27654
H	-0.78325	9.546394	0.179297
H	-1.16127	7.831005	0.242599
C	-2.88971	9.126737	-0.05099
O	-3.63287	9.315321	-1.02014
N	-3.27364	9.21262	1.229629
H	-2.5544	9.17638	1.967695
C	-4.65503	9.440028	1.614555
H	-5.13218	10.08476	0.86013
C	-5.44714	8.136184	1.762963
H	-5.42443	7.589665	0.807963
H	-4.94263	7.496317	2.504003
C	-6.89596	8.365568	2.186361
H	-7.42173	8.965058	1.425786
H	-6.93006	8.96347	3.116236
C	-7.67835	7.06591	2.392997
H	-8.74026	7.287433	2.586504
H	-7.63867	6.455353	1.483791
N	-7.16603	6.28108	3.511051
H	-7.23926	6.708505	4.426904
C	-6.4651	5.132089	3.449619
N	-6.2376	4.495424	2.308301
H	-6.57577	4.805145	1.375347
H	-5.75098	3.598231	2.393274
N	-5.96662	4.618649	4.586693
H	-6.23661	4.994785	5.484742
H	-5.47775	3.708685	4.521405
C	-5.51149	-0.06344	-3.4823
H	-6.51861	-0.42036	-3.73329
H	-5.47854	1.013722	-3.69779
N	-4.53522	-0.77403	-4.29216

H	-4.64759	-1.79886	-4.45354
C	-3.32469	-0.31607	-4.59354
N	-2.92195	0.912631	-4.21773
H	-3.324	1.328224	-3.37486
H	-1.93099	1.106055	-4.33474
N	-2.49428	-1.08165	-5.32191
H	-2.80804	-2.00417	-5.59635
H	-1.4885	-0.98328	-5.18866
O	-3.2115	1.717174	-1.54806
C	-2.82524	2.708924	-0.89421
O	-1.61824	2.898585	-0.52523
C	-3.77687	3.825907	-0.52579
H	-3.6603	4.020625	0.549153
O	-0.19523	0.549646	0.076447
O	-1.26828	-0.86091	2.062757
Fe	0.028001	1.87068	-1.08681
C	-2.50193	-2.11483	-1.71087
N	-1.89476	-0.76745	-1.56881
H	-1.28195	-0.60344	-0.73702
H	-1.28786	-0.54231	-2.39444
H	-2.58102	0.012677	-1.5246
C	-3.52563	-2.36459	-0.58823
O	-3.15551	-2.09269	0.601078
O	-4.61926	-2.82653	-0.93518
C	-1.43591	-3.21221	-1.82241
C	-0.5588	-3.46935	-0.58864
C	0.746069	-2.66856	-0.47525
C	1.355079	-2.9325	0.896115
N	2.648485	-2.26475	1.148355
H	2.530444	-1.24223	1.165949
H	3.058469	-2.59066	2.110242
H	3.34434	-2.48954	0.422214
H	-10.1216	-0.04621	0.538591
H	2.423063	9.057754	4.248647
H	2.652581	10.30076	-0.42472
H	6.858699	4.399344	-4.20079
H	12.08614	-3.83836	0.268155
H	-2.64498	8.665178	5.289007
H	-4.65401	9.996087	2.565459
H	0.838949	7.796107	-4.08072
H	-3.39287	4.726204	-1.03333
H	4.560001	-6.16793	4.58929
H	0.263075	-9.51793	1.731363
H	0.108013	-6.99807	7.521119
H	-3.64492	1.92855	5.732018
H	-6.79608	-1.98904	3.921022
H	0.717678	-7.24695	-3.04223

H	-2.58642	-9.51753	-1.62303
H	-6.91659	-2.4514	-5.31613
H	-0.86129	-0.31049	1.336937
H	-1.90457	-1.44161	1.573671
H	0.668655	-2.57773	1.671888
H	1.499961	-4.00778	1.06803
H	0.573611	-1.58667	-0.58486
H	1.451859	-2.96007	-1.27268
H	-1.1644	-3.30241	0.310919
H	-0.30504	-4.53921	-0.55279
H	-0.82545	-3.00139	-2.71547
H	-1.99973	-4.12813	-2.05264
H	-3.06713	-2.10919	-2.64807
H	-5.33991	-0.21696	-2.40355
O	1.973729	0.58418	1.538707
H	1.154537	0.503876	0.964039
H	1.722772	0.101069	2.380065
O	0.093903	2.420279	2.440511
H	-0.52986	2.029617	1.802826
H	0.953317	2.079509	2.10875
O	3.26048	-0.01212	-0.77476
H	2.468624	0.199124	-1.31002
H	3.080524	0.3615	0.111029
O	1.043092	-0.76455	3.594593
H	0.161217	-0.97348	3.215154
H	0.854709	-0.07759	4.272327
O	0.318812	1.501021	4.948329
H	0.162039	1.877573	4.044642
H	1.025038	2.042462	5.326164
O	7.427101	4.15724	-0.91548
H	6.865778	3.438164	-1.26358
H	8.314209	3.751395	-0.81174
C	-5.23917	3.627677	-0.90592
H	-5.29221	3.133506	-1.8867
H	-5.77082	2.981082	-0.19661
C	-5.92496	4.984227	-1.0401
O	-5.43364	5.772656	-1.87289
O	-6.92552	5.248902	-0.28804
C	-7.4146	8.562724	-1.3165
N	-8.24282	7.546957	-1.0457
N	-6.15632	8.311452	-1.63275
N	-7.85652	9.841891	-1.24096
H	-7.26732	10.54207	-1.67562
H	-5.86245	7.303944	-1.768
H	-5.40577	8.993867	-1.49796
H	-7.82966	6.603313	-0.85165
H	-9.24394	7.680938	-1.03479

C	-9.20512	10.22243	-0.86565
H	-9.50407	9.713122	0.063833
H	-9.23116	11.30312	-0.67614
H	-9.94873	9.995688	-1.65041
